# Direct electrochemical defluorinative carboxylation of α-CF_3_ alkenes with carbon dioxide†

**DOI:** 10.1039/d0sc04091f

**Published:** 2020-09-11

**Authors:** Xiao-Tong Gao, Zheng Zhang, Xin Wang, Jun-Song Tian, Shi-Liang Xie, Feng Zhou, Jian Zhou

**Affiliations:** Shanghai Key Laboratory of Green Chemistry and Chemical Processes, Shanghai Engineering Research Center of Molecular Therapeutics and New Drug Development, School of Chemistry and Molecular Engineering, East China Normal University 3663N Zhongshan Road Shanghai 200062 P. R. China fzhou@chem.ecnu.edu.cn; College of Chemistry, Sichuan University Chengdu Sichuan 610064 P. R. China; State Key Laboratory of Organometallic Chemistry, Shanghai Institute of Organic Chemistry, Chinese Academy of Sciences Shanghai 200032 P. R. China

## Abstract

An unprecedented γ-carboxylation of α-CF_3_ alkenes with CO_2_ is reported. This approach constitutes a rare example of using electrochemical methods to achieve regioselectivity complementary to conventional metal catalysis. Accordingly, using platinum plate as both a working cathode and a nonsacrificial anode in a user-friendly undivided cell under constant current conditions, the γ-carboxylation provides efficient access to vinylacetic acids bearing a *gem*-difluoroalkene moiety from a broad range of substrates. The synthetic utility is further demonstrated by gram-scale synthesis and elaboration to several value-added products. Cyclic voltammetry and density functional theory calculations were performed to provide mechanistic insights into the reaction.

## Abstract 2

A γ-carboxylation of α-CF_3_ alkenes with CO_2_ using platinum plate as both working cathode and nonsacrificial anode has been developed.

## Graphical abstract

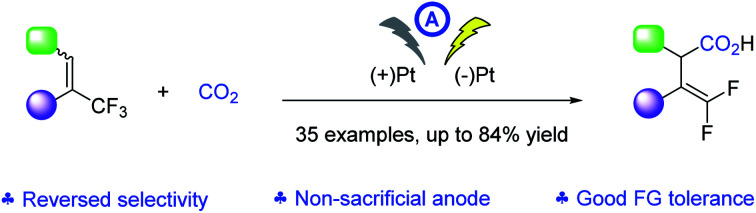

## Introduction

**Scheme 1:**
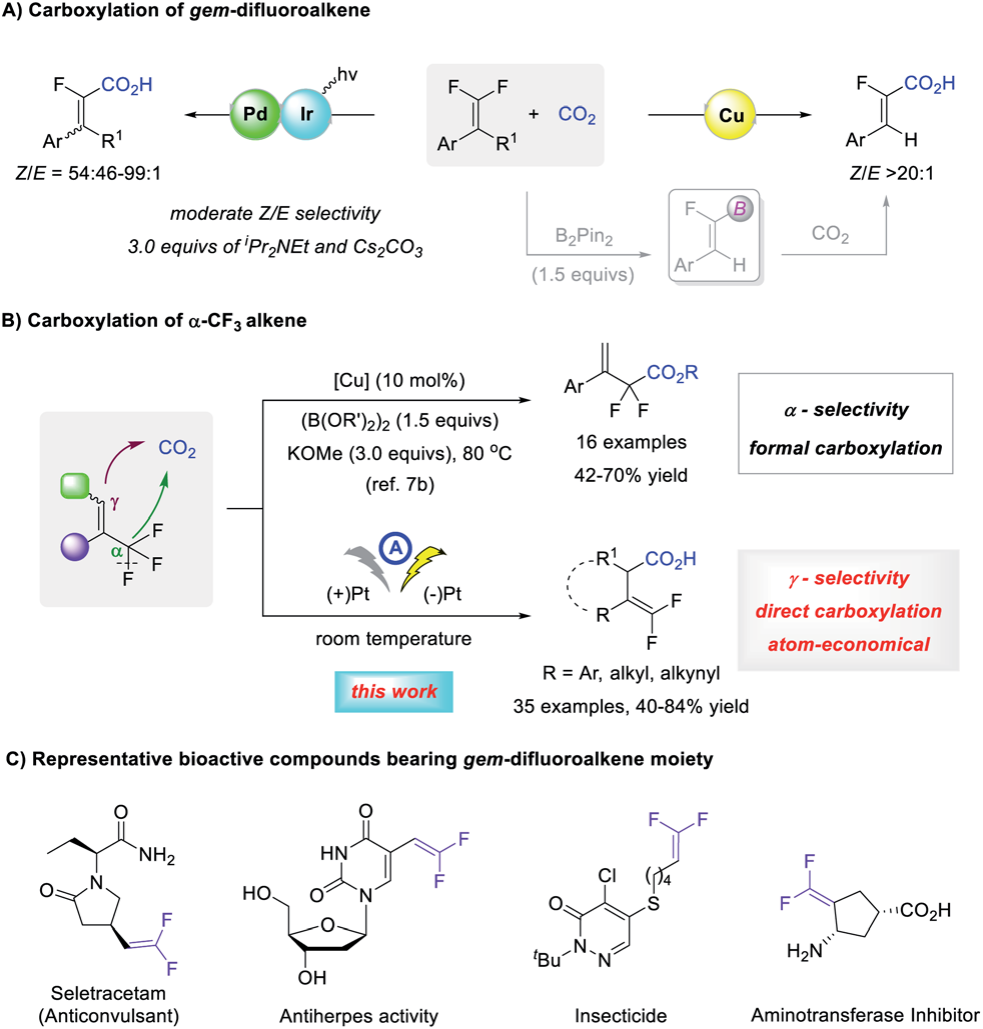
C–F bond carboxylation with CO_2_.

The carboxylation of organic halides using CO_2_ as an abundant and nontoxic C1 synthon^1^ is an important strategy to access carboxylic acids, which are widely distributed in natural products and pharmaceuticals.^2^ Despite good progress in the carboxylation of carbon–hetero bonds,^3^ the activation of C–F bonds for reaction development is largely undeveloped.^4^ This is possibly because the C–F bond is the strongest carbon–hetero bond with a high bond dissociation energy, the activation of which is kinetically unfavorable since fluorine is neither a good Lewis base nor a good leaving group.^5^ In this context, the selective C–F bond carboxylation of lightly fluorinated compounds could facilitate access to fluorinated carboxylic acids that are much sought-after substances for organic synthesis, materials science, and medicinal chemistry.^6^ However, to date, only a limited number of catalytic processes have been realized (Scheme 1A).^7^ Feng and co-workers combined photoredox/Pd dual catalysis to realize an sp^2^ C–F bond carboxylation of *gem*-difluoroalkenes with moderate *Z*/*E* selectivity.^7*a*^ A Cu-catalyzed formal carboxylation was reported by Yu^7*b*^ and by us^7*c*^ respectively, in which carboxylation of the vinylboronate intermediate yielded α-fluoroacrylic acids with high *Z*-selectivity. Yu and co-workers also achieved a formal sp^3^ C–F bond carboxylation of α-CF_3_ styrenes, which was conducted at 80 °C with 1.5 equivalents of diboron reagent and 3.0 equivalents of base, giving α,α-difluorocarboxylates regioselectively.^7*b*^ Because the C–F bond cleavage of α-CF_3_ alkenes might lead to both α- and γ-carboxylation, it is interesting to develop γ-carboxylation of α-CF_3_ alkenes using CO_2_ for the synthesis of carboxylic acids with a *gem*-difluoroalkene moiety. As a carbonyl bioisostere with less susceptibility to *in vivo* metabolism, *gem*-difluoroalkene is a prominent structural motif that is found widely in biologically active compounds (Scheme 1C).^8^ Moreover, they are versatile fluorinated building blocks in organic synthesis.^9^ Therefore, the efficient γ-carboxylation of α-CF_3_ alkenes using CO_2_ under mild conditions with broad substrate scope is highly desirable.

Recently, increasing attention has been paid to synthetic organic electrochemistry.^10^ Using electricity as a driving force, the use of toxic or expensive reducing agents might be avoided, and room temperature is often sufficient to promote the reaction. Interestingly, electrochemical processes facilitated access to high-energy species or new mediators, thus affording opportunities that conventional chemistry may not have achieved.^11^ Intrigued by these attractive features, we postulated that electrochemistry might be a powerful strategy to develop direct and green defluorinative carboxylation of α-CF_3_ alkenes to secure a level of γ-carboxylation unattainable by copper catalysis and avoid the use of diboron reagent and bases. Here, we wish to report our results in electrochemical γ-selective carboxylation of α-CF_3_ alkenes with CO_2_ to structurally diverse γ,γ-difluoro vinylacetic acids with good functional group tolerance, using platinum plate as both cathode and nonsacrificial anode under mild conditions (Scheme 1B).

Currently, most electrocarboxylations are conducted with sacrificial anodes and/or require (quasi-)divided cell to forestall the undesired oxidation of starting material or carboxylic acid products.^12^ From the viewpoint of practicability and sustainability, the development of non-sacrificial metal system is more desirable.^13^ Just recently, Malkov and Buckley achieved highly regioselective electrosynthetic hydrocarboxylation of β,β-trisubstituted alkenes and conjugated dienes using non-sacrificial anode system.^14^ Encouraged by these elegant advances in nonsacrificial metal-based electrochemical carboxylation of alkenes,^15^ along with the seminal study on electrochemical C–F bond carboxylation of benzotrifluoride by Troupel,^16^ we tried to develop electrochemical carboxylation of α-CF_3_ alkenes using a nonsacrificial anode, with our interest in chemical fixation of CO_2_ to value-added chemicals.^17^ Accordingly, the reaction of α-CF_3_ substituted styrene **1a** and bubbling CO_2_ was undertaken in DMF containing Et_4_NOTs to evaluate different nonsacrificial anodes, at a constant current of 8 mA in an undivided cell, with Pt-plate as cathode (Table 1).

**Table 1:** Condition optimization^a^

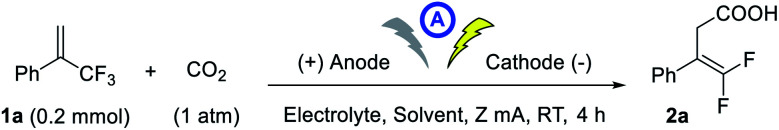						
Entry	Anode	Cathode	Electrolyte	Solvent	*Z* (mA)	Yield^b^ (%)
1	C	Pt	Et_4_NOTs	DMF	8	32
2	RVC	Pt	Et_4_NOTs	DMF	8	14
3	Pt	Pt	Et_4_NOTs	DMF	8	57
4	Pt	C	Et_4_NOTs	DMF	8	50
5	Pt	RVC	Et_4_NOTs	DMF	8	38
6	Pt	Pt	Et_4_NOTs	DMF	10	54
7	Pt	Pt	Et_4_NOTs	DMF	6	42
8	Pt	Pt	^*n*^Bu_4_NOTs	DMF	8	60
9	Pt	Pt	^*n*^Bu_4_NClO_4_	DMF	8	72
10	Pt	Pt	^*n*^Bu_4_NClO_4_	DMA	8	59
11	Pt	Pt	^*n*^Bu_4_NClO_4_	DCE	8	20
12	Pt	Pt	^*n*^Bu_4_NClO_4_	THF	8	70
13^c^	Pt	Pt	^*n*^Bu_4_NClO_4_	DMF	8	83
14^c^^,^^d^	Pt	Pt	^*n*^Bu_4_NClO_4_	DMF	8	82

a Reaction conditions: electrolyte (0.06 M), CO_2_ bubbling in solvent (6 mL).

b Isolated yield.

c With ^*n*^Bu_4_NClO_4_ (0.07 M), DMF (7 mL).

d 7 hours.

## Results and discussion

First, we evaluated graphite, RVC and Pt anodes, and found that the reaction indeed proceeded at room temperature for 4 hours, with a total charge of 6 Faraday/mol to yield the γ-carboxylation product **2a** in 32, 14, and 57% yields, respectively, and no α-carboxylation adducts were detected (entries 1–3). This result unambiguously supported our working hypothesis and encouraged us to conduct further optimization studies using the Pt anode. Varying the cathode from Pt-plate to graphite or RVC gave no better results (entries 4 and 5). Increasing the current to 10 mA resulted in almost no change in yield but decreasing the current to 6 mA led to a reduction in the yield to 42% due to incomplete reaction (entries 6 and 7). Since the supporting electrolyte could affect the local environment near the electrode as part of the electrical double-layer,^18^ we next evaluated its influence. Using ^*n*^Bu_4_NClO_4_ as the electrolyte, the yield of **2a** improved significantly to 72% (entry 9). The solvent effects were also investigated, but DMF still proved to be the solvent of choice (entries 10–12 *vs.* 9). Finally, by increasing the electrolyte concentration to 0.07 M and performing the reaction with an ElectraSyn 2.0 instrument (see ESI†), the desired carboxylic acid, **2a** could be obtained in 83% yield (entry 13). To investigate the stability of the carboxylation product under constant current conditions in an undivided cell,^19^ we extended the reaction time to 7 hours and found that almost the same yield of **2a** was obtained as that after 4 hours (entry 14 *vs.* 13). This indicated the carboxylation product was sufficiently stable and did not decompose during the reaction (for detail of optimization, see ESI†).

Having established the optimal reaction conditions, we next evaluated the scope of the reaction with respect to α-CF_3_ styrene derivatives. With various substituents on the phenyl rings, including methyl, *tert*-butyl, phenyl, terminal alkene, halogen, ether, cyano, and ester groups, alkenes **1b–r** readily afforded the corresponding γ,γ-difluoro vinylacetic acids, **2b–r** in 45–84% yields. The electronic and steric effects of the phenyl substituents had little impact on the carboxylation. For instance, the reaction of isomeric substrates with a methyl, chloro, fluoro, or OCF_3_ group at the *para*- or *meta*-position proceeded smoothly to afford the corresponding products **2b**, **2c**, **2j–l** and **2p–r** in similarly good to high yields. The α-CF_3_ alkene derivatives bearing 2-naphthyl, 2-thiophenyl, or 2-benzofuranyl group also worked well to furnish **2s–u** in 40–62% yields.

Notably, α-CF_3_ alkenes with α-alkyl substituents were also viable substrates. For example, alkenes bearing an α-phenethyl, phenylpropyl, or *n*-nonyl group produced the γ-carboxylation products **3a–c** in 46–57% yields. Furthermore, alkenes with an α-alkynyl moiety worked well to give the desired acids, **3d** and **3e** in 48 and 65% yields, respectively. Trisubstituted alkenes were further investigated, furnishing corresponding acids **3f–i** in 40–56% yields. Cyclic alkenes based on 1,2-dihydronaphthalene skeleton were amenable, giving **3j** and **3k** in 52 and 55% yields, respectively. To our delight, several complex substrates derived from β-d-glucose, estrone and fructose also reacted well, affording the desired adducts **4a–c** in reasonable yields. These results clearly demonstrated the good functional group tolerance of our method. For some substrates indicated in Table 2, the addition of H_2_O was beneficial for the reaction yield, but the reason for this is not clear.^20^

**Table 2:** Substrate scope of the electrochemical defluorinative carboxylation^a^

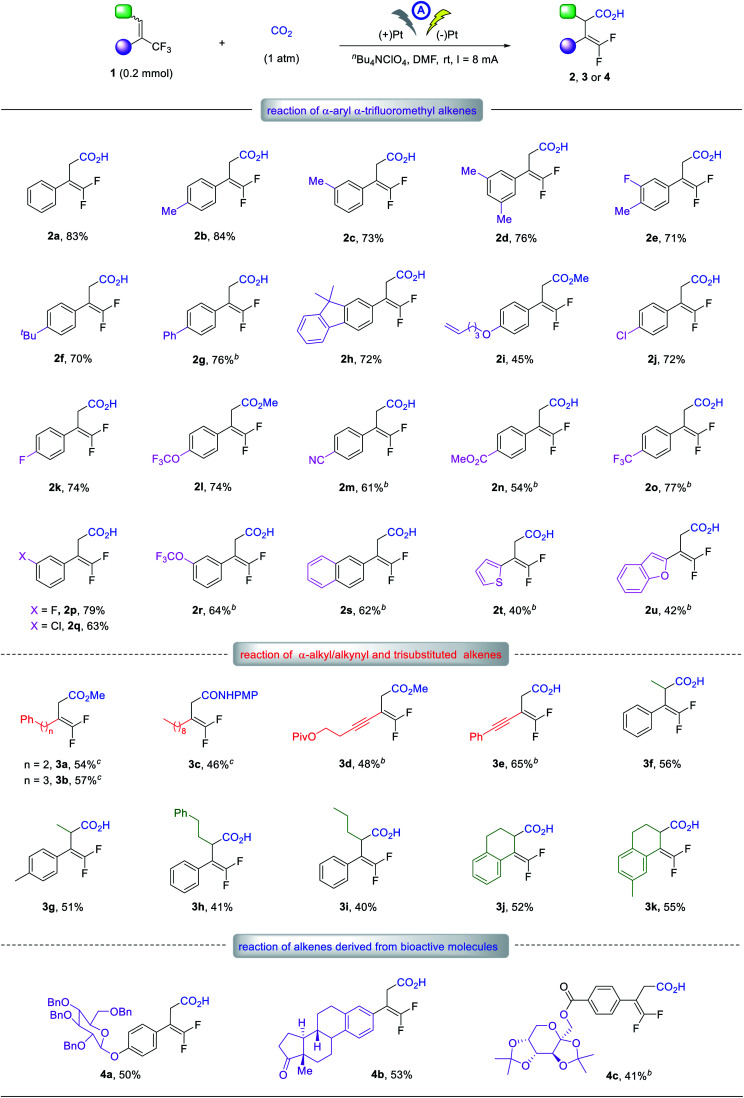

a With ^*n*^Bu_4_NClO_4_ (0.07 M), CO_2_ bubbling in DMF (7 mL), 4–7 h, isolated yield.

b 200 μL H_2_O as additive.

c Graphite as the anode.

Given that a wide range of functional groups are tolerated, such as alkene, alkyne, and halogen groups, this methodology should be orthogonal to classical cross-coupling chemistry, which would further extend its synthetic utility. Also noteworthy was the perfect regioselectivity: only γ-carboxylation occurred for all the reactions discussed above, and no α-carboxylation products were detected. In addition, for substrates bearing a fluoro, CF_3_, or OCF_3_ group, possessing different sp^2^ or sp^3^ C–F bonds, the defluorinative carboxylation occurred at the α-CF_3_ alkenes moiety exclusively.

**Scheme 2:**
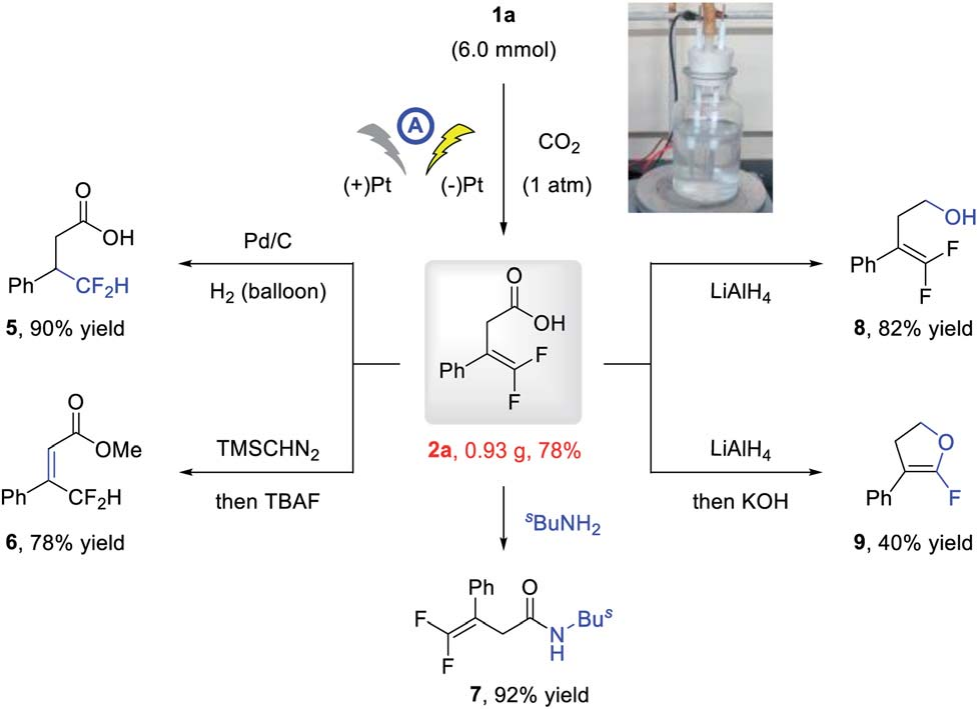
Synthetic elaboration of **2a**.

To further demonstrate the practicability of the developed electrochemical carboxylation, a gram-scale reaction of **1a** was conducted on a 6.0 mmol scale, and the product **2a** was isolated in 0.93 g with comparable yield (78%; Scheme 2). Moreover, the thus obtained carboxylic acid could be readily elaborated to valuable fluorine-containing molecules. Under Pd/C catalysis, the alkene moiety of **2a** could be readily hydrogenated to give β-difluoromethyl carboxylic acid **5** in 90% yield. The methylation and subsequent TBAF promoted olefin isomerization delivered **6** in 78% yield. Condensation with amine afforded direct access to amide **7** in 92% yield, and reduction of the carboxylic acid moiety with LiAlH_4_ delivered alcohol **8** in 82% yield. The α-fluoro dihydrofuran **9** could be obtained in 40% yield *via* LiAlH_4_-mediated reduction and base-promoted defluorinative cyclization.

**Fig. 1:**
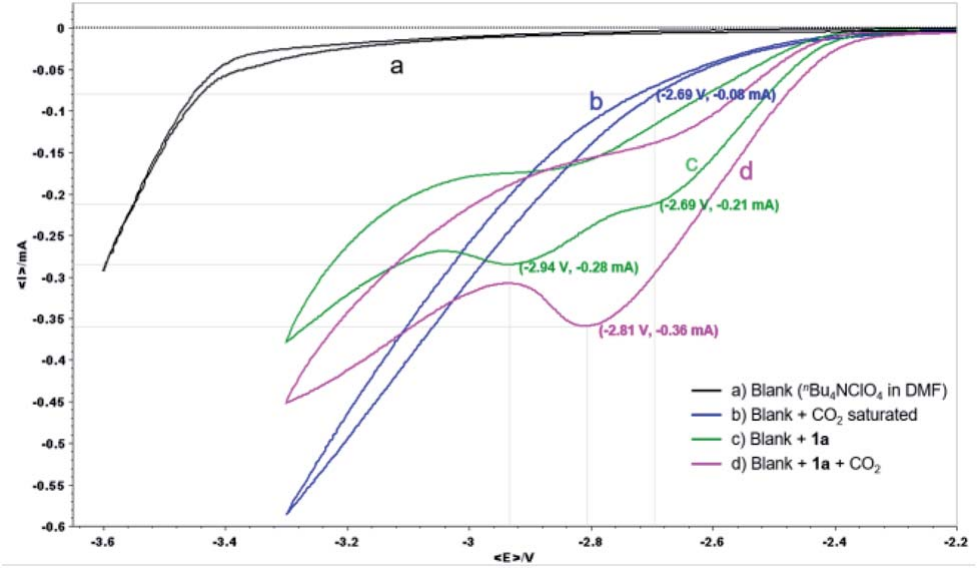
CV analysis of **1a**. ^*n*^Bu_4_NClO_4_ (0.07 M) in DMF as electrolyte and a 100 mV s^−1^ scan rate using a Pt disk WE and Pt pillar CE along with Ag/AgNO_3_ as RE.

The reaction mechanism was then studied experimentally and computationally to shed light on the two attractive features of our protocol: the γ-carboxylation complementary to the α-carboxylation obtained by copper catalysis,^7*b*^ and the obviation of a sacrificial anode. First, cyclic voltammetry (CV) analyses were conducted to investigate the electrochemical process on the cathode (Fig. 1).

For the CV of α-CF_3_ styrene **1a**, a one-electron reduction peak in the potential at −2.69 V and a second at −2.94 V was observed (green line), whereas at a potential of −2.69 V, the reduction current of CO_2_ was less than 0.1 mA (blue line), indicating that **1a** is easier to reduce than CO_2_. After the solution of **1a** was saturated with CO_2_ (pink line), only one reduction peak was observed at −2.81 V with an associated peak current increase from 0.21 to 0.36 mA (*ca.* 1.7 times). The influence of potential on the reaction was further studied by constant potential electrolysis. When the reaction was conducted with cathode potential less than −2.70 V, the yield decreased gradually (Table S7 in ESI†). These results suggested that an ECEC process might be involved, in which a radical anion that could react immediately with CO_2_ might be generated after the first one-electron electroreduction, then the second electron transfer is facilitated at a less negative potential thus leading to a significant increase in current observed. Accordingly, since a different species is being reduced in the presence of CO_2_, the second peak at −2.94 V is not observed.^21^ Due to the higher stability of the tertiary alkyl radical, after the first one-electron electroreduction, carboxylation at the less substituted carbon of the alkene moiety should be favored.

**Scheme 3:**
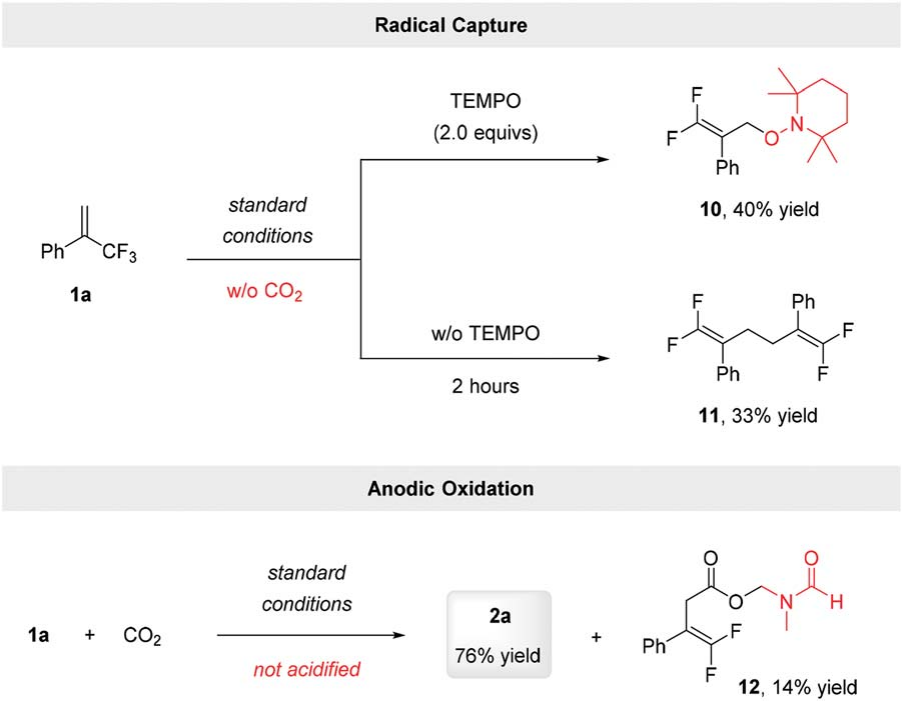
Control experiments. Standard conditions: constant current electrolysis under 8 mA using Pt-plate as cathode and anode with ^*n*^Bu_4_NClO_4_ (0.07 M) in DMF, 7 hours.

To gain more evidence for the intermediacy of a radical anion, we subjected **1a** to electrocarboxylation conditions in the presence of several known radical traps. Unfortunately, we were unable to trap the putative radical anion generated *via* the single-electron reduction or tertiary alkyl radical formed after the addition of CO_2_. This might be because the radical reduction and subsequent defluorination are favored under electroreduction conditions. However, the addition of 2.0 equivalents of TEMPO to the reaction in the absence of CO_2_ led to the formation of TEMPO adduct **10**, in 40% yield. Furthermore, when the reaction was conducted in the absence of TEMPO and CO_2_, the allylic radical dimerization product **11**, was obtained in 33% yield (Scheme 3). These results suggested that the radical anion was involved during the reaction and that its defluorination produced the allylic radical.

**Fig. 2:**
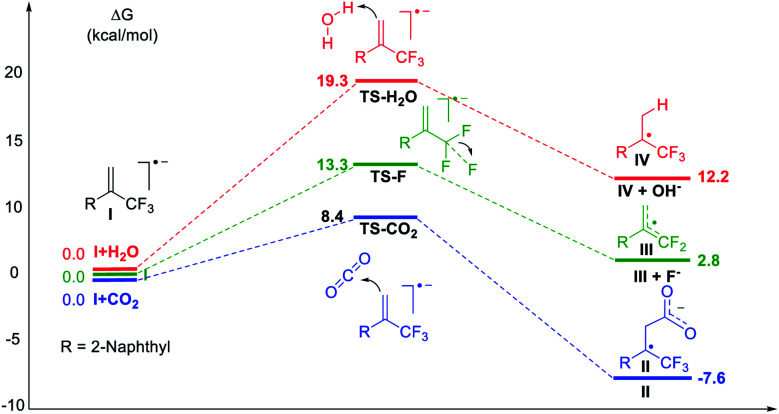
DFT calculations. Gibbs free-energy profile for possible reaction channels at the IEFPCM-M06-2X/6-311++G(d,p) level in solvent DMF.

Subsequently, density functional theory (DFT) calculations were performed as shown in Fig. 2. The results revealed that the reaction of CO_2_ with radical anion **I**, generated *via* one-electron reduction of α-CF_3_ alkene, was thermodynamically spontaneous with a low free-energy barrier of 8.4 kcal mol^−1^. Defluorination or protonation of radical anion **I** had higher free energy barriers of 13.3 and 19.3 kcal mol^−1^, respectively. These results are consistent with the experimental data and give a good explanation for the high regioselectivity and chemical selectivity of the reaction process.

To identify the sacrificial reductant on the anode, we analyzed the reaction mixture of **1a** with CO_2_ directly without acidification, and detected a DMF-protected carboxylate **12** (Scheme 3). This result suggested that a Shono oxidation of DMF might occur.^22^ Considering that water has a lower oxidation potential than DMF (1.23 and 1.9 V *vs.* SHE, respectively),^23^ it was more likely to act as sacrificial reductant.^14*b*^ Inspired by Chen's work,^24^ the capture of oxygen generated *via* the potential anode oxidation of H_2_O was conducted using labeled H_2_^18^O as additive. However, probably due to oxygen exchange of H_2_^18^O with CO_2_*via* the formation of H_2_CO_3_, only the release of ^16^O_2_ was detected (for details, see ESI†).

**Fig. 3:**
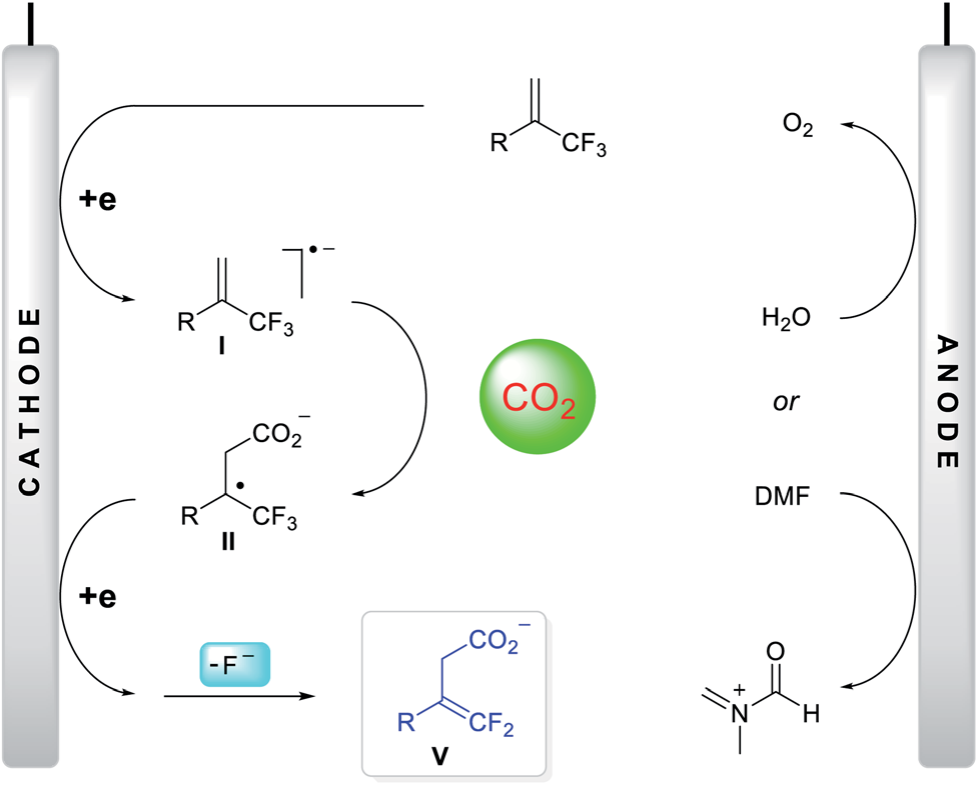
Proposed reaction mechanism.

Based on the above investigation of the mechanism, a putative reaction pathway was proposed, as shown in Fig. 3. Initially, a one-electron reduction of α-CF_3_ alkene generated the corresponding radical anion **I**, which reacted immediately with CO_2_ at the γ-position to give tertiary alkyl radical **II**. The secondary, one-electron reduction was then followed by a defluorination process to form carboxylate anion **V**. Meanwhile, the oxidation of DMF or H_2_O occurred at the anode, delivering the imine cation or hydrogen cation, both of which can interact with carboxylate anion **V** to yield the protected carboxylates or deliver the carboxylic acids directly.

## Conclusions

In summary, we have developed a regioselective electrochemical γ-carboxylation of α-CF_3_ alkenes using CO_2_ in a user-friendly undivided cell under constant current conditions, without the sacrifice of the anode. Both di- and trisubstituted α-CF_3_ alkenes work well to afford structurally diverse vinylacetic acids bearing a *gem*-difluoroalkene moiety in acceptable yields under mild conditions, with good tolerance of functional groups. Notably, this protocol constitutes a rare example of using an electrochemical process to secure regioselectivity that differs from that of the metal-catalyzed process, suggesting the potential of the electrochemistry approach for divergent synthesis. The application of this atom-economical electrochemical method for the synthesis of a diverse range of fluorine-containing carboxylic acids from well-known greenhouse gases CO_2_ and hydrofluorocarbons,^25^ is now in progress.

## Conflicts of interest

There are no conflicts to declare.

## Supplementary Material

SC-011-D0SC04091F-s001
