# Supplementary material for: Direct electrochemical defluorinative carboxylation of α-CF3 alkenes with carbon dioxide
Source: Chem Sci. 2020 Sep 11;11(38):10414–20. doi: 10.1039/d0sc04091f (PMC8162267; doi:10.1039/d0sc04091f)
Supplement: SC-011-D0SC04091F-s001 [file SC-011-D0SC04091F-s001.pdf]

Electronic Supplementary Material (ESI) for Chemical Science.
This journal is © The Royal Society of Chemistry 2020

Direct electrochemical defluorinative carboxylation of α-CF_3 alkenes with carbon dioxide

Xiao-Tong Gao,[a] Zheng Zhang,[a] Xin Wang,[b] Jun-Song Tian,[a] Shi-Liang Xie,[a] Feng Zhou *[a] and Jian Zhou[ac]

[a] Shanghai Key Laboratory of Green Chemistry and Chemical Processes; Shanghai Engineering Research Center of Molecular Therapeutics and New Drug Development, School of Chemistry and Molecular Engineering, East China Normal University, 3663N Zhongshan Road, Shanghai 200062, P. R. China.

[b] College of Chemistry, Sichuan University, Chengdu, Sichuan 610064, P. R. China.

[c] State Key Laboratory of Organometallic Chemistry, Shanghai Institute of Organic Chemistry, Shanghai 200032, China.

E-mail: fzhou@chem.ecnu.edu.cn

Supporting Information

Table of Contents	Page
1. General information	1
2. Reaction condition optimization	2-5
3. Photographic guide for electrochemical reaction	6-9
4. General procedure for the electrolysis	10-22
5. Product elaboration	23-25
6. Mechanistic studies	26-45
7. NMR spectra	46-174
8. References	175

1. General information

Reactions were monitored by thin layer chromatography using UV light, I_2 or $KMnO_4$ to visualize the course of reaction. Purification of reaction products was carried out by flash chromatography on silica gel. Chemical yields refer to pure isolated substances. Infrared (IR) spectra were obtained using a Bruker tensor 27 infrared spectrometer. ^1H NMR, ^{13}C NMR and ^{19}F NMR spectra were obtained using Bruker DPX 400 or 500 MHz Spectrometer. Chemical shifts were reported in ppm with TMS as the internal standard. The following abbreviations were used to designate chemical shift multiplicities: s = singlet, d = doublet, t = triplet, q = quartet, h = heptet, m = multiplet, br = broad.

Unless mentioned, all reactions were performed under an atmosphere of N_2 and carried out with dry solvents. $^nBu_4NClO_4$ were purchased from Macklin and used as received. α-Aryl α-trifluoromethyl alkenes were prepared by Suzuki cross coupling reaction of the corresponding arylboronic acid with 2-bromo-3,3,3-trifluoropropene.[1,2] α-Alkyl α-trifluoromethyl alkenes were prepared by Wittig olefination of the corresponding ketone.[1] α-Alkynyl α-trifluoromethyl alkenes were prepared by Sonagashira coupling of the corresponding alkyne.[3,4] Trisubstituted alkenes were prepared by Wittig olefination of the corresponding ketone.[3,5] Substrates derived from bioactive compounds, such as fructose, β-D-glucose and estrone were prepared according to reported procedure.[2,6,7]

List of abbreviation:

Entry	Chemical name	Abbreviation
1	Petroleum ether	PE
2	Ethyl acetate	EtOAc
3	Tetrahydrofuran	THF
4	*N,N*-Dimethylformamide	DMF
5	*N,N*-Dimethylacetamide	DMA
6	*N*-methyl-2-pyrrolidone	NMP
7	Dimethyl sulfoxide	DMSO
8	Hexamethyl phosphoryl triamide	HMPA

2. Reaction condition optimization

The reaction of α-trifluoromethyl styrene **1a** with CO_2 was selected as model reaction for condition optimization. We first examined the influence of electrode on the carboxylation of **1a**, by conducting the reaction in DMF containing Et_4NOTs at constant current of 8 mA in an undivided cell under room temperature with CO_2 bubbling, as shown in Table S1. Of several typical non-sacrificial anode screened (entries 1-4), Pt-plate turned out to be the best, affording the desired γ-carboxylation product **2a** in 57% yield (entry 1). The performance of sacrificial anode was also studied, and Mg-plate gave a slightly higher 60% yield (entries 5-7). Aiming to develop a non-sacrificial metal system, Pt-plate was selected as the anode. Further variation of cathode failed to give better results than that of Pt-plate (entries 8-12).

Table S1. Screening of anode and cathode

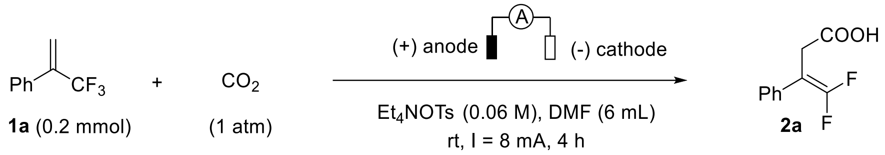

Entry	Anode	Cathode	Isolated yield (%)
1	graphite	Pt	32
2	RVC	Pt	14
3	Pt	Pt	57
4	glassy carbon	Pt	45
5	Mg	Pt	60
6	Fe	Pt	41
7	Ni	Pt	44
8	Pt	graphite	50
9	Pt	RVC	38
10	Pt	glassy carbon	44
11	Pt	Ni	43
12	Pt	Foam Ni	28

The influence of current intensity on the reaction was then studied, as shown in Table S2. It was found that under the current lower than 8 mA with the total charge less than 6 F/mol, the reaction yield decreased gradually due to the incomplete reaction. However, by increasing the current higher than 8 mA, there was almost no further influence on the reaction yield.

Table S2. Influence of electric current

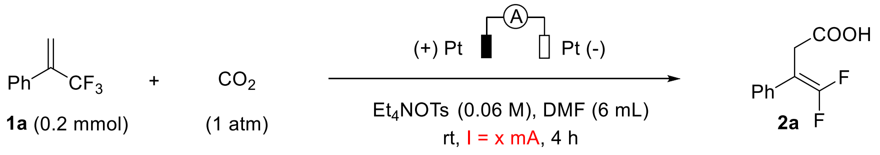

Entry	I (mA)	F/mol	Isolated yield (%)
1	4	3.0	30
2	6	4.5	42
3	8	6.0	57
4	9	6.7	50
5	10	7.5	54
6	11	8.2	53

The supporting electrolyte is another important parameter in affecting the reaction outcome, as it could influence the local environment near the electrode as part of the electrical double-layer. By changing the electrolyte from Et_4NOTs to $^nBu_4NOTs$, a slightly higher 60% yield was obtained (Table S3, entry 1). Further varying the counter anions in the tetrabutylammonium family had a positive impact on the reaction (entries 2-5), and 72% yield could be achieved by using $^nBu_4NClO_4$ as electrolyte (entry 5). Other perchlorate, such as Et_4NClO_4, was also tried, but no better result was obtained (entry 6).

Table S3. Screening of supporting electrolyte

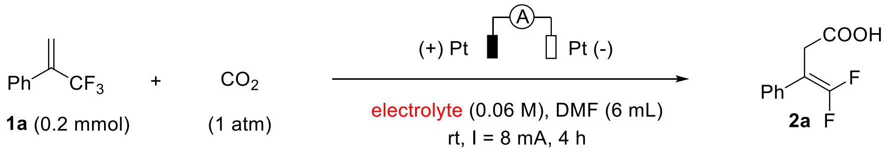

Entry	Electrolyte	Isolated yield (%)
1	$^nBu_4NOTs$	60
2	$^nBu_4NPF_6$	62
3	$^nBu_4NBr$	27
4	$^nBu_4NBF_4$	53
5	$^nBu_4NClO_4$	72
6	Et_4NClO_4	61

With $^{n}Bu_4NClO_4$ as electrolyte, the solvent effects were then examined with typical results shown in Table S4. The reaction conducted in DMA, NMP and HMPA gave lower yield than that in DMF (entries 1-4). No reaction occurred by using DMSO or CH_3CN as solvent (entries 5-6). DCE and THF could give 20% and 70% yield, respectively (entries 7-8). DMF was finally selected, not only because it could give higher reaction yield, but also due to its less volatility, especially under bubbling CO_2 condition. Further study revealed that the volume of solvent could also affect the reaction outcome. By performing the 0.2 mmol scale reaction in 7.0 mL DMF, 81% yield could be achieved (entries 9-12).

Table S4. Screening of solvent.

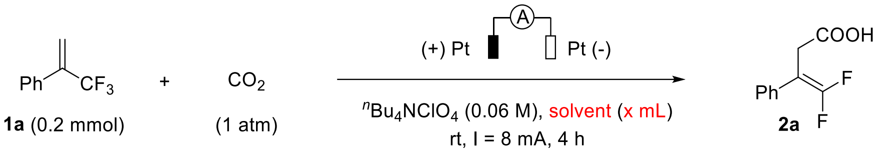

Entry	Solvent	x (mL)	Isolated yield (%)
1	DMF	6.0	72
2	DMA	6.0	59
3	NMP	6.0	69
4	HMPA	6.0	44
5	DMSO	6.0	Trace
6	CH_3CN	6.0	Trace
7	DCE	6.0	20
8	THF	6.0	70
9	DMF	3.0	56
10	DMF	5.0	63
11	DMF	7.0	81
12	DMF	9.0	78

Subsequently, the concentration of electrolyte was studied to further improved the reaction outcome. As shown in Table S5, by increasing the concentration of $^{n}Bu_4NClO_4$ to 0.07 M, the desired carboxylic acid **2a** could be obtained in 83% isolated yield (entry 3). Further increasing or decreasing the concentration gave no better results. Finally, we determined to perform the carboxylation of **1a** (0.2 mmol) under room temperature in DMF (7.0 mL) containing $^{n}Bu_4NClO_4$ (0.07 M) at constant current of 8 mA in an undivided cell with CO_2 bubbling and Pt-plate as both cathode and anode.

Table S5. Screening of electrolyte concentration

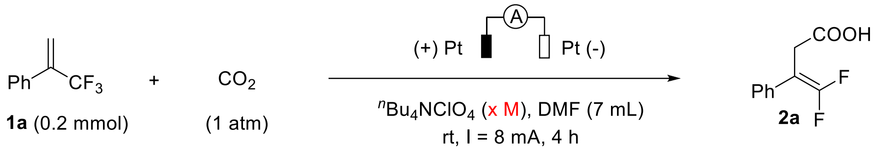

Entry	x (M)	Isolated yield (%)
1	0.05	76
2	0.06	81
3	0.07	83
4	0.08	79
5	0.09	79

During the investigation of substrate scope, we found that under the established optimal reaction condition for the reaction of **1a**, the carboxylation of **1s** bearing a 2-naphthyl gave only 23% yield. Considering that H_2O possessing an oxidation potential of 1.23V vs. SHE,[8] which might be utilized as sacrificial oxidant to avoid unexpected oxidation, we tried using H_2O as additive to improve the reaction outcome. Fortunately, as shown in Table S6, the addition of 200 μL H_2O in 7 mL DMF could promote the carboxylation of **1s** to give the desired **2s** in 62% yield. Consequently, water was added for some substrates to improve the reaction yield.

Table S6. Influence of H_2O as additive for the reaction of 1s

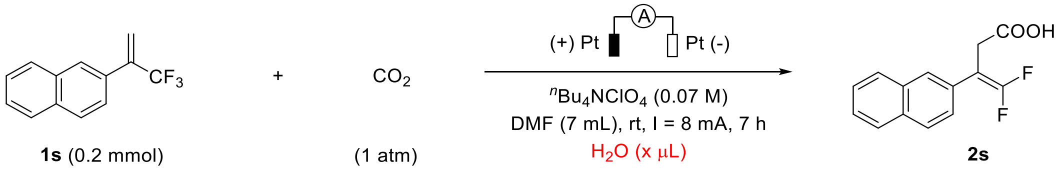

Entry	H_2O (x μL)	Isolated yield (%)
1	---	23
2	80	48
3	120	50
4	200	62
5	400	44

3. Photographic guide for electrochemical reaction

Electrode electrochemical reactions were carried out using the following three kinds of equipment,

- In a 10 mL hydrogenation tube using Pt-plate (10 mm*10 mm*0.2 mm) as anode and cathode with the current supplied from a 36 V constant-current power supply purchased from Xiamen Bodong Biotechnology Ltd. (for 0.2 mmol scale).
- In a 10 mL electrochemical cell using corresponding Pt-plate as anode and cathode with the current supplied from an ElectraSyn 2.0 instrument purchased from IKA®-Werke GmbH & CO. KG. (for 0.2 mmol scale).
- In a 250 mL wild-mouth bottle using Pt-plate (30 mm*30 mm*0.1 mm) as anode and cathode with the current supplied from a 36 V constant-current power supply purchased from Xiamen Bodong Biotechnology Ltd. (for 6.0 mmol gram-scale reaction).

3.1 Photographic guide for first kind of equipment (0.2 mmol)

Step 0. Overview of materials used.

From left to right: 1) two Pt plates [anode and cathode, 10 mm*10 mm*0.2 mm]; 2) a 10 mL hydrogenation tube; 3) a rubber stopper; 4) two hypodermic needles (the longer one for intake, the shorter for exhaust)

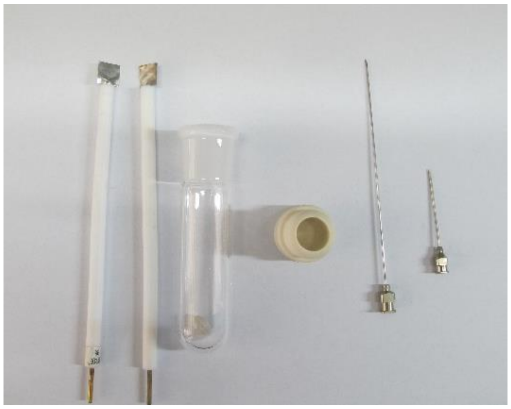

Step 1. Assembling the cell

1) Pierce the rubber stopper with two Pt plates; 2) The stopper was fitted into the tube; 3) Pierce the rubber stopper with the two hypodermic needles (the longer one for intake, the shorter for exhaust).

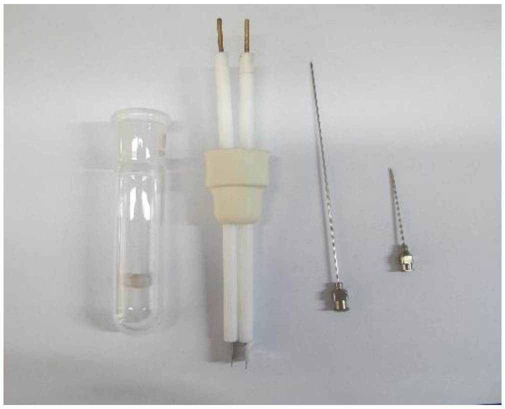
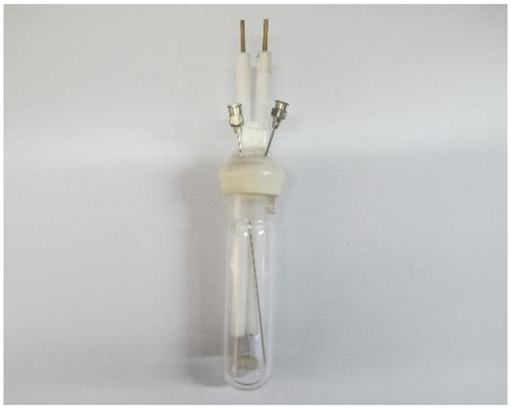

Step 2. Electrolysis

After the addition of all materials, CO_2 was bubbled for 10 mins and then conducted constant current electrolysis (I = 8.0 mA) using a constant-current power supply under continuous bubbling of CO_2.

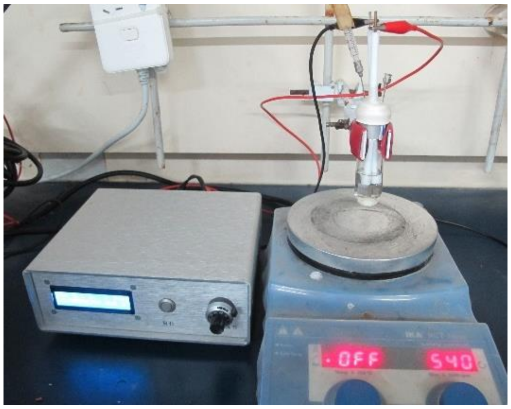

3.2 Photographic guide for the second kind of equipment (0.2 mmol, ElectraSyn 2.0)

Step 0. Overview of materials used.

From left to right: 1) electrochemical cell; 2) two Pt plates [anode and cathode]; 3) a hypodermic needle for intake; 4) cap.

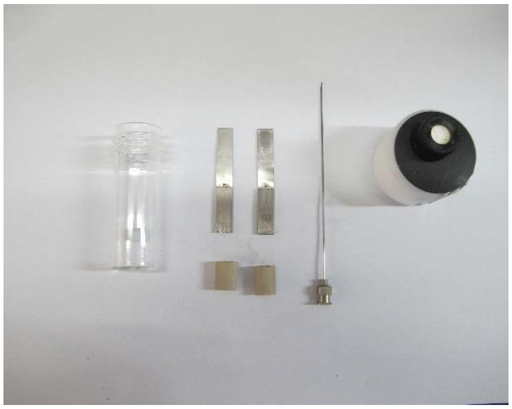

Step 1. Assembling the cell

1) Install two Pt plates to the cap; 2) The cap was fitted into the cell; 3) Pierce the rubber of cap with the hypodermic needle for intake.

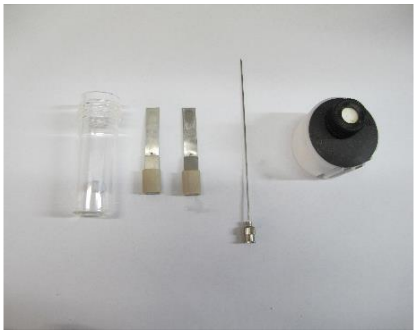
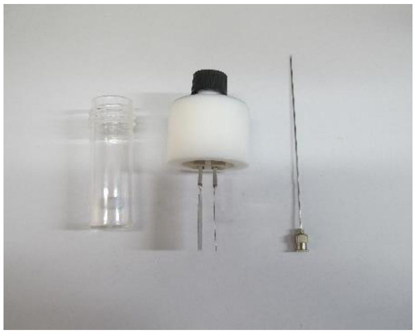
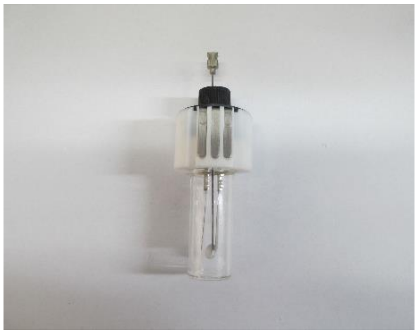

Step 2. Electrolysis

After the addition of all materials, CO_2 was bubbled for 10 mins and then conducted constant current electrolysis (I = 8.0 mA) using ElectraSyn 2.0 under continuous bubbling of CO_2.

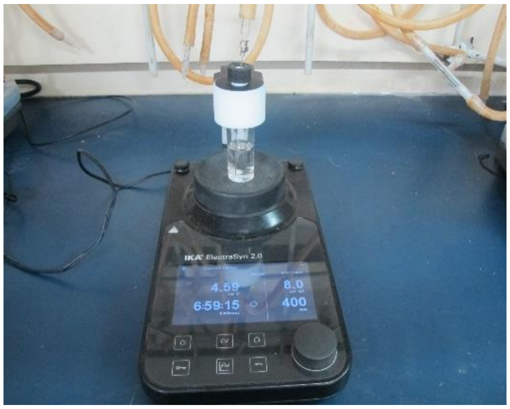

3.3 Photographic guide for the third kind of equipment (for 6 mmol gram-scale reaction)

Step 0. Overview of materials used.

From left to right: 1) 250 mL wild-mouth bottle; 2) a rubber stopper with three holes; 3) a glass tube for intake; 4) two Pt plates [anode and cathode, 30 mm*30 mm*0.1 mm]

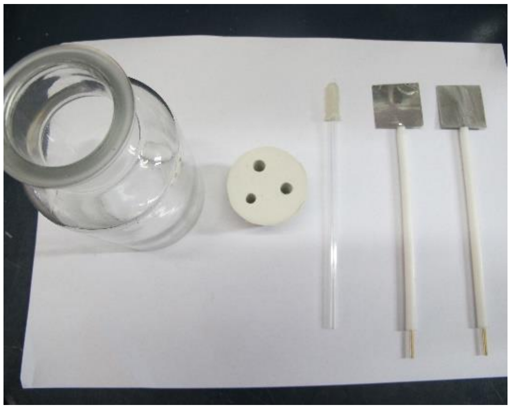

Step 1. Assembling the cell

1) Install two Pt plates and the glass tube to the rubber stopper; 2) The rubber stopper was fitted into the bottle.

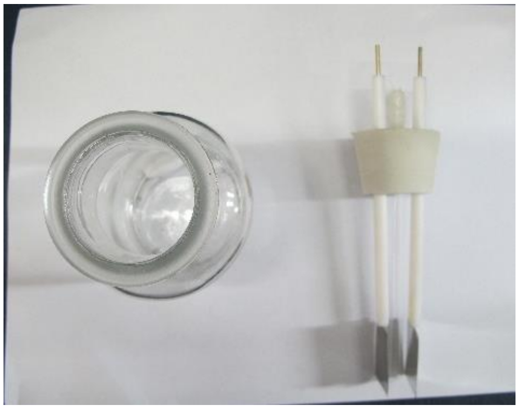

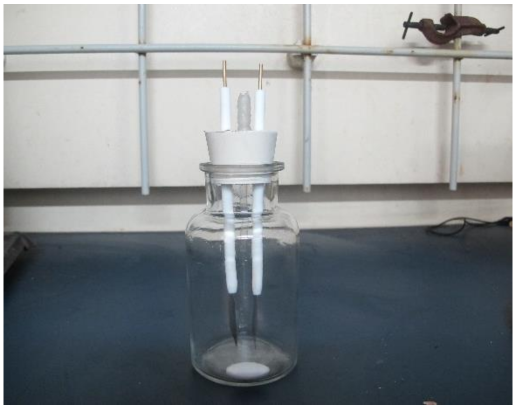

Step 2. Electrolysis

After the addition of all materials, CO_2 was bubbled for 10 mins and then conducted constant current electrolysis (I = 72.0 mA) using a constant-current power supply under continuous bubbling of CO_2.

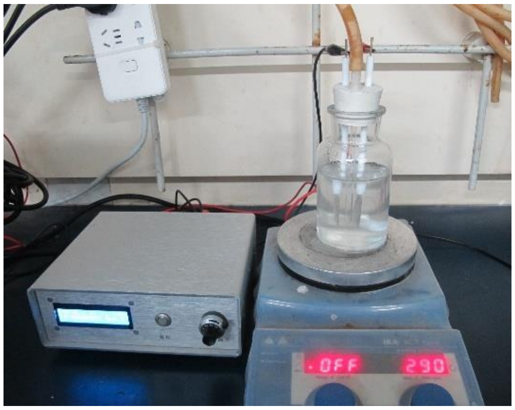

4. General Procedure for the Electrolysis

4.1 General procedure A. (for synthesis of acids **2a-2h**, **2j-2k**, **2m-2u**, **3e-3k** and **4a-4c**.)

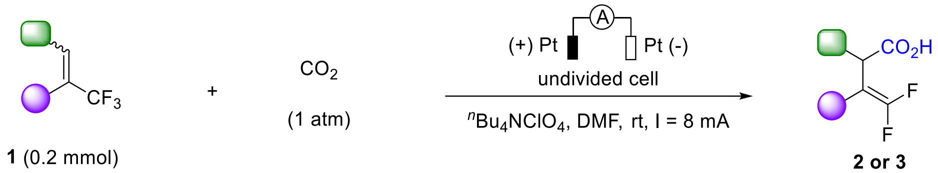

To a 10 mL hydrogenation tube or a cell of ElectraSyn 2.0 containing a stir bar were added ^{n}Bu$_4$NClO$_4$ (167.5 mg, 0.49 mmol) and DMF (7.0 mL), followed by the addition of **1** (0.2 mmol). Then the tube was installed with two Pt-plates as cathode and anode. After bubbling of CO_2 gas into the electrolytes for 10 mins, the reaction mixture was electrolyzed under a constant current of 8 mA until the complete consumption of the starting materials as monitored by TLC (4-7 hours). After that, the reaction mixture was transferred to a 50 mL Erlenmeyer flask and acidized with HCl (2 N, 10 mL). The aqueous layer extracted with EtOAc (2 x 20 mL) and the combined organics were washed with sat. NH_4Cl (2 x 20 mL), dried over Na_2SO_4, and concentrated in vacuo. The crude product was purified by column chromatography using PE/EtOAc (3:1, v/v) as the eluent to afford the desired product.

Note: for the synthesis of **2g**, **2m-o**, **2r-u**, **3e** and **4c**, H_2O (200 µL) was added.

4.2 General procedure B. (for synthesis of esters **2i**, **2l**, **3a**, **3b** and **3d**).

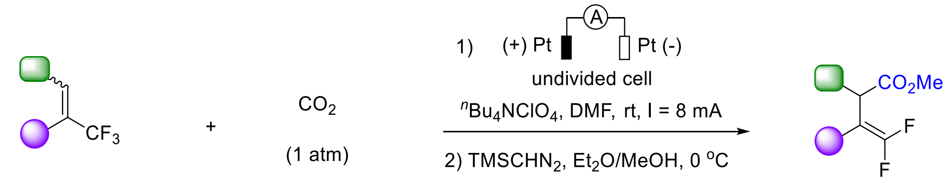

To a 10 mL hydrogenation tube or a cell of ElectraSyn 2.0 containing a stir bar were added ^{n}Bu$_4$NClO$_4$ (167.5 mg, 0.49 mmol) and DMF (7.0 mL), followed by the addition of **1** (0.2 mmol). Then the tube was installed with two Pt-plates as cathode and anode. After bubbling of CO_2 gas into the electrolytes for 10 mins, the reaction mixture was electrolyzed under a constant current of 8 mA until the complete consumption of the starting materials as monitored by TLC (4-7 hours). After that, the reaction mixture was transferred to a 50 mL Erlenmeyer flask and acidized with HCl (2 N, 10 mL). The aqueous layer extracted with EtOAc (2 x 20 mL) and the combined organics were washed with sat. NH_4Cl (2 x 20 mL), dried over Na_2SO_4, and concentrated in vacuo. The crude residue was dissolved in the mixture solvent of Et_2O (2.0 mL) and MeOH (0.5 mL) and cooled to 0 °C. The hexane solution of

$TMSCHN_2$ (0.2 mL, 2 mol/L, 0.4 mmol) was added and the mixture was stirred at 0 °C for 30 mins. The solvent was removed under reduced pressure and the crude residue was purified by column chromatography using PE/EtOAc (10:1, v/v) as the eluent to afford the desired product.

Note: 1) for the synthesis of **3a** and **3b**, graphite was used as anode; 2) H_2O (200 μL) was added for the synthesis of **3d**.

4.3 General procedure C. (for synthesis of amide **3c**)

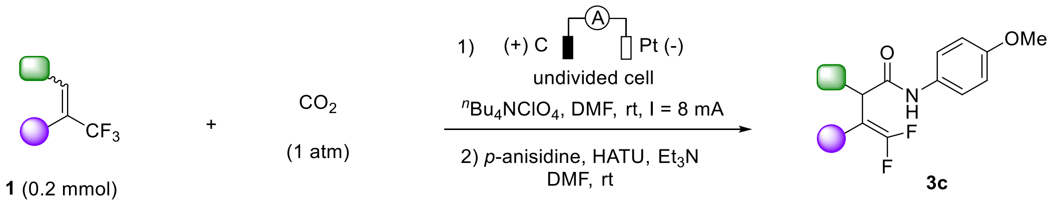

To a 10 mL hydrogenation tube or a cell of ElectraSyn 2.0 containing a stir bar were added $^{n}Bu_4NClO_4$ (167.5 mg, 0.49 mmol) and DMF (7.0 mL), followed by the addition of **1** (0.2 mmol). Then the tube was installed with Pt-plate as cathode and graphite as anode. After bubbling of CO_2 gas into the electrolytes for 10 mins, the reaction mixture was electrolyzed under a constant current of 8 mA until the complete consumption of the starting materials as monitored by TLC analysis (4-7 hours). After that, the reaction mixture was transferred to a 50 mL Erlenmeyer flask and acidized with HCl (2 N, 10 mL). The aqueous layer extracted with EtOAc (2 x 20 mL) and the combined organics were washed with sat. NH_4Cl (2 x 20 mL), dried over Na_2SO_4, and concentrated in vacuo. The crude residue was dissolved in DMF (2 mL), then *p*-anisidine (36.9 mg, 0.3 mmol), Et_3N (60.7 mg, 0.6 mmol), and *o*-(7-azabenzotriazol-1-yl)-*N,N,N',N'*-tramethyluronium hexafluorophosphate (HATU, 152.0 mg, 0.4 mmol) were added. The resulting mixture was stirred for 5 h at room temperature. The reaction was quenched with H_2O and extracted with EtOAc (3 x 10 mL). The combined organic layer was dried over Na_2SO_4 and concentrated under reduced pressure. The residue was purified by column chromatography using PE/EtOAc (5:1, v/v) as the eluent to afford the desired product.

4.4 General procedure for the gram-scale reaction.

To a 250 mL wild-mouth bottle containing a stir bar were added $^{n}Bu_4NClO_4$ (5.0 g, 14.7 mmol) and DMF (210 mL), followed by the addition of **1a** (1.03 g, 6.0 mmol). Then the tube was installed with two Pt-plates (30 mm x 30 mm x 0.1 mm) as cathode and anode. After bubbling of CO_2 gas into the electrolytes for 10 mins, the reaction mixture was electrolyzed under a constant current of 72 mA until the complete consumption of the starting materials as monitored by TLC (20 hours). After that, the

reaction mixture was transferred to a 500 mL round-bottom flask and then removing most of the solvent by distillation in vacuum. The residue was diluted with 50 mL EtOAc, and acidized with HCl (2 N, 40 mL). After separation of organic layer, the aqueous layer was extracted with EtOAc (2 x 40 mL) and the combined organics were washed with sat. NH_4Cl (2 x 40 mL), dried over Na_2SO_4, and concentrated in vacuo. The crude product was purified by column chromatography using PE/EtOAc (3:1, v/v) as the eluent to afford the desired **2a** in 0.93 g with 78% yield.

4.5 Characterization data of products

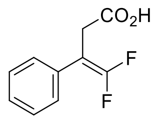

2a

Product **2a** was obtained in 32.8 mg (83% yield) as colorless oil; ^{1}H NMR (400 MHz, $CDCl_3$): δ 7.39-7.33 (m, 4H), 7.31-7.27 (m, 1H), 3.45 (t, J = 2.0 Hz, 2H); ^{13}C NMR (100 MHz, $CDCl_3$): δ 176.38, 155.00 (dd, J = 291.3 Hz, 287.7 Hz, 1C), 132.72 (t, J = 3.8 Hz, 1C), 128.60, 127.80 (t, J = 3.5 Hz, 1C), 127.69, 86.62 (dd, J = 21.5 Hz, 18.0 Hz, 1C), 33.60 (d, J = 2.8 Hz, 1C); ^{19}F NMR (376 MHz, $CDCl_3$): δ -87.13 (d, J = 33.5 Hz, 1F), -88.55 (d, J = 33.8 Hz, 1F); IR (ATR) ν 2972.3, 2887.4, 1728.2, 1712.8, 1498.7, 1413.8, 1247.9, 1176.6, 1128.4, 1006.8 cm^{-1}; HRMS (ESI): Exact mass calcd for $C_{10}H_8F_2NaO_2$ [M+Na]$^+$: 221.0385, found: 221.0383.

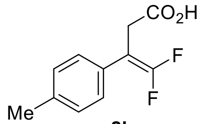

2b

Product **2b** was obtained in 35.6 mg (84% yield) as light yellow solid; Mp 52-54 °C; ^{1}H NMR (400 MHz, $CDCl_3$): δ 7.24-7.22 (m, 2H), 7.18-7.16 (m, 2H), 3.43 (t, J = 2.0 Hz, 2H), 2.35 (s, 3H); ^{13}C NMR (125 MHz, $CDCl_3$): δ 176.34, 154.85 (dd, J = 290.8 Hz, 287.4 Hz, 1C), 137.52, 129.63 (t, J = 3.8 Hz, 1C), 129.30, 127.60 (t, J = 3.5 Hz, 1C), 86.39 (dd, J = 21.1 Hz, 18.0 Hz, 1C), 33.56, 21.12; ^{19}F NMR (376 MHz, $CDCl_3$): δ -87.67 (d, J = 35.0 Hz, 1F), -88.91 (d, J = 35.0 Hz, 1F); IR (ATR) ν 2970.3, 1716.6, 1516.0, 1415.7, 1382.9, 1238.3, 1107.1, 1004.9, 879.5 cm^{-1}; HRMS (ESI): Exact mass calcd for $C_{11}H_{10}F_2NaO_2$ [M+Na]$^+$: 235.0541, found: 235.0542.

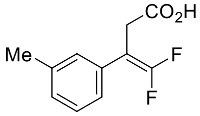

2c

Product **2c** was obtained in 30.9 mg (73% yield) as light yellow oil; ^{1}H NMR (400 MHz, $CDCl_3$): δ 7.26-7.22 (m, 1H), 7.14-7.08 (m, 3H), 3.42 (t, J = 2.4 Hz, 2H), 2.35 (s, 3H); ^{13}C NMR (100 MHz, $CDCl_3$): δ 176.56 (dd, J = 4.4 Hz, 2.8 Hz, 1C), 154.94 (dd, J = 291.0 Hz, 287.6 Hz, 1C), 138.23, 132.63 (t, J = 3.8 Hz, 1C), 128.48, 128.46, 128.42, 124.85 (t, J = 3.5 Hz, 1C), 86.63 (dd, J = 21.0 Hz, 18.0 Hz, 1C), 33.66 (d, J = 2.8 Hz, 1C), 21.40; ^{19}F NMR (376 MHz, $CDCl_3$): δ -87.34 (d, J = 33.8 Hz, 1F), -88.64 (d, J = 34.2 Hz, 1F); IR (ATR) ν 3020.5, 1716.6, 1695.4, 1604.7, 1490.9, 1413.8, 1311.5, 1240.2, 1182.3, 912.3 cm^{-1}; HRMS (ESI): Exact mass calcd for $C_{11}H_{10}F_2NaO_2$ [M+Na]$^+$: 235.0541, found: 235.0539.

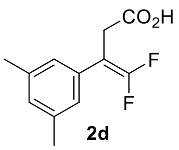

2d

Product **2d** was obtained in 34.3 mg (76% yield) as light yellow oil; ^{1}H NMR (400 MHz, $CDCl_3$): δ 6.94-6.93 (m, 3H), 3.42 (t, *J* = 2.0 Hz, 2H), 2.31 (s, 6H); ^{13}C NMR (100 MHz, $CDCl_3$): δ 176.70, 154.91 (dd, *J* = 290.9 Hz, 287.5 Hz, 1C), 138.10, 132.50 (t, *J* = 3.8 Hz, 1C), 129.44, 125.54 (t, *J* = 3.4 Hz, 1C), 86.58 (dd, *J* = 20.7 Hz, 18.2 Hz, 1C), 33.67 (d, *J* = 2.8 Hz, 1C), 21.28; ^{19}F NMR (376 MHz, $CDCl_3$): δ -87.50 (dd, *J* = 34.2 Hz, 1.5 Hz, 1F), -88.69 (d, *J* = 34.6 Hz, 1F), -116.80 (s, 1F); IR (ATR) ν 2987.7, 1716.6, 1602.8, 1411.8, 1259.5, 1176.5, 1062.7, 918.1, 850.6, 821.6 cm^{-1}; HRMS (ESI): Exact mass calcd for $C_{12}H_{12}F_2NaO_2$ [M+Na]$^+$: 249.0698, found: 249.0701.

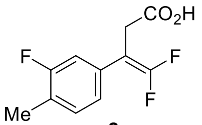

2e

Product **2e** was obtained in 32.6 mg (71% yield) as light yellow oil; ^{1}H NMR (400 MHz, $CDCl_3$): δ 7.16 (t, *J* = 8.0 Hz, 1H), 7.02 (d, *J* = 4.8 Hz, 1H), 7.00 (s, 1H), 3.41 (t, *J* = 2.4 Hz, 2H), 2.26 (d, *J* = 1.6 Hz, 3H); ^{13}C NMR (100 MHz, $CDCl_3$): δ 176.11, 161.15 (d, *J* = 243.4 Hz, 1C), 155.04 (dd, *J* = 291.9 Hz, 288.0 Hz, 1C), 132.04-131.88 (m, 1C), 131.53 (d, *J* = 5.6 Hz, 1C), 124.42 (d, *J* = 17.2 Hz,1C), 123.04 (dd, *J* = 7.1 Hz, 3.5 Hz, 1C), 114.47 (ddd, *J* = 23.7 Hz, 4.6 Hz, 3.1 Hz, 1C), 85.99 (ddd, *J* = 21.9 Hz, 17.6 Hz, 2.2 Hz, 1C), 33.38 (d, *J* = 2.8 Hz, 1C), 14.24 (d, *J* = 3.4 Hz, 1C); ^{19}F NMR (376 MHz, $CDCl_3$): δ -86.51 (d, *J* = 32.7 Hz, 1F), -87.50 (d, *J* = 32.7 Hz, 1F), -116.80 (s, 1F); IR (ATR) ν 2546.0, 1728.2, 1695.4, 1570.0, 1423.4, 1317.3, 1253.7, 1180.4, 1026.1, 848.6 cm^{-1}; HRMS (ESI): Exact mass calcd for $C_{11}H_9F_3NaO_2$ [M+Na]$^+$: 253.0447, found: 253.0457.

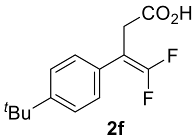

2f

Product **2f** was obtained in 35.6 mg (70% yield) as white solid; Mp 55-57 °C; ^{1}H NMR (400 MHz, $CDCl_3$): δ 7.40-7.37 (m, 2H), 7.30-7.27 (m, 2H), 3.44 (t, *J* = 2.0 Hz, 2H), 1.33 (s, 9H); ^{13}C NMR (100 MHz, $CDCl_3$): δ 176.43, 155.00 (dd, *J* = 291.2 Hz, 287.4 Hz, 1C), 150.66, 129.66 (t, *J* = 3.8 Hz, 1C), 127.35 (t, *J* = 3.8 Hz, 1C), 125.54, 86.35 (dd, *J* = 21.1 Hz, 17.8 Hz, 1C), 34.54, 33.51 (d, *J* = 2.9 Hz, 1C), 31.22; ^{19}F NMR (376 MHz, $CDCl_3$): δ -87.31 (d, *J* = 34.6 Hz, 1F), -88.65 (d, *J* = 34.2 Hz, 1F); IR (ATR) ν 2976.1, 1732.0, 1614.4, 1317.3, 1249.8, 1178.5, 1049.2, 1004.9, 881.4, 833.2 cm^{-1}; HRMS (ESI): Exact mass calcd for $C_{14}H_{16}F_2NaO_2$ [M+Na]$^+$: 277.1011, found: 277.1013.

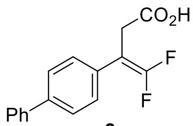

2g

Product **2g** was obtained in 41.6 mg (76% yield) as white solid with 200 μL H_2O as additive; Mp 120-122 °C; ^{1}H NMR (400 MHz, $CDCl_3$): δ 7.62-7.59 (m, 4H), 7.48-7.43 (m, 4H), 7.39-7.35 (m, 1H), 3.50 (t, *J* = 2.4 Hz, 2H); ^{13}C NMR (100 MHz, $CDCl_3$): δ 176.45, 155.07 (dd, *J* = 291.8 Hz, 288.2 Hz, 1C), 140.50, 140.35, 131.58 (t, *J* = 3.9 Hz, 1C), 128.80,

128.10 (t, J = 3.7 Hz, 1C), 127.49, 127.27, 127.00, 86.39 (dd, J = 21.5 Hz, 17.9 Hz, 1C), 33.48 (d, J = 2.8 Hz, 1C); ^{19}F NMR (376 MHz, $CDCl_3$): δ -86.53 (d, J = 32.7 Hz, 1F), -87.91 (d, J = 32.7 Hz, 1F); IR (ATR) ν 2972.3, 1734.0, 1693.5, 1489.0, 1417.6, 1404.1, 1249.8, 1078.2, 952.8, 839.0 cm^{-1}; HRMS (ESI): Exact mass calcd for $C_{16}H_{12}F_2NaO_2$ [M+Na]$^+$: 297.0698, found: 297.0693.

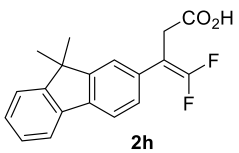

2h

Product **2h** was obtained in 45.2 mg (72% yield) as light yellow solid; Mp 125-127 °C; ^{1}H NMR (400 MHz, $CDCl_3$): δ 7.72-7.68 (m, 2H), 7.44-7.41 (m, 2H), 7.35-7.30 (m, 3H), 3.50 (t, J = 2.4 Hz, 2H), 1.48 (s, 6H); ^{13}C NMR (100 MHz, $CDCl_3$): δ 176.46, 155.02 (dd, J = 291.2 Hz, 287.9 Hz, 1C), 153.90, 153.80, 138.84, 138.50, 131.50 (t, J = 3.8 Hz, 1C), 127.49, 127.01, 126.61 (t, J = 3.6 Hz, 1C), 122.58, 122.04 (dd, J = 4.6 Hz, 3.1 Hz, 1C), 120.10, 120.04, 87.03 (dd, J = 21.1 Hz, 17.8 Hz, 1C), 46.88, 33.84 (d, J = 2.7 Hz, 1C), 27.04.; ^{19}F NMR (376 MHz, $CDCl_3$): δ -86.97 (d, J = 33.8 Hz, 1F), -88.21 (d, J = 34.2 Hz, 1F); IR (ATR) ν 3483.4, 3251.9, 1712.7, 1409.9, 1313.5, 1228.6, 1016.4, 900.7, 819.7, 783.1 cm^{-1}; HRMS (EI): Exact mass calcd for $C_{19}H_{16}O_2$ F_2 [M]$^+$: 314.1118, found: 314.1116.

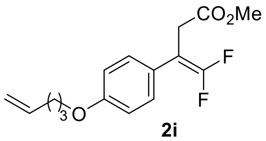

2i

Product **2i** was obtained in 26.6 mg (45% yield) as colorless oil; ^{1}H NMR (400 MHz, $CDCl_3$): δ 7.26-7.23 (m, 2H), 6.89-6.86 (m, 2H), 5.90-5.80 (m, 1H), 5.09-5.03 (m, 1H), 5.02-4.98 (m, 1H), 3.96 (t, J = 6.4 Hz, 2H), 3.67 (s, 3H), 3.37 (t, J = 2.4 Hz, 2H), 2.26-2.21 (m, 2H), 1.91-1.84 (m, 2H); ^{13}C NMR (125 MHz, $CDCl_3$): δ 170.77, 158.37, 154.64 (t, J = 286.4 Hz, 1C), 137.73, 128.95 (t, J = 3.5 Hz, 1C), 124.93, 115.22, 114.53, 86.57 (t, J = 19.1 Hz, 1C), 67.13, 52.17, 33.76, 30.08, 28.37; ^{19}F NMR (376 MHz, $CDCl_3$): δ -89.00 (d, J = 38.0 Hz, 1F), -90.00 (d, J = 38.0 Hz, 1F); IR (ATR) ν 2978.0, 1743.6, 1610.5, 1516.0, 1436.9, 1346.3, 1246.0, 1174.6, 914.2, 831.3 cm^{-1}; HRMS (ESI): Exact mass calcd for $C_{16}H_{18}F_2NaO_3$ [M+Na]$^+$: 319.1116, found: 319.1119.

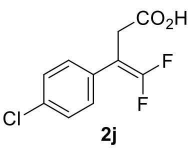

2j

Product **2j** was obtained in 33.4 mg (72% yield) as light yellow oil; ^{1}H NMR (400 MHz, $CDCl_3$): δ 7.34-7.32 (m, 2H), 7.28-7.25 (m, 2H), 3.42 (t, J = 2.4 Hz, 2H); ^{13}C NMR (100 MHz, $CDCl_3$): δ 176.35, 154.95 (dd, J = 291.7 Hz, 288.7 Hz, 1C), 133.64, 131.10 (t, J = 3.9 Hz, 1C), 129.12 (t, J = 3.7 Hz, 1C), 128.84, 85.89 (dd, J = 21.9 Hz, 17.9 Hz, 1C), 33.43 (d, J = 2.6 Hz, 1C); ^{19}F NMR (376 MHz, $CDCl_3$): δ -86.38 (d, J = 32.3 Hz, 1F), -87.69 (d, J = 32.3 Hz, 1F); IR (ATR) ν 2972.3, 1712.7, 1496.7, 1423.4, 1342.4, 1317.3, 1238.3, 1093.6, 1002.9, 912.3 cm^{-1}; HRMS (ESI): Exact mass calcd for $C_{10}H_7ClF_2NaO_2$ [M+Na]$^+$: 254.9995, found: 254.9993.

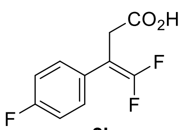

2k

Product **2k** was obtained in 31.9 mg (74% yield) as colorless oil; ^{1}H NMR (400 MHz, $CDCl_3$): δ 7.32-7.29 (m, 2H), 7.08-7.02 (m, 2H), 3.42 (t, *J* = 2.4 Hz, 2H); ^{13}C NMR (100 MHz, $CDCl_3$): δ 176.17, 162.10 (d, *J* = 246.2 Hz, 1C), 154.94 (t, *J* = 289.7 Hz, 1C), 129.73-129.58 (m, 1C), 128.62 (dd, *J* = 7.3 Hz, 3.6 Hz, 1C), 115.63 (d, *J* = 21.6 Hz, 1C), 85.90 (dd, *J* = 21.9 Hz, 18.6 Hz, 1C), 33.71 (d, *J* = 2.6 Hz, 1C); ^{19}F NMR (376 MHz, $CDCl_3$): δ -87.39 (dd, *J* = 34.2 Hz, 2.2 Hz, 1F), -88.70 (d, *J* = 34.2 Hz, 1F), -113.81 (d, *J* = 1.9 Hz, 1F); IR (ATR) ν 2999.3, 2563.4, 1747.5, 1722.4, 1604.7, 1510.2, 1417.6, 1313.5, 1247.9, 1006.8 cm^{-1}; HRMS (ESI): Exact mass calcd for $C_{10}H_7F_3NaO_2$ $[M+Na]^+$: 239.0290, found: 239.0282.

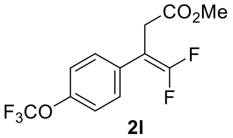

2l

Product **2l** was obtained in 43.8 mg (74% yield) as colorless oil; ^{1}H NMR (400 MHz, $CDCl_3$): δ 7.38-7.36 (m, 2H), 7.22-7.19 (m, 2H), 3.69 (s, 3H), 3.40 (t, *J* = 2.4 Hz, 2H); ^{13}C NMR (125 MHz, $CDCl_3$): δ 170.39 (dd, *J* = 4.5 Hz, 2.4 Hz, 1C), 154.95 (dd, *J* = 291.1 Hz, 288.1 Hz, 1C), 148.43, 131.66 (t, *J* = 4.1 Hz, 1C), 129.35 (t, *J* = 3.5 Hz, 1C), 120.96, 120.42 (q, *J* = 255.8 Hz, 1C), 86.28 (dd, *J* = 22.2 Hz, 17.6 Hz, 1C), 52.22, 33.48 (d, *J* = 2.8 Hz, 1C); ^{19}F NMR (376 MHz, $CDCl_3$): δ -57.88 (s, 3F), -86.86 (d, *J* = 33.1 Hz, 1F), -88.16 (d, *J* = 32.3 Hz, 1F); IR (ATR) ν 3523.9, 2976.1, 1743.6, 1610.5, 1512.1, 1438.9, 1352.1, 1159.2, 1006.8, 852.5 cm^{-1}; HRMS (ESI): Exact mass calcd for $C_{12}H_9F_5NaO_3$ $[M+Na]^+$: 319.0364, found: 319.0361.

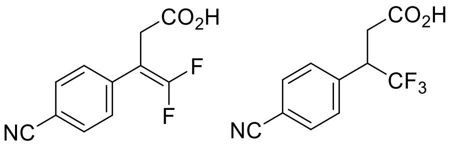

2m 2m'

Inseparable mixture **2m** and **2m'** was obtained in 29.8 mg (**2m**, 61% yield; **2m'**, 6% yield) as light yellow oil with 200 μL H_2O as additive; Selectivity (desired C-F cleavage product : byproduct) = 10:1. ^{1}H NMR (400 MHz, $CDCl_3$): δ 7.65 (d, *J* = 8.4 Hz, 2H), 7.45 (d, *J* = 8.0 Hz, 2H), 3.47 (t, *J* = 2.0 Hz, 2H); ^{13}C NMR (125 MHz, $CDCl_3$): δ 175.35, 155.34 (dd, *J* = 294.2 Hz, 290.5 Hz, 1C), 137.57 (t, *J* = 4.4 Hz, 1C), 132.37, 128.38 (t, *J* = 3.2 Hz, 1C), 118.36, 111.42, 86.11 (dd, *J* = 22.4 Hz, 16.9 Hz, 1C), 32.94; ^{19}F NMR (376 MHz, $CDCl_3$) (**2m**): δ -83.32 (d, *J* = 25.9 Hz, 1F), -85.07 (d, *J* = 25.9 Hz, 1F); ^{19}F NMR (376 MHz, $CDCl_3$) (**2m'**): -69.98 (s, 3F); IR (ATR) ν 3408.2, 2225.8, 1718.5, 1606.7, 1411.8, 1313.5, 1251.8, 1157.2, 1109.0, 1004.9 cm^{-1}; HRMS (ESI): Exact mass calcd for $C_{11}H_7F_2NO_2$ $[M+Na]^+$: 246.0337, found: 246.0334. The by-product might be formed via a β-hydrocarboxylation as recently reported by Malkov & Buckley[9a] and Nam[9b], respectively.

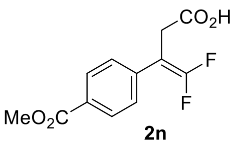

2n

Product **2n** was obtained in 27.6 mg (54% yield) as white solid with 200 μL H_2O as additive; Mp 77-79 ºC; ^{1}H NMR (400 MHz, $CDCl_3$): δ 8.03-8.01 (m, 2H), 7.42-7.40 (m, 2H), 3.91 (s, 3H), 3.47 (t, *J* = 2.0 Hz, 2H); ^{13}C NMR (125 MHz, $CDCl_3$): δ 175.77 (t, *J* = 3.2 Hz, 1C), 166.64, 155.22 (dd, *J* = 293.5 Hz, 289.5 Hz, 1C), 137.41 (t, *J* = 4.2 Hz, 1C), 129.83, 129.22, 127.65 (t, *J* = 3.8 Hz, 1C), 86.39 (dd, *J* = 21.8 Hz, 17.1 Hz, 1C), 52.21, 33.16 (d, *J* = 2.6 Hz, 1C); ^{19}F NMR (376 MHz, $CDCl_3$): δ -84.63 (d, *J* = 28.6 Hz, 1F), -86.15 (d, *J* = 28.6 Hz, 1F); IR (ATR) ν 2980.0, 2576.9, 1724.3, 1699.2, 1608.6, 1435.0, 1284.5, 1249.8, 1105.2, 945.1 cm^{-1}; HRMS (ESI): Exact mass calcd for $C_{12}H_{10}F_2NaO_4$ $[M+Na]^+$: 279.0439, found: 279.0440.

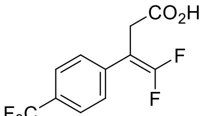

2o

Product **2o** was obtained in 40.9 mg (77% yield) as white solid with 200 μL H_2O as additive; Mp 88-90 ºC; ^{1}H NMR (400 MHz, $CDCl_3$): δ 7.62 (d, *J* = 8.0 Hz, 2H), 7.46 (d, *J* = 8.4 Hz, 2H), 3.47 (t, *J* = 2.4 Hz, 2H); ^{13}C NMR (125 MHz, $CDCl_3$): δ 176.07, 155.28 (dd, *J* = 292.9 Hz, 289.2 Hz, 1C), 136.43, 129.80 (q, *J* = 32.5 Hz, 1C), 128.12 (t, *J* = 3.6 Hz, 1C), 125.58 (q, *J* = 3.8 Hz, 1C), 123.88 (q, *J* = 270.4 Hz, 1C), 86.10 (dd, *J* = 22.2 Hz, 17.8 Hz, 1C), 33.32; ^{19}F NMR (376 MHz, $CDCl_3$): δ -62.79 (s, 3F), -84.90 (d, *J* = 29.0 Hz, 1F), -86.55 (d, *J* = 29.0 Hz, 1F); IR (ATR) ν 2976.1, 1751.3, 1734.0, 1616.3, 1498.6, 1328.9, 1246.0, 1161.1, 1008.7, 846.7 cm^{-1}; HRMS (ESI): Exact mass calcd for $C_{11}H_7F_5NaO_2$ $[M+Na]^+$: 289.0258, found: 289.0256.

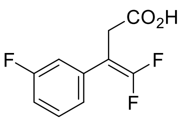

2p

Product **2p** was obtained in 34.1 mg (79% yield) as colorless oil; ^{1}H NMR (400 MHz, $CDCl_3$): δ 7.36-7.30 (m, 1H), 7.13-7.06 (m, 2H), 6.99 (td, *J* = 8.4 Hz, 2.4 Hz, 1H), 3.44 (t, *J* = 2.0 Hz, 2H); ^{13}C NMR (100 MHz, $CDCl_3$): δ 176.43 (t, *J* = 3.2 Hz, 1C), 162.74 (d, *J* = 244.5 Hz, 1C), 155.16 (dd, *J* = 292.5 Hz, 288.5 Hz, 1C), 134.89-134.72 (m, 1C), 130.11 (d, *J* = 8.4 Hz, 1C), 123.38 (dd, *J* = 6.9 Hz, 3.4 Hz, 1C), 114.93 (ddd, *J* = 22.7 Hz, 4.4 Hz, 3.1 Hz, 1C), 114.80, 86.27-85.85 (m, 1C), 33.37 (d, *J* = 2.6 Hz, 1C); ^{19}F NMR (376 MHz, $CDCl_3$): δ -85.74 (d, *J* = 30.8 Hz, 1F), -86.93 (d, *J* = 30.5 Hz, 1F), -112.48 (s, 1F); IR (ATR) ν 3560.5, 1724.3, 1614.4, 1585.4, 1490.9, 1411.8, 1253.7, 1120.6, 1022.2, 921.9 cm^{-1}; HRMS (ESI): Exact mass calcd for $C_{10}H_7F_3NaO_2$ $[M+Na]^+$: 239.0290, found: 239.0290.

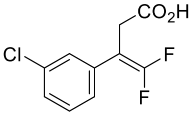

2q

Product **2q** was obtained in 29.2 mg (63% yield) as light yellow oil; ^{1}H NMR (400 MHz, $CDCl_3$): δ 7.37-7.28 (m, 3H), 7.23-7.21 (m, 1H), 3.43 (t, *J* = 2.0 Hz, 2H); ^{13}C NMR (100 MHz, $CDCl_3$): δ 175.76, 155.13 (dd, *J* = 292.3 Hz, 288.9 Hz, 1C), 134.53-134.46 (m), 129.84, 128.00-127.92 (m), 125.97 (t, *J* = 3.5 Hz, 1C), 85.92 (dd, *J* = 22.1 Hz, 17.9 Hz,

1C), 33.32 (d, J = 2.0 Hz, 1C); ^{19}F NMR (376 MHz, $CDCl_3$): δ -85.73 (d, J = 30.8 Hz, 1F), -87.03 (d, J = 30.8 Hz, 1F); IR (ATR) ν 3660.8, 2993.5, 2623.1, 1712.7, 1566.2, 1409.9, 1247.9, 1128.3, 1080.1, 881.4 cm^{-1}; HRMS (ESI): Exact mass calcd for $C_{10}H_7ClF_2NaO_2$ $[M+Na]^+$: 254.9995, found: 254.9990.

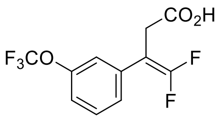
2r

Product **2r** was obtained in 37.9 mg (64% yield) as light yellow oil with 200 μL H_2O as additive; ^{1}H NMR (400 MHz, $CDCl_3$): δ 7.39 (t, J = 8.0 Hz, 1H), 7.28-7.26 (m, 1H), 7.21 (s, 1H), 7.17-7.14 (m, 1H), 3.44 (t, J = 2.0 Hz, 2H); ^{13}C NMR (100 MHz, $CDCl_3$): δ 175.85, 155.22 (dd, J = 292.6 Hz, 289.0 Hz, 1C), 149.38 (d, J = 1.9 Hz, 1C), 134.80 (t, J = 4.0 Hz, 1C), 129.97, 126.12 (t, J = 3.6 Hz, 1C), 120.58 (t, J = 3.5 Hz, 1C), 120.43 (q, J = 255.9 Hz, 1C), 120.07, 85.99 (dd, J = 22.5 Hz, 17.6 Hz, 1C), 33.36 (d, J = 2.5 Hz, 1C); ^{19}F NMR (376 MHz, $CDCl_3$): δ -57.89 (s, 3F), -85.42 (d, J = 30.1 Hz, 1F), -86.76 (d, J = 30.1 Hz, 1F); IR (ATR) ν 2985.8, 2875.8, 1732.0, 1716.6, 1583.5, 1423.4, 1321.2, 1253.7, 1155.3, 1041.5 cm^{-1}; HRMS (ESI): Exact mass calcd for $C_{11}H_7F_5NaO_3$ $[M+Na]^+$: 305.0208, found: 305.0205.

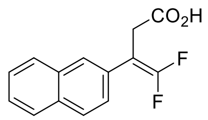
2s

Product **2s** was obtained in 30.7 mg (62% yield) as light yellow solid with 200 μL H_2O as additive; Mp 78-80 °C; ^{1}H NMR (400 MHz, $CDCl_3$): δ 7.84-7.81 (m, 3H), 7.79 (s, 1H), 7.51-7.46 (m, 3H), 3.55 (t, J = 2.0 Hz, 2H); ^{13}C NMR (100 MHz, $CDCl_3$): δ 176.76 (dd, J = 4.3 Hz, 2.7 Hz, 1C), 155.16 (dd, J = 291.6 Hz, 288.2 Hz, 1C), 133.14, 132.53, 130.04 (t, J = 3.8 Hz, 1C), 128.27, 128.00, 127.55, 126.93 (t, J = 3.7 Hz, 1C), 126.38, 126.35, 125.44 (dd, J = 4.5 Hz, 2.6 Hz, 1C), 86.74 (dd, J = 21.3 Hz, 17.9 Hz, 1C), 33.66 (d, J = 2.8 Hz, 1C); ^{19}F NMR (376 MHz, $CDCl_3$): δ -86.54 (d, J = 33.1 Hz, 1F), -88.15 (d, J = 32.7 Hz, 1F); IR (ATR) ν 3412.1, 2976.2, 1708.9, 1523.7, 1415.7, 1259.5, 1230.5, 1118.7, 935.4, 815.8 cm^{-1}; HRMS (ESI): Exact mass calcd for $C_{14}H_{10}F_2NaO_2$ $[M+Na]^+$: 271.0541, found: 271.0538.

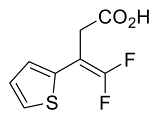
2t

Product **2t** was obtained in 16.3 mg (40% yield) as light yellow solid with 200 μL H_2O as additive; Mp 70-72 °C; ^{1}H NMR (400 MHz, $CDCl_3$): δ 7.31-7.29 (m, 1H), 7.05-7.01 (m, 2H), 3.48 (t, J = 2.0 Hz, 2H); ^{13}C NMR (125 MHz, $CDCl_3$): δ 176.26 (dd, J = 3.8 Hz, 3.1 Hz, 1C), 154.74 (dd, J = 294.5 Hz, 288.6 Hz, 1C), 134.55 (dd, J = 7.2 Hz, 2.9 Hz, 1C), 127.13, 125.44 (dd, J = 6.8 Hz, 3.0 Hz, 1C), 125.34 (dd, J = 5.9 Hz, 4.0 Hz, 1C), 83.26 (dd, J = 25.8 Hz, 18.5 Hz, 1C), 33.29 (d, J = 3.4 Hz, 1C); ^{19}F NMR (376 MHz, $CDCl_3$): δ -82.37 (d, J = 27.1 Hz, 1F), -88.53 (d, J = 26.7 Hz, 1F); IR (ATR) ν 2991.5, 1724.3, 1693.5, 1406.1, 1354.0, 1253.7, 1101.3, 1053.1, 869.9, 690.5 cm^{-1}; HRMS (ESI): Exact mass calcd for $C_8H_6F_2NaO_2S$ $[M+Na]^+$: 226.9949, found: 226.9948.

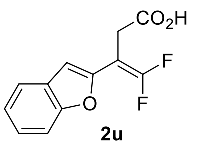

2u

Product **2u** was obtained in 20.0 mg (42% yield) as light yellow solid with 200 µL H_2O as additive; Mp 92-94 ºC; ^{1}H NMR (400 MHz, $CDCl_3$): δ 7.53-7.51 (m, 1H), 7.42 (d, *J* = 8.0 Hz, 1H), 7.25-7.18 (m, 2H), 6.74 (s, 1H), 3.54 (t, *J* = 2.0 Hz, 2H); ^{13}C NMR (125 MHz, $CDCl_3$): δ 175.98 (dd, *J* = 3.9 Hz, 3.0 Hz, 1C), 155.60 (dd, *J* = 298.2 Hz, 289.4 Hz, 1C), 154.27, 148.27 (dd, *J* = 7.2 Hz, 5.0 Hz, 1C), 128.57, 124.34, 123.11, 120.85, 111.08, 104.53 (dd, *J* = 9.4 Hz, 5.5 Hz, 1C), 81.19 (dd, *J* = 27.5 Hz, 16.9 Hz, 1C), 30.28 (d, *J* = 2.8 Hz, 1C); ^{19}F NMR (376 MHz, $CDCl_3$): δ -78.30 (d, *J* = 18.4 Hz, 1F), -84.86 (d, *J* = 18.4 Hz, 1F); IR (ATR) ν 2974.2, 1755.2, 1612.4, 1516.0, 1352.1, 1253.7, 1172.7, 1018.4, 935.4, 750.3 cm^{-1}; HRMS (ESI): Exact mass calcd for $C_{12}H_8F_2NaO_3$ $[M+Na]^+$: 261.0334, found: 261.0337.

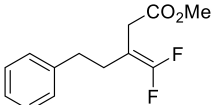

3a

Product **3a** was obtained in 25.9 mg (54% yield) as colorless oil with graphite rod as anode; ^{1}H NMR (400 MHz, $CDCl_3$): δ 7.23-7.19 (m, 2H), 7.14-7.09 (m, 3H), 3.62 (s, 3H), 2.91 (t, *J* = 2.0 Hz, 2H), 2.65-2.61 (m, 2H), 2.33-2.28 (m, 2H); ^{13}C NMR (100 MHz, $CDCl_3$): δ 170.85 (dd, *J* = 4.1 Hz, 3.0 Hz, 1C), 154.54 (dd, *J* = 284.7 Hz, 283.7 Hz, 1C), 140.78, 128.42, 128.27, 126.16, 83.59 (dd, *J* = 22.3 Hz, 16.5 Hz, 1C), 52.04, 33.52 (dd, *J* = 2.7 Hz, 2.5 Hz, 1C), 31.94 (dd, *J* = 3.2 Hz, 0.6 Hz, 1C), 28.52 (d, *J* = 1.8 Hz, 1C); ^{19}F NMR (376 MHz, $CDCl_3$): δ -92.50 (d, *J* = 47.0 Hz, 1F), -92.90 (d, *J* = 47.0 Hz, 1F); IR (ATR) ν 3736.1, 3066.8, 2958.8, 1753.2, 1604.7, 1436.9, 1350.1, 1257.5, 1172.7, 1064.7 cm^{-1}; HRMS (ESI): Exact mass calcd for $C_{13}H_{14}F_2NaO_2$ $[M+Na]^+$: 263.0854, found: 263.0858.

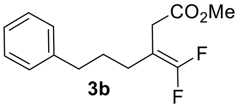

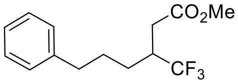

3b'

Inseparable mixture **3b** and **3b'** was obtained in 30.9 mg (**3b**, 57% yield; **3b'**, 4% yield) as light yellow oil with graphite rod as anode; Selectivity (desired C-F cleavage product : byproduct) = 13:1; ^{1}H NMR (400 MHz, $CDCl_3$): δ 7.29-7.25 (m, 2H), 7.19-7.15 (m, 3H), 3.68 (s, 3H), 2.99 (t, *J* = 2.0 Hz, 2H), 2.60 (t, *J* = 7.6 Hz, 2H), 2.13-2.08 (m, 2H); 1.75-1.68 (m, 2H); ^{13}C NMR (100 MHz, $CDCl_3$): δ 170.88 (dd, *J* = 4.3 Hz, 2.7 Hz, 1C), 154.42 (dd, *J* = 284.5 Hz, 283.1 Hz, 1C), 141.73, 128.32, 128.30, 125.85, 83.73 (dd, *J* = 22.6 Hz, 16.1 Hz, 1C), 51.99, 35.24, 31.78 (d, *J* = 3.5 Hz, 1C), 28.72 (t, *J* = 2.6 Hz, 1C), 26.36 (d, *J* = 1.7 Hz, 1C); ^{19}F NMR (376 MHz, $CDCl_3$) (**3b**): δ -92.55 (d, *J* = 48.1 Hz, 1F), -93.38 (d, *J* = 48.1 Hz, 1F); ^{19}F NMR (376 MHz, $CDCl_3$) (**3b'**): δ -71.31 (s, 3F); IR (ATR) ν 3028.2, 2953.0, 1755.2, 1604.7, 1496.7, 1352.1, 1278.8, 1170.7, 1093.6, 991.4 cm^{-1}; HRMS (ESI): Exact mass calcd for $C_{14}H_{17}O_2F_2$ $[M+H]^+$: 255.1191, found: 255.1182. The by-product might be formed via a β-hydrocarboxylation as recently reported by Malkov & Buckley[9a] and Nam[9b], respectively

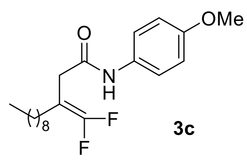
3c

Product **3c** was obtained in 32.5 mg (46% yield) as light yellow oil with graphite rod as anode;; ^{1}H NMR (400 MHz, $CDCl_3$): δ 7.40-7.35 (m, 3H), 6.86-6.82 (m, 2H), 3.78 (s, 3H), 3.03 (t, *J* = 2.4 Hz, 2H), 2.12-2.07 (m, 2H), 1.46-1.39 (m, 2H), 1.29-1.25 (m, 12H), 0.87 (t, *J* = 6.8 Hz, 3H); ^{13}C NMR (100 MHz, $CDCl_3$): δ 167.59-167.52 (m, 1C), 156.64, 154.49 (t, *J* = 284.4 Hz, 1C), 130.58, 122.00, 114.11, 85.16 (dd, *J* = 20.8 Hz, 15.8 Hz, 1C), 55.42, 35.07 (d, *J* = 2.6 Hz, 1C), 31.81, 29.47, 29.28, 29.22, 29.08, 27.20 (t, *J* = 2.5 Hz, 1C), 26.66, 22.61, 14.03; ^{19}F NMR (376 MHz, $CDCl_3$): δ -92.58 (d, *J* = 48.9 Hz, 1F), -92.82 (d, *J* = 48.9 Hz, 1F); IR (ATR) ν 3267.4, 2854.6, 1753.2, 1649.1, 1531.4, 1413.8, 1246.0, 1170.7, 1026.1, 827.4 cm^{-1}; HRMS (ESI): Exact mass calcd for $C_{20}H_{30}O_2NF_2$ $[M+H]^+$: 354.2239, found: 354.2228.

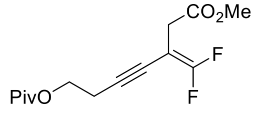
3d

Product **3d** was obtained in 27.6 mg (48% yield) as light yellow oil with 200 μL H_2O as additive; ^{1}H NMR (400 MHz, $CDCl_3$): δ 4.15 (t, *J* = 6.8 Hz, 2H), 3.72 (s, 3H), 3.06 (t, *J* = 2.0 Hz, 2H), 2.64 (tt, *J* = 6.4 Hz, 1.2 Hz, 2H), 1.20 (s, 9H); ^{13}C NMR (125 MHz, $CDCl_3$): δ 178.25, 169.62 (dd, *J* = 4.1 Hz, 3.0 Hz, 1C), 160.13 (dd, *J* = 294.1 Hz, 292.4 Hz, 1C), 90.52 (t, *J* = 5.8 Hz, 1C), 73.07 (dd, *J* = 34.0 Hz, 21.0 Hz, 1C), 72.56 (dd, *J* = 7.4 Hz, 4.4 Hz, 1C), 61.82, 52.27, 38.71, 33.18, 27.07, 19.91; ^{19}F NMR (376 MHz, $CDCl_3$): δ -79.67 (d, *J* = 12.8 Hz, 1F), -83.65 (d, *J* = 12.8 Hz, 1F); IR (ATR) ν 3522.0, 2978.0, 2879.7, 2243.2, 1728.2, 1438.9, 1352.1, 1286.5, 1151.5, 983.7 cm^{-1}; HRMS (ESI): Exact mass calcd for $C_{14}H_{18}F_2NaO_4$ $[M+Na]^+$: 311.1065, found: 311.1058.

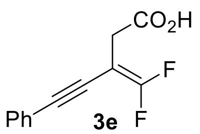
3e

Product **3e** was obtained in 28.8 mg (65% yield) as light yellow oil with 200 μL H_2O as additive; ^{1}H NMR (400 MHz, $CDCl_3$): δ 7.46-7.42 (m, 2H), 7.33-7.29 (m, 3H), 3.26 (t, *J* = 2.0 Hz, 2H); ^{13}C NMR (125 MHz, $CDCl_3$): δ 174.74, 159.86 (dd, *J* = 296.0 Hz, 294.5 Hz, 1C), 131.50, 128.64, 128.29, 122.39, 93.99 (t, *J* = 5.9 Hz, 1C), 79.71 (dd, *J* = 7.2 Hz, 4.6 Hz, 1C), 73.09 (dd, *J* = 34.1 Hz, 21.0 Hz, 1C), 33.08; ^{19}F NMR (376 MHz, $CDCl_3$): δ -77.46 (d, *J* = 8.6 Hz, 1F), -81.96 (d, *J* = 8.6 Hz, 1F); IR (ATR) ν 2968.4, 1720.5, 1490.9, 1411.8, 1286.5, 1159.2, 1087.8, 914.2 cm^{-1}; HRMS (ESI): Exact mass calcd for $C_{12}H_8F_2NaO_2$ $[M+Na]^+$: 245.0385, found: 245.0383.

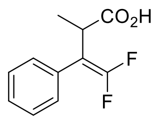
3f

Product **3f** was obtained in 23.7 mg (56% yield) as light yellow oil; ^{1}H NMR (400 MHz, $CDCl_3$): δ 7.39-7.36 (m, 1H), 7.35-7.30 (m, 2H), 7.29-7.27 (m, 2H), 3.67 (q, *J* = 7.2 Hz, 1H), 1.32 (d, *J* = 7.2 Hz, 3H); ^{13}C NMR (100 MHz, $CDCl_3$): δ 179.15, 154.34 (t, *J* = 288.7 Hz, 1C), 131.87 (d, *J* = 1.9 Hz, 1C), 129.22 (t, *J* = 2.6 Hz, 1C), 128.46, 127.96, 92.68 (t, *J* = 19.0 Hz,

1C), 39.09, 14.94; ^{19}F NMR (376 MHz, $CDCl_3$): δ -88.74 (s, 2F); IR (ATR) ν 2987.7, 2607.7, 1728.2, 1714.7, 1498.6, 1409.9, 1323.1, 1238.3, 1186.2, 1049.2 cm^{-1}; HRMS (ESI): Exact mass calcd for $C_{11}H_{10}F_2NaO_2$ $[M+Na]^+$: 235.0541, found: 235.0541.

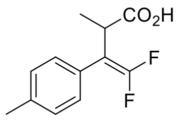

3g

Product **3g** was obtained in 23.0 mg (51% yield) as light yellow solid; Mp 90-92 °C; ^{1}H NMR (500 MHz, $CDCl_3$): δ 7.16 (s, 4H), 3.65 (q, *J* = 7.5 Hz, 1H), 2.35 (s, 3H), 1.30 (d, *J* = 7.5 Hz, 3H); ^{13}C NMR (125 MHz, $CDCl_3$): δ 178.90-178.83 (m, 1C), 154.24 (t, *J* = 288.4 Hz, 1C), 137.78, 129.18, 129.06 (t, *J* = 2.8 Hz, 1C), 128.71 (d, *J* = 2.8 Hz, 1C), 92.43 (t, *J* = 19.0 Hz, 1C), 38.96, 21.17, 14.91; ^{19}F NMR (376 MHz, $CDCl_3$): δ -89.10 (s, 1F), -89.11 (s, 1F); IR (ATR) ν 2980.0, 2617.4, 1728.2, 1697.3, 1516.0, 1450.4, 1317.3, 1236.3, 1053.1, 935.4 cm^{-1}; HRMS (ESI): Exact mass calcd for $C_{12}H_{12}F_2NaO_2$ $[M+Na]^+$: 249.0698, found: 249.0694.

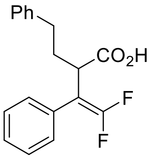

3h

Product **3h** was obtained in 24.8 mg (41% yield) as light yellow oil; ^{1}H NMR (400 MHz, $CDCl_3$): δ 7.38-7.32 (m, 3H), 7.29-7.23 (m, 4H), 7.20-7.16 (m, 1H), 7.09-7.07 (m, 2H), 3.53 (dd, *J* = 8.8 Hz, 6.4 Hz, 1H), 2.71-2.56 (m, 2H), 2.26-2.17 (m, 1H), 1.92-1.83 (m, 1H); ^{13}C NMR (100 MHz, $CDCl_3$): δ 178.35-178.29 (m, 1C), 154.83 (t, *J* = 289.5 Hz, 1C), 140.75, 131.64 (dd, *J* = 4.1 Hz, 2.1 Hz, 1C), 129.22 (t, *J* = 2.7 Hz, 1C), 128.52, 128.44, 128.39, 128.03, 126.14, 91.17 (dd, *J* = 19.2 Hz, 18.2 Hz, 1C), 43.69, 33.32, 30.60 (t, *J* = 2.1 Hz, 1C); ^{19}F NMR (376 MHz, $CDCl_3$): δ -87.45 (d, *J* = 35.0 Hz, 1F), -87.88 (d, *J* = 34.6 Hz, 1F); IR (ATR) ν 2904.8, 2600.0, 1734.0, 1705.0, 1496.7, 1409.9, 1244.0, 1176.5, 1010.7, 948.9 cm^{-1}; HRMS (ESI): Exact mass calcd for $C_{18}H_{16}F_2NaO_2$ $[M+Na]^+$: 325.1011, found: 325.1011.

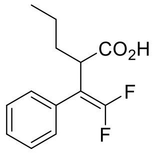

3i

Product **3i** was obtained in 19.2 mg (40% yield) as colorless oil; ^{1}H NMR (400 MHz, $CDCl_3$): δ 7.39-7.32 (m, 3H), 7.31-7.27 (m, 2H), 3.54 (dd, *J* = 8.4 Hz, 6.4 Hz, 1H), 1.90-1.81 (m, 1H), 1.62-1.52 (m, 1H), 1.44-1.34 (m, 2H), 0.90 (t, *J* = 7.2 Hz, 3H); ^{13}C NMR (125 MHz, $CDCl_3$): δ 179.04 (t, *J* = 3.0 Hz, 1C), 154.75 (t, *J* = 289.0 Hz, 1C), 131.86 (dd, *J* = 4.2 Hz, 2.2 Hz, 1C), 129.24 (t, *J* = 2.8 Hz, 1C), 128.44, 127.93, 91.26 (dd, *J* = 19.4 Hz, 18.4 Hz, 1C), 44.32 (d, *J* = 1.9 Hz, 1C), 30.90 (t, *J* = 2.1 Hz, 1C), 20.49, 13.67; ^{19}F NMR (376 MHz, $CDCl_3$): δ -87.81 (d, *J* = 36.1 Hz, 1F), -88.53 (d, *J* = 36.1 Hz, 1F); IR (ATR) ν 2962.6, 1732.0, 1716.6, 1583.5, 1496.7, 1411.8, 1317.3, 1246.0, 1047.3, 970.1 cm^{-1}; HRMS (ESI): Exact mass calcd for $C_{13}H_{14}F_2NaO_2$ $[M+Na]^+$: 263.0854, found: 263.0857.

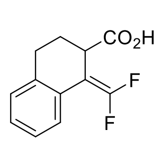

3j

Product **3j** was obtained in 23.3 mg (52% yield) as white solid; Mp 100-102 ºC; ^{1}H NMR (400 MHz, $CDCl_3$): δ 7.62-7.59 (m, 1H), 7.23-7.11 (m, 3H), 3.78 (td, *J* = 5.2 Hz, 2.4 Hz, 1H), 2.93-2.85 (m, 1H), 2.74 (dt, *J* = 16.4 Hz, 4.8 Hz, 1H), 2.31-2.24 (m, 1H), 2.12-2.04 (m, 1H); ^{13}C NMR (100 MHz, $CDCl_3$): δ 178.13 (dd, *J* = 3.6 Hz, 2.7 Hz, 1C), 154.27 (dd, *J* = 297.1 Hz, 285.4 Hz, 1C), 136.49 (dd, *J* = 6.2 Hz, 0.9 Hz, 1C), 128.78, 127.46-127.36 (m, 1C), 127.22 (d, *J* = 0.5 Hz, 1C), 127.02 (t, *J* = 2.0 Hz, 1C), 126.54, 87.32 (dd, *J* = 22.4 Hz, 11.5 Hz, 1C), 39.34 (t, *J* = 2.3 Hz, 1C), 27.07, 25.01; ^{19}F NMR (376 MHz, $CDCl_3$): δ -84.32 (d, *J* = 33.8 Hz, 1F), -86.08 (d, *J* = 33.8 Hz, 1F); IR (ATR) ν 3026.3, 2802.5, 2601.9, 1707.0, 1489.0, 1417.6, 1317.3, 1165.0, 991.4 cm^{-1}; HRMS (ESI): Exact mass calcd for $C_{12}H_{10}F_2NaO_2$ $[M+Na]^+$: 247.0541, found: 247.0541.

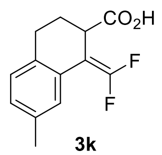

3k

Product **3k** was obtained in 26.2 mg (55% yield) as light yellow solid; Mp 128-130 ºC; ^{1}H NMR (400 MHz, $CDCl_3$): δ 7.42 (t, *J* = 2.0 Hz, 1H), 7.04-6.98 (m, 2H), 3.79-3.76 (m, 1H), 2.88-2.80 (m, 1H), 2.74-2.68 (m, 1H), 2.34 (s, 3H), 2.29-2.24 (m, 1H), 2.11-2.02 (m, 1H); ^{13}C NMR (125 MHz, $CDCl_3$): δ 179.23 (dd, *J* = 3.5 Hz, 2.4 Hz, 1C), 154.14 (dd, *J* = 297.0 Hz, 285.1 Hz, 1C), 135.96, 133.47 (d, *J* = 6.1 Hz, 1C), 128.64, 127.96 (t, *J* = 2.0 Hz, 1C), 127.71 (dd, *J* = 13.9 Hz, 0.8 Hz, 1C), 127.14 (dd, *J* = 6.2 Hz, 3.2 Hz, 1C), 87.36 (dd, *J* = 22.1 Hz, 11.5 Hz, 1C), 39.36 (t, *J* = 2.5 Hz, 1C), 26.63, 25.16, 21.25; ^{19}F NMR (376 MHz, $CDCl_3$): δ -84.58 (d, *J* = 34.2 Hz, 1F), -86.13 (d, *J* = 34.2 Hz, 1F); IR (ATR) ν 3053.3, 1747.5, 1697.3, 1496.7, 1417.6, 1317.3, 1246.0, 1166.9, 1076.2, 937.4 cm^{-1}; HRMS (ESI): Exact mass calcd for $C_{13}H_{12}F_2NaO_2$ $[M+Na]^+$: 261.0698, found: 261.0697.

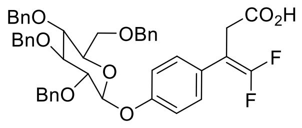

4a

Product **4a** was obtained in 73.6 mg (50% yield) as light yellow solid; Mp 108-110 ºC; ^{1}H NMR (400 MHz, $CDCl_3$): δ 7.34-7.22 (m, 20H), 7.19-7.16 (m, 2H), 7.06-7.04 (m, 2H), 5.02-4.98 (m, 2H), 4.96-4.93 (m, 1H), 4.85-4.80 (m, 3H), 4.59-4.54 (m, 2H), 4.52-4.49 (m, 1H), 3.80-3.73 (m, 3H), 3.69-3.65 (m, 2H), 3.62-3.58 (m, 1H), 3.39 (t, *J* = 2.0 Hz, 2H); ^{13}C NMR (100 MHz, $CDCl_3$): δ 175.92-175.85 (m, 1C), 156.63, 154.81 (dd, *J* = 290.7 Hz, 287.4 Hz, 1C), 138.40, 138.08, 137.97, 137.90, 129.02 (t, *J* = 3.7 Hz, 1C), 128.38, 128.36, 128.29, 128.19, 127.94, 127.84, 127.80, 127.76, 127.70, 127.64, 127.58, 126.88 (t, *J* = 3.9 Hz, 1C), 116.90, 101.42, 86.08 (dd, *J* = 21.5 Hz, 18.0 Hz, 1C), 84.59, 81.87, 77.60, 75.74, 75.09, 75.03, 75.02, 73.44, 68.72, 33.59; ^{19}F NMR (376 MHz, $CDCl_3$): δ -87.66 (d, *J* = 35.3 Hz, 1F), -89.05 (d, *J* = 35.3 Hz, 1F); IR (ATR) ν 3030.1, 2362.8, 1720.5, 1516.0, 1423.4, 1240.2, 1066.6, 831.3 cm^{-1}; HRMS (ESI): Exact mass calcd for $C_{44}H_{42}F_2NaO_8$ $[M+Na]^+$: 759.2740, found: 759.2748.

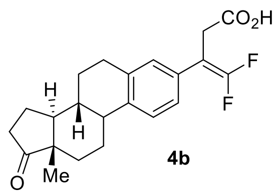

4b

Product **4b** was obtained in 39.6 mg (53% yield) as light yellow oil; ^{1}H NMR (400 MHz, $CDCl_3$): δ 7.28 (d, *J* = 8.0 Hz, 1H), 7.13 (d, *J* = 8.0 Hz, 1H), 7.08 (s, 1H), 3.43 (t, *J* = 2.0 Hz, 2H), 2.92 (d, *J* = 4.0 Hz, 1H), 2.90 (d, *J* = 4.4 Hz, 1H), 2.55-2.48 (m, 1H), 2.44-2.39 (m, 1H), 2.32-2.26 (m, 1H), 2.20-2.11 (m, 1H), 2.10-1.95 (m, 3H), 1.66-1.43 (m, 6H), 0.91 (s, 3H); ^{13}C NMR (100 MHz, $CDCl_3$): δ 175.88 (t, *J* = 2.8 Hz, 1C), 154.90 (dd, *J* = 290.9 Hz, 287.3 Hz, 1C), 139.34, 136.74, 130.08 (t, *J* = 3.7 Hz, 1C), 128.22 (t, *J* = 3.5 Hz, 1C), 125.58, 125.06 (t, *J* = 3.5 Hz, 1C), 86.31 (dd, *J* = 21.0 Hz, 17.9 Hz, 1C), 50.46, 47.98, 44.29, 37.98, 35.82, 33.42 (d, *J* = 2.8 Hz, 1C), 31.51, 29.36, 26.39, 25.55, 21.55, 13.79; ^{19}F NMR (376 MHz, $CDCl_3$): δ -87.31 (d, *J* = 34.6 Hz, 1F), -88.63 (d, *J* = 34.6 Hz, 1F); IR (ATR) ν 3419.7, 1735.9, 1516.0, 1406.1, 1288.4, 1247.9, 1087.8, 1008.7, 912.3, 881.4 cm^{-1}; HRMS (ESI): Exact mass calcd for $C_{22}H_{24}F_2NaO_3$ $[M+Na]^+$: 397.1586, found: 397.1585.

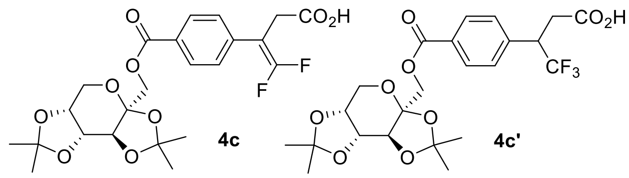

4c 4c'

Inseparable mixture **4c** and **4c'** was obtained in 44.5 mg (**4c**, 41% yield; **4c'**, 5% yield) as light yellow oil with 200 μL H_2O as additive; Selectivity (desired C-F cleavage product : byproduct) = 8:1; ^{1}H NMR (400 MHz, $CDCl_3$): δ 8.06-8.04 (m, 2H), 7.43-7.40 (m, 2H), 4.68-4.62 (m, 2H), 4.44 (d, *J* = 2.4 Hz, 1H), 4.34-4.31 (m, 1H), 4.27-4.25 (m, 1H), 3.97-3.93 (m, 1H), 3.82-3.78 (m, 1H), 3.46 (t, *J* = 2.0 Hz, 2H), 1.55 (s, 3H), 1.45 (s, 3H), 1.37 (s, 3H), 1.34 (s, 3H); ^{13}C NMR (100 MHz, $CDCl_3$): δ 174.49, 165.46, 155.20 (dd, *J* = 282.6 Hz, 278.2 Hz, 1C), 137.91-137.82 (m, 1C), 129.93, 128.83, 127.69-127.62 (m, 1C), 109.17, 108.84, 101.58, 86.67 (dd, *J* = 21.9 Hz, 16.6 Hz Hz, 1C), 70.73, 70.50, 70.03, 65.40, 61.30, 49.49, 33.43, 33.23, 26.44, 25.78, 25.48, 23.96; ^{19}F NMR (376 MHz, $CDCl_3$) (**4c**): δ -84.41 (d, *J* = 28.2 Hz, 1F), -86.01 (d, *J* = 28.2 Hz, 1F); ^{19}F NMR (376 MHz, $CDCl_3$) (**4c'**): δ -70.05 (d, *J* = 3.4 Hz, 3F); IR (ATR) ν 3408.2, 2989.6, 1720.5, 1610.5, 1456.2, 1375.2, 1276.8, 1163.0, 1066.6, 887.2 cm^{-1}; HRMS (ESI): Exact mass calcd for $C_{23}H_{26}F_2NaO_9$ $[M+Na]^+$: 507.1437, found: 507.1436. The by-product might be formed via a β-hydrocarboxylation as recently reported by Malkov & Buckley[9a] and Nam[9b], respectively.

5. Product elaboration

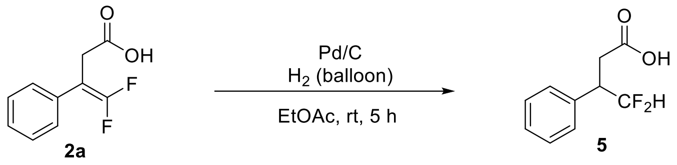

A 10 mL flask containing **2a** (0.2 mmol, 39.6 mg), Pd/C (5.0 mg, 10 wt%) and EtOAc (3.0 mL) was charged with H_2 balloon. The reaction mixture was stirred for 5 h at room temperature and monitored by TLC until full conversion of **2a**. Then the reaction mixture was diluted with EtOAc, filtered, and dried with Na_2SO_4, and concentrated in vacuo. The residue was purified by column chromatography using PE/EtOAc (1:1, v/v) as the eluent to afford **5**[10] in 36 mg with 90% yield as colorless oil. ^{1}H NMR (400 MHz, $CDCl_3$): δ 7.37-7.33 (m, 2H), 7.32-7.29 (m, 1H), 7.28-7.27 (m, 1H), 7.26-7.25 (m, 1H), 5.91 (td, J = 56.4 Hz, 3.2 Hz, 1H), 3.65-3.53 (m, 1H), 3.00 (dd, J = 16.8 Hz, 5.6 Hz, 1H), 2.81 (dd, J = 16.8 Hz, 8.8 Hz, 1H); ^{13}C NMR (100 MHz, $CDCl_3$): δ 177.08, 135.35 (dd, J = 5.3 Hz, 2.3 Hz, 1C), 128.82, 128.64, 128.12, 116.74 (t, J = 243.6 Hz, 1C), 45.49 (t, J = 20.3 Hz, 1C), 33.13 (t, J = 4.1 Hz, 1C); ^{19}F NMR (376 MHz, $CDCl_3$): δ -119.20 (d, J = 277.9 Hz, 1F), -123.96 (d, J = 277.5 Hz, 1F).

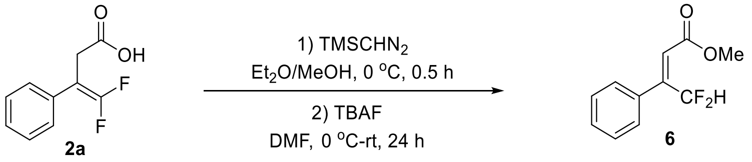

To a 10 mL flask were added **2a** (0.2 mmol, 39.6 mg), Et_2O (2.0 mL) and MeOH (0.5 mL), and the hexane solution of $TMSCHN_2$ (0.2 mL, 2 mol/L, 0.4 mmol) was added at 0 °C. The mixture was stirred at that temperature for another 30 mins. Then the solvent was removed under reduced pressure, and the crude residue was dissolved in DMF (1 mL) and cooled to 0 °C, followed by the addition of 0.24 mL of TBAF (0.24 mmol, 1.0 M in THF). The resulting mixture was slowly warmed to room temperature and stirred for 24 h. After monitored by TLC till full conversion to the product, the reaction was quenched by saturated aqueous solution of NaCl (5 mL) and extracted with Et_2O (5 mL x 3). The combined organic layer was dried over anhydrous Na_2SO_4 and concentrated under reduced pressure. The residue was purified by column chromatography using PE/EtOAc (10:1, v/v) as eluent to afford **6** in 33.0 mg with 78% yield as light yellow oil. ^{1}H NMR (400 MHz, $CDCl_3$): δ 7.42-7.40 (m, 3H), 7.28-7.26 (m, 2H), 6.37 (t, J = 2.0 Hz, 1H), 6.25 (t, J = 55.6 Hz, 1H), 3.61 (s, 3H); ^{13}C NMR (125 MHz, $CDCl_3$): δ 165.07, 147.34 (t, J = 20.0 Hz, 1C), 132.36, 129.01, 128.24, 128.20, 122.30 (t, J = 8.9 Hz, 1C), 114.12 (t, J = 241.4 Hz, 1C), 51.76; ^{19}F NMR (376 MHz, $CDCl_3$): δ -116.38 (s, 2F); HRMS (EI): Exact mass calcd for $C_{11}H_{10}O_2F_2$ $[M]^+$: 212.0649, found: 212.0652.

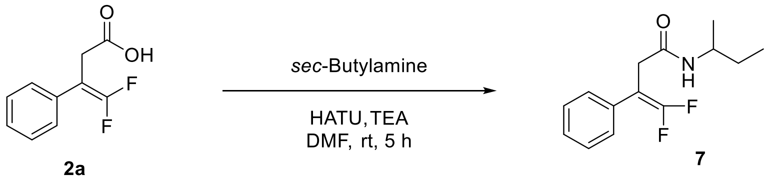

The carboxylic acid **2a** (0.2 mmol, 39.6 mg) was dissolved in DMF (2 mL), then *sec*-butylamine (21.9 mg, 0.3 mmol), *o*-(7-Azabenzotriazol-1-yl)-*N,N,N',N'*-tramethyluronium hexafluorophosphate (HATU, 152.0 mg, 0.4 mmol) and Et_3N (60.7 mg, 0.6 mmol) were added. The resulting mixture was stirred for 5 h at room temperature till full conversion of **2a**. Then the reaction was quenched with H_2O and extracted with EtOAc (3 x 10 mL). The combined organic layer was dried over Na_2SO_4 and concentrated under reduced pressure. The residue was purified by column chromatography using PE/EtOAc (2:1, v/v) as eluent to afford **7** in 46.2 mg with 92% yield as white solid. Mp 85-87 ºC; ^{1}H NMR (400 MHz, $CDCl_3$): δ 7.37-7.34 (m, 4H), 7.32-7.27 (m, 1H), 5.31 (br, 1H), 3.90-3.80 (m, 1H), 3.31 (t, *J* = 2.4 Hz, 2H), 1.40-1.28 (m, 2H), 1.00 (d, *J* = 6.8 Hz, 3H), 0.74 (t, *J* = 7.6 Hz, 3H); ^{13}C NMR (100 MHz, $CDCl_3$): δ 168.12, 154.75 (dd, *J* = 292.6 Hz, 287.7 Hz, 1C), 132.59 (t, *J* = 3.8 Hz, 1C), 128.72, 127.78 (d, *J* = 4.0 Hz, 1C), 127.74 (d, *J* = 3.4 Hz, 1C), 87.89 (dd, *J* = 21.0 Hz, 15.6 Hz, 1C), 46.82, 35.98 (d, *J* = 1.8 Hz, 1C), 29.45, 20.24, 10.01; ^{19}F NMR (376 MHz, $CDCl_3$): δ -86.94 (d, *J* = 33.8 Hz, 1F), -87.73 (d, *J* = 33.8 Hz, 1F); IR (ATR) ν 3900.0, 2968.4, 1747.5, 1633.7, 1448.5, 1309.6, 1232.5, 1159.2, 1080.1, 881.4 cm^{-1}; HRMS (ESI): Exact mass calcd for $C_{14}H_{17}F_2NNaO$ $[M+Na]^+$: 276.1170, found: 276.1179.

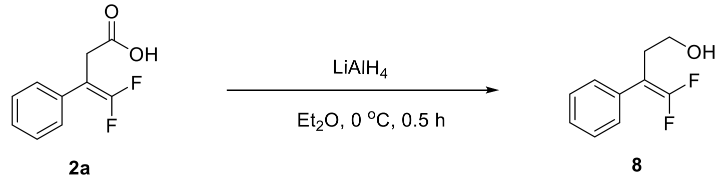

The carboxylic acid **2a** (0.2 mmol, 39.6 mg) was dissolved in Et_2O (2 mL) and cooled to 0 °C. Then $LiAlH_4$ (7.6 mg, 0.2 mmol, 1.0 equiv) was added. The resulting mixture was stirred for 0.5 h at 0 °C. After full conversion of **2a** monitored by TLC, the reaction was quenched by H_2O and extracted with Et_2O (10 mL). The combined organic layer was dried over Na_2SO_4 and concentrated under reduced pressure. The residue was purified by column chromatography using PE/EtOAc (10:1, v/v) as eluent to afford **8**[11] in 30.2 mg with 82% yield as colorless oil. ^{1}H NMR (500 MHz, $CDCl_3$): δ 7.38-7.33 (m, 4H), 7.31-7.28 (m, 1H), 3.66 (t, *J* = 7.0 Hz, 2H), 2.68-2.66 (m, 2H); ^{13}C NMR (125 MHz, $CDCl_3$): δ 154.26 (dd, *J* = 289.5 Hz, 286.0 Hz, 1C), 133.14 (t, *J* = 3.4 Hz, 1C), 128.54, 128.22 (t, *J* = 3.1 Hz, 1C), 127.47, 89.28 (dd, *J* = 21.4 Hz, 14.4 Hz, 1C), 60.46 (dd, *J* = 3.4 Hz, 2.6 Hz, 1C), 31.20 (d, *J* = 1.8 Hz,

1C); ^{19}F NMR (471 MHz, $CDCl_3$): δ -89.68 (d, J = 41.0 Hz, 1F), -90.57 (d, J = 41.0 Hz, 1F).

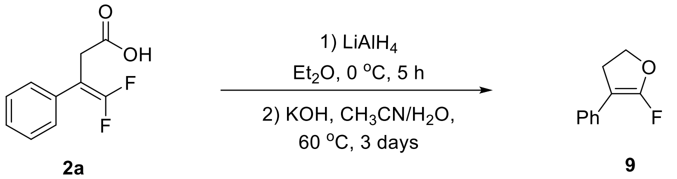

The carboxylic acid **2a** (0.2 mmol, 39.6 mg) was dissolved in Et_2O (2 mL) and cooled to 0 °C. Then $LiAlH_4$ (7.6 mg, 0.2 mmol, 1.0 equiv) was added. The resulting mixture was stirred for 0.5 h at 0 °C. After the full conversion of **2a** monitored by TLC analysis, the reaction was quenched with H_2O and extracted with Et_2O (10 mL). The combined organic layer was dried over Na_2SO_4 and concentrated under reduced pressure. Then the crude residue was dissolved in CH_3CN (2 mL) and KOH (13.4 mg, 0.24 mmol) with H_2O (60 μL) were added. The resulting mixture was stirred for 3 days at 60 °C. After the full transformation to final product by TLC analysis, the reaction was quenched with H_2O and extracted with Et_2O (10 mL). The combined organic layer was dried over Na_2SO_4 and concentrated under reduced pressure at 0 °C. The residue was purified by column chromatography using PE as eluent to afford **9** in 13.0 mg with 40% yield as colorless oil. ^{1}H NMR (400 MHz, CD_2Cl_2): δ 7.38-7.31 (m, 1H), 7.31-7.29 (m, 1H), 7.28-7.26 (m, 2H), 7.14-7.10 (m, 1H), 4.54 (t, J = 8.8 Hz, 2H), 3.07 (td, J = 9.2 Hz, 3.6 Hz, 2H); ^{13}C NMR (125 MHz, CD_2Cl_2): δ 157.36 (d, J = 275.8 Hz, 1C),132.59 (d, J = 6.2 Hz, 1C), 128.39, 124.90 (d, J = 1.9 Hz, 1C), 124.55 (d, J = 5.8 Hz, 1C), 81.37 (d, J = 10.9 Hz, 1C), 67.67 (d, J = 4.2 Hz, 1C), 29.26 (d, J = 3.2 Hz, 1C); ^{19}F NMR (376 MHz, CD_2Cl_2): δ -108.47 (s, 1F). HRMS (EI): Exact mass calcd for $C_{10}H_9OF$ $[M]^+$: 164.0637, found: 164.0640.

Note: The final product **9** should be very volatile, as it can be removed by water pump under room temperature, so the concentration is suggested to be performed at low temperature.

6. Mechanistic studies

6.1 Cyclic voltammetry (CV) analysis

Cyclic voltammograms were recorded with a Bio-logic VSP Potentiostat/Galvanostat equipped with electrochemical analysis software at room temperature in DMF. *n*-Bu_4NClO_4 (0.07 M) was used as the supporting electrolyte, and a Pt disk electrode was used as the working electrode. The auxiliary electrode was a Pt pillar. All potentials are referenced against the Ag/$AgNO_3$ (0.1 M in CH_3CN) redox couple. The scan rate is 100 mV s^{-1}.

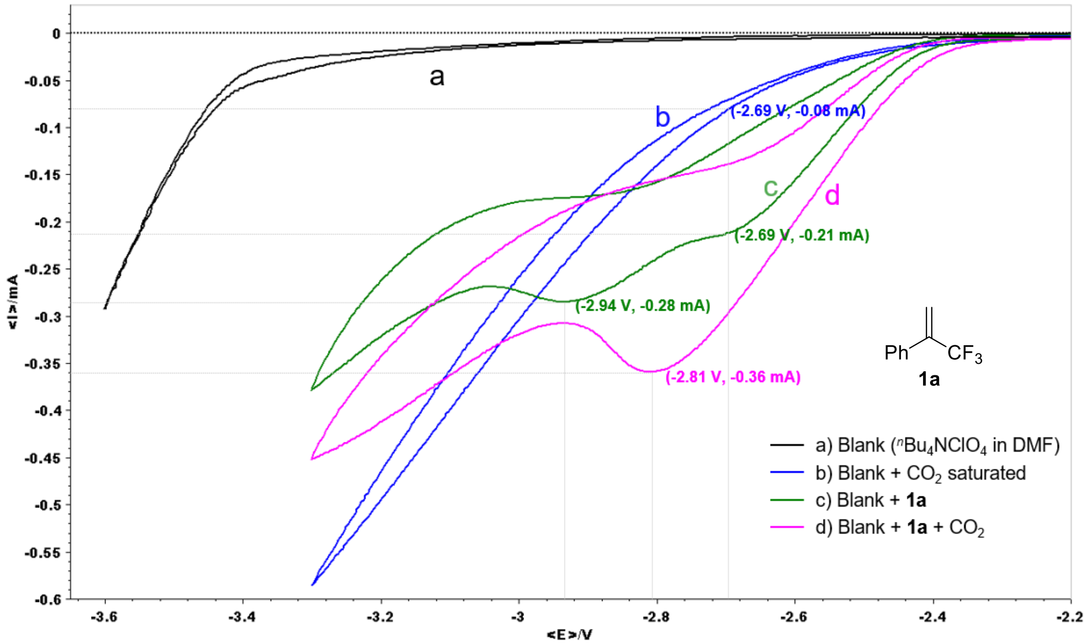

Figure S1. CV analyses of **1a**. (a) DMF containing 0.07 M *n*-Bu_4NClO_4; (b) DMF containing 0.07 M *n*-Bu_4NClO_4, with CO_2 saturated; (c) DMF containing 0.07 M *n*-Bu_4NClO_4 after addition of 0.02 M **1a**; (d) DMF containing 0.07 M *n*-Bu_4NClO_4 after addition of 0.02 M **1a** and CO_2 saturated.

For the CV of α-trifluoromethyl styrene **1a** (Figure S1), a one-electron reduction peak in the potential at -2.69 V and a second one at -2.94 V was observed (green line), whereas at the potential of -2.69 V, the reduction current of CO_2 was less than 0.1 mA (blue line), indicating that **1a** should be easier to reduce than CO_2. After the solution of **1a** was saturated with CO_2 (pink line), only one reduction peak was observed at -2.81 V with an associated peak current increase from 0.21 to 0.36 mA (ca. 1.7 times). These results suggested that an ECEC process might be involved, in which a radical anion might be generated after the first one-electron electroreduction, that could react immediately with CO_2, then the second electron transfer is facilitated at a less negative potential thus leading to a significant increase in

current observed. Accordingly, since a different species is being reduced in the presence of CO_2, the second peak at -2.94 V is not observed.

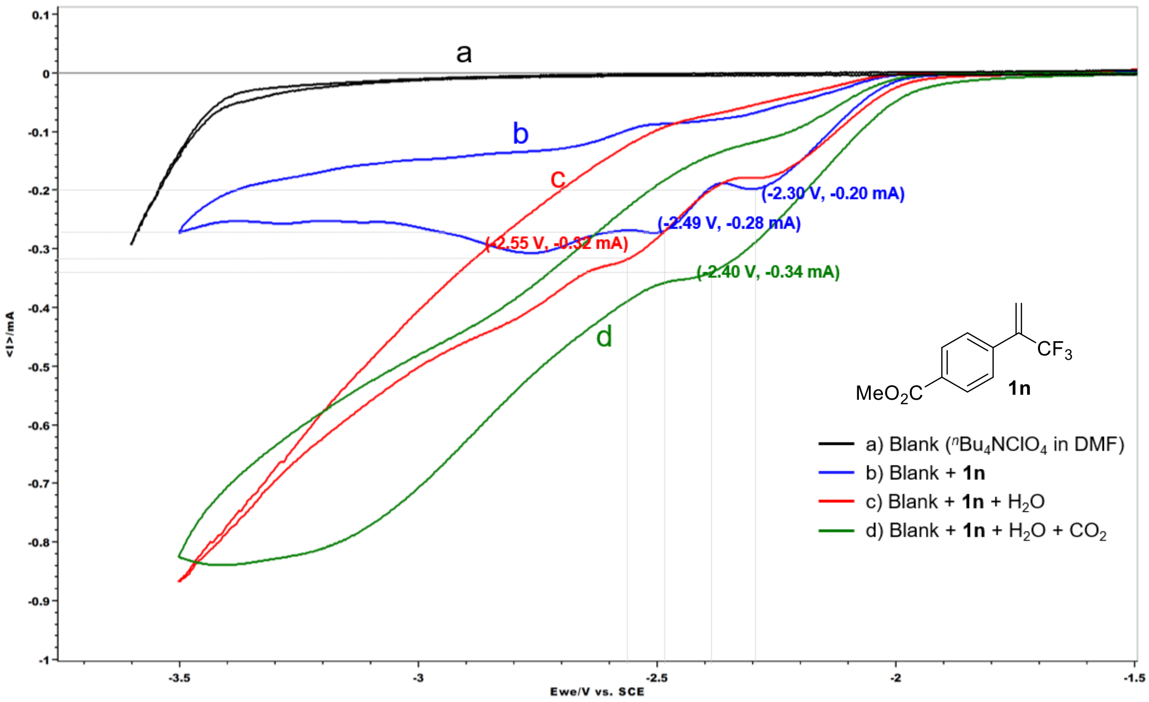

Figure S2. CV analyses of **1n**. (a) DMF containing 0.07 M *n*-Bu_4NClO_4; (b) DMF containing 0.07 M *n*-Bu_4NClO_4, after addition of 0.02 M **1n**; (c) DMF containing 0.07 M *n*-Bu_4NClO_4, after addition of 0.02 M **1n** and 55 M H_2O; (d) DMF containing 0.07 M *n*-Bu_4NClO_4, after addition of 0.02 M **1n**, 55 M H_2O and CO_2 saturated.

Considering that H_2O could promote the electro-carboxylation reaction of **1n** to give a higher 54% yield (without H_2O, 28% yield), the CV analysis of **1n** was then conducted. As shown in Figure S2, a one-electron reduction peak in the potential at -2.30 V and a second one at -2.49 V was observed (blue line). After addition of H_2O, there was no obvious changes on its first single-electron reduction process, but the current was increased from 0.28 to 0.32 mA at the second reduction wave (red line). These results indicate that H_2O might be able to react with the intermediate formed after two-electron transfer. After the solution of **1n** containing H_2O was saturated with CO_2, the first reduction peak disappeared and only one reduction peak was observed at -2.40 V with the peak current increased from 0.20 to 0.34 mA (ca. 1.7 times) (green line), which indicated that a fast chemical reaction might be occurred between CO_2 with the intermediate generated after the first single-electron reduction.

6.2 Controlled-potential electrolysis

Since there are two reduction peaks (-2.69 V and -2.94 V) were observed for the CV analysis of **1a**, the influence of potential on the reaction was further studied via controlled-potential electrolysis, which was conducted on Bio-logic VSP Potentiostat/Galvanostat, using a three electrodes system, with Pt-plate as working cathode and counter anode, the Ag/AgNO$_3$ (0.1 M in CH$_3$CN) as a reference electrode.

As shown in Table S7, when the potential less than -2.7 V (vs Ag/AgNO$_3$) was applied to the cathode, the efficiency of the reaction decreased precipitously with only 14% and 33% yield obtained at -2.5 V and -2.6 V, respectively (entries 1 and 2). At the potential of -2.7 V, the carboxylic acid could be obtained in 78% yield (entry 3). By increasing the potential to -2.8 V, a higher 88% yield was achieved (entry 4). Further enhancing the potential to -3.1 or 3.4 V only gave slightly increase of the reaction yield (entries 5-6). These results suggested that the first one-electron reduction process was critical to the reaction, and the thus generated radical anion should be the real species that reacted with CO_2.

Table S7. The influence of potential.

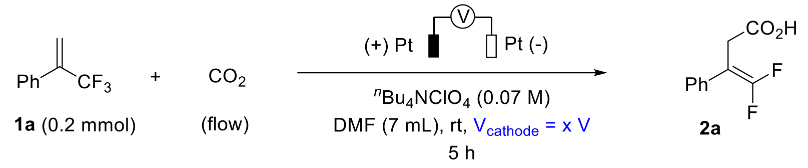

Entry	$V_{cathode}$ (x V)	Isolated yield (%)
1	-2.5	14
2	-2.6	33
3	-2.7	78
4	-2.8	88
5	-3.1	87
6	-3.4	90

6.3 Radical capture experiments

To get more evidence for the intermediacy of possible radical species, the following radical capture experiments in the presence or absence of CO_2 was performed.

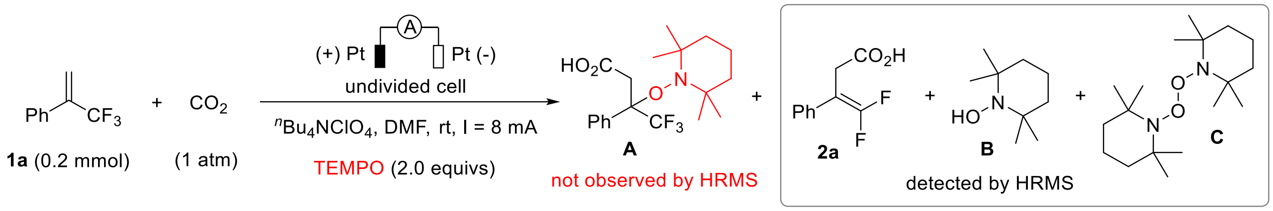

At first, the capture of possible radical intermediates generated after the addition of CO_2 was conducted based on the following procedure: To a 10 mL hydrogenation tube containing a stir bar were added $^nBu_4NClO_4$ (167.5 mg, 0.49 mmol), DMF (7.0 mL) and TEMPO (62.4 mg, 0.4 mmol), followed by the addition of **1a** (34.4 mg, 0.2 mmol). Then the tube was installed with two Pt plates (10 mm x 10 mm x 0.2 mm) as cathode and anode. After bubbling of CO_2 gas into the electrolytes for 10 min, the reaction mixture was electrolyzed under a constant current of 8 mA for 7 hours. The reaction mixture was separated into two portions, one was analyzed by HRMS directly and the other was analyzed after acidification. Unfortunately, both of which showed no incorporation of TEMPO, just unreacted styrene **1a**, carboxylation product **2a**, TEMPOH and $(TEMPO)_2$ were detected.

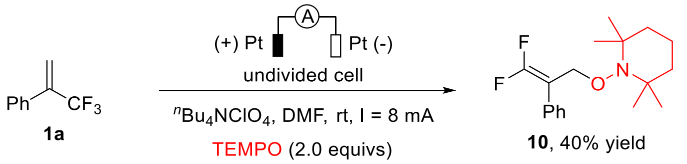

Then the reaction in the absence of CO_2 was conducted and a TEMPO adduct **10** of allyl radical was obtained based on the following procedure: To a 10 mL hydrogenation tube containing a stir bar were added $^nBu_4NClO_4$ (167.5 mg, 0.49 mmol, 0.07 M), DMF (7.0 mL) and TEMPO (62.4 mg, 0.4 mmol), followed by the addition of **1a** (34.4 mg, 0.2 mmol). Then the tube was installed with two Pt plates (10 mm x 10 mm x 0.2 mm) as cathode and anode. The reaction mixture was electrolyzed under a constant current of 8 mA for 7 hours. The reaction mixture was transferred to a 50 mL Erlenmeyer flask and acidized with HCl (2 N, 10 mL). The aqueous layer extracted with EtOAc (2 x 20 mL) and the combined organics were washed with sat. NH_4Cl (2 x 20 mL), dried over Na_2SO_4, and concentrated in vacuo. The residue was purified by column chromatography by using PE as eluate to afford **10** in 24.7 mg with 40% yield as colorless oil. ^1H NMR (400 MHz, $CDCl_3$): δ 7.45-7.43 (m, 2H), 7.38-7.34 (m, 2H), 7.31-7.26

(m, 1H), 4.55 (t, *J* = 2.8 Hz, 2H), 1.47-1.32 (m, 6H), 1.14 (s, 6H), 1.07 (s, 6H); ^{13}C NMR (100 MHz, $CDCl_3$): δ 155.62 (dd, *J* = 291.7 Hz, 289.8 Hz, 1C), 133.27 (dd, *J* = 3.9 Hz, 3.4 Hz, 1C), 128.71 (t, *J* = 3.2 Hz, 1C), 128.22, 127.38, 90.87 (dd, *J* = 20.2 Hz, 14.6 Hz, 1C), 72.72 (d, *J* = 5.0 Hz, 1C), 59.95, 39.79, 32.90, 20.11, 17.08; ^{19}F NMR (376 MHz, $CDCl_3$): δ -87.34 (d, *J* = 32.0 Hz, 1F), -88.46 (d, *J* = 31.6 Hz, 1F); HRMS (EI): Exact mass calcd for $C_{18}H_{25}NOF_2$ $[M]^+$: 309.1904, found: 309.1899.

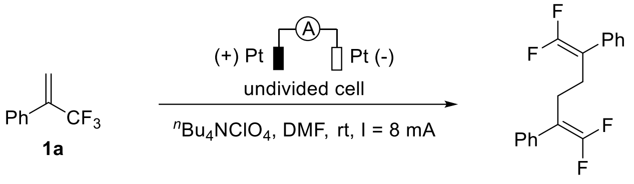

11, 33% yield

A S_N2'-type reaction of **1a** with TEMPO anion generated via electroreduction might also give the TEMPO adduct **10**. To rule out this possibility, we further conducted the reaction in the absence of CO_2 and TEMPO, and an allyl radical dimerization product **11** was successfully obtained based on the following procedure: To a 10 mL hydrogenation tube containing a stir bar were added ^{n}Bu$_4$NClO$_4$ (167.5 mg, 0.49 mmol), DMF (7.0 mL), followed by the addition of **1a** (34.4 mg, 0.2 mmol). Then the tube was installed with two Pt plates (10 mm x 10 mm x 0.2 mm) as cathode and anode. The reaction mixture was electrolyzed under a constant current of 8 mA. After 2 hours, the reaction mixture was transferred to a 50 mL Erlenmeyer flask and acidized with HCl (2 N, 10 mL). The aqueous layer extracted with EtOAc (2 x 20 mL) and the combined organics were washed with sat. NH_4Cl (2 x 20 mL), dried over Na_2SO_4, and concentrated in vacuo. The residue was purified by column chromatography by using PE as eluate to afford **11** in 10.1 mg with 33% yield as colorless oil. ^{1}H NMR (400 MHz, $CDCl_3$): δ 7.36-7.32 (m, 4H), 7.30-7.27 (m, 2H), 7.22-7.20 (m, 4H), 2.48 (t, *J* = 1.6 Hz, 4H); ^{13}C NMR (100 MHz, $CDCl_3$): δ 153.73 (dd, *J* = 289.4 Hz, 285.6 Hz), 133.12 (dd, *J* = 4.3 Hz, 3.2 Hz), 128.43, 128.17 (t, *J* = 3.3 Hz), 127.35, 91.53 (dd, *J* = 21.3 Hz, 13.7 Hz), 26.24-26.17 (m); ^{19}F NMR (376 MHz, $CDCl_3$): δ -90.72 (d, *J* = 41.4 Hz, 1F), -90.14 (d, *J* = 41.4 Hz, 1F); HRMS (EI): Exact mass calcd for $C_{18}H_{14}F_4$ $[M]^+$: 306.1032, found: 306.1030.

There results suggested that the radical anion should be generated during the reaction via one-electron reduction of α-trifluoromethyl alkenes, which could react with CO_2 immediately. While in the absence of CO_2, a defluorination process occurred to give the allyl radical.

As suggested by one reviewer, to identify the possibility for the generation of a CO_2 radical anion via the direct reduction of CO_2, one equivalent of styrene or 1,1-diphenylethylene was added to the reaction of **1a**. It was found that there was almost no influence on the carboxylation of **1a**, and no carboxylation product of styrene or 1,1-diphenylethylene was detected by NMR and LC-MS analysis. To avoid the possibility of carboxylic acids decomposing at the anode, the reaction using Mg instead of Pt-plate as anode was also performed, with similar phenomena observed. These results suggested that the CO_2 radical anion might not be involved during the reaction course.

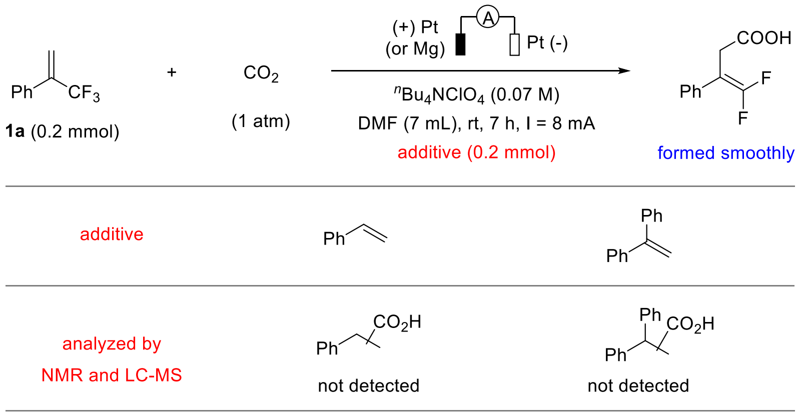

6.4 DFT calculation

To better understand the reaction mechanism, three possible reaction channels of the radical anion generated via the one-electron reduction of α-trifluoromethyl alkenes were studied by the theoretical calculations. The density functional theory (DFT) calculations were performed with Gaussian 09 program.[12] The Truhlar's M06-2X exchange-correlation functional[13] were used with the standard 6-311++G(d,p) basis set. The geometries of reactant, transition states, and product were fully optimized, followed by vibrational frequency calculations at the same levels of theory to obtain the zero-point energies (ZPE) and verify whether it is a transition state on the potential energy surfaces (PES). To estimate the bulk solvent effects on the reaction, all the structures were optimized in the DMF solvent with the polarized continuum model using the integral equation formalism variant (IEFPCM)[14]. The temperature-dependent enthalpy corrections and the entropy effects are computed at 298K and 1 atmosphere of pressure. Natural bond orbital (NBO) analysis[15] are also performed to compute the charge and spin distribution on the radical anion intermediate **II.**

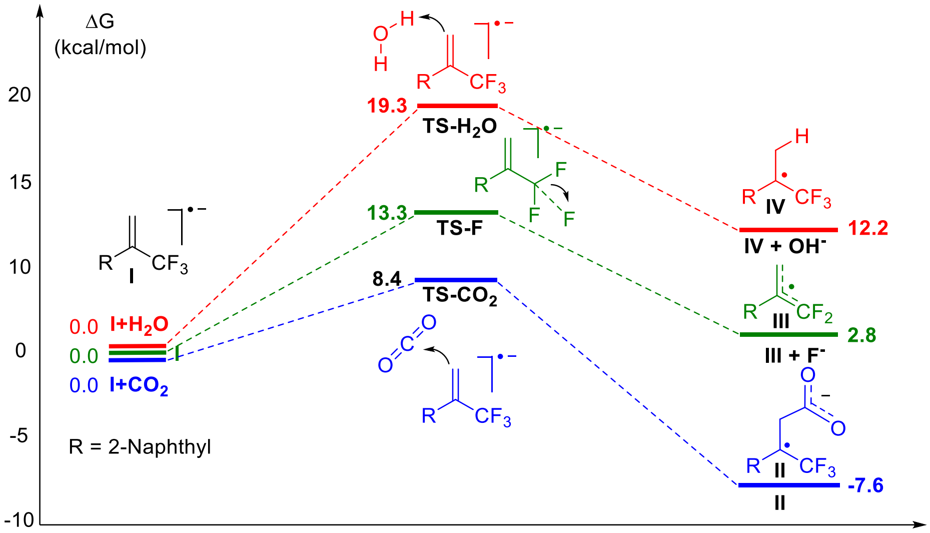

Figure S3. DFT calculation, Gibbs free energy profile for possible reaction channels at the IEFPCM-M06-2X/6-311++G(d,p) level in solvent DMF.

Three possible reaction pathways have been identified and the corresponding potential energy profile has been shown in Figure S3. The optimized structures of the reaction species were present in Figure S4. As shown in Figure S3, the addition of CO_2 to **I** has a low free energy barrier of 8.4 kcal/mol. More importantly, the reaction Gibbs free energy change ΔG of this reaction channel is negative, which indicates that the reaction via the pathway is thermodynamic spontaneous. But for the other two

pathways, fluorine anion releasing and H_2O addition pathways, their free energy barriers are 4.9 and 10.9 kcal/mol higher than that of the CO_2 addition pathway, respectively. In addition, the two pathways have positive the reaction Gibbs free energy change (+2.8 and +12.2 kcal/mol). Thus, the two reaction pathways should be not thermodynamic spontaneous. The present calculated results show that the reaction prefers to proceed though the CO_2 addition pathway, which are in good agreement with the experimental data.

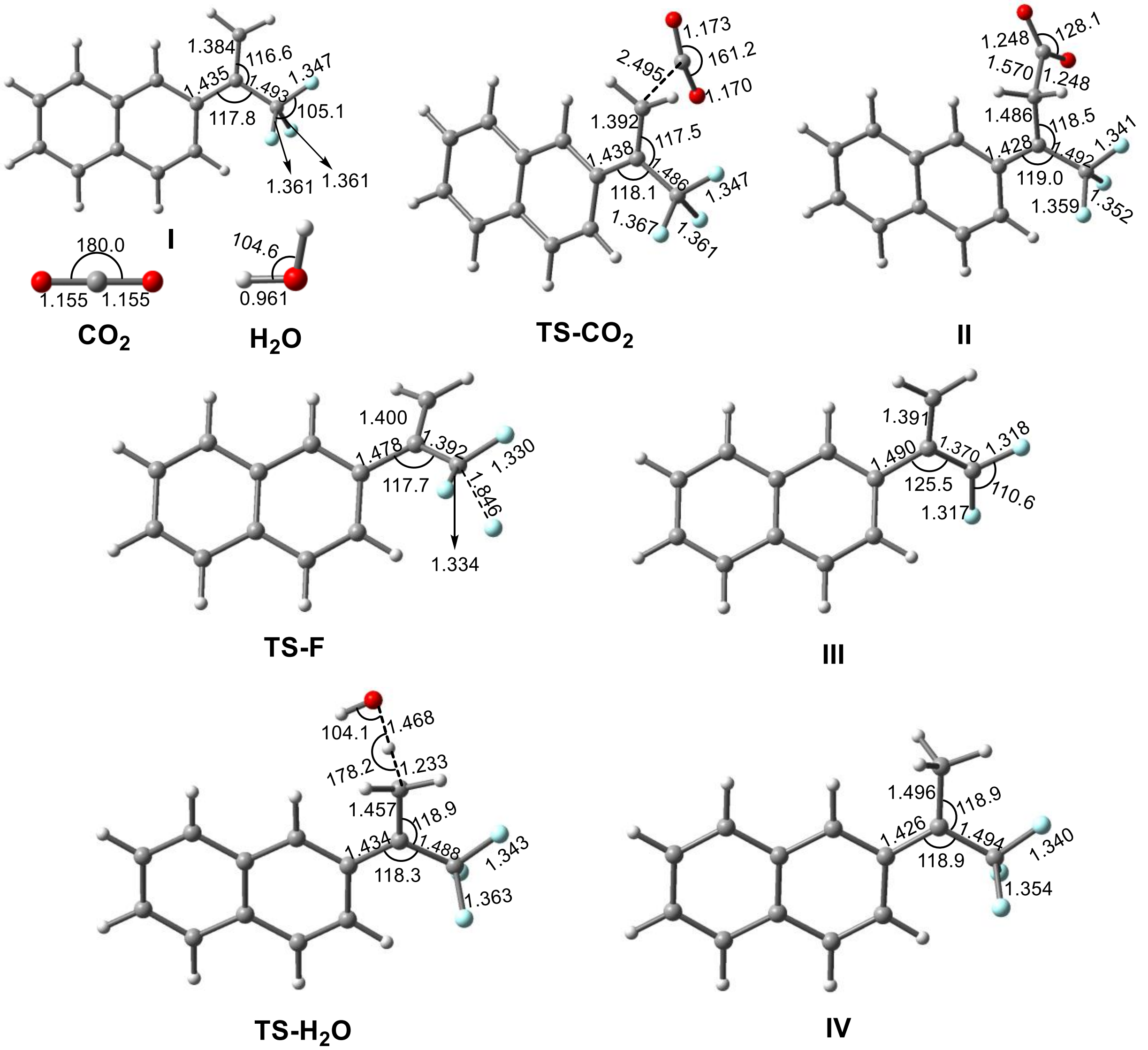

Figure S4. Optimized geometries of the reaction species at the IEFPCM-M06-2X/6-311++G(d,p) level in solvent DMF. The bond distances are in angstroms and the bond angles are in degrees. Carbon atoms are in grey, oxygen in red, hydrogen in white, and fluorine in grey blue.

In order to figure out where the radical and anion are situated in the intermediate **II**, the NBO analysis of which is performed. The nature charges and spins of the **II** are shown in Figure S5. The calculated charges show that the most negative charge located on the carboxylic group. Carbon atom C1 (see numbers in Figure S5) has a positive charge 0.81 but its two adjacent oxygen atoms have negative charge -0.82. Thus, the total net charges of the three atoms are -0.83. For the other carbon atoms, when the charges of their adjacent atoms such as hydrogen or fluorine atom were added to them, the total net charges of the corresponding group are very small. As a result, the most negative charge of molecule **II** is situated in the carboxylic group. In addition, NBO analysis also shows that the carbon atom C2 has the largest nature spin of 0.68, the single electron of radical should locate in the C2. According to the NBO analysis, we can speculate the structure of **II** should be more rational than that of **II'**.

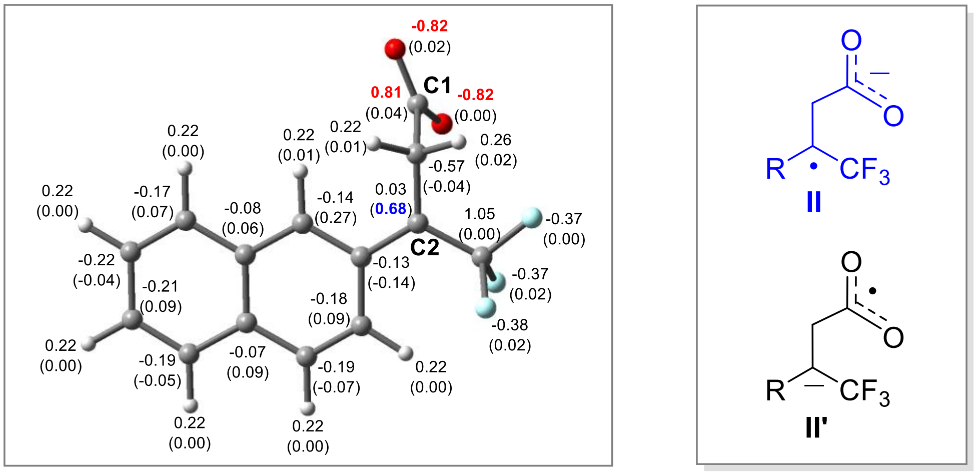

Figure S5. Nature charges and spins (in parenthesis) of the molecule **II** computed by NBO analysis at the IEFPCM-M06-2X/6-311++G(d,p) level in solvent dimethylformamide.

Coordinates for the optimized IMs and TSs at the IEFPCM-M06-2X/6-311++G(d,p).

I

Center	Atomic	Atomic	Coordinates (Angstroms)		
Number	Number	Type	X	Y	Z
1	6	0	-6.233394	-0.711006	0.207779
2	6	0	-4.855728	-0.732281	0.192644

3	6	0	-4.096678	0.469424	0.068836
4	6	0	-4.823112	1.698986	-0.038919
5	6	0	-6.227800	1.688194	-0.020415
6	6	0	-6.938315	0.504180	0.100977
7	1	0	-2.183452	-0.476462	0.135046
8	1	0	-6.782587	-1.641851	0.303152
9	1	0	-4.324213	-1.675411	0.275706
10	6	0	-2.688400	0.478114	0.050268
11	6	0	-4.058964	2.906412	-0.163073
12	1	0	-6.754859	2.634435	-0.103795
13	1	0	-8.021539	0.510084	0.113825
14	6	0	-2.694520	2.891487	-0.178985
15	6	0	-1.935739	1.675854	-0.073363
16	1	0	-4.589227	3.850206	-0.245680
17	1	0	-2.180990	3.840221	-0.275327
18	6	0	-0.500640	1.648161	-0.089449
19	6	0	0.218596	2.950103	-0.222467
20	6	0	0.284678	0.512515	0.007667
21	1	0	-0.157432	-0.467831	0.106319
22	1	0	1.362664	0.569532	-0.011807
23	9	0	1.559457	2.829371	-0.228486
24	9	0	-0.081270	3.625998	-1.365347
25	9	0	-0.053288	3.829925	0.779727

Zero-point correction= 0.182088 (A.U.)

Thermal correction to Gibbs Free Energy= 0.141857 (A.U.)

Sum of electronic and thermal Free Energies= -800.200311 (A.U.)

The number of imaginary frequencies: 0

II

Center Number	Atomic Number	Atomic Type	Coordinates (Angstroms) X	Y	Z
1	6	0	4.463917	1.265501	-0.129787
2	6	0	3.123371	1.495573	-0.306577
3	6	0	2.186064	0.433567	-0.190679
4	6	0	2.661022	-0.871416	0.114107
5	6	0	4.048225	-1.080031	0.290506
6	6	0	4.932624	-0.034852	0.171541
7	1	0	0.464820	1.653474	-0.581222
8	1	0	5.171631	2.080965	-0.220521
9	1	0	2.759280	2.490970	-0.537587
10	6	0	0.799993	0.644887	-0.367794
11	6	0	1.710673	-1.927439	0.233187
12	1	0	4.402622	-2.078683	0.522657
13	1	0	5.994151	-0.203164	0.308990
14	6	0	0.378297	-1.706023	0.063337
15	6	0	-0.127009	-0.393474	-0.251473
16	1	0	2.069094	-2.925236	0.463019
17	1	0	-0.305396	-2.539391	0.154776
18	6	0	-1.518392	-0.130133	-0.437512
19	6	0	-2.506834	-1.194716	-0.097270
20	6	0	-2.028030	1.197973	-0.867995
21	1	0	-1.394297	1.624207	-1.646043
22	1	0	-3.032186	1.109121	-1.286690
23	9	0	-3.776940	-0.774209	-0.185652
24	9	0	-2.415604	-2.274436	-0.916675
25	9	0	-2.356128	-1.682961	1.154070
26	6	0	-2.140158	2.239940	0.301325

27	8	0	-2.119676	3.436130	-0.053451
28	8	0	-2.267104	1.780877	1.454380

Zero-point correction= 0.198594 (A.U.)

Thermal correction to Gibbs Free Energy= 0.153026 (A.U.)

Sum of electronic and thermal Free Energies= -988.798975 (A.U.)

The number of imaginary frequencies: 0

III

Center Number	Atomic Number	Atomic Type	Coordinates (Angstroms) X	Y	Z
1	6	0	-3.917162	1.055080	-0.462074
2	6	0	-2.613393	1.476590	-0.445087
3	6	0	-1.568603	0.578157	-0.101408
4	6	0	-1.893168	-0.766905	0.222468
5	6	0	-3.252675	-1.174641	0.197617
6	6	0	-4.240789	-0.285480	-0.137008
7	1	0	0.032382	2.011969	-0.337376
8	1	0	-4.708993	1.746654	-0.724261
9	1	0	-2.360977	2.502190	-0.692934
10	6	0	-0.210205	0.986215	-0.077919
11	6	0	-0.842985	-1.659494	0.564376
12	1	0	-3.494445	-2.202466	0.447036
13	1	0	-5.276267	-0.604594	-0.154391
14	6	0	0.458818	-1.239054	0.579864
15	6	0	0.790139	0.105105	0.253784
16	1	0	-1.089240	-2.684381	0.820872
17	1	0	1.248365	-1.928541	0.855677
18	6	0	2.204083	0.573363	0.302322
19	6	0	3.164825	-0.190944	-0.306131

20	6	0	2.549434	1.759326	0.942061
21	1	0	1.793045	2.329185	1.462074
22	1	0	3.569424	2.117123	0.949332
23	9	0	4.447647	0.109991	-0.310894
24	9	0	2.943108	-1.286773	-1.002797

Zero-point correction= 0.180031 (A.U.)

Thermal correction to Gibbs Free Energy= 0.140494 (A.U.)

Sum of electronic and thermal Free Energies= -700.204179 (A.U.)

The number of imaginary frequencies: 0

IV

Center Number	Atomic Number	Atomic Type	Coordinates (Angstroms) X	Y	Z
1	6	0	4.305698	0.984818	-0.000087
2	6	0	3.022671	1.469447	-0.000154
3	6	0	1.915080	0.579340	-0.000081
4	6	0	2.157771	-0.821522	0.000050
5	6	0	3.489956	-1.293154	0.000119
6	6	0	4.543008	-0.409662	0.000054
7	1	0	0.430879	2.125600	-0.000246
8	1	0	5.145185	1.669721	-0.000141
9	1	0	2.835392	2.537828	-0.000259
10	6	0	0.584380	1.053143	-0.000119
11	6	0	1.036830	-1.703414	0.000100
12	1	0	3.667489	-2.363197	0.000222
13	1	0	5.561581	-0.778925	0.000113
14	6	0	-0.239454	-1.231786	0.000049
15	6	0	-0.511317	0.184964	-0.000029

16	1	0	1.218095	-2.772835	0.000196
17	1	0	-1.056997	-1.940031	0.000110
18	6	0	-1.833933	0.718384	-0.000046
19	6	0	-2.993035	-0.224676	-0.000074
20	6	0	-2.098846	2.190332	0.000341
21	1	0	-1.653858	2.663695	-0.880497
22	1	0	-3.162770	2.410628	0.001048
23	9	0	-4.178814	0.399013	-0.000341
24	9	0	-2.999596	-1.043750	-1.077811
25	9	0	-2.999939	-1.043395	1.077929
26	1	0	-1.652713	2.663414	0.880735

Zero-point correction= 0.196048 (A.U.)

Thermal correction to Gibbs Free Energy= 0.154893 (A.U.)

Sum of electronic and thermal Free Energies= -800.682324 (A.U.)

The number of imaginary frequencies: 0

TS-CO_2

Center Number	Atomic Number	Atomic Type	Coordinates (Angstroms) X	Y	Z
1	6	0	4.471396	1.225707	-0.264241
2	6	0	3.134728	1.430596	-0.509597
3	6	0	2.179802	0.396037	-0.294199
4	6	0	2.654120	-0.859727	0.187962
5	6	0	4.032282	-1.042624	0.428627
6	6	0	4.933692	-0.022898	0.209335
7	1	0	0.479279	1.557798	-0.882142
8	1	0	5.180490	2.028326	-0.434729
9	1	0	2.786030	2.392115	-0.873567
10	6	0	0.800306	0.582309	-0.535839

11	6	0	1.690750	-1.890511	0.411815
12	1	0	4.373448	-2.006507	0.794393
13	1	0	5.989779	-0.173571	0.398739
14	6	0	0.363526	-1.690054	0.172437
15	6	0	-0.144412	-0.436769	-0.321809
16	1	0	2.034832	-2.852984	0.777953
17	1	0	-0.318872	-2.511852	0.350568
18	6	0	-1.539260	-0.223869	-0.598814
19	6	0	-2.510236	-1.244225	-0.125748
20	6	0	-2.052331	0.929467	-1.185647
21	1	0	-1.398750	1.599847	-1.727123
22	1	0	-3.109763	1.017529	-1.393865
23	9	0	-3.795327	-0.870963	-0.279106
24	9	0	-2.411314	-2.444753	-0.772035
25	9	0	-2.384394	-1.563733	1.191198
26	6	0	-2.119719	2.626957	0.641496
27	8	0	-2.253790	3.579398	-0.029183
28	8	0	-2.004338	1.943350	1.583507

Zero-point correction= 0.194700 (A.U.)

Thermal correction to Gibbs Free Energy= 0.148535 (A.U.)

Sum of electronic and thermal Free Energies= -988.773493 (A.U.)

The number of imaginary frequencies: 1

Imaginary frequency -313.5464 cm^{-1}; IR Intensity 3260.3165 KM/Mole

TS-F

Center Number	Atomic Number	Atomic Type	Coordinates (Angstroms) X	Y	Z
1	6	0	-4.309307	0.938599	0.199774
2	6	0	-3.037239	1.450941	0.193669

3	6	0	-1.910476	0.597659	0.043111
4	6	0	-2.126362	-0.800466	-0.098729
5	6	0	-3.453131	-1.301915	-0.090755
6	6	0	-4.521851	-0.454300	0.055425
7	1	0	-0.434144	2.167606	0.164331
8	1	0	-5.159929	1.600419	0.314432
9	1	0	-2.872391	2.517923	0.303116
10	6	0	-0.584249	1.100052	0.038478
11	6	0	-0.994825	-1.646752	-0.249674
12	1	0	-3.607969	-2.370339	-0.201635
13	1	0	-5.532177	-0.845871	0.061776
14	6	0	0.273269	-1.134769	-0.255079
15	6	0	0.505602	0.266294	-0.101734
16	1	0	-1.154368	-2.713029	-0.374871
17	1	0	1.128110	-1.784757	-0.402585
18	6	0	1.879264	0.810524	-0.144395
19	6	0	2.912420	-0.006010	0.308218
20	6	0	2.160706	2.056285	-0.718693
21	1	0	1.383332	2.614211	-1.222315
22	1	0	3.164554	2.457200	-0.720254
23	9	0	4.124761	0.517623	0.465028
24	9	0	2.701874	-0.917303	1.259444
25	9	0	3.431371	-1.255899	-0.946659

Zero-point correction= 0.194700 (A.U.)

Thermal correction to Gibbs Free Energy= 0.148535 (A.U.)

Sum of electronic and thermal Free Energies= -800.179095 (A.U.)

The number of imaginary frequencies: 1

Imaginary frequency -313.5464 cm^{-1}; IR Intensity 3260.3165 KM/Mole

TS-H_2O

Center Number	Atomic Number	Atomic Type	Coordinates (Angstroms) X	Y	Z
1	6	0	4.357809	0.979362	-0.193000
2	6	0	3.050325	1.359262	-0.362313
3	6	0	1.994664	0.418504	-0.212374
4	6	0	2.322092	-0.924845	0.120862
5	6	0	3.678081	-1.289327	0.288025
6	6	0	4.678660	-0.359180	0.135341
7	1	0	0.418050	1.822409	-0.609419
8	1	0	5.152695	1.706622	-0.311239
9	1	0	2.802726	2.385340	-0.614268
10	6	0	0.640130	0.786575	-0.379744
11	6	0	1.255666	-1.857792	0.276016
12	1	0	3.915689	-2.317898	0.540183
13	1	0	5.715142	-0.646536	0.265863
14	6	0	-0.044593	-1.485167	0.111860
15	6	0	-0.402267	-0.131947	-0.231325
16	1	0	1.496455	-2.885749	0.527429
17	1	0	-0.818641	-2.232102	0.231963
18	6	0	-1.759057	0.286681	-0.429026
19	6	0	-2.849399	-0.641380	-0.024293
20	6	0	-2.107983	1.623974	-0.890214
21	1	0	-1.454854	1.967144	-1.696776
22	1	0	-3.150393	1.719341	-1.188264
23	9	0	-4.068270	-0.079659	-0.074526
24	9	0	-2.927175	-1.754413	-0.806384
25	9	0	-2.716756	-1.116954	1.240905
26	8	0	-1.612963	3.366620	1.112129

27	1	0	-1.902695	2.420892	0.027719
28	1	0	-0.745415	3.098499	1.430935

Zero-point correction= 0.202795 (A.U.)

Thermal correction to Gibbs Free Energy= 0.158578 (A.U.)

Sum of electronic and thermal Free Energies= -876.594417 (A.U.)

The number of imaginary frequencies: 1

Imaginary frequency -707.1457 cm^{-1}; IR Intensity 7788.7992 KM/Mole

6.5 Anodic Oxidation.

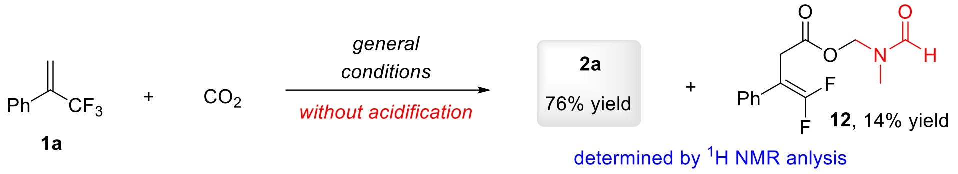

Since a non-sacrificial anode was employed, to identify the real sacrificial oxidant on anode, we analyzed the reaction mixture directly without acidification, based on the following procedure: To a 10 mL hydrogenation tube containing a stir bar were added $^{n}Bu_4NClO_4$ (167.5 mg, 0.49 mmol), DMF (7.0 mL), followed by the addition of **1a** (0.2 mmol). Then the tube was installed with two Pt-plates as cathode and anode. After bubbling of CO_2 gas into the electrolytes for 10 min, the reaction mixture was electrolyzed under a constant current of 8 mA for 7 hours. After that, the reaction mixture was transferred to a 50 mL Erlenmeyer flask and quenched with sat. NaCl (20 mL). The aqueous layer extracted with EtOAc (20 mL) and dried over Na_2SO_4. After concentrated in vacuo, the residue was analyzed by ^{1}H NMR with benzotrifluoride (29.3 mg, 0.2 mmol) as internal standard. As shown in Figure S6, the formation of carboxylate **12** was detected in 14% yield, along with the generation of carboxylic acid **2a** in 76% yield, which indicated that a Shono oxidation of DMF might be occurred.

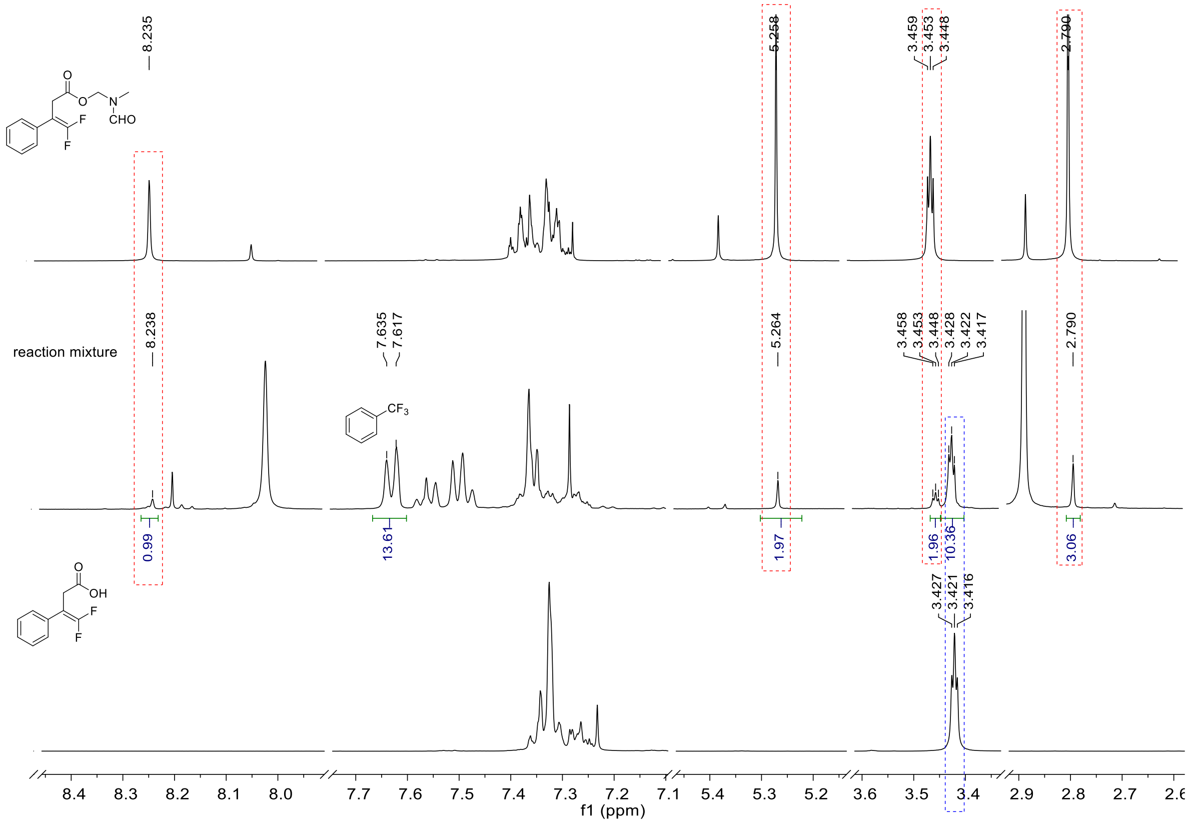

Figure S6. ^{1}H NMR of reaction mixture without acidification

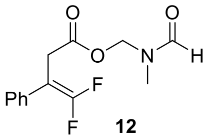

12

Product **12** could be obtained by column chromatography with CH_2Cl_2/Et_2O (9:1, v/v) as eluate, which is colorless oil. ^1H NMR (400 MHz, $CDCl_3$): δ 8.16 (s, 1H), 7.31-7.27 (m, 2H), 7.26-7.19 (m, 3H), 5.18 (s, 2H), 3.38 (t, J = 2.4 Hz, 2H), 2.72 (s, 3H); ^{13}C NMR (100 MHz, $CDCl_3$): δ 169.86 (dd, J = 4.3 Hz, 2.6 Hz, 1C), 163.76, 154.82 (dd, J = 291.6 Hz, 288.0 Hz, 1C), 132.49 (t, J = 3.8 Hz, 1C), 128.70, 127.86, 127.72 (t, J = 3.4 Hz, 1C), 86.72 (dd, J = 21.1 Hz, 18.2 Hz, 1C), 74.19, 33.76 (d, J = 2.7 Hz, 1C), 29.60; ^{19}F NMR (376 MHz, $CDCl_3$): δ -87.44 (d, J = 34.2 Hz, 1F), -88.53 (d, J = 33.8 Hz, 1F); IR (ATR) ν 3495.0, 2980.0, 1743.6, 1683.8, 1498.6, 1394.5, 1311.5, 1244.0, 1155.3, 1070.4 cm^{-1}; HRMS (ESI): Exact mass calcd for $C_{13}H_{13}F_2NNaO_3$ $[M+Na]^+$: 292.0756, found: 292.0755.

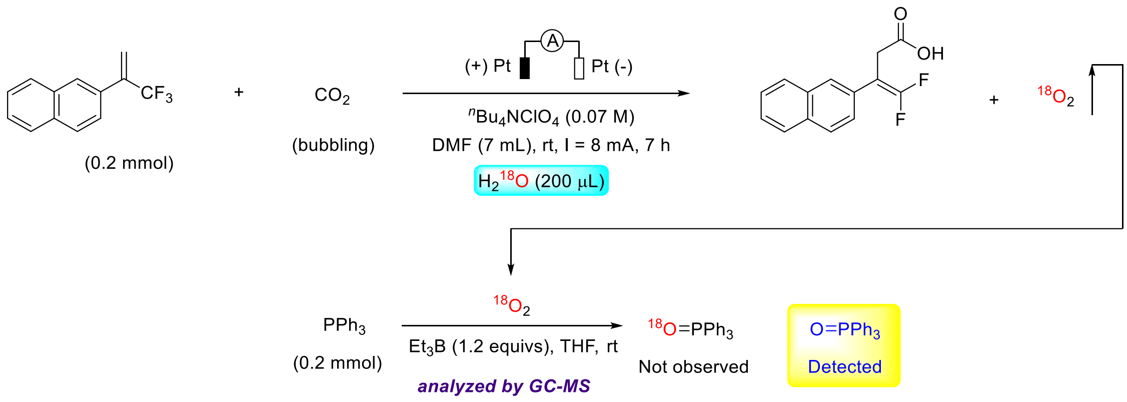

Figure S7. Oxygen capture experiment

Inspired by the Cheng's latest work,[16] the capture of oxygen generated via the potential anode oxidation of H_2O was conducted by using labeled $H_2{}^{18}O$. As shown in Figure S7, if $^{18}O_2$ generated through the anode oxidation of $H_2{}^{18}O$, it could oxidize PPh_3 to $^{18}O{=}PPh_3$, which could be detected by MS analysis. Unfortunately, the formation of $O{=}PPh_3$ was detected instead of $^{18}O{=}PPh_3$. Meanwhile, GC-MS analysis revealed that $H_2{}^{18}O$ in the reaction system was disappeared and replaced by H_2O. It was speculated that during the reaction course, the reversible reaction of $H_2{}^{18}O$ and bubbling CO_2 lead to the generation of H_2O and $C^{18}O_2$, and ultimately resulted in the formation of $O{=}PPh_3$. As O_2 penetrating plastic tube readily, the influence of environment O_2 could not be excluded. However, based on the fact that H_2O possesses lower oxidation potential than DMF (1.23 and 1.9 V vs. SHE, respectively),[17] it was more likely act as sacrificial oxidant.

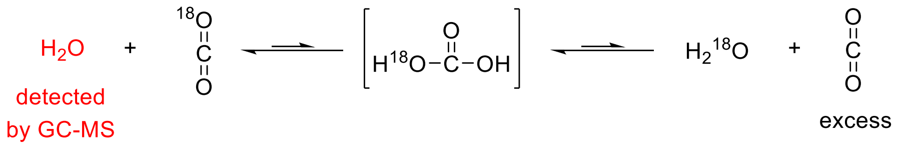

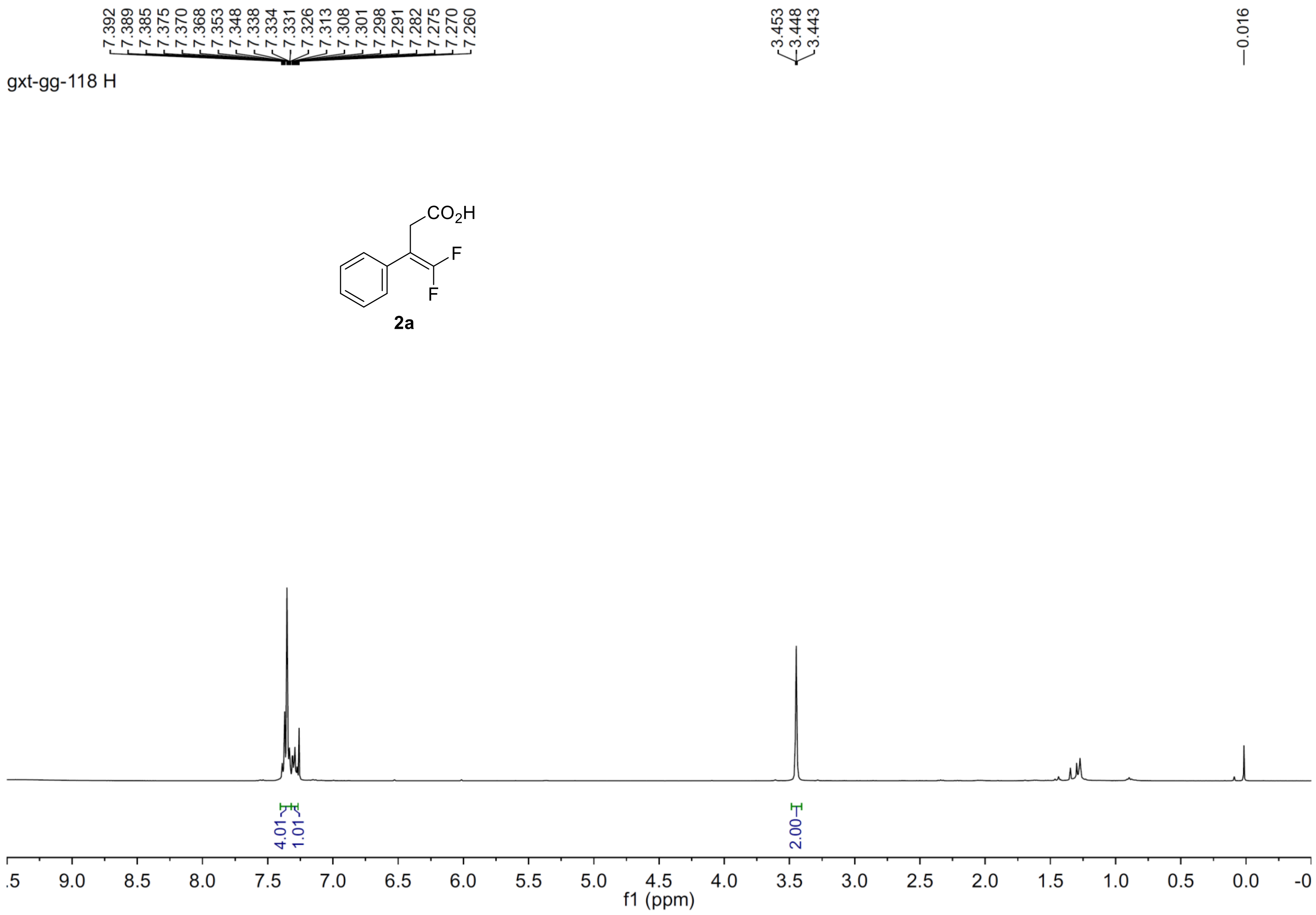

7.392
7.389
7.385
7.375
7.370
7.368
7.353
7.348
7.338
7.334
7.331
7.326
7.313
7.308
7.301
7.298
7.291
7.282
7.275
7.270
7.260
3.453
3.448
3.443
0.016
gxt-gg-118 H
CO2H
F
F
2a
4.01
1.01
2.00
.5
9.0
8.5
8.0
7.5
7.0
6.5
6.0
5.5
5.0
4.5
4.0
3.5
3.0
2.5
2.0
1.5
1.0
0.5
0.0
-0
f1 (ppm)

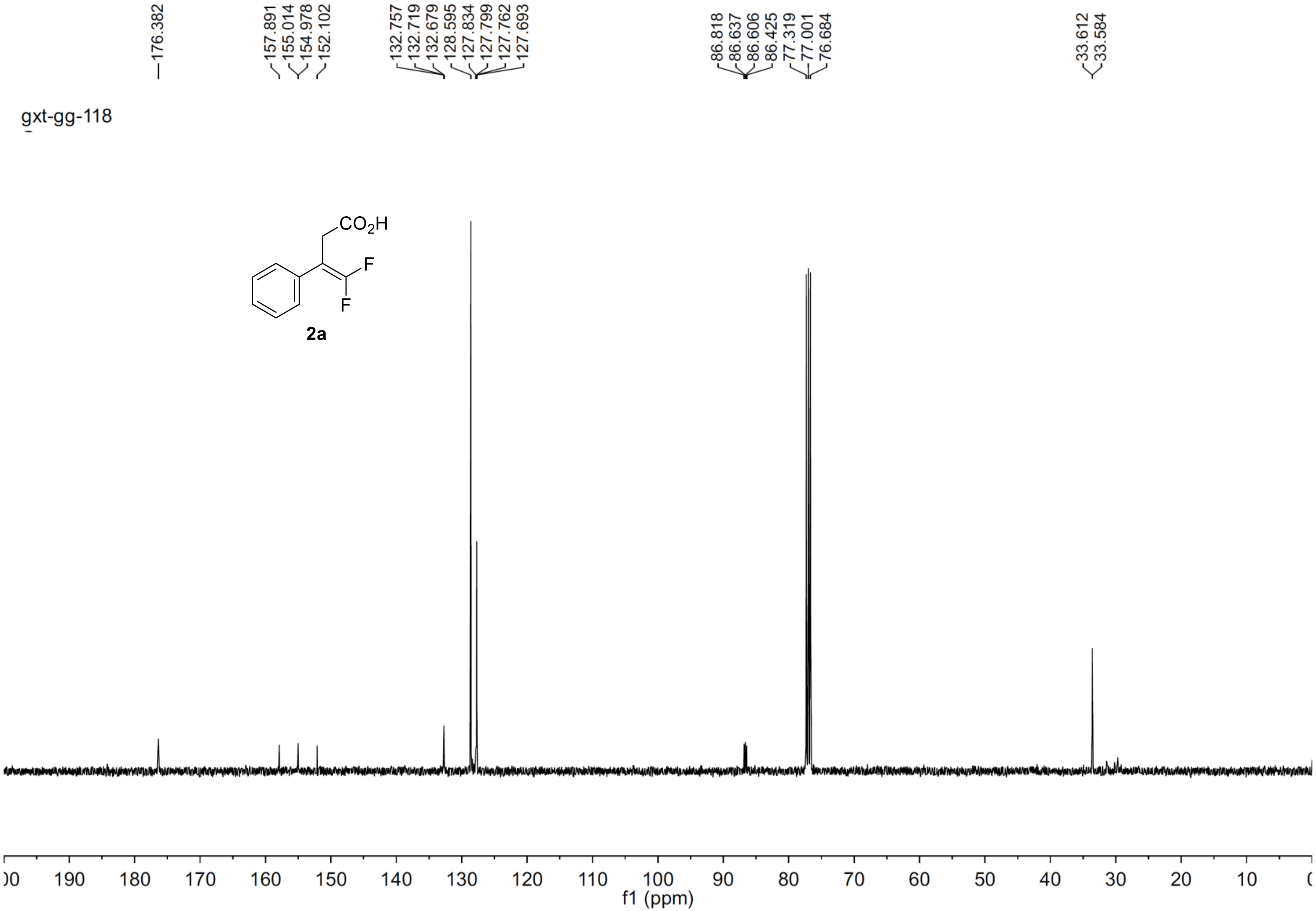
176.382
157.891
155.014
154.978
152.102
132.757
132.719
132.679
128.595
127.834
127.799
127.762
127.693
86.818
86.637
86.606
86.425
77.319
77.001
76.684
33.612
33.584
gxt-gg-118
CO2H
F
F
2a
190 180 170 160 150 140 130 120 110 100 90 80 70 60 50 40 30 20 10
f1 (ppm)

gxt-gg-118 F

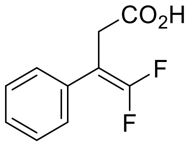

2a

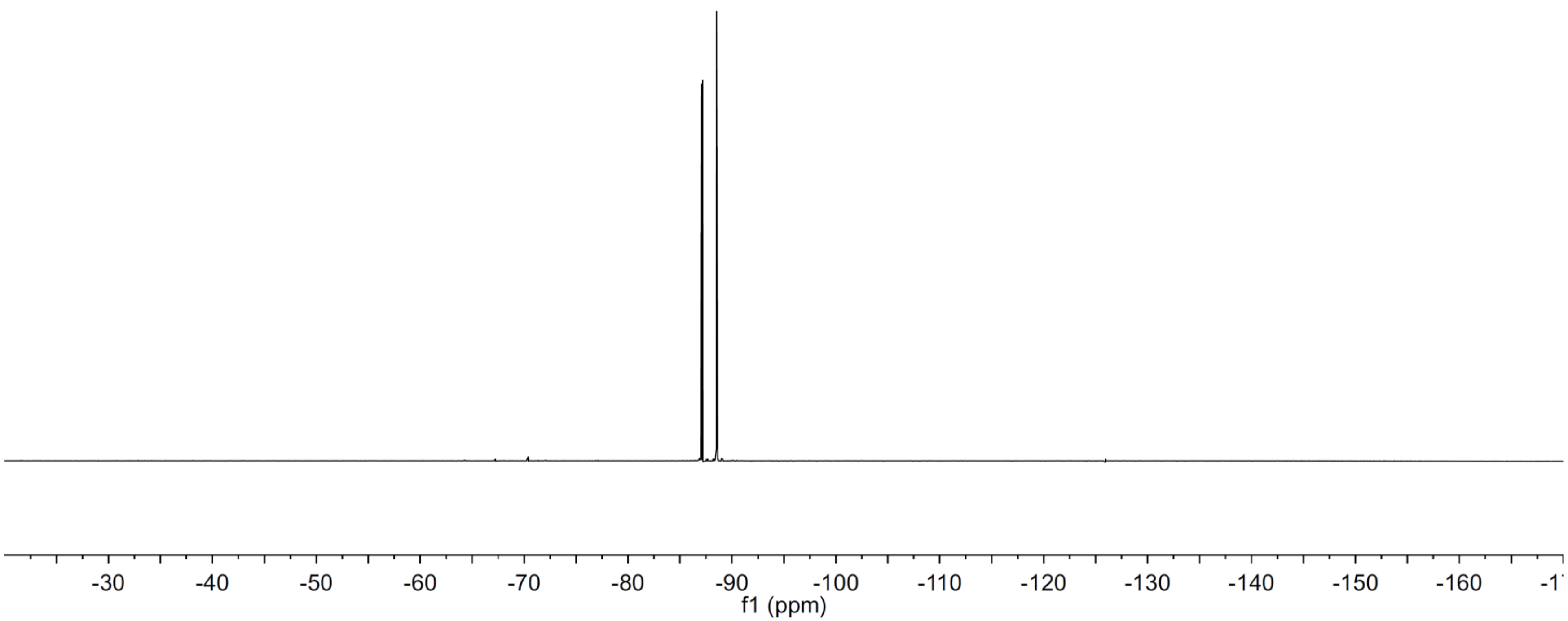

gxt-gh-114 H

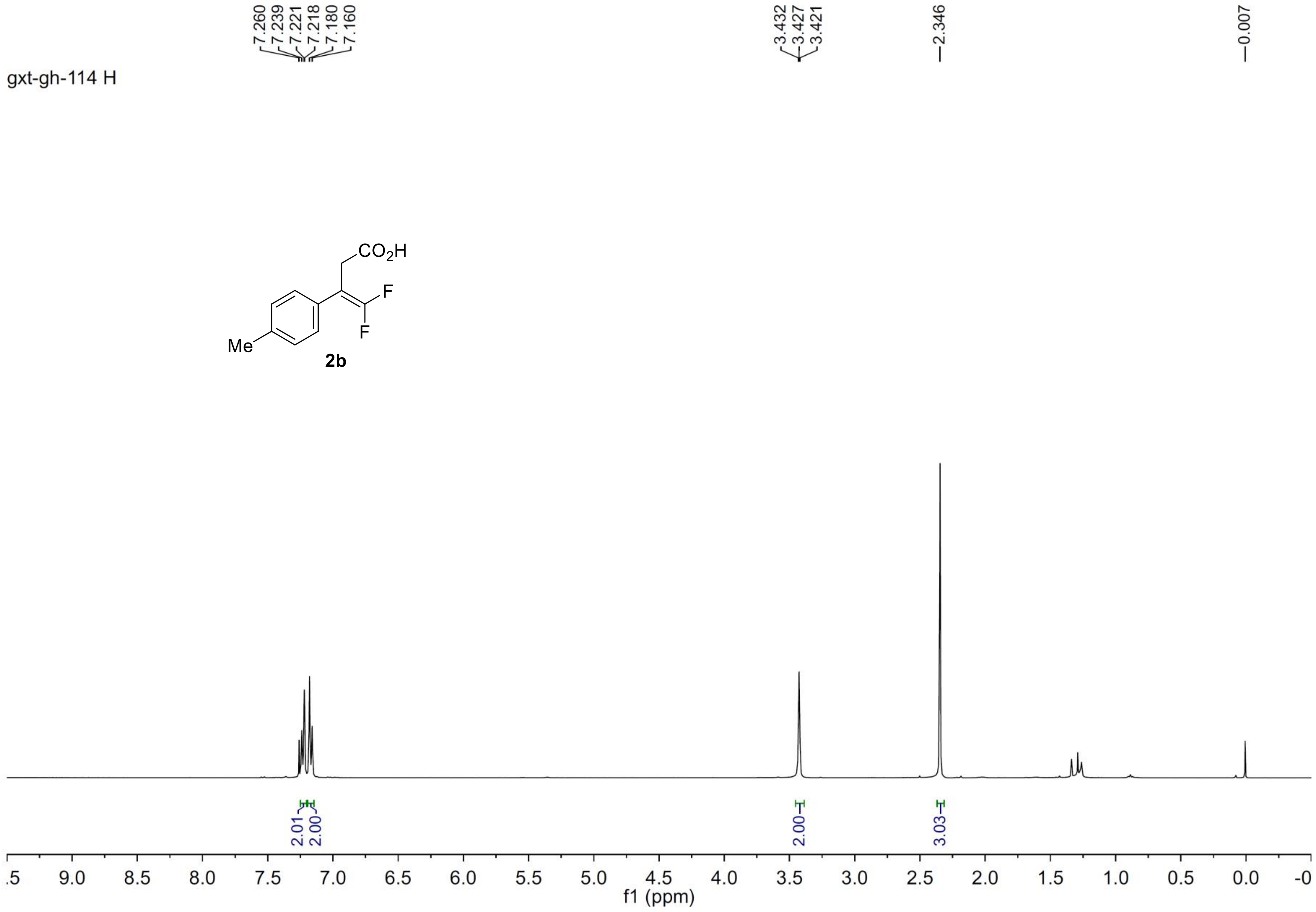

7.260
7.239
7.221
7.218
7.180
7.160
3.432
3.427
3.421
2.346
0.007
CO_2H
F
F
Me
2b
2.01
2.00
2.00
3.03
.5 9.0 8.5 8.0 7.5 7.0 6.5 6.0 5.5 5.0 4.5 4.0 3.5 3.0 2.5 2.0 1.5 1.0 0.5 0.0 -0
f1 (ppm)

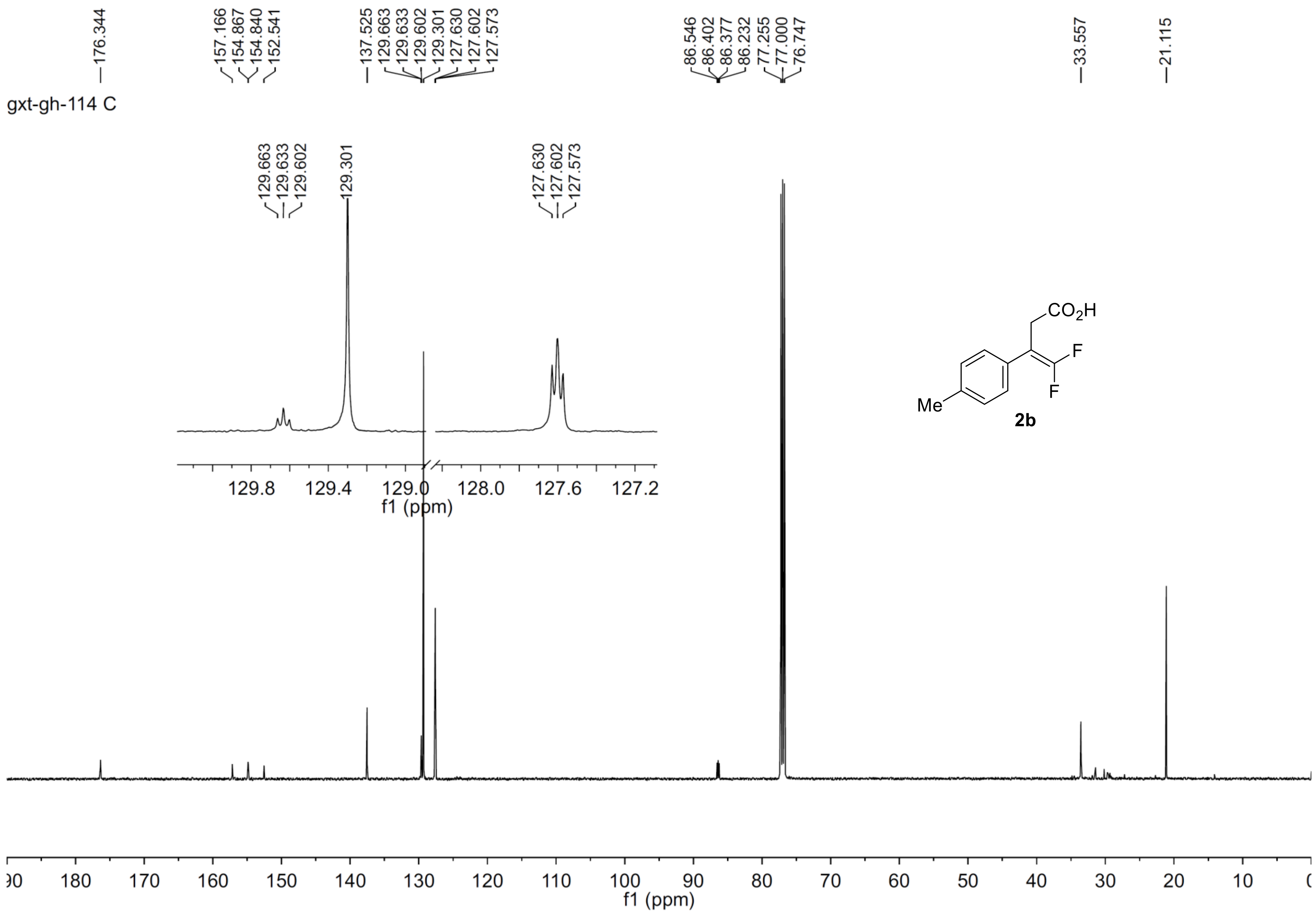
gxt-gh-114 C
176.344
157.166
154.867
154.840
152.541
137.525
129.663
129.633
129.602
129.301
127.630
127.602
127.573
86.546
86.402
86.377
86.232
77.255
77.000
76.747
33.557
21.115
129.8 129.4 129.0 128.0 127.6 127.2
f1 (ppm)
CO2H
F
F
Me
2b
180 170 160 150 140 130 120 110 100 90 80 70 60 50 40 30 20 10
f1 (ppm)

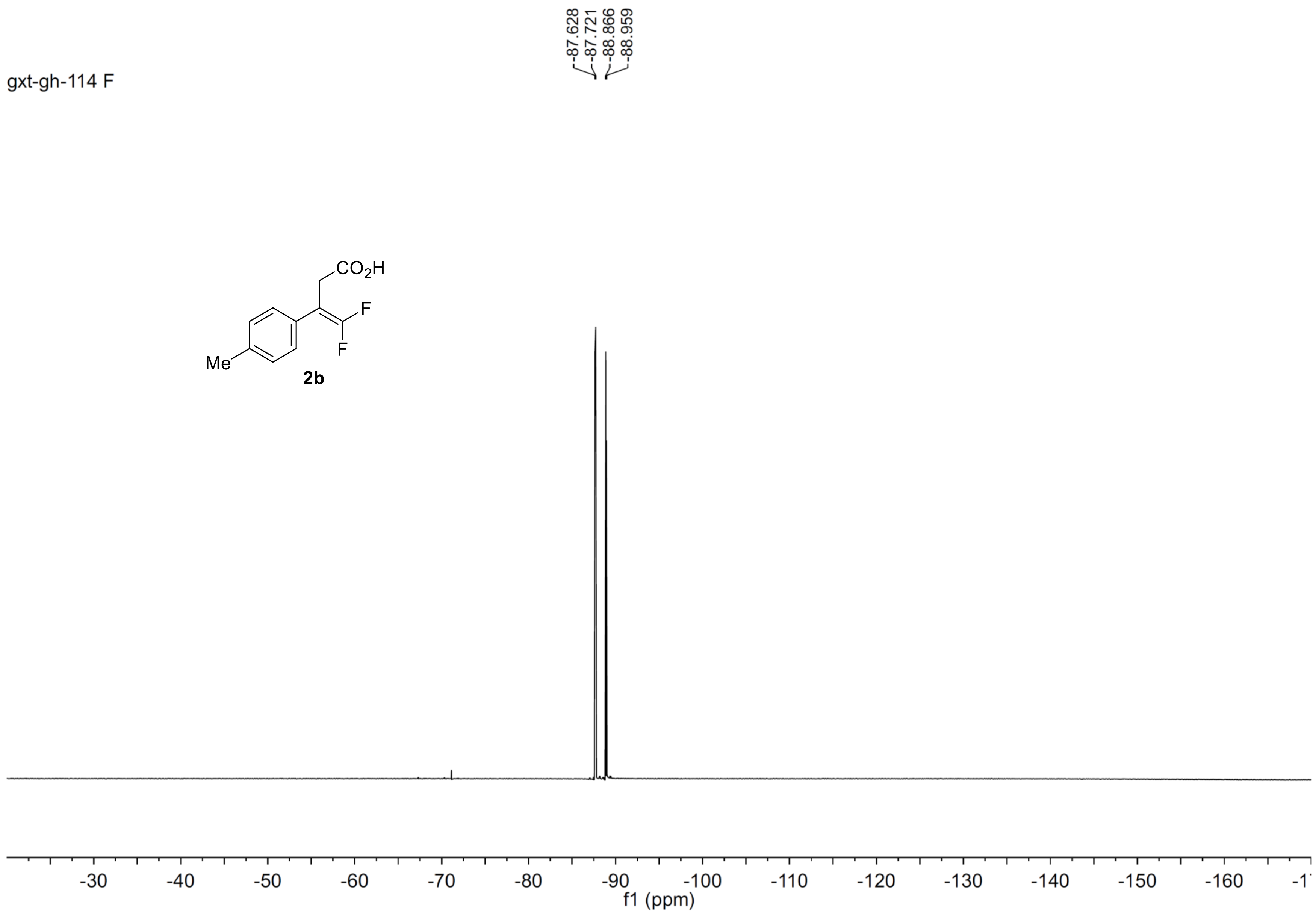
gxt-gh-114 F
-87.628
-87.721
-88.866
-88.959
CO2H
F
F
Me
2b
-30
-40
-50
-60
-70
-80
-90
-100
-110
-120
-130
-140
-150
-160
f1 (ppm)

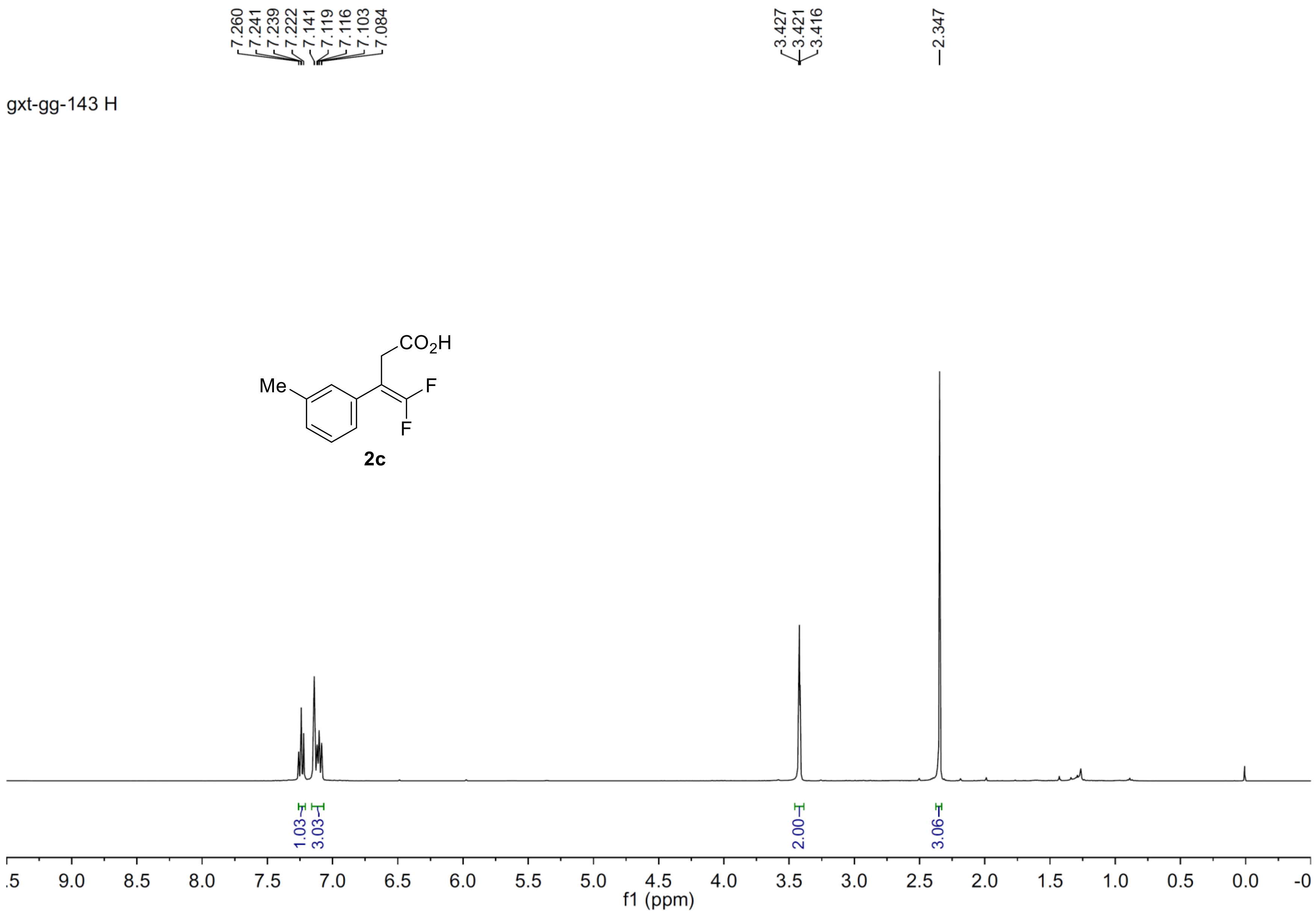
gxt-gg-143 H
7.260
7.241
7.239
7.222
7.141
7.119
7.116
7.103
7.084
3.427
3.421
3.416
2.347
CO2H
Me
F
F
2c
1.03
3.03
2.00
3.06
.5
9.0
8.5
8.0
7.5
7.0
6.5
6.0
5.5
5.0
4.5
4.0
3.5
3.0
2.5
2.0
1.5
1.0
0.5
0.0
-0
f1 (ppm)

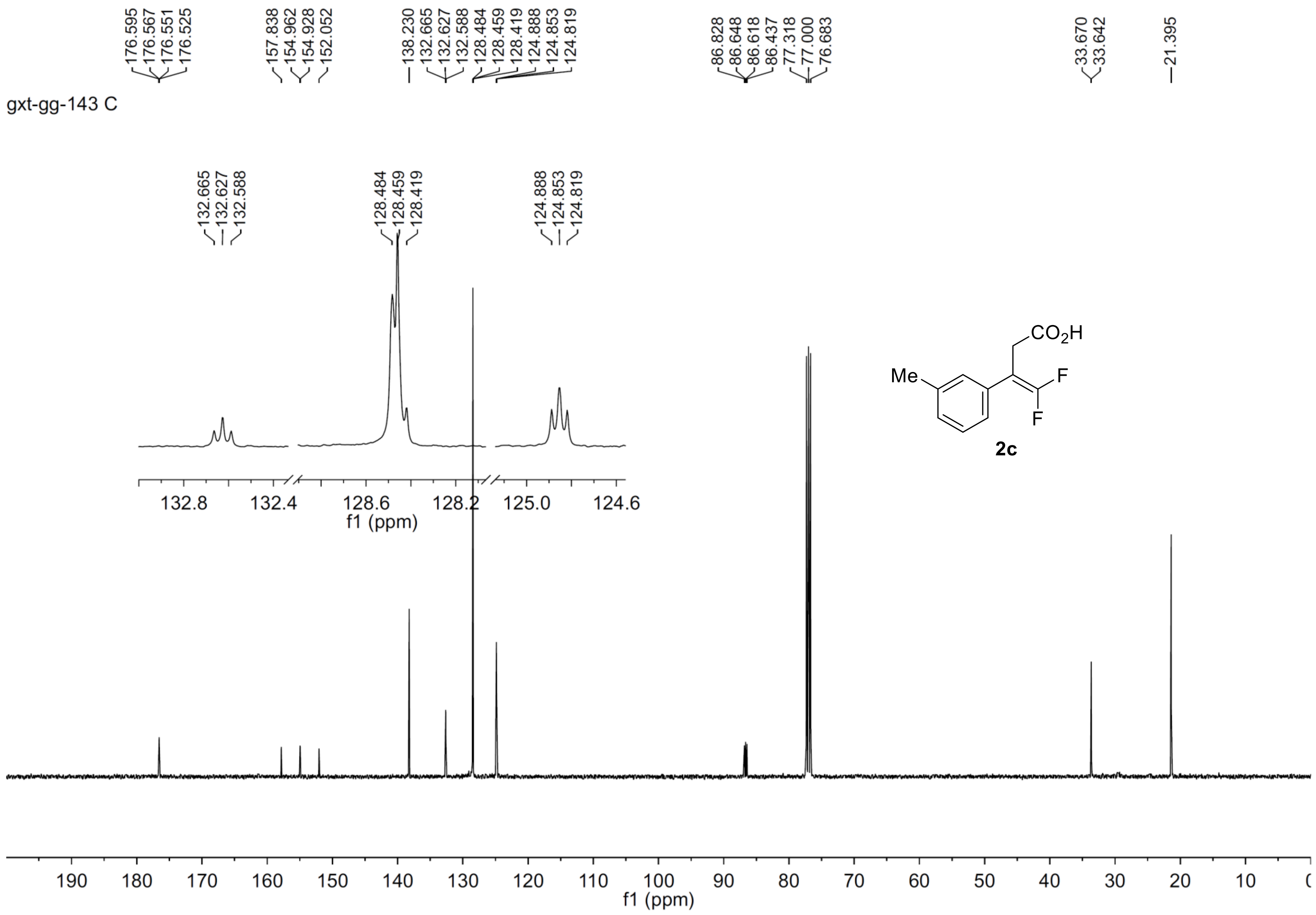
gxt-gg-143 C
176.595
176.567
176.551
176.525
157.838
154.962
154.928
152.052
138.230
132.665
132.627
132.588
128.484
128.459
128.419
124.888
124.853
124.819
86.828
86.648
86.618
86.437
77.318
77.000
76.683
33.670
33.642
21.395
CO2H
Me
F
F
2c
132.8
132.4
128.6
128.2
125.0
124.6
f1 (ppm)
190 180 170 160 150 140 130 120 110 100 90 80 70 60 50 40 30 20 10
f1 (ppm)

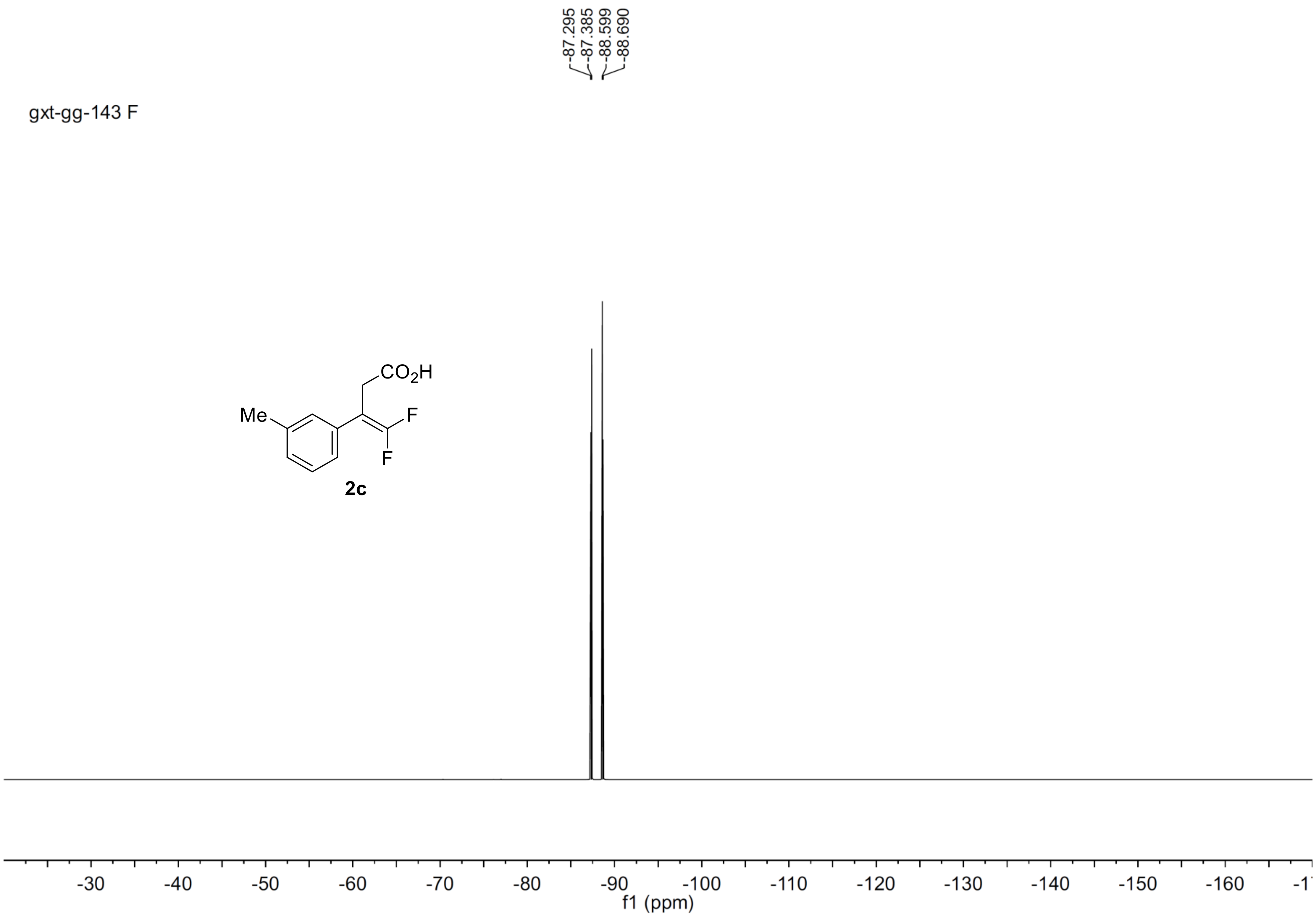
-87.295
-87.385
-88.599
-88.690
gxt-gg-143 F
Me
CO2H
F
F
2c
-30
-40
-50
-60
-70
-80
-90
-100
-110
-120
-130
-140
-150
-160
f1 (ppm)

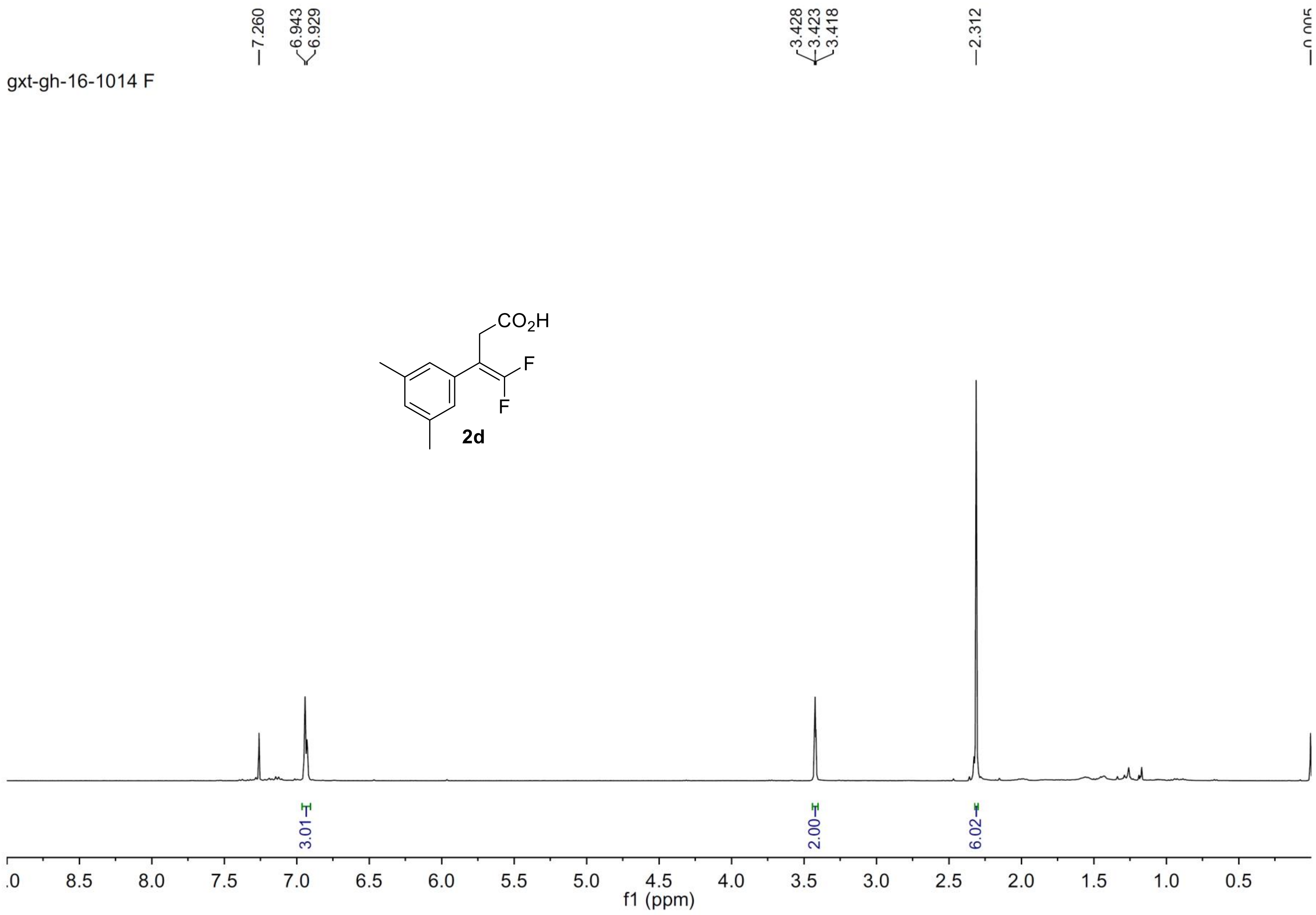
gxt-gh-16-1014 F
7.260
6.943
6.929
3.428
3.423
3.418
2.312
0.005
CO2H
F
F
2d
3.01
2.00
6.02
.0
8.5
8.0
7.5
7.0
6.5
6.0
5.5
5.0
4.5
4.0
3.5
3.0
2.5
2.0
1.5
1.0
0.5
f1 (ppm)

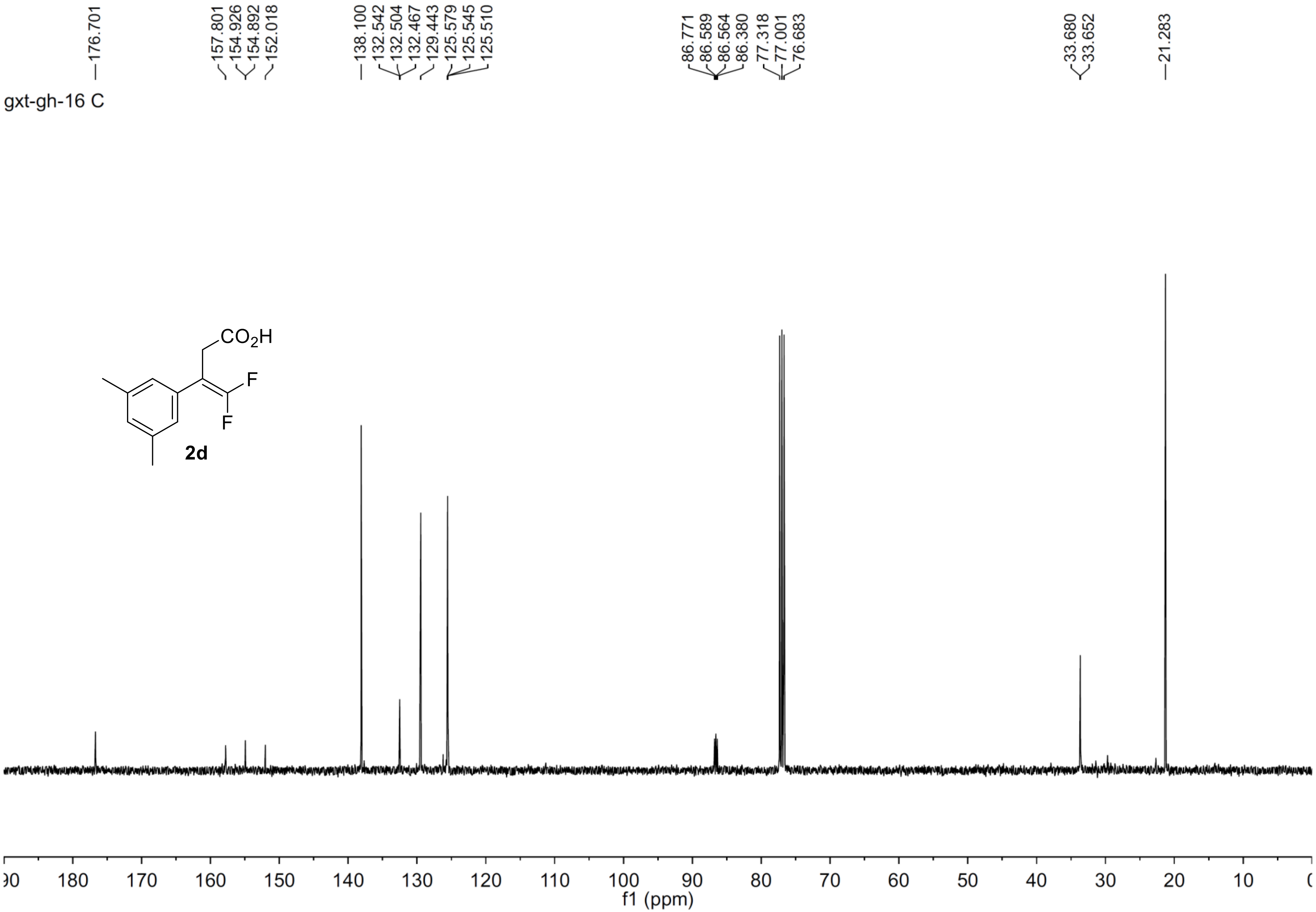
gxt-gh-16 C
176.701
157.801
154.926
154.892
152.018
138.100
132.542
132.504
132.467
129.443
125.579
125.545
125.510
86.771
86.589
86.564
86.380
77.318
77.001
76.683
33.680
33.652
21.283
CO2H
F
F
2d
180
170
160
150
140
130
120
110
100
90
80
70
60
50
40
30
20
10
f1 (ppm)

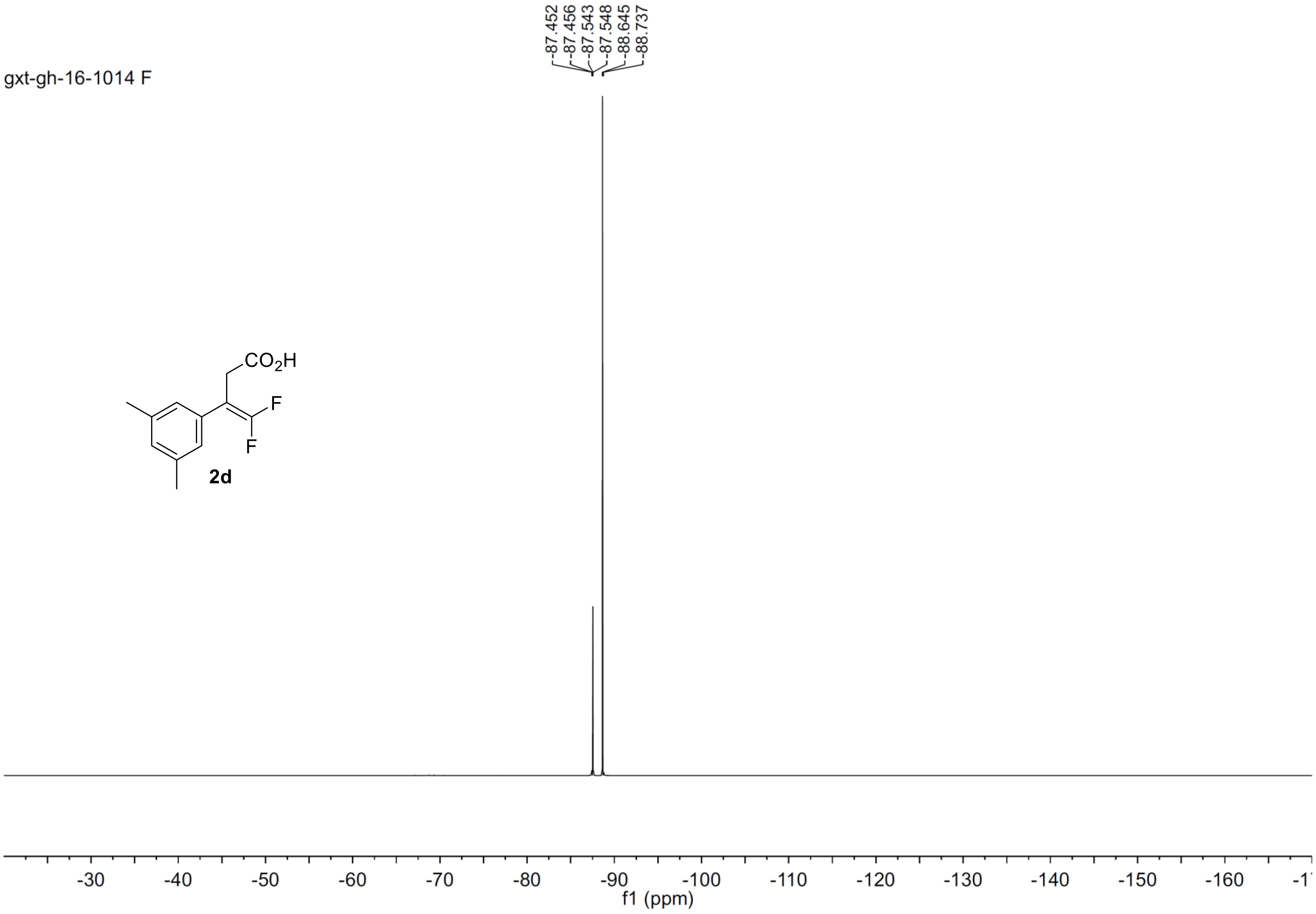
gxt-gh-16-1014 F
-87.452
-87.456
-87.543
-87.548
-88.645
-88.737
CO2H
F
F
2d
-30
-40
-50
-60
-70
-80
-90
-100
-110
-120
-130
-140
-150
-160
f1 (ppm)

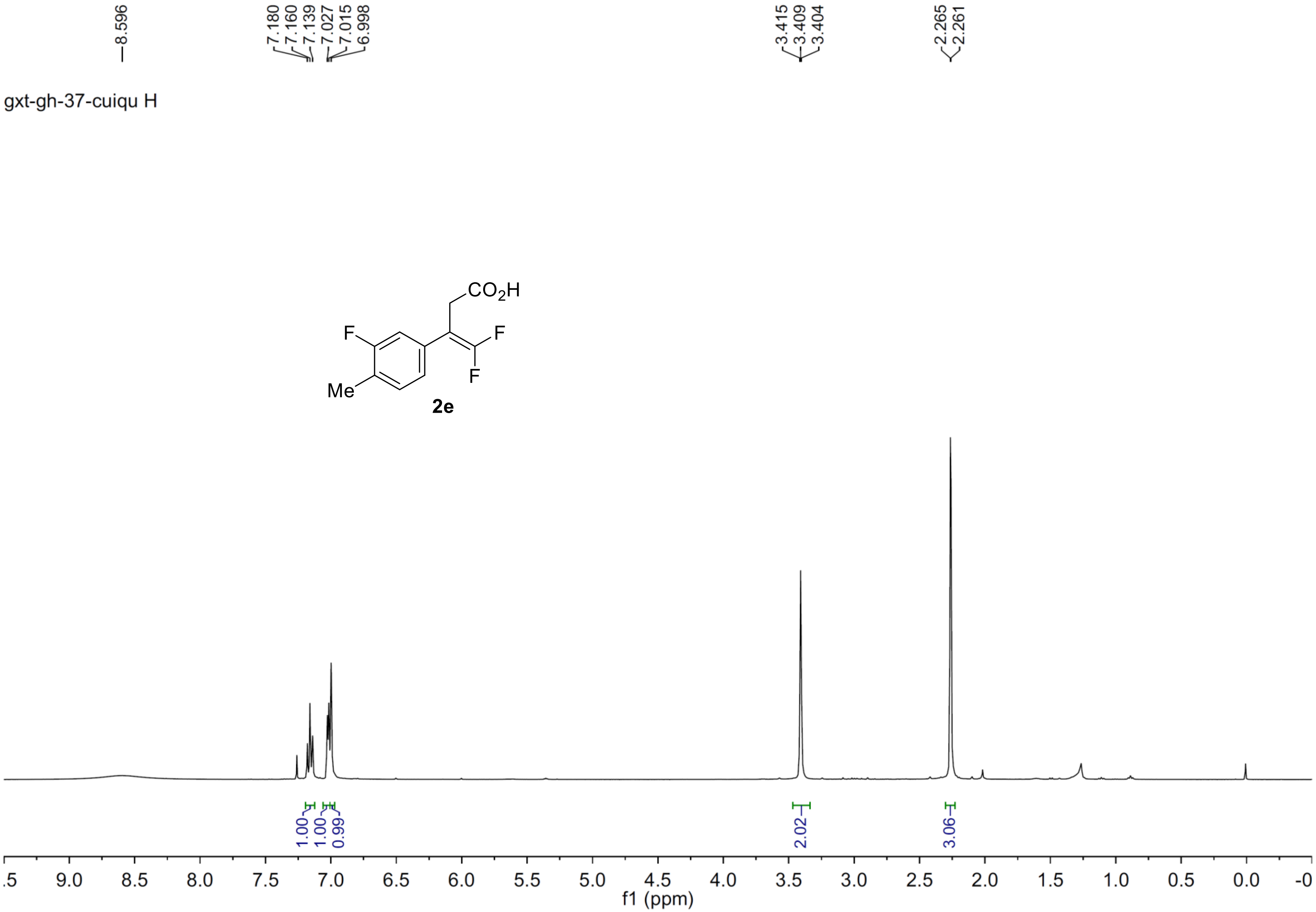
8.596
7.180
7.160
7.139
7.027
7.015
6.998
3.415
3.409
3.404
2.265
2.261
gxt-gh-37-cuiqu H
CO2H
F
F
F
Me
2e
1.00
1.00
0.99
2.02
3.06
f1 (ppm)

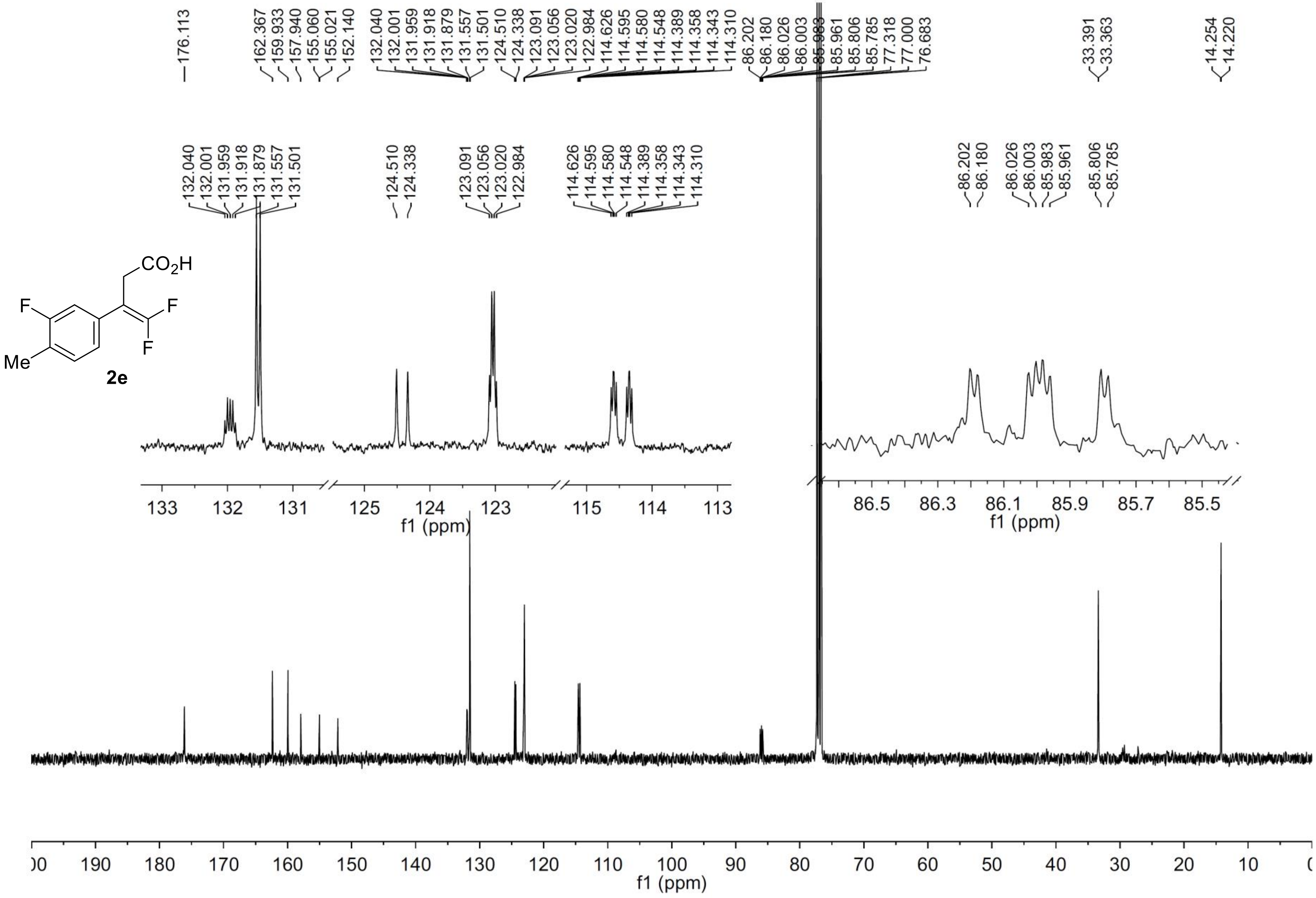
176.113
162.367
159.933
157.940
155.060
155.021
152.140
132.040
132.001
131.959
131.918
131.879
131.557
131.501
124.510
124.338
123.091
123.056
123.020
122.984
114.626
114.595
114.580
114.548
114.389
114.358
114.343
114.310
86.202
86.180
86.026
86.003
85.983
85.961
85.806
85.785
77.318
77.000
76.683
33.391
33.363
14.254
14.220
CO2H
F
F
F
Me
2e
f1 (ppm)
f1 (ppm)
f1 (ppm)

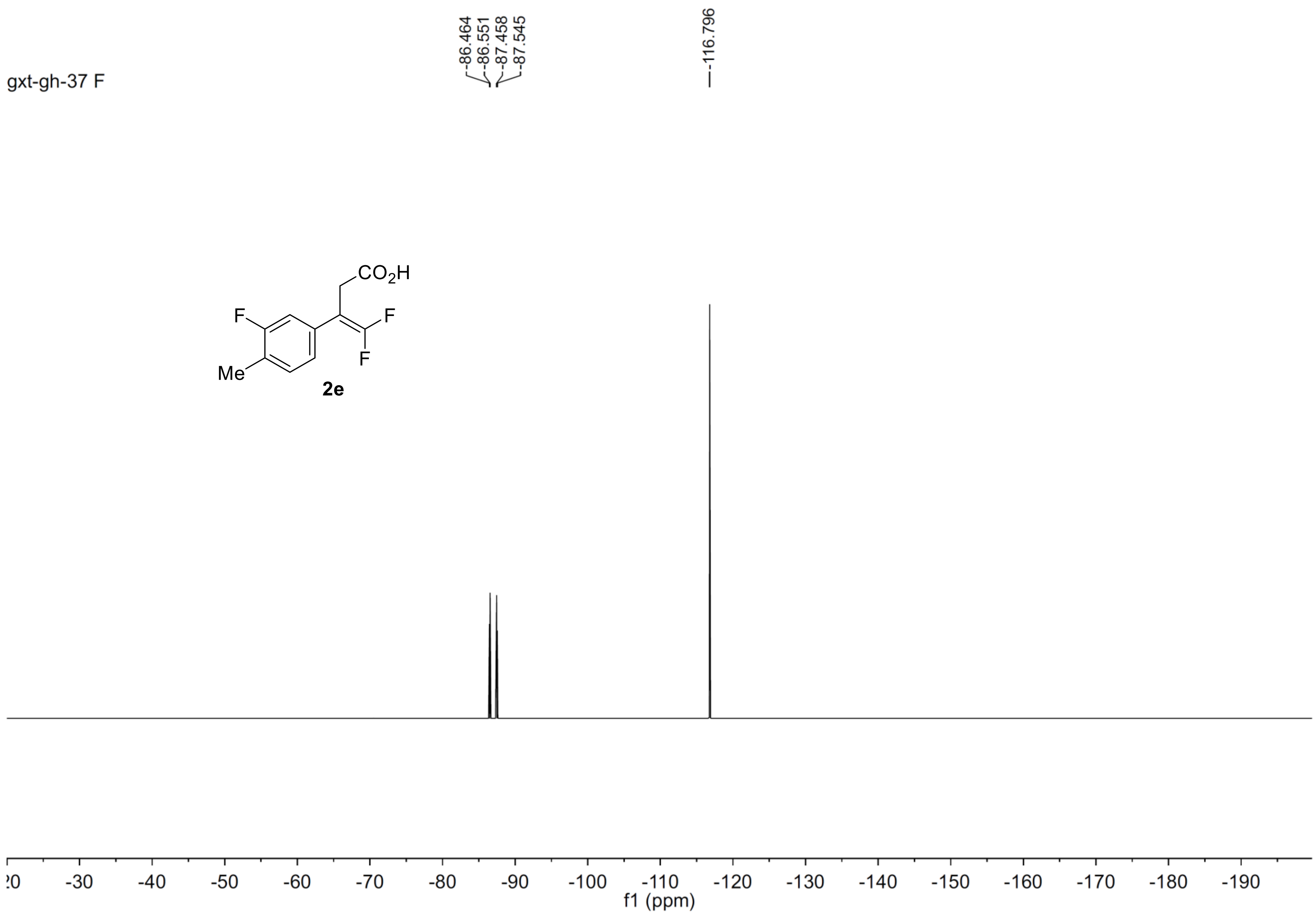
gxt-gh-37 F
-86.464
-86.551
-87.458
-87.545
-116.796
CO2H
F
F
F
Me
2e
-30
-40
-50
-60
-70
-80
-90
-100
-110
-120
-130
-140
-150
-160
-170
-180
-190
f1 (ppm)

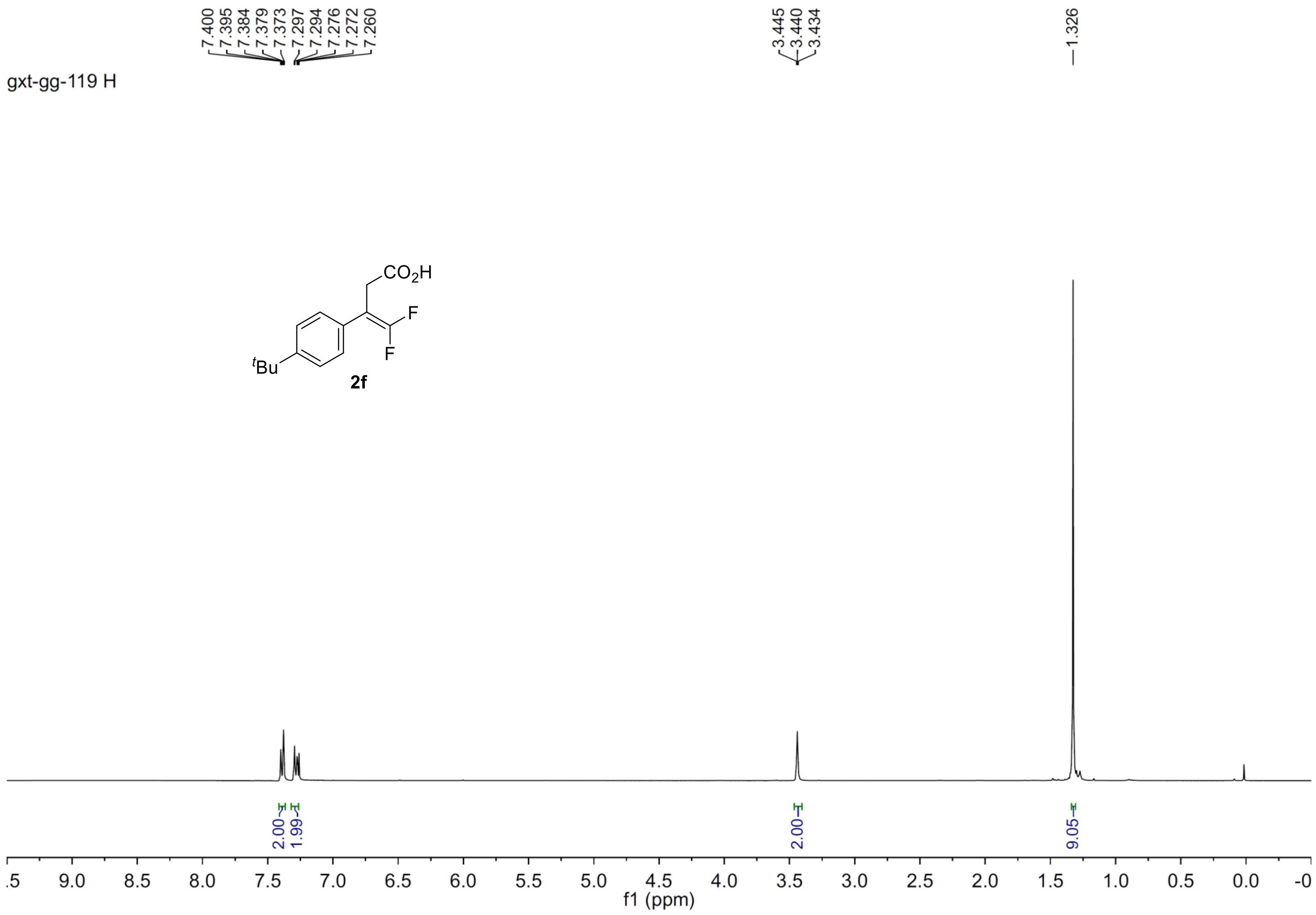

gxt-gg-119 H
7.400
7.395
7.384
7.379
7.373
7.297
7.294
7.276
7.272
7.260
3.445
3.440
3.434
1.326
CO2H
F
F
tBu
2f
2.00
1.99
2.00
9.05
f1 (ppm)

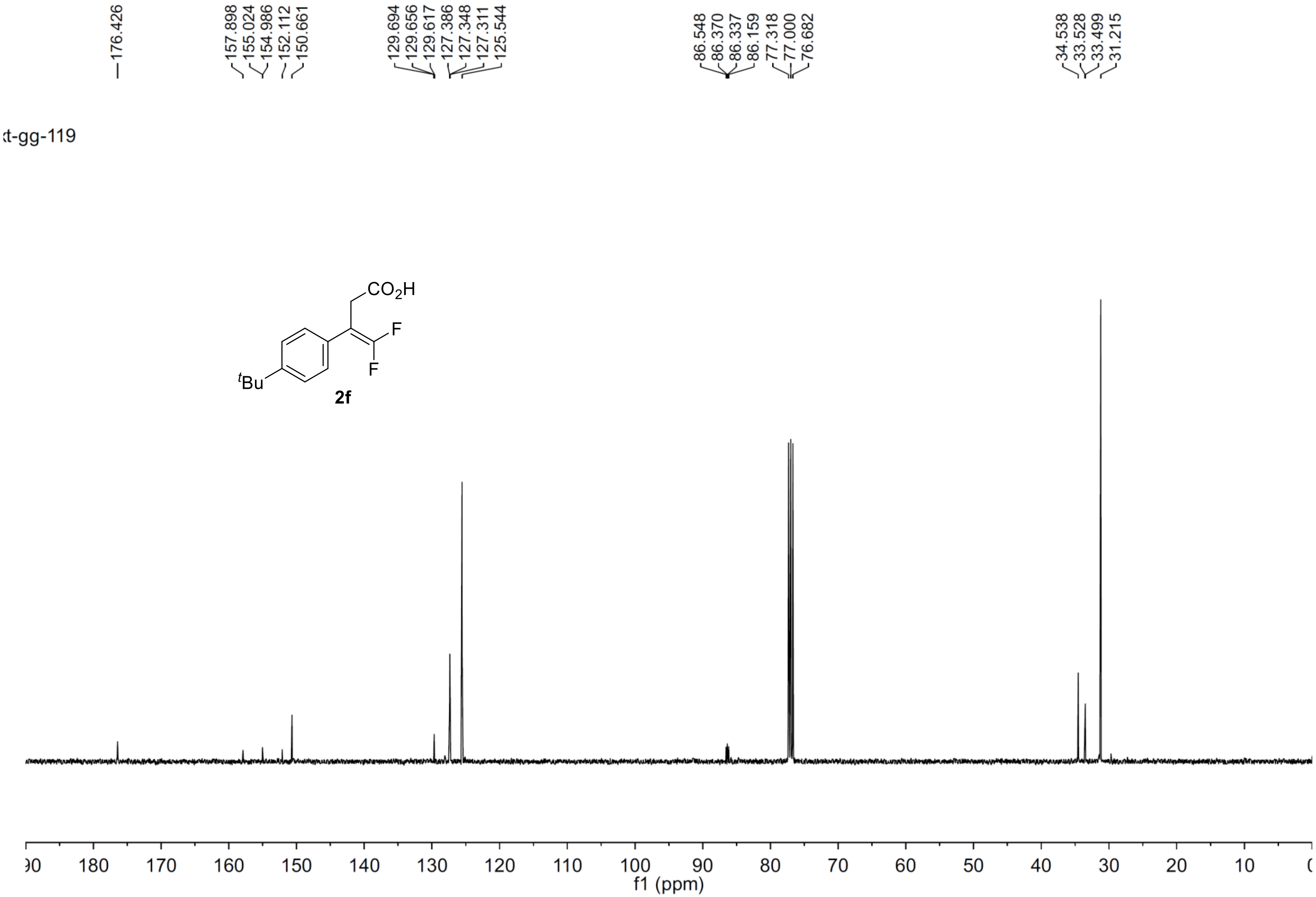
176.426
157.898
155.024
154.986
152.112
150.661
129.694
129.656
129.617
127.386
127.348
127.311
125.544
86.548
86.370
86.337
86.159
77.318
77.000
76.682
34.538
33.528
33.499
31.215
t-gg-119
CO2H
F
F
tBu
2f
f1 (ppm)

gxt-gg-119 F

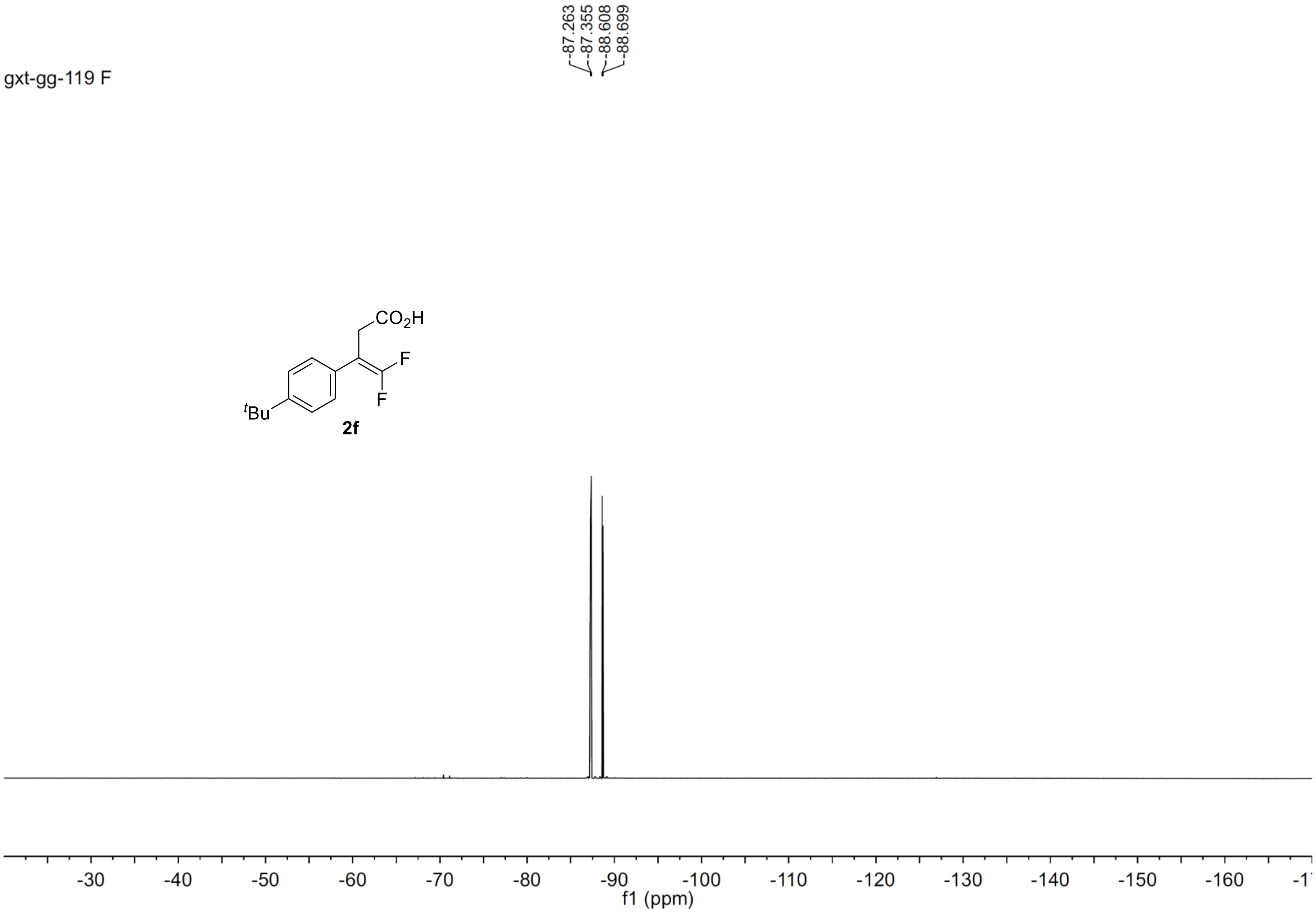
-87.263
-87.355
-88.608
-88.699
CO2H
F
F
tBu
2f
-30
-40
-50
-60
-70
-80
-90
-100
-110
-120
-130
-140
-150
-160
f1 (ppm)

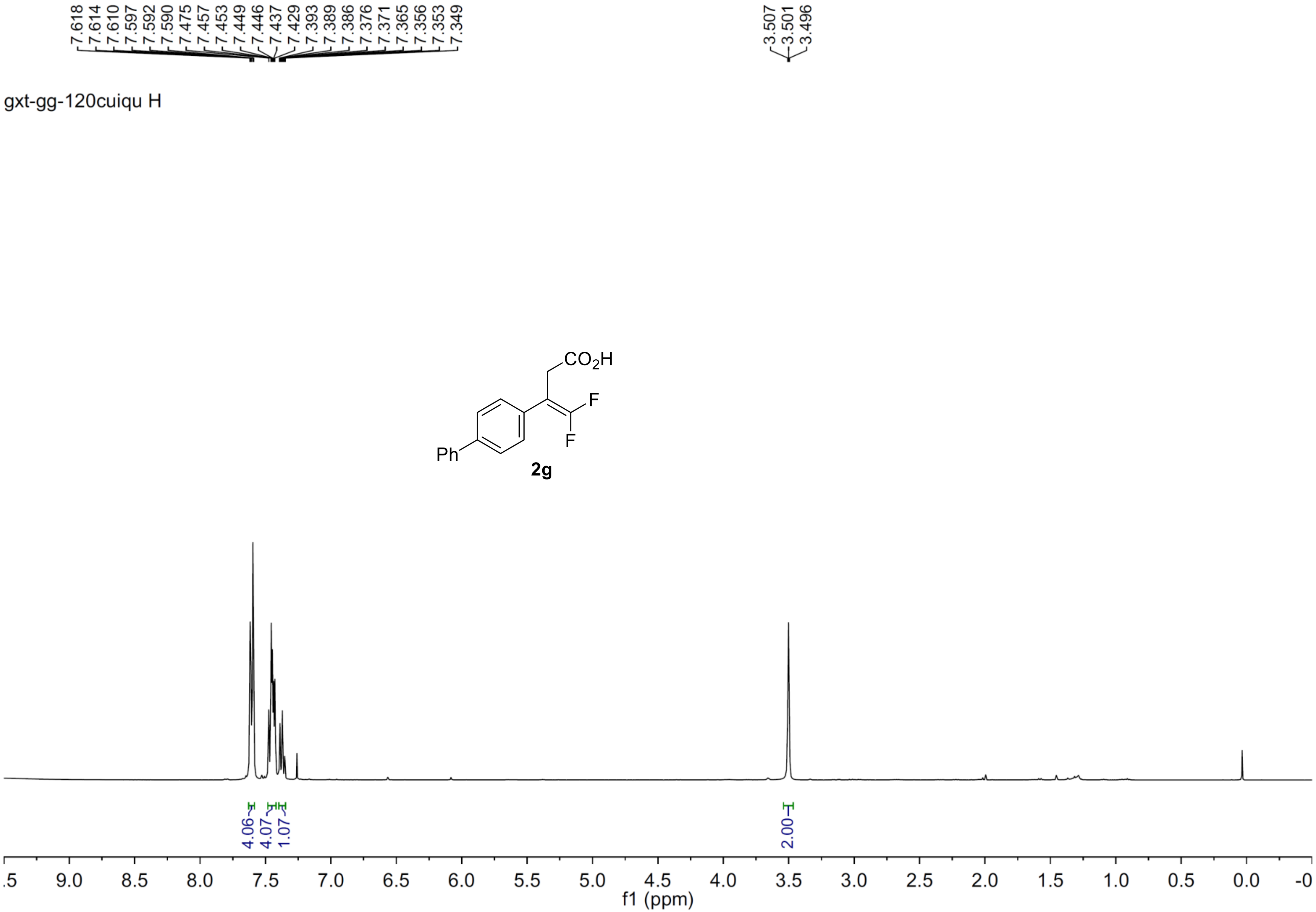
7.618
7.614
7.610
7.597
7.592
7.590
7.475
7.457
7.453
7.449
7.446
7.437
7.429
7.393
7.389
7.386
7.376
7.371
7.365
7.356
7.353
7.349
3.507
3.501
3.496
gxt-gg-120cuiqu H
CO2H
F
F
Ph
2g
4.06
4.07
1.07
2.00
9.0 8.5 8.0 7.5 7.0 6.5 6.0 5.5 5.0 4.5 4.0 3.5 3.0 2.5 2.0 1.5 1.0 0.5 0.0
f1 (ppm)

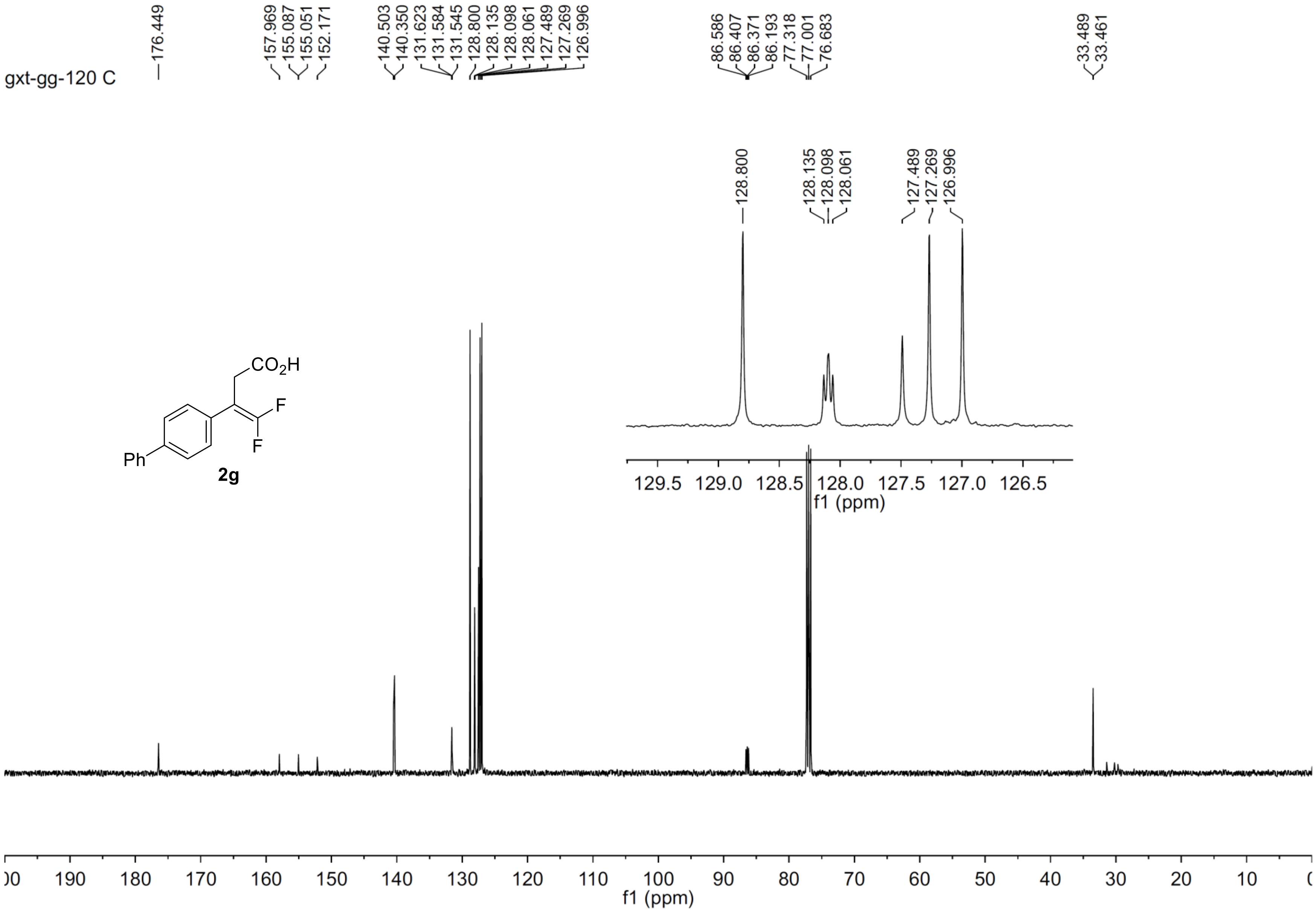
gxt-gg-120 C
176.449
157.969
155.087
155.051
152.171
140.503
140.350
131.623
131.584
131.545
128.800
128.135
128.098
128.061
127.489
127.269
126.996
86.586
86.407
86.371
86.193
77.318
77.001
76.683
33.489
33.461
CO2H
F
F
Ph
2g
129.5 129.0 128.5 128.0 127.5 127.0 126.5
f1 (ppm)
190 180 170 160 150 140 130 120 110 100 90 80 70 60 50 40 30 20 10
f1 (ppm)

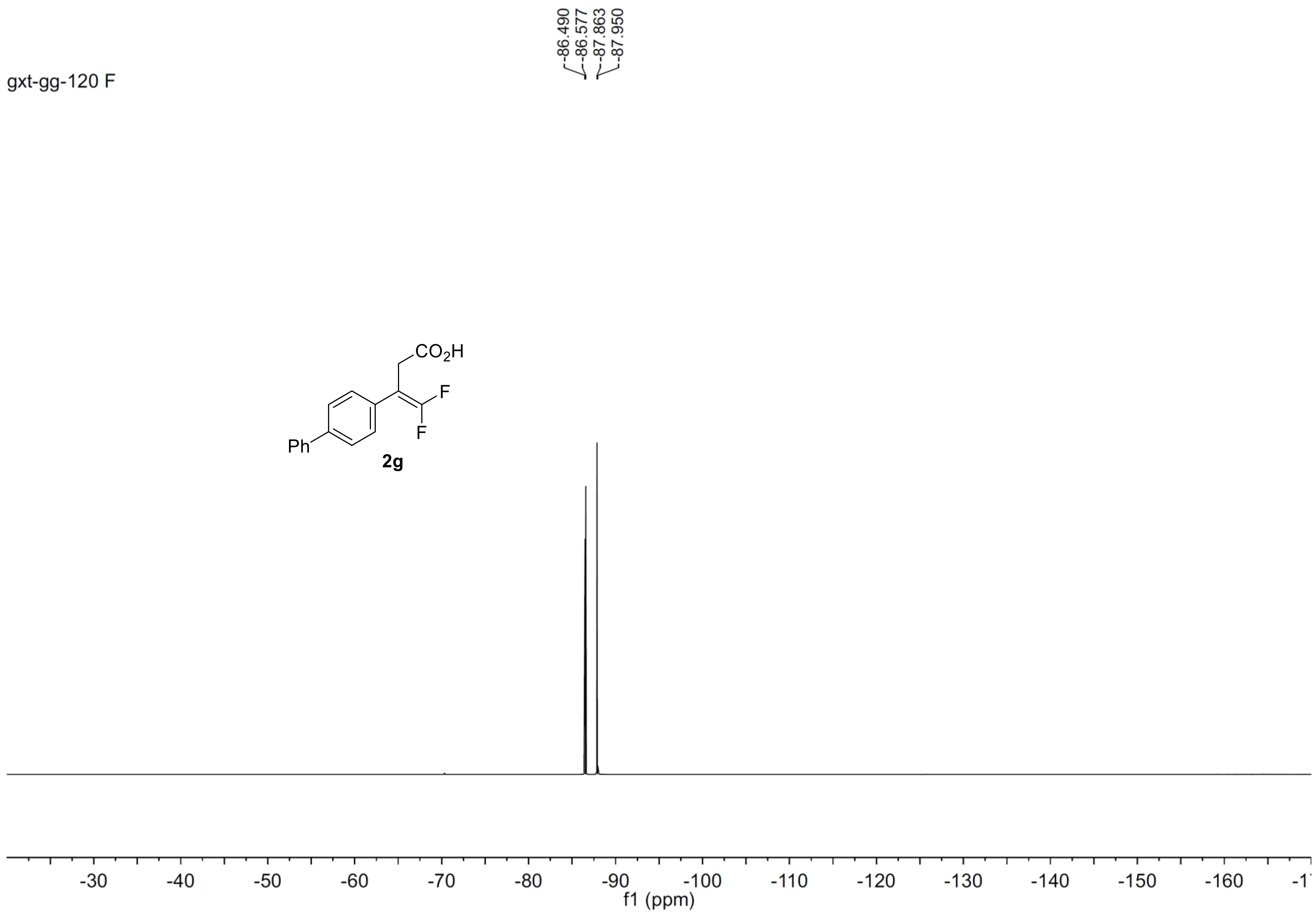
gxt-gg-120 F
-86.490
-86.577
-87.863
-87.950
CO2H
F
F
Ph
2g
-30
-40
-50
-60
-70
-80
-90
-100
-110
-120
-130
-140
-150
-160
f1 (ppm)

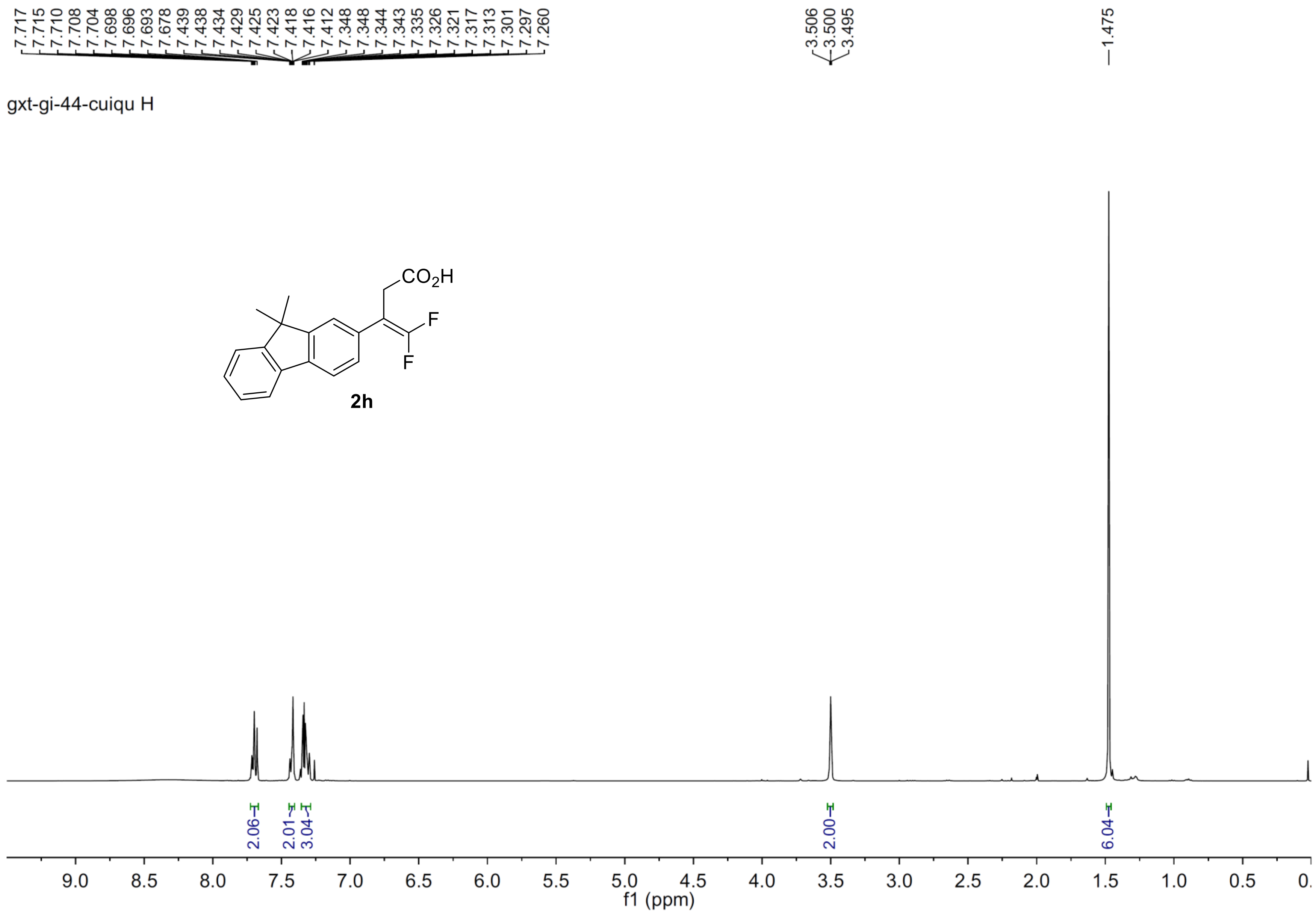
7.717
7.715
7.710
7.708
7.704
7.698
7.696
7.693
7.678
7.439
7.438
7.434
7.429
7.425
7.423
7.418
7.416
7.412
7.348
7.348
7.344
7.343
7.335
7.326
7.321
7.317
7.313
7.301
7.297
7.260
3.506
3.500
3.495
1.475
gxt-gi-44-cuiqu H
CO2H
F
F
2h
2.06
2.01
3.04
2.00
6.04
9.0
8.5
8.0
7.5
7.0
6.5
6.0
5.5
5.0
4.5
4.0
3.5
3.0
2.5
2.0
1.5
1.0
0.5
f1 (ppm)

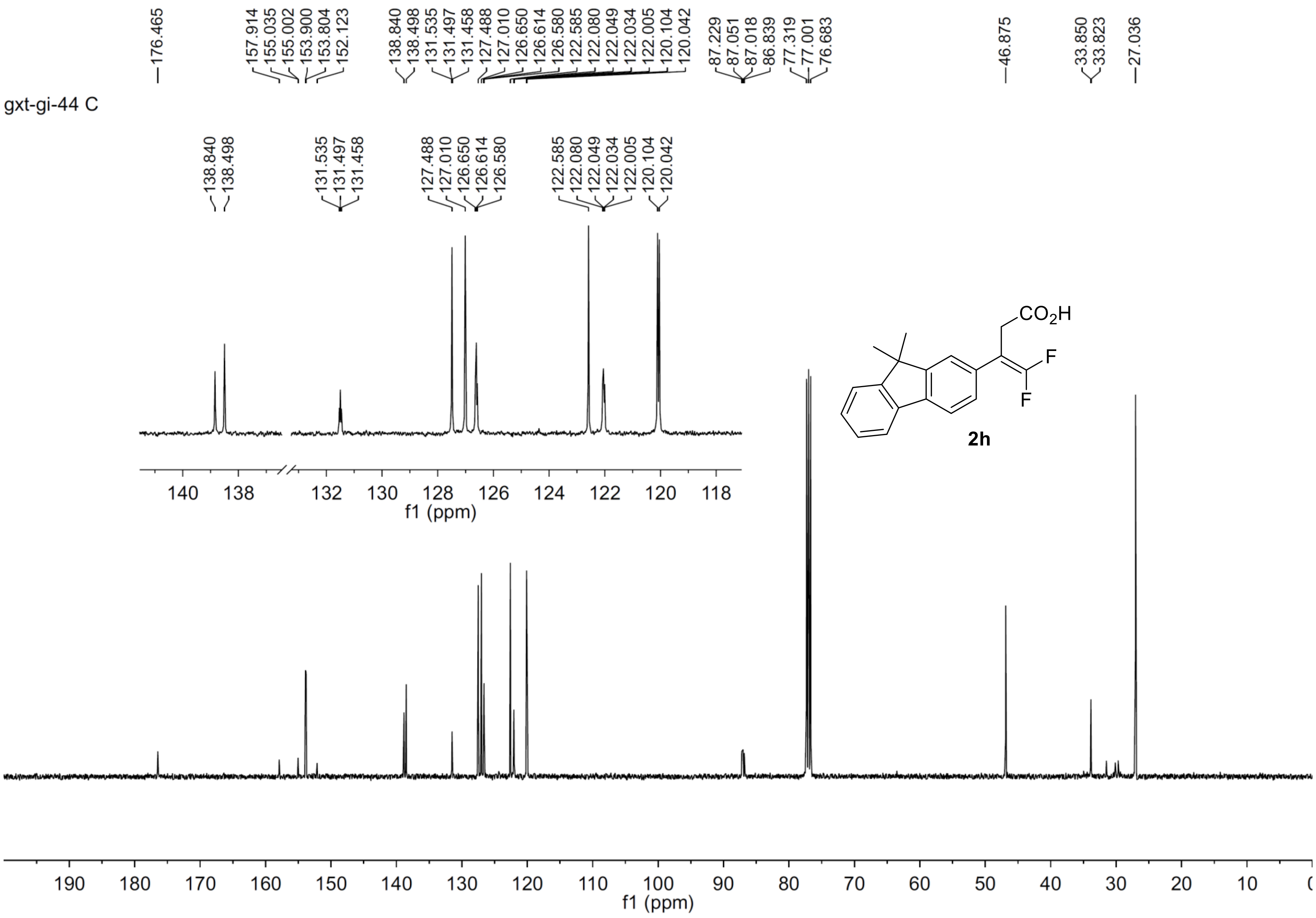
gxt-gi-44 C
176.465
157.914
155.035
155.002
153.900
153.804
152.123
138.840
138.498
131.535
131.497
131.458
127.488
127.010
126.650
126.614
126.580
122.585
122.080
122.049
122.034
122.005
120.104
120.042
87.229
87.051
87.018
86.839
77.319
77.001
76.683
46.875
33.850
33.823
27.036
f1 (ppm)
CO_2H
F
F
2h

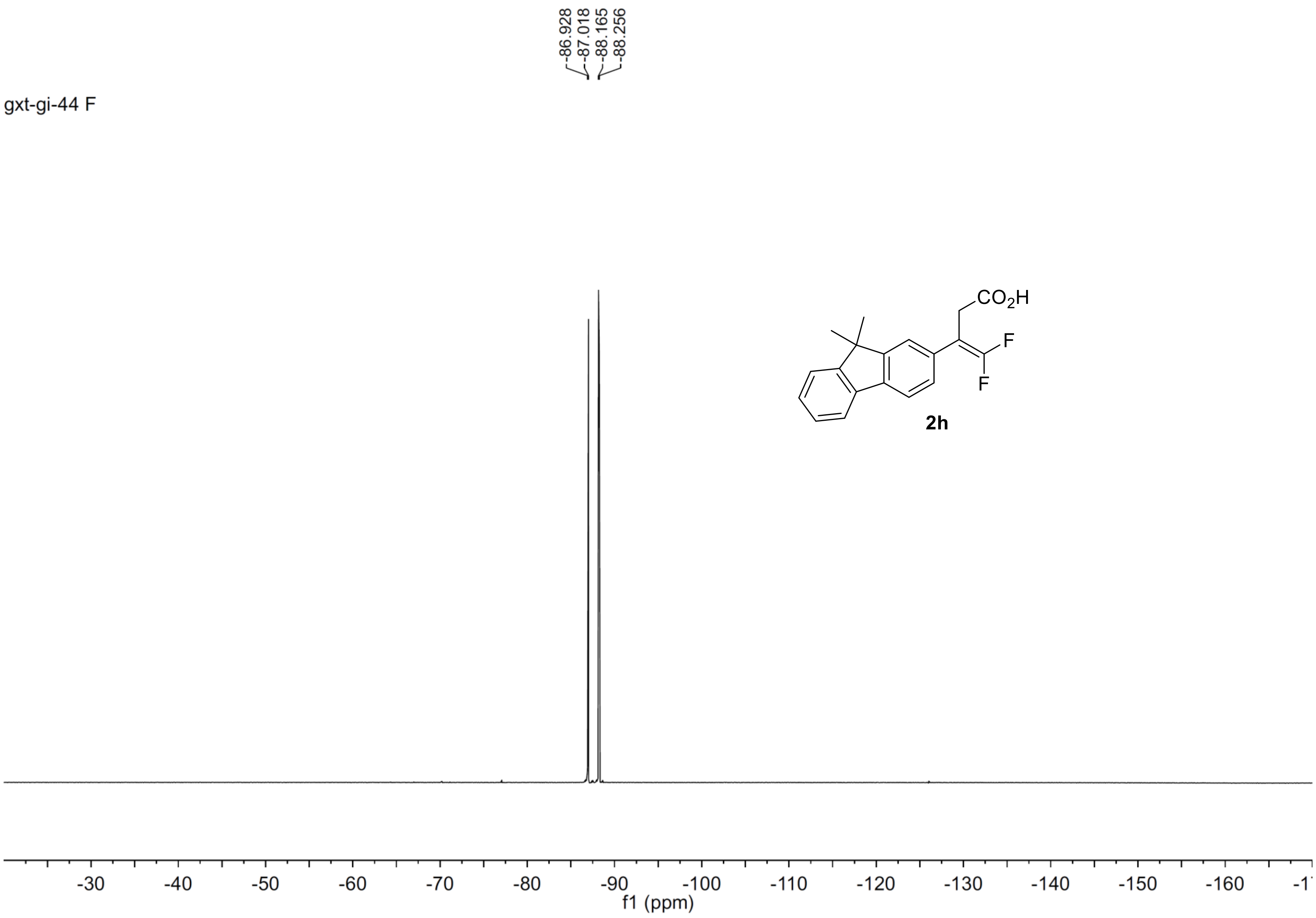
-86.928
-87.018
-88.165
-88.256
gxt-gi-44 F
CO2H
F
F
2h
-30 -40 -50 -60 -70 -80 -90 -100 -110 -120 -130 -140 -150 -160
f1 (ppm)

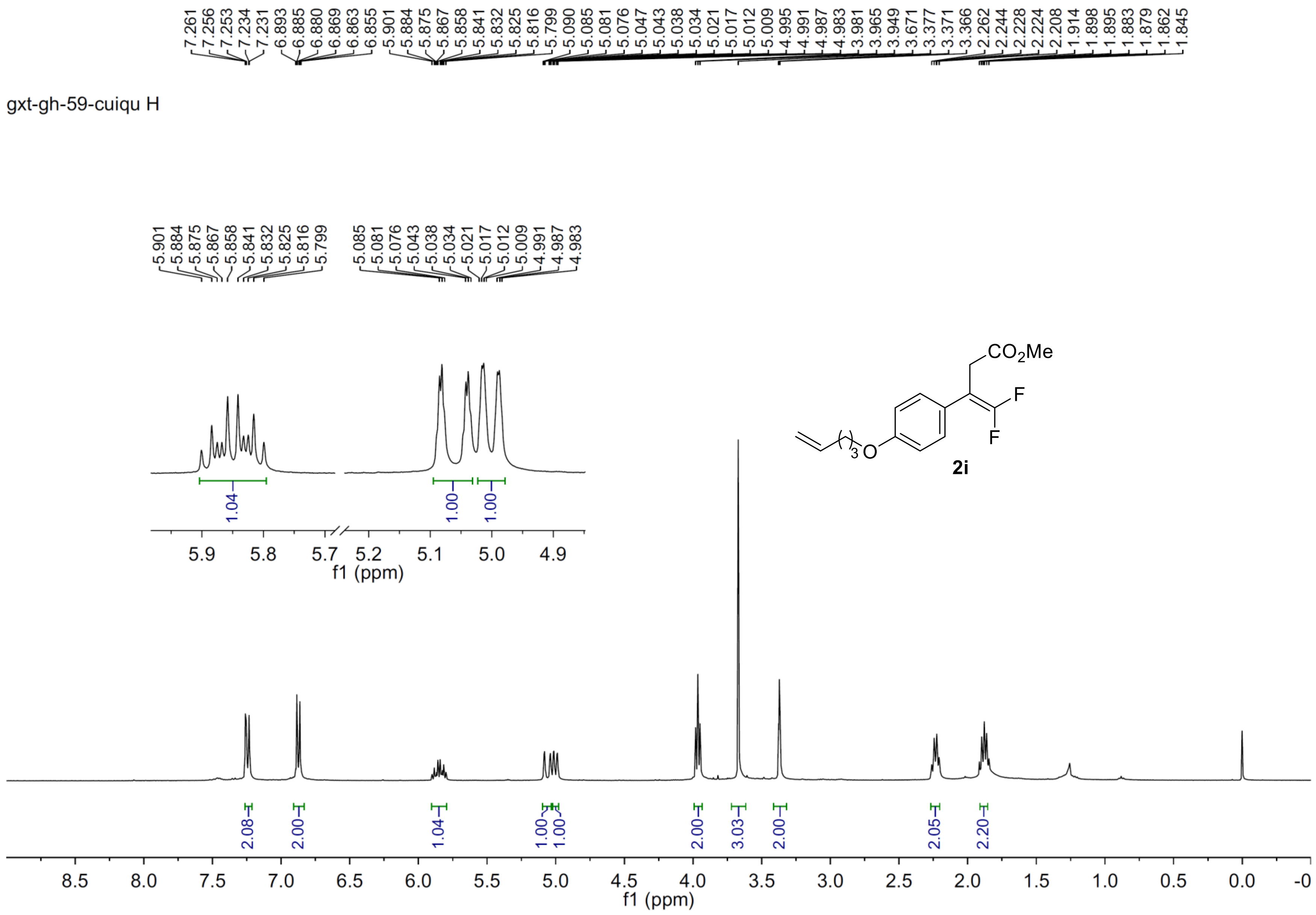

gxt-gh-59-cuiqu H
7.261 7.256 7.253 7.234 7.231 6.893 6.885 6.880 6.869 6.863 6.855 5.901 5.884 5.875 5.867 5.858 5.841 5.832 5.825 5.816 5.799 5.090 5.085 5.081 5.076 5.047 5.043 5.038 5.034 5.021 5.017 5.012 5.009 4.995 4.991 4.987 4.983 3.981 3.965 3.949 3.671 3.377 3.371 3.366 2.262 2.244 2.228 2.224 2.208 1.914 1.898 1.895 1.883 1.879 1.862 1.845
5.901 5.884 5.875 5.867 5.858 5.841 5.832 5.825 5.816 5.799
5.085 5.081 5.076 5.043 5.038 5.034 5.021 5.017 5.012 5.009 4.991 4.987 4.983
1.04 1.00 1.00
5.9 5.8 5.7 5.2 5.1 5.0 4.9
f1 (ppm)
CO2Me
F
F
O
3
2i
2.08 2.00 1.04 1.00 1.00 2.00 3.03 2.00 2.05 2.20
8.5 8.0 7.5 7.0 6.5 6.0 5.5 5.0 4.5 4.0 3.5 3.0 2.5 2.0 1.5 1.0 0.5 0.0 -0
f1 (ppm)

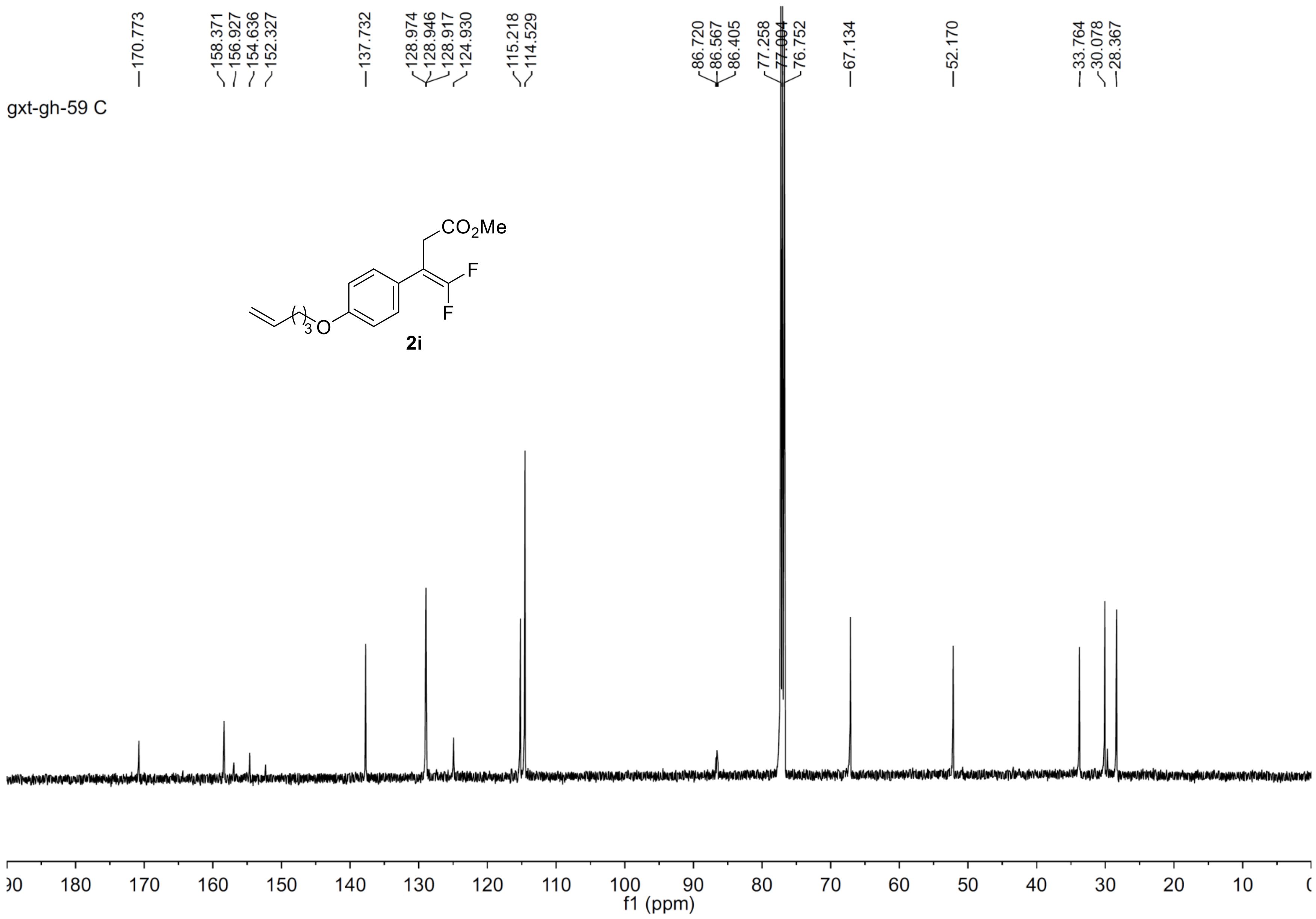
gxt-gh-59 C
170.773
158.371
156.927
154.636
152.327
137.732
128.974
128.946
128.917
124.930
115.218
114.529
86.720
86.567
86.405
77.258
77.004
76.752
67.134
52.170
33.764
30.078
28.367
CO2Me
F
F
3
O
2i
f1 (ppm)

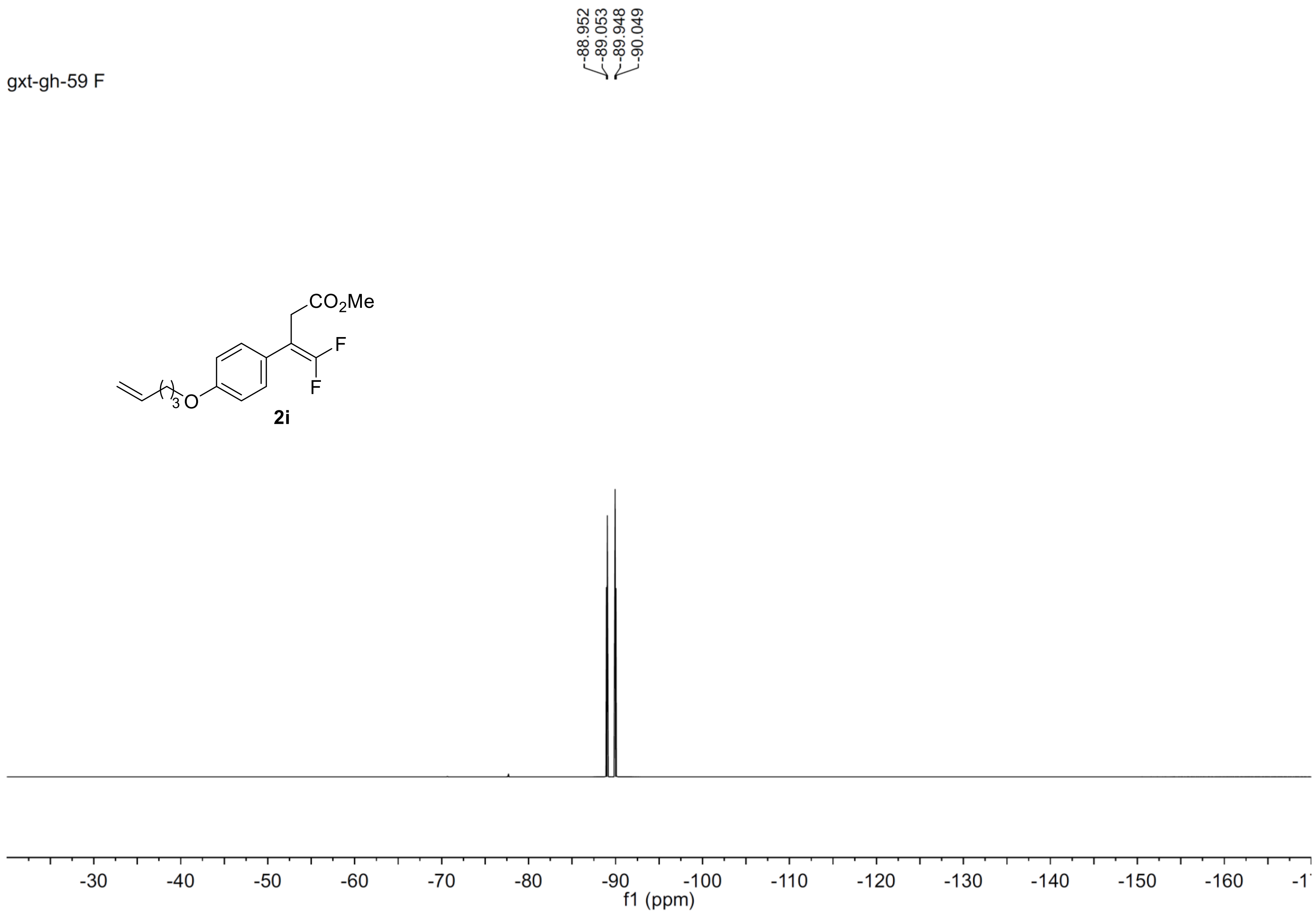
gxt-gh-59 F
-88.952
-89.053
-89.948
-90.049
CO2Me
F
F
3
O
2i
-30
-40
-50
-60
-70
-80
-90
-100
-110
-120
-130
-140
-150
-160
f1 (ppm)

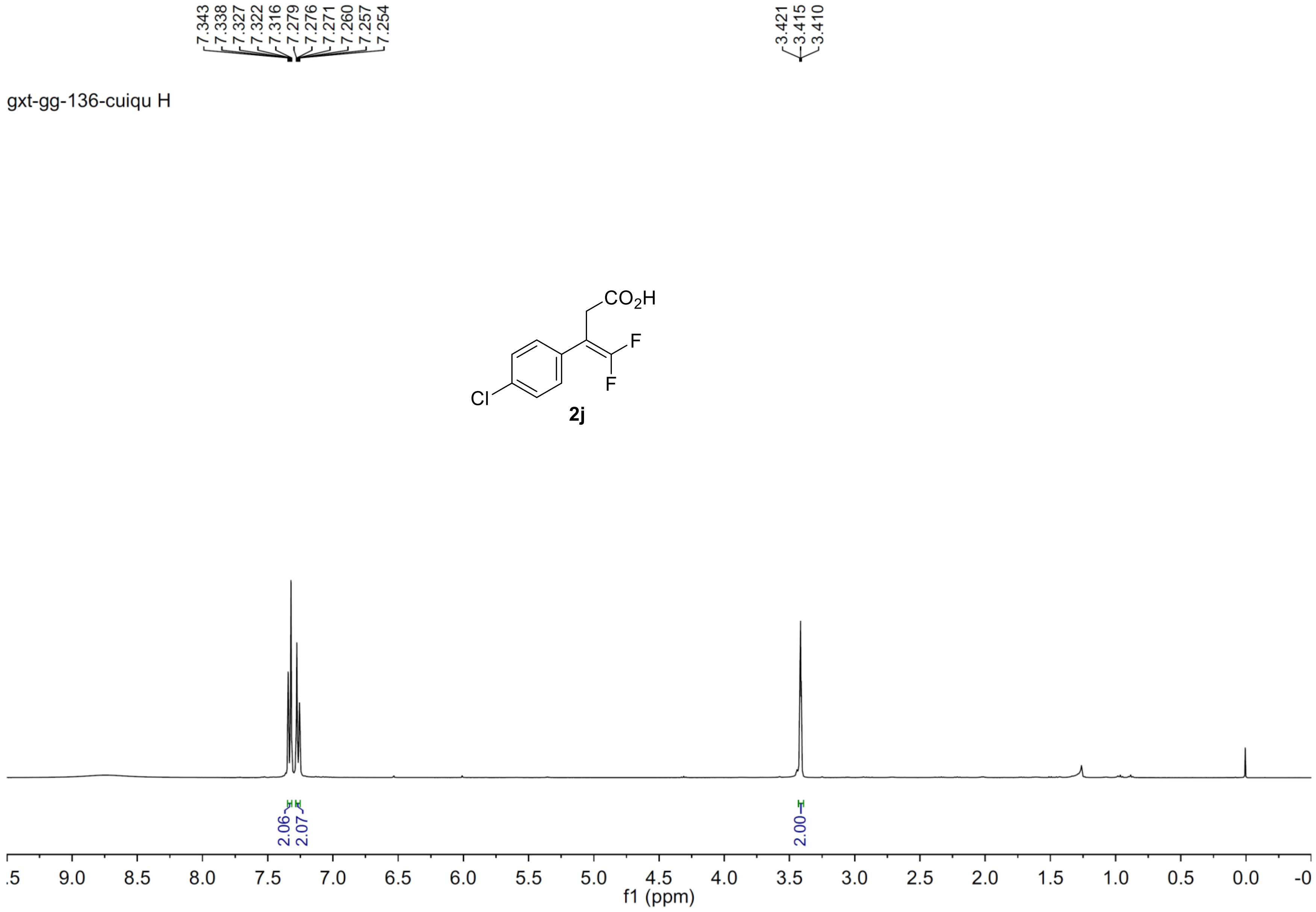
7.343
7.338
7.327
7.322
7.316
7.279
7.276
7.271
7.260
7.257
7.254
3.421
3.415
3.410
gxt-gg-136-cuiqu H
CO2H
F
F
Cl
2j
2.06
2.07
2.00
.5 9.0 8.5 8.0 7.5 7.0 6.5 6.0 5.5 5.0 4.5 4.0 3.5 3.0 2.5 2.0 1.5 1.0 0.5 0.0 -0
f1 (ppm)

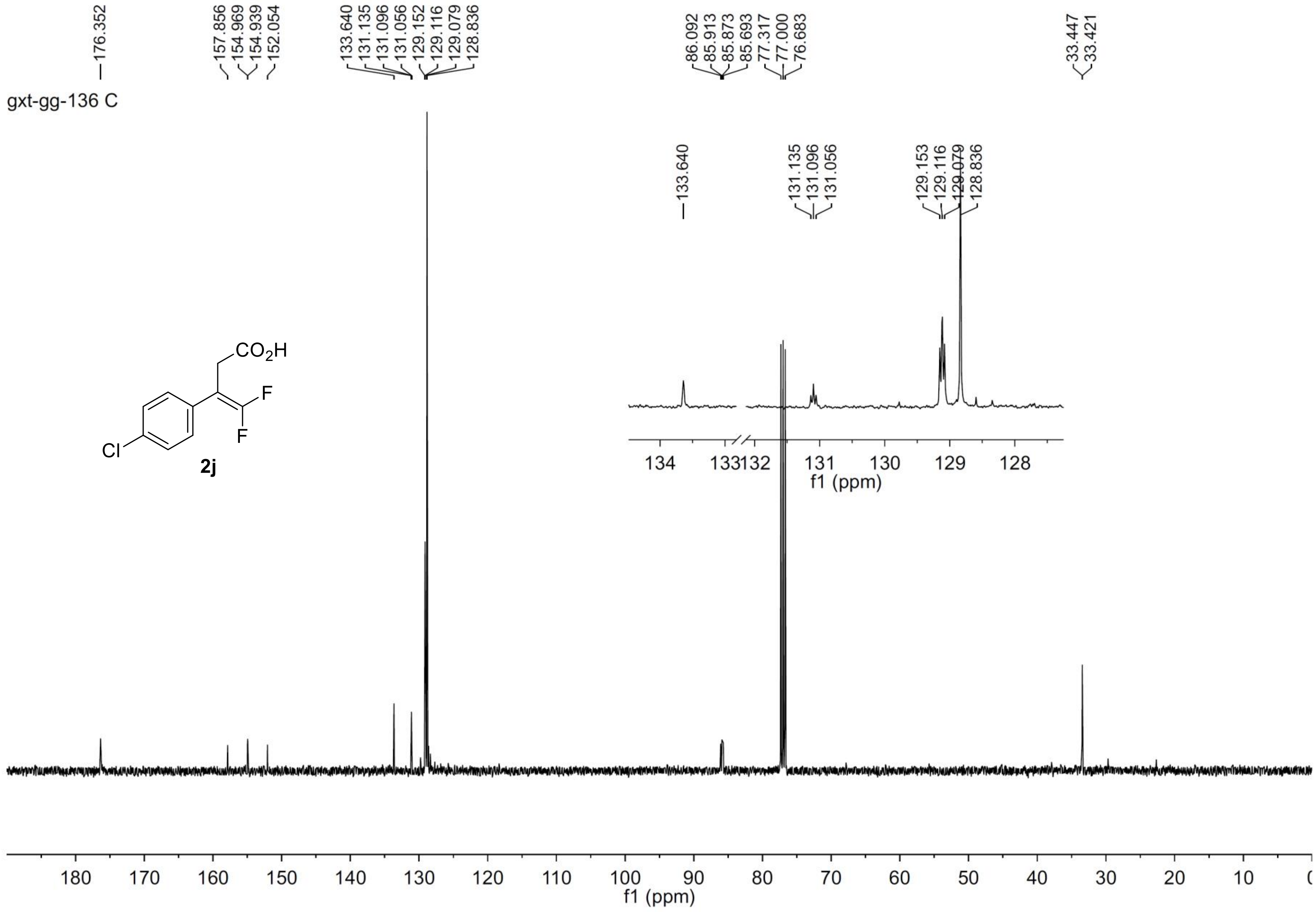
gxt-gg-136 C
176.352
157.856
154.969
154.939
152.054
133.640
131.135
131.096
131.056
129.152
129.116
129.079
128.836
86.092
85.913
85.873
85.693
77.317
77.000
76.683
33.447
33.421
133.640
131.135
131.096
131.056
129.153
129.116
129.079
128.836
134
133132
131
130
129
128
f1 (ppm)
CO_2H
F
F
Cl
2j
180
170
160
150
140
130
120
110
100
90
80
70
60
50
40
30
20
10
f1 (ppm)

gxt-gg-136 F

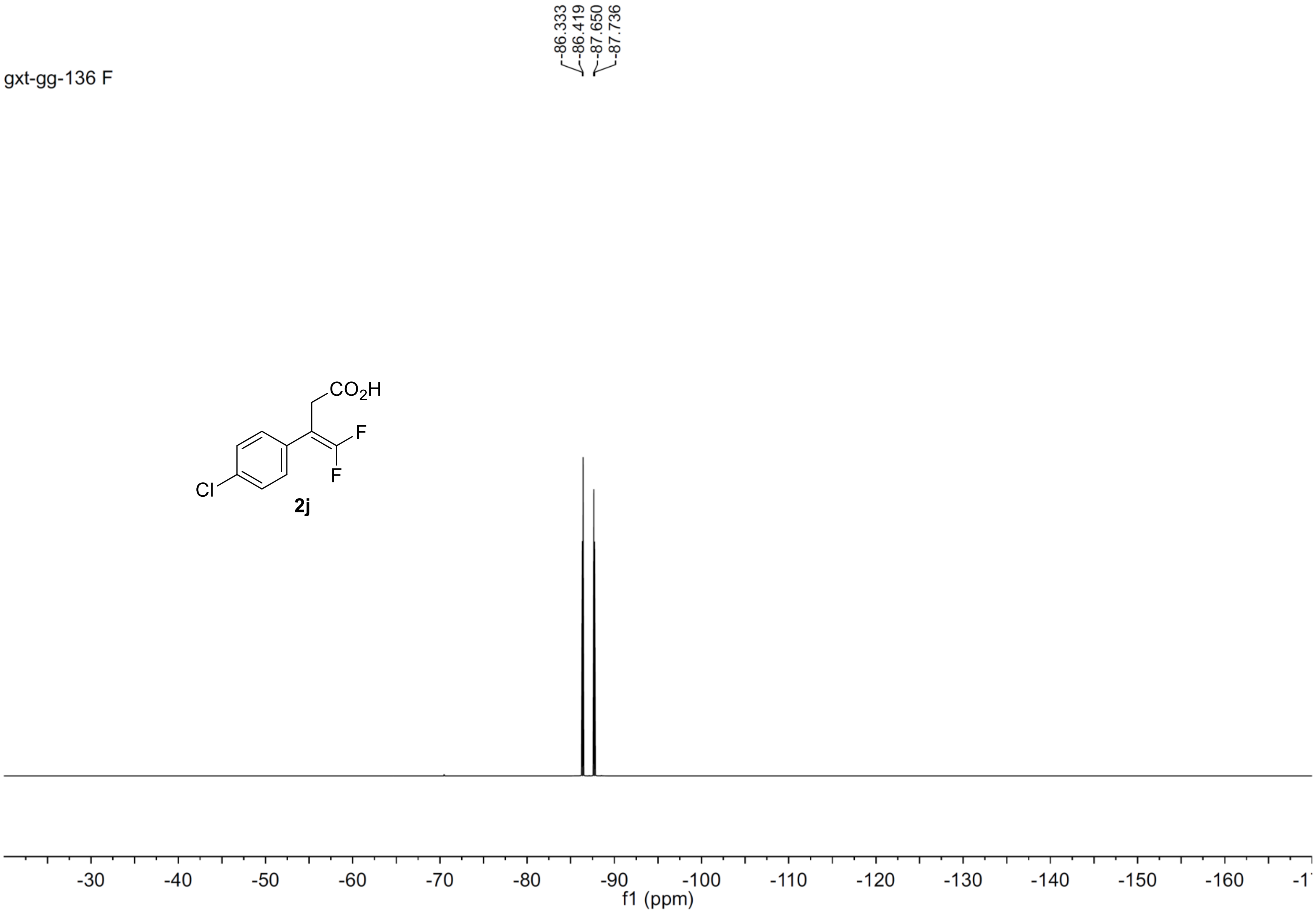
-86.333
-86.419
-87.650
-87.736
CO2H
F
F
Cl
2j
-30
-40
-50
-60
-70
-80
-90
-100
-110
-120
-130
-140
-150
-160
f1 (ppm)

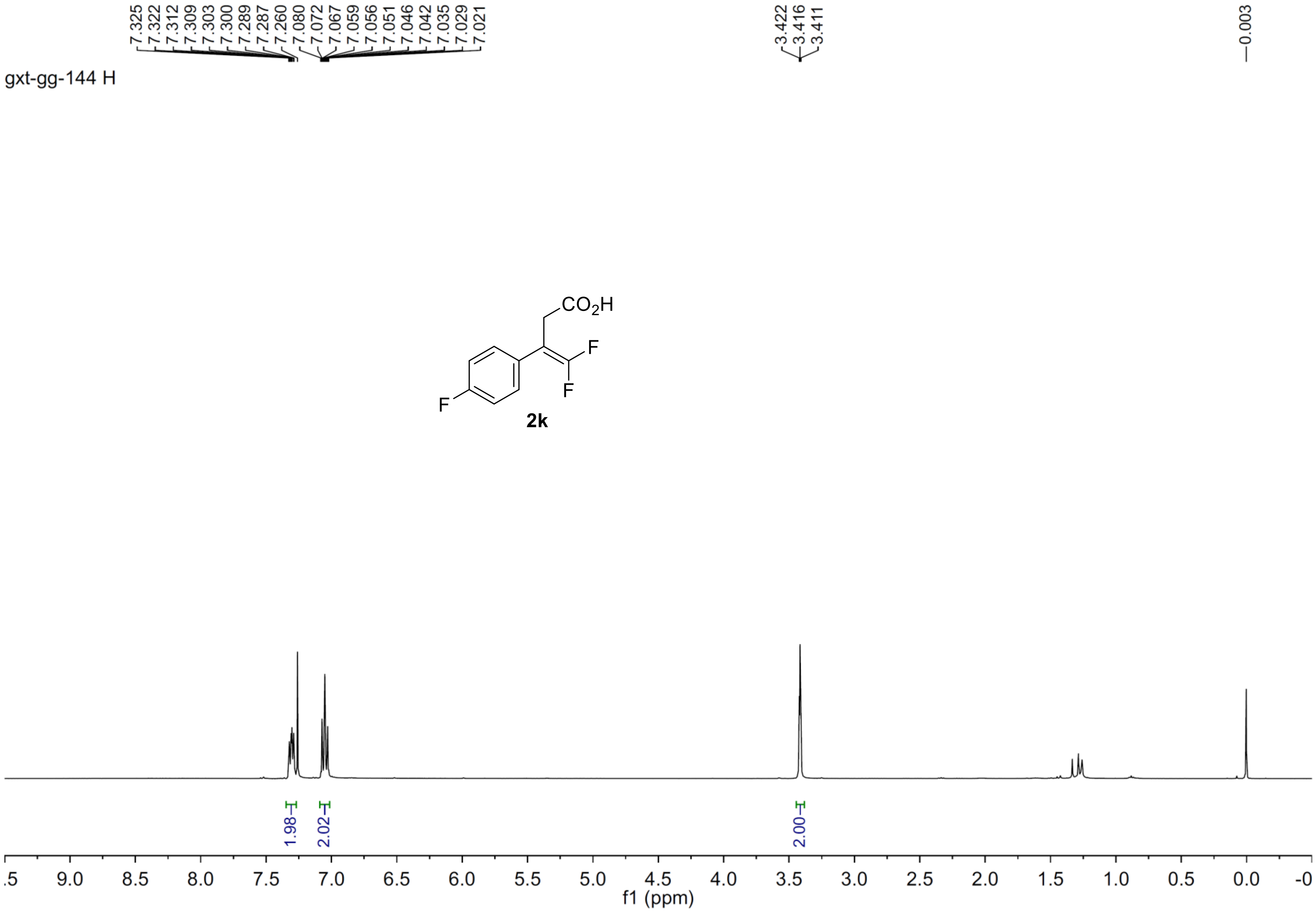
gxt-gg-144 H
7.325
7.322
7.312
7.309
7.303
7.300
7.289
7.287
7.260
7.080
7.072
7.067
7.059
7.056
7.051
7.046
7.042
7.035
7.029
7.021
3.422
3.416
3.411
0.003
CO2H
F
F
F
2k
1.98
2.02
2.00
.5
9.0
8.5
8.0
7.5
7.0
6.5
6.0
5.5
5.0
4.5
4.0
3.5
3.0
2.5
2.0
1.5
1.0
0.5
0.0
-0
f1 (ppm)

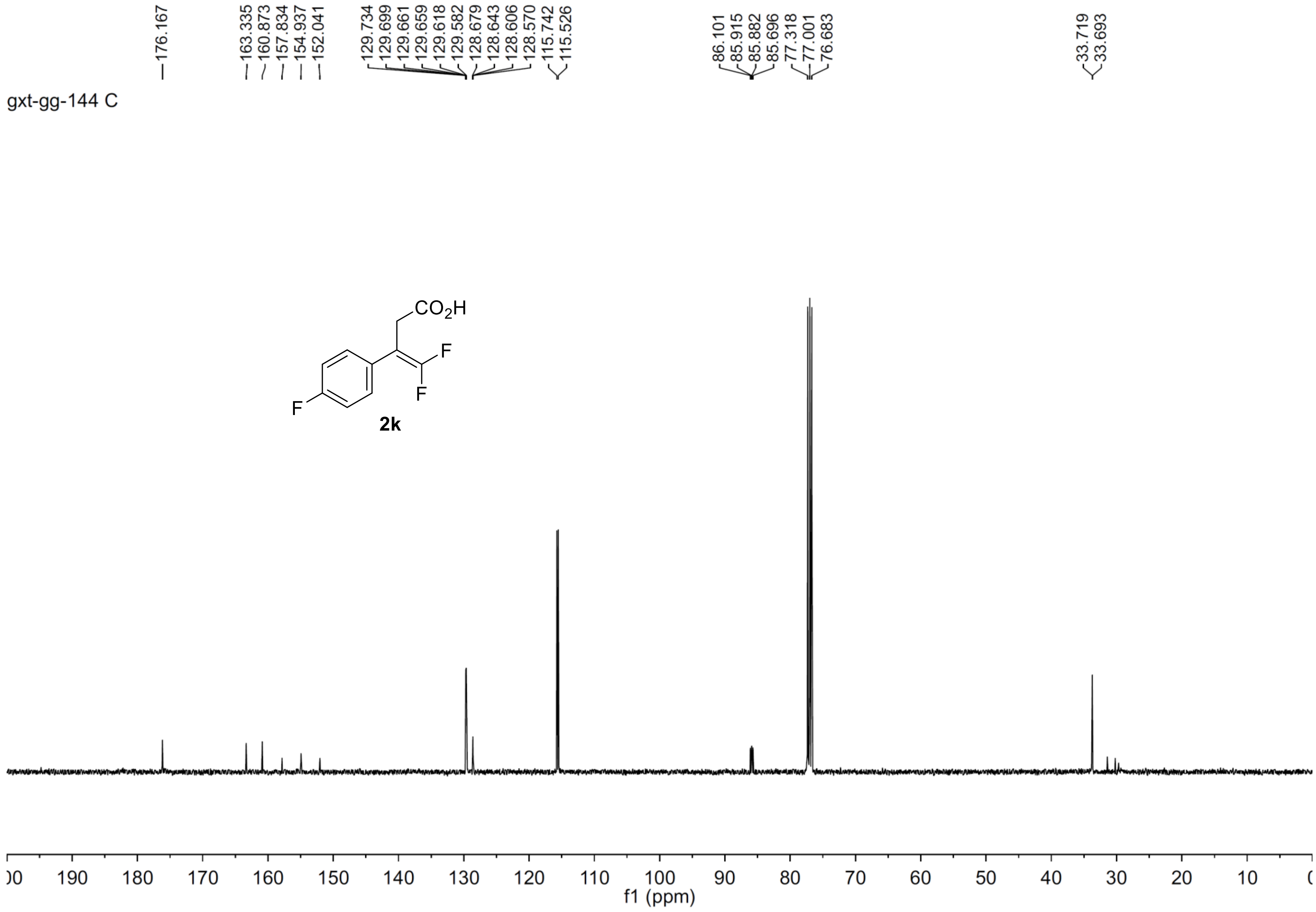
gxt-gg-144 C
176.167
163.335
160.873
157.834
154.937
152.041
129.734
129.699
129.661
129.659
129.618
129.582
128.679
128.643
128.606
128.570
115.742
115.526
86.101
85.915
85.882
85.696
77.318
77.001
76.683
33.719
33.693
CO2H
F
F
F
2k
190
180
170
160
150
140
130
120
110
100
90
80
70
60
50
40
30
20
10
f1 (ppm)

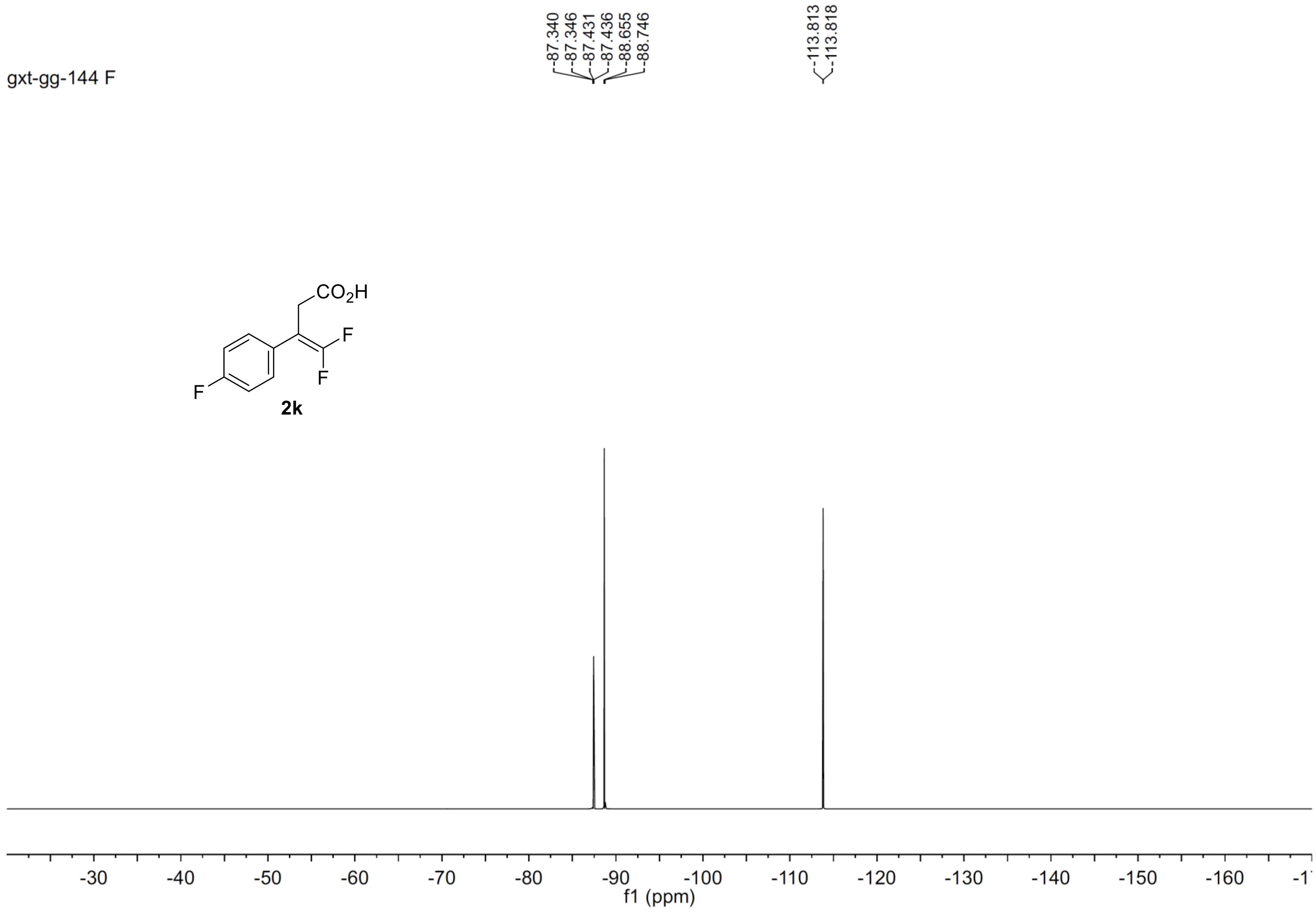
gxt-gg-144 F
-87.340
-87.346
-87.431
-87.436
-88.655
-88.746
-113.813
-113.818
CO2H
F
F
F
2k
-30 -40 -50 -60 -70 -80 -90 -100 -110 -120 -130 -140 -150 -160
f1 (ppm)

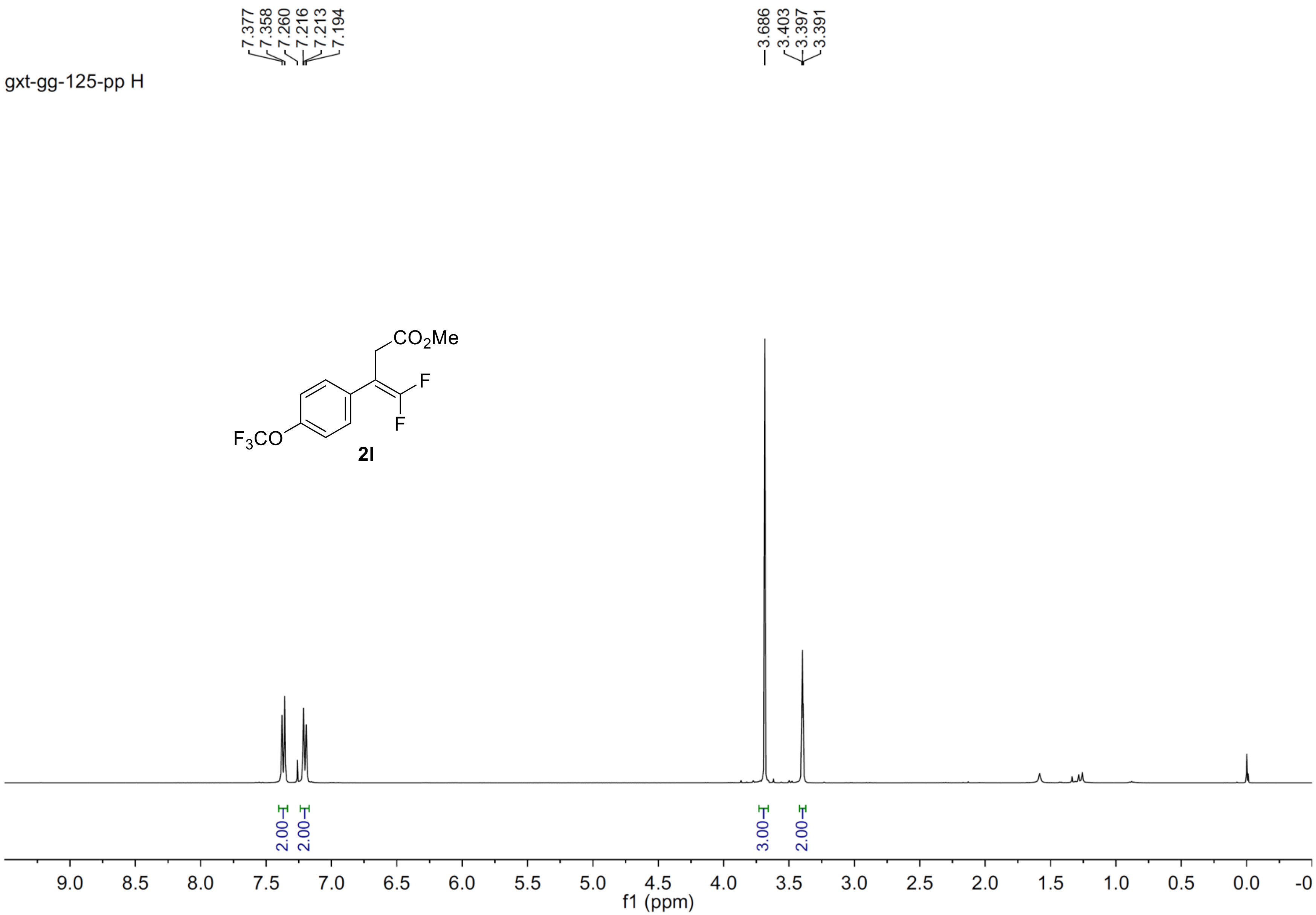
gxt-gg-125-pp H
7.377
7.358
7.260
7.216
7.213
7.194
3.686
3.403
3.397
3.391
CO2Me
F
F
F3CO
2l
2.00
2.00
3.00
2.00
9.0 8.5 8.0 7.5 7.0 6.5 6.0 5.5 5.0 4.5 4.0 3.5 3.0 2.5 2.0 1.5 1.0 0.5 0.0 -0
f1 (ppm)

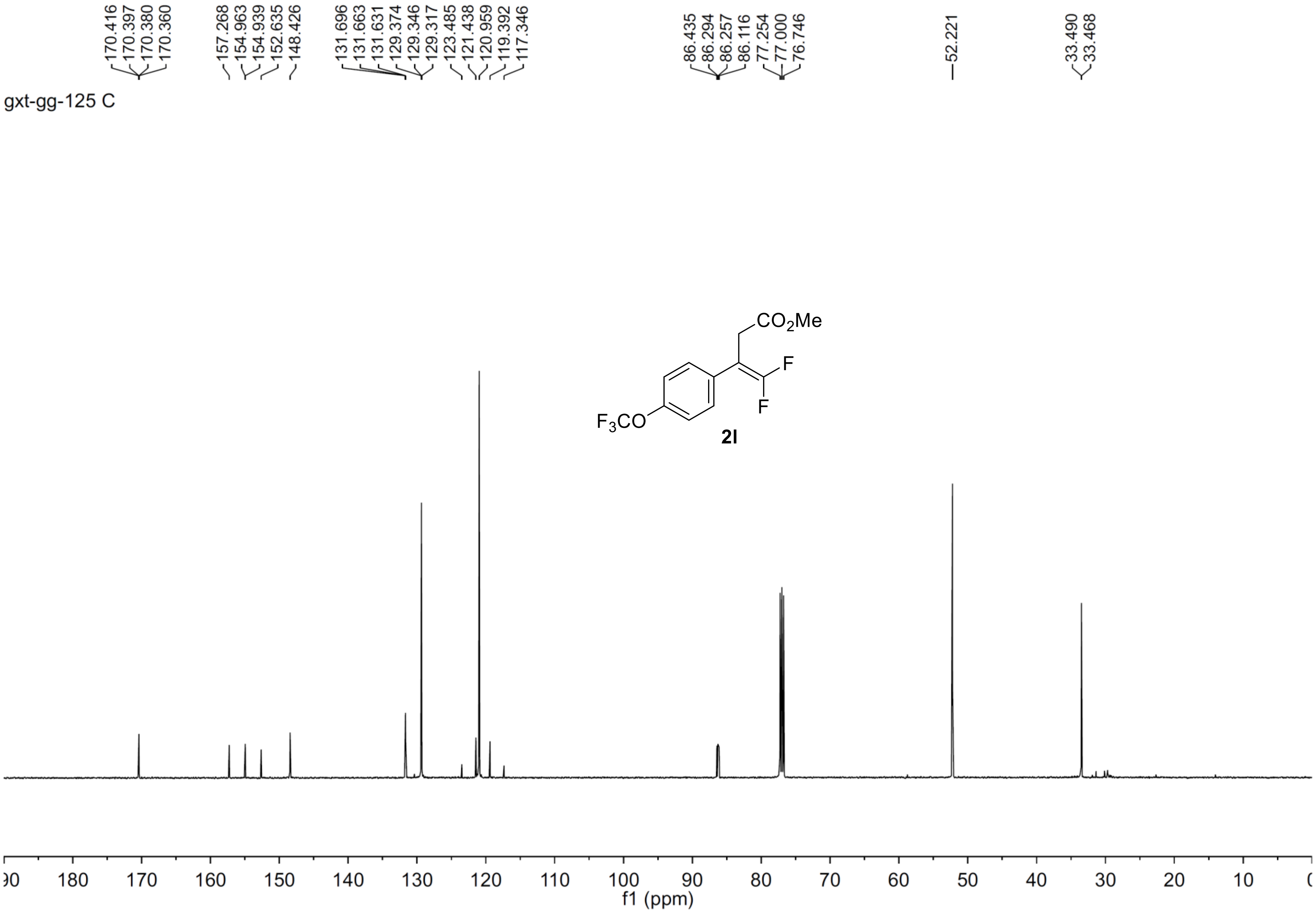
170.416
170.397
170.380
170.360
157.268
154.963
154.939
152.635
148.426
131.696
131.663
131.631
129.374
129.346
129.317
123.485
121.438
120.959
119.392
117.346
86.435
86.294
86.257
86.116
77.254
77.000
76.746
52.221
33.490
33.468
gxt-gg-125 C
CO2Me
F
F
F3CO
2l
180
170
160
150
140
130
120
110
100
90
80
70
60
50
40
30
20
10
f1 (ppm)

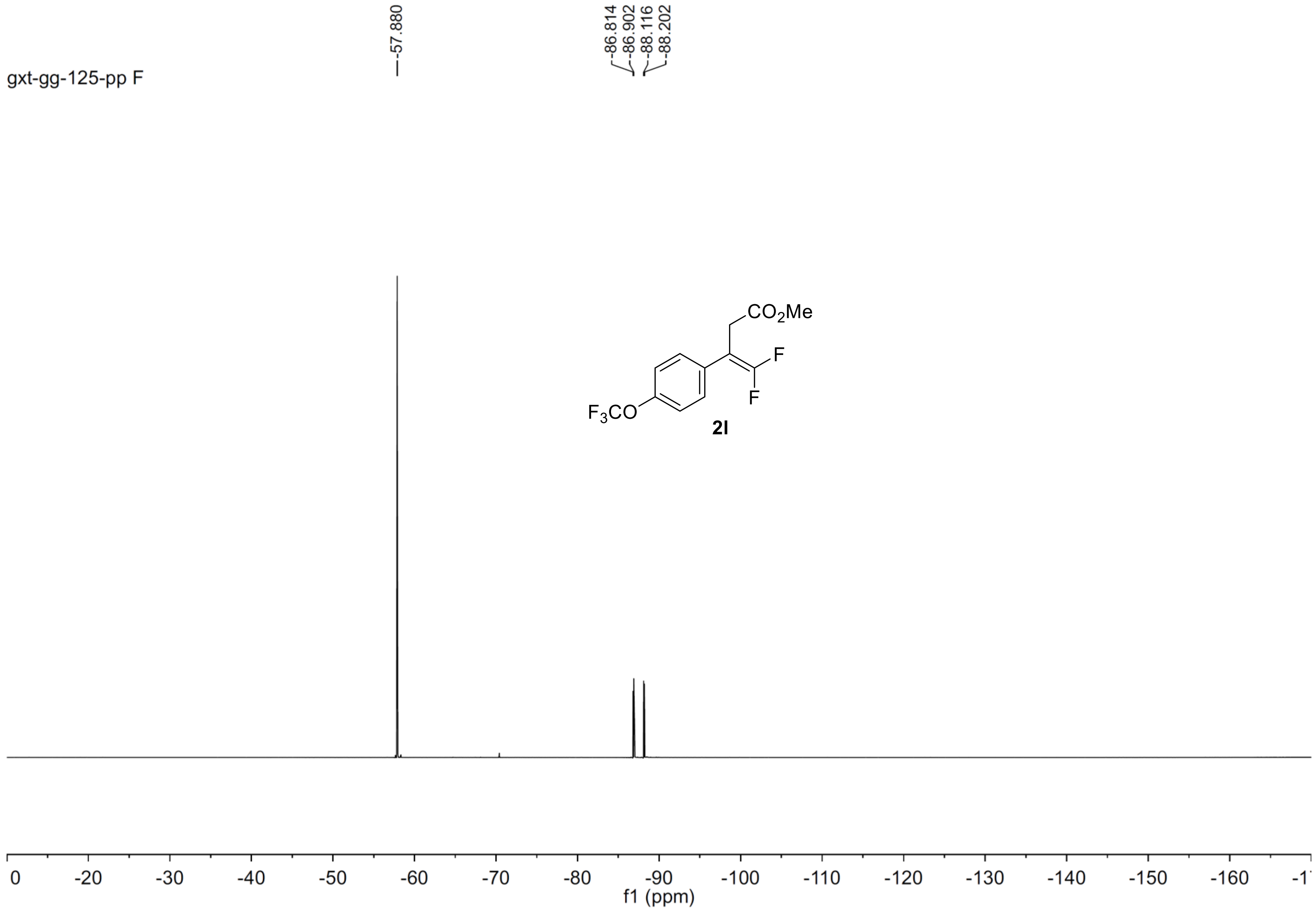
gxt-gg-125-pp F
-57.880
-86.814
-86.902
-88.116
-88.202
CO2Me
F
F
F3CO
2l
0
-20
-30
-40
-50
-60
-70
-80
-90
-100
-110
-120
-130
-140
-150
-160
f1 (ppm)

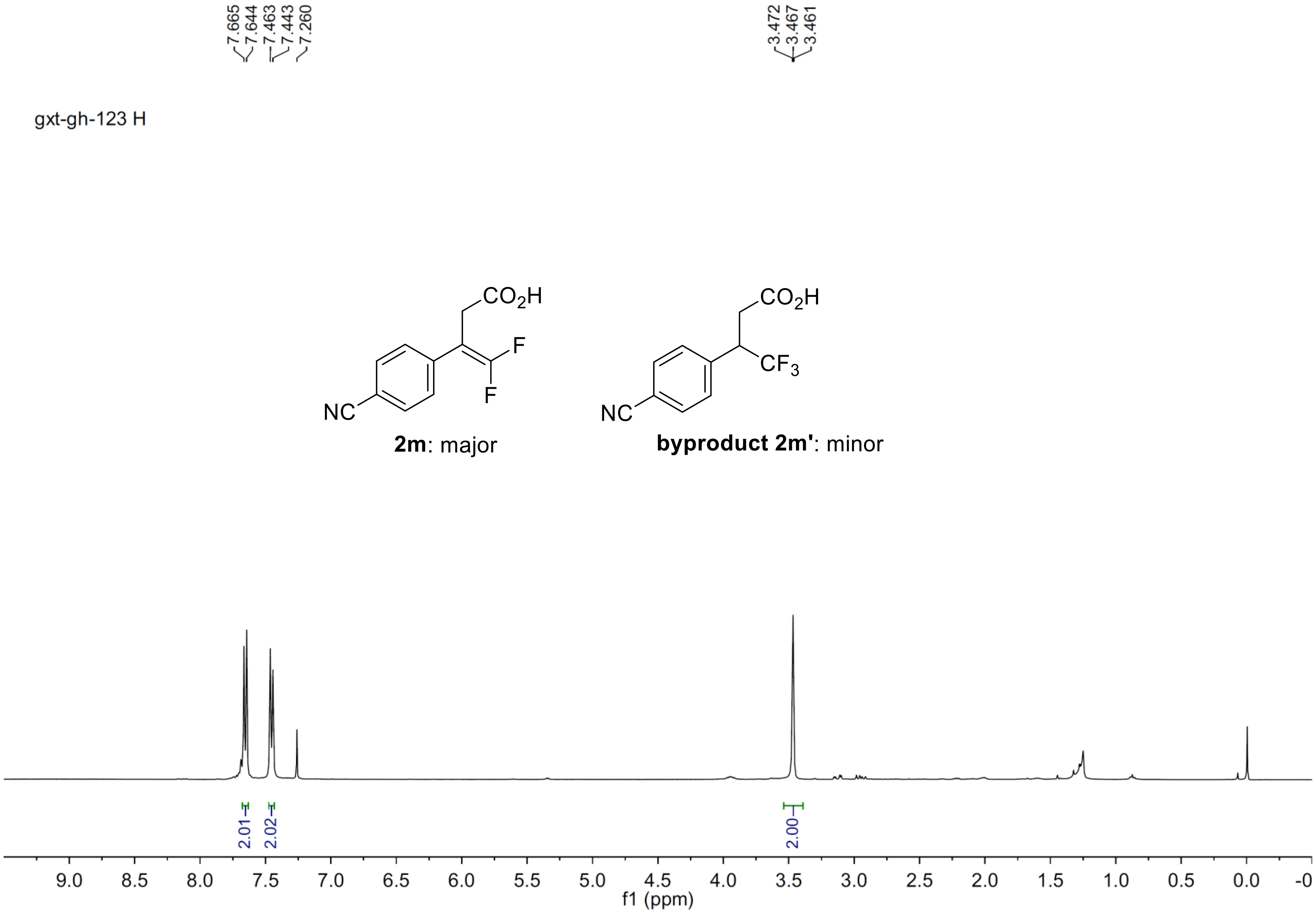
7.665
7.644
7.463
7.443
7.260
3.472
3.467
3.461
gxt-gh-123 H
CO2H
F
F
NC
2m: major
CO2H
CF3
NC
byproduct 2m': minor
2.01
2.02
2.00
9.0
8.5
8.0
7.5
7.0
6.5
6.0
5.5
5.0
4.5
4.0
3.5
3.0
2.5
2.0
1.5
1.0
0.5
0.0
-0
f1 (ppm)

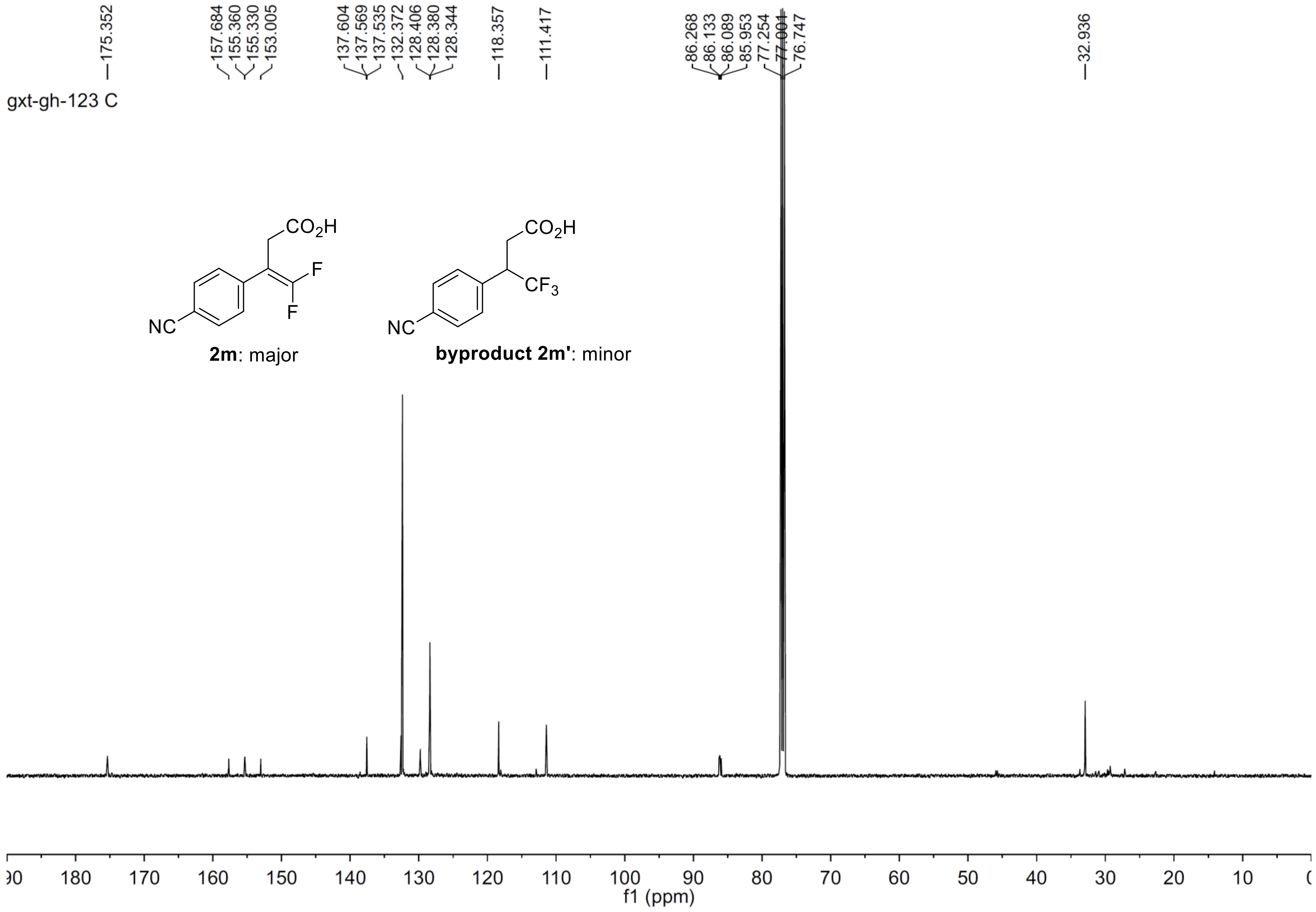

175.352
157.684
155.360
155.330
153.005
137.604
137.569
137.535
132.372
128.406
128.380
128.344
118.357
111.417
86.268
86.133
86.089
85.953
77.254
77.001
76.747
32.936
gxt-gh-123 C
CO2H
F
F
NC
2m: major
CO2H
CF3
NC
byproduct 2m': minor
180 170 160 150 140 130 120 110 100 90 80 70 60 50 40 30 20 10
f1 (ppm)

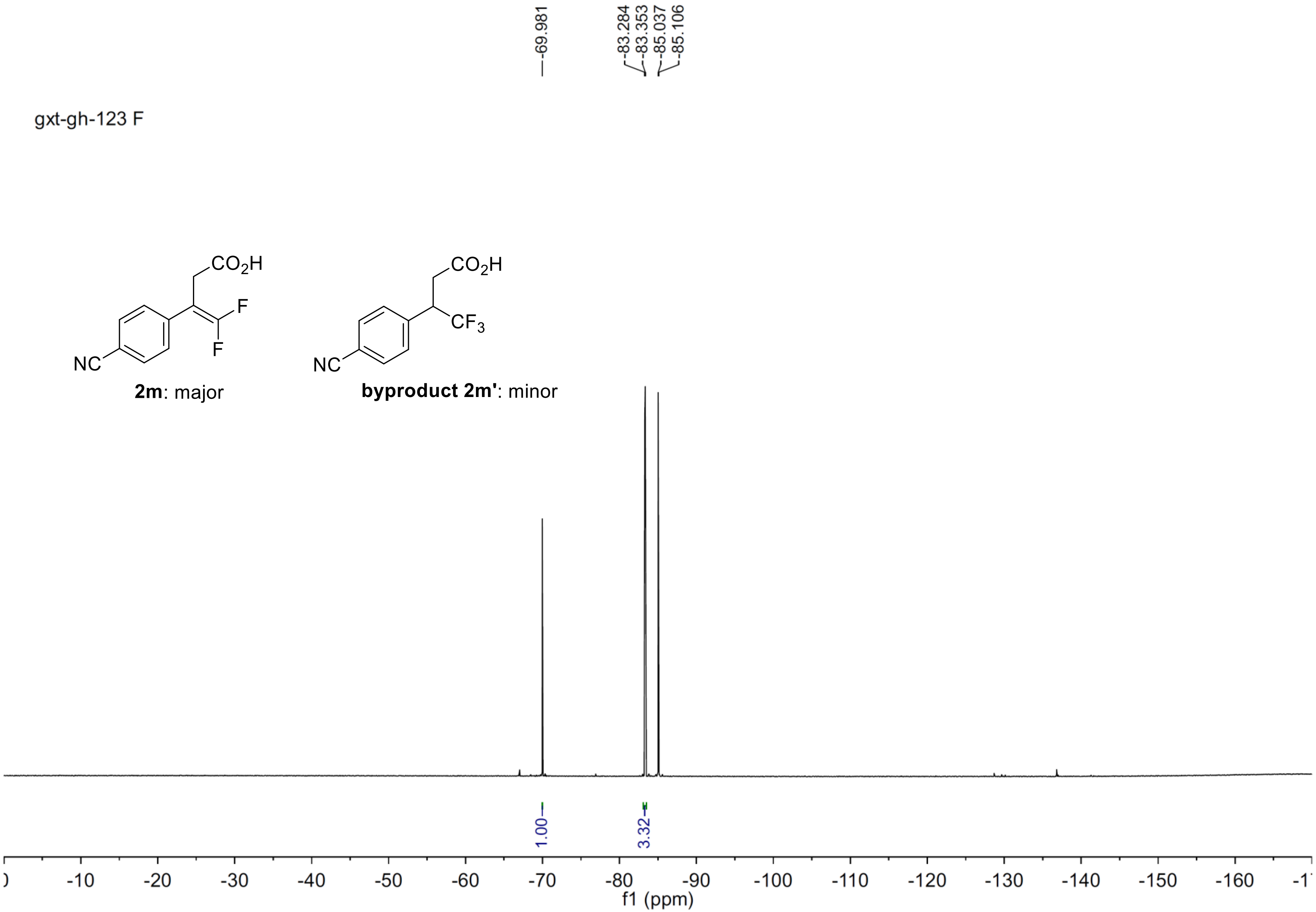

-69.981
-83.284
-83.353
-85.037
-85.106
gxt-gh-123 F
CO2H
F
F
NC
2m: major
CO2H
CF3
NC
byproduct 2m': minor
1.00
3.32
-10
-20
-30
-40
-50
-60
-70
-80
-90
-100
-110
-120
-130
-140
-150
-160
f1 (ppm)

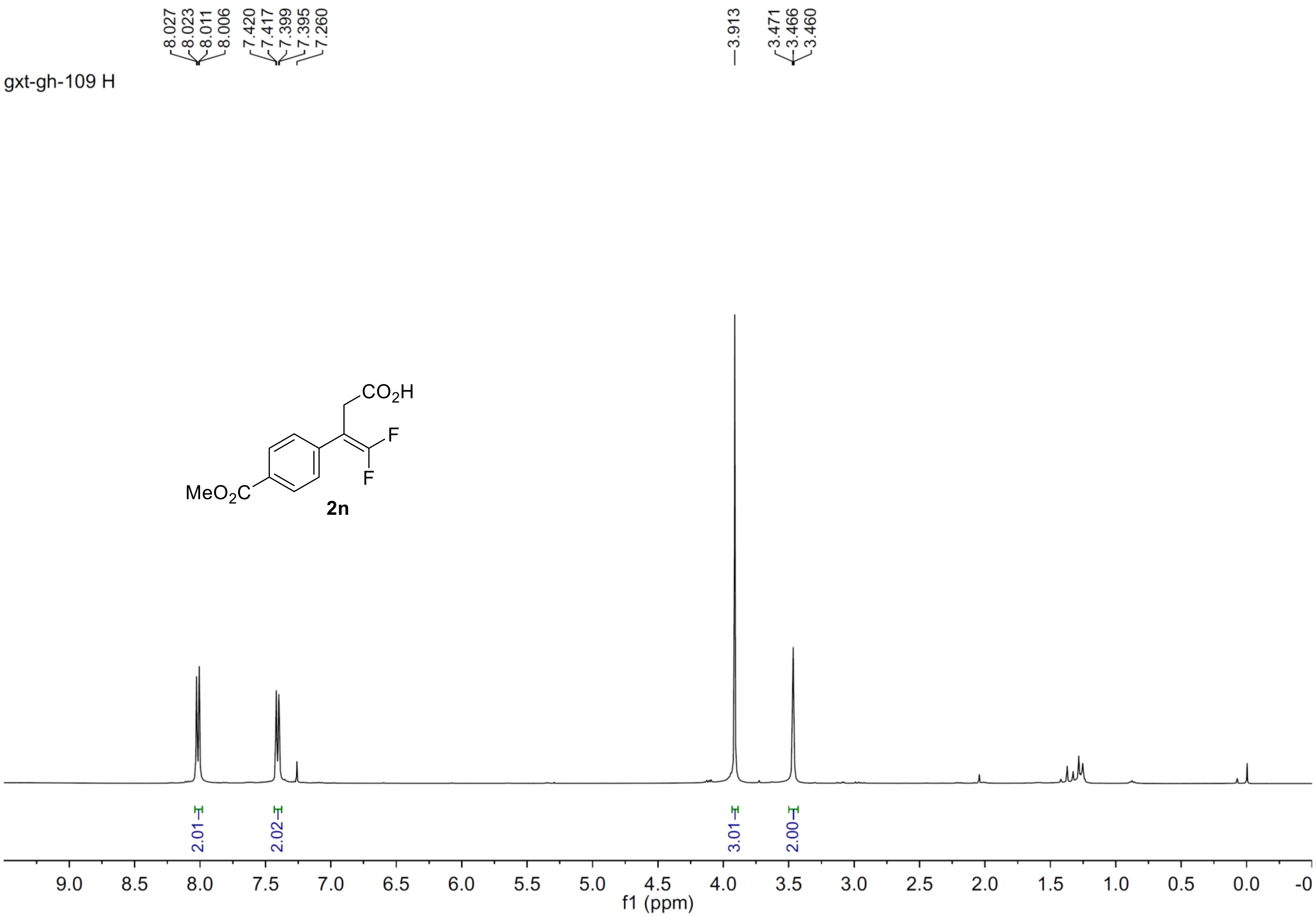
gxt-gh-109 H
8.027
8.023
8.011
8.006
7.420
7.417
7.399
7.395
7.260
3.913
3.471
3.466
3.460
CO_2H
F
F
MeO_2C
2n
2.01
2.02
3.01
2.00
9.0 8.5 8.0 7.5 7.0 6.5 6.0 5.5 5.0 4.5 4.0 3.5 3.0 2.5 2.0 1.5 1.0 0.5 0.0 -0
f1 (ppm)

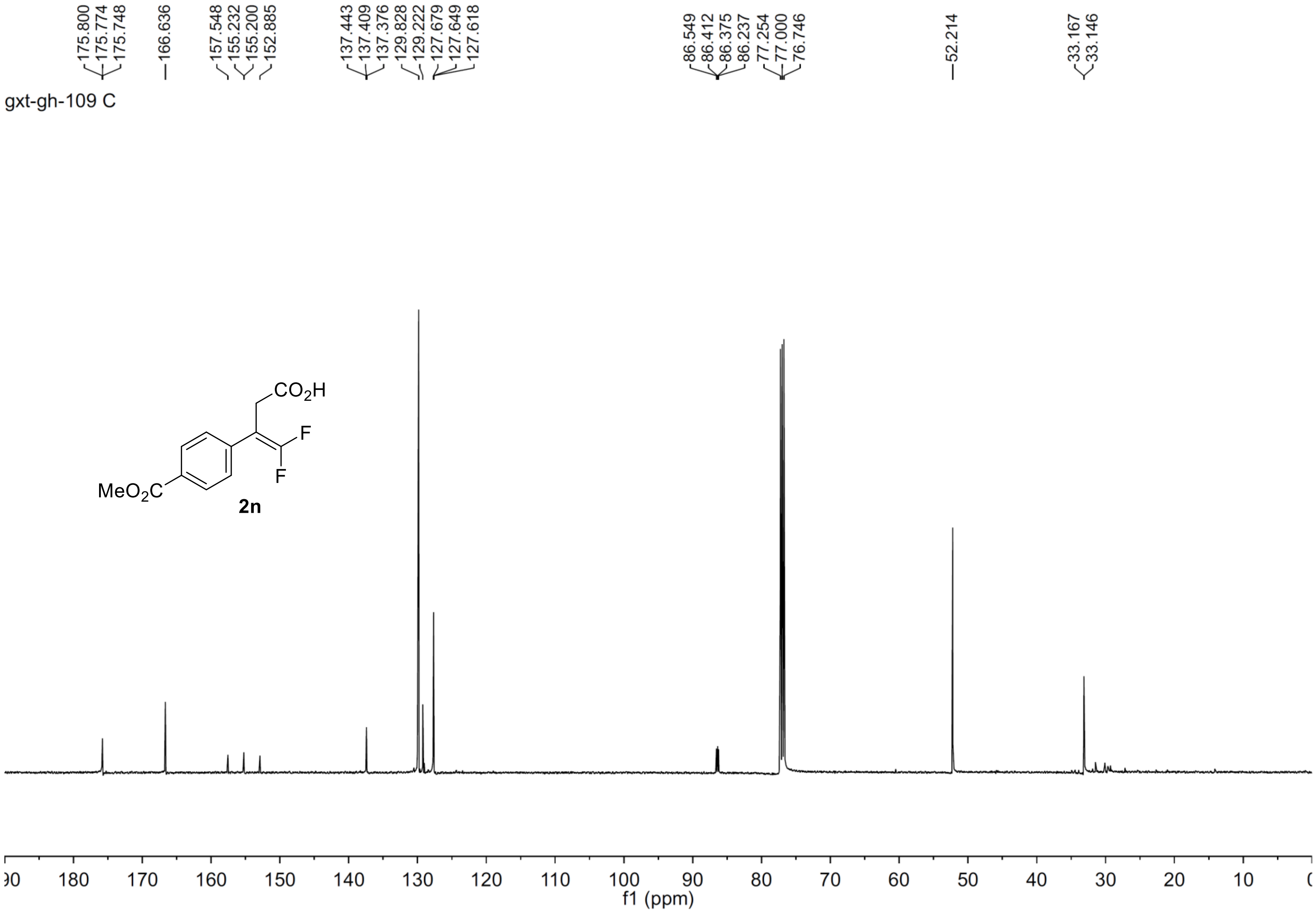
175.800
175.774
175.748
166.636
157.548
155.232
155.200
152.885
137.443
137.409
137.376
129.828
129.222
127.679
127.649
127.618
86.549
86.412
86.375
86.237
77.254
77.000
76.746
52.214
33.167
33.146
gxt-gh-109 C
CO2H
F
F
MeO2C
2n
f1 (ppm)

gxt-gh-109 F

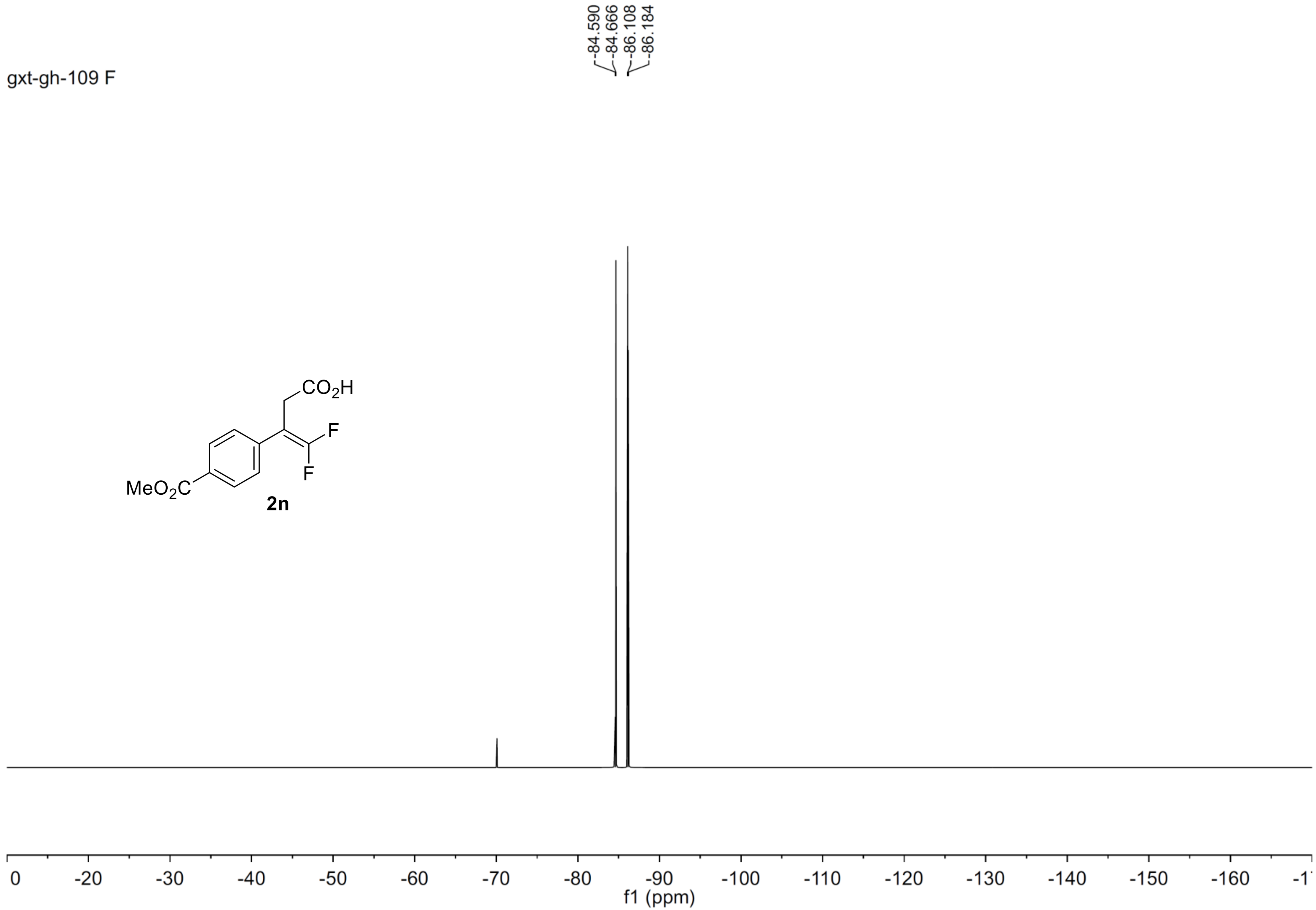
-84.590
-84.666
-86.108
-86.184
CO2H
F
F
MeO2C
2n
0
-20
-30
-40
-50
-60
-70
-80
-90
-100
-110
-120
-130
-140
-150
-160
f1 (ppm)

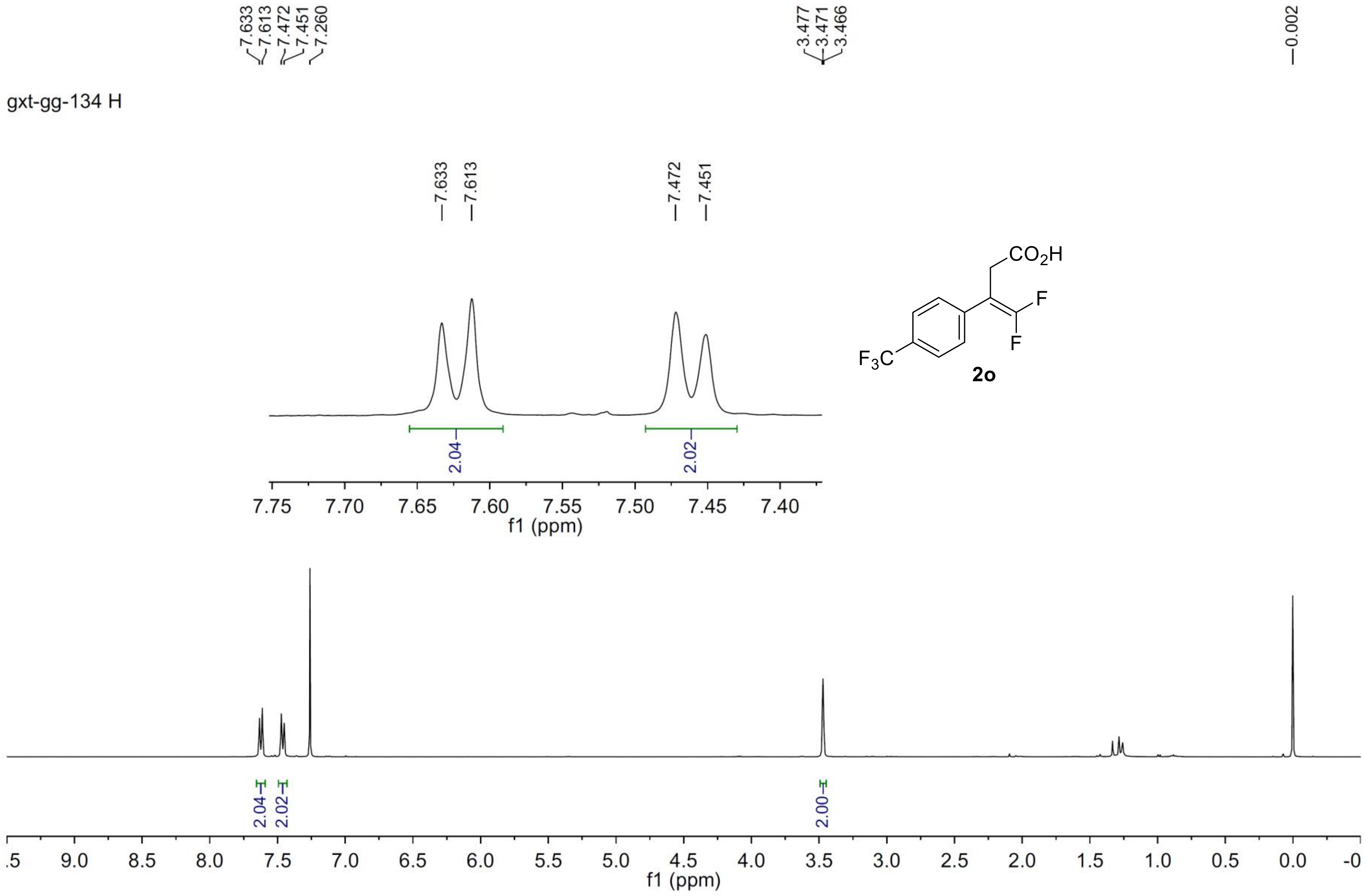
gxt-gg-134 H
7.633
7.613
7.472
7.451
7.260
3.477
3.471
3.466
0.002
CO2H
F
F
F3C
2o
2.04
2.02
7.75 7.70 7.65 7.60 7.55 7.50 7.45 7.40
f1 (ppm)
2.04
2.02
2.00
.5 9.0 8.5 8.0 7.5 7.0 6.5 6.0 5.5 5.0 4.5 4.0 3.5 3.0 2.5 2.0 1.5 1.0 0.5 0.0 -0
f1 (ppm)

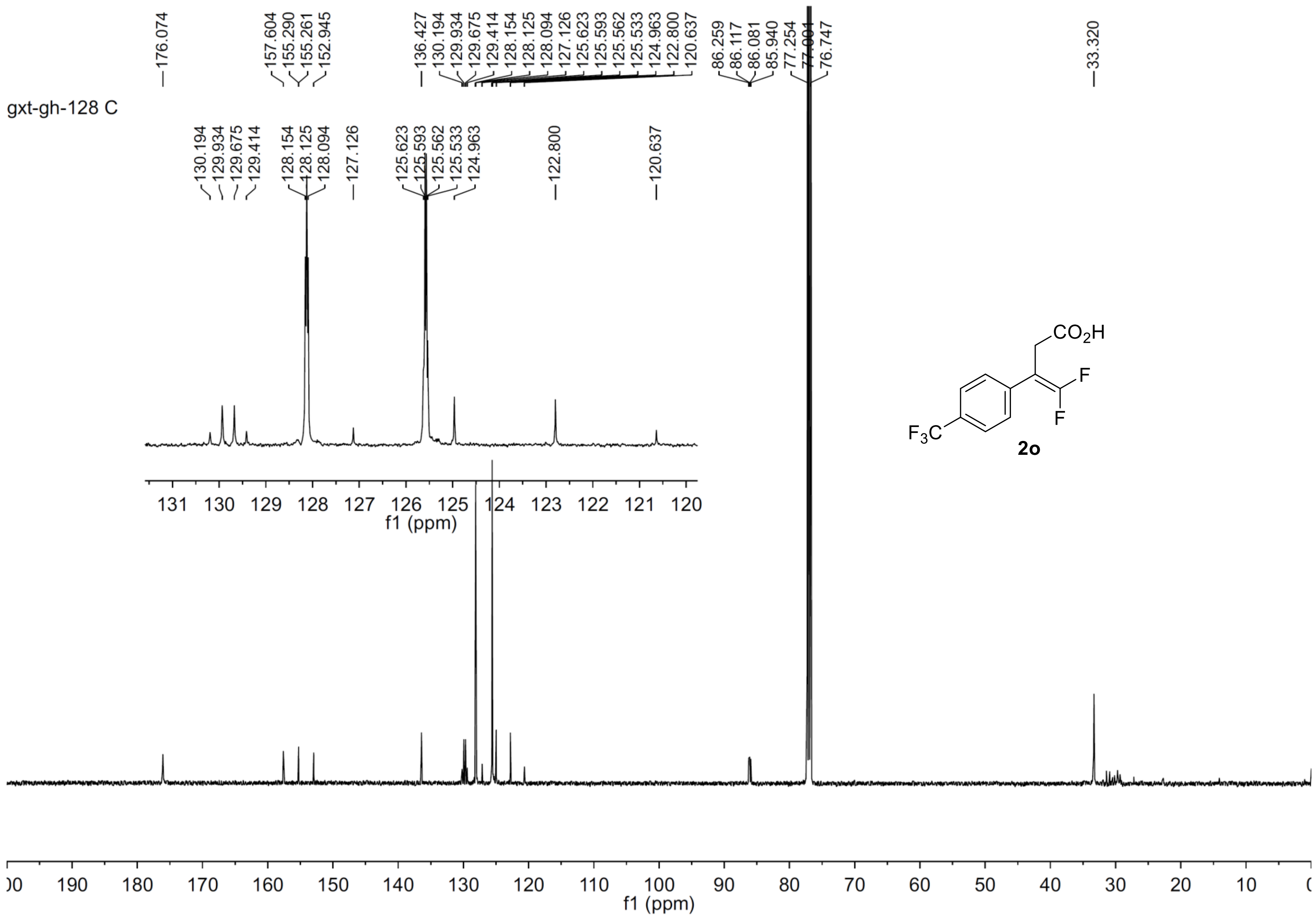
gxt-gh-128 C
176.074
157.604
155.290
155.261
152.945
136.427
130.194
129.934
129.675
129.414
128.154
128.125
128.094
127.126
125.623
125.593
125.562
125.533
124.963
122.800
120.637
86.259
86.117
86.081
85.940
77.254
77.001
76.747
33.320
f1 (ppm)
CO2H
F
F
F3C
2o

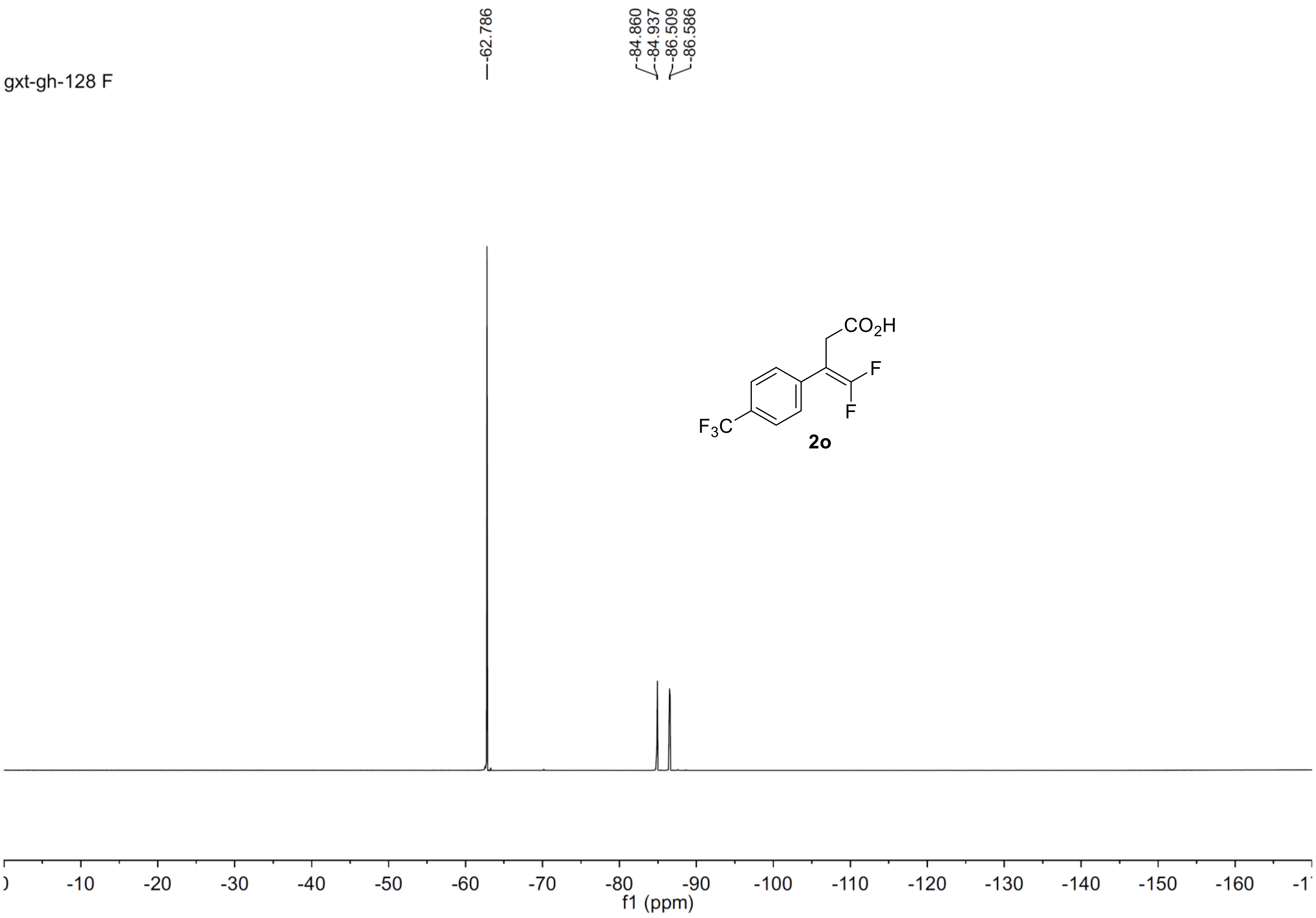
gxt-gh-128 F
-62.786
-84.860
-84.937
-86.509
-86.586
CO2H
F
F
F3C
2o
-10
-20
-30
-40
-50
-60
-70
-80
-90
-100
-110
-120
-130
-140
-150
-160
f1 (ppm)

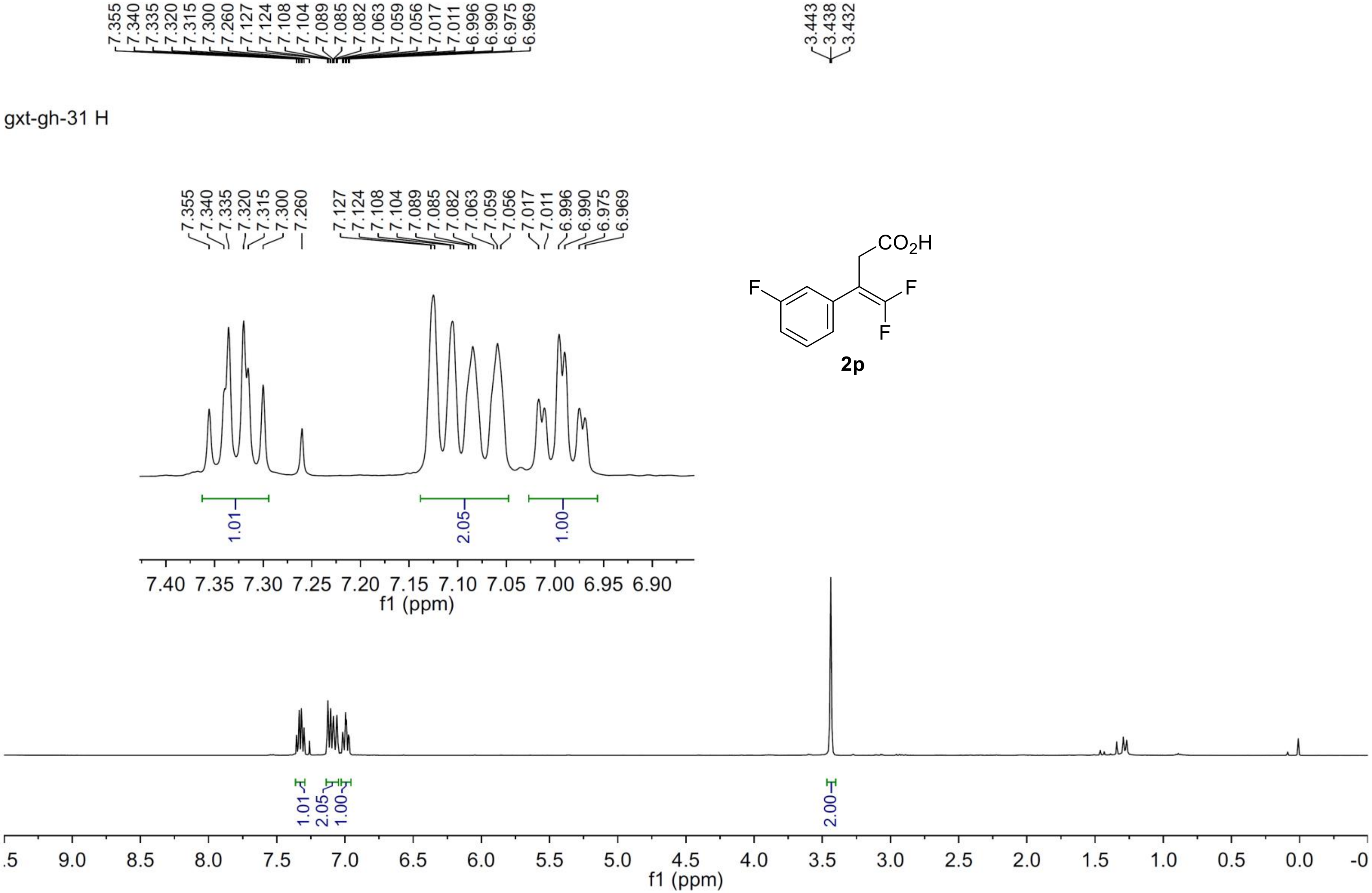
gxt-gh-31 H
7.355 7.340 7.335 7.320 7.315 7.300 7.260 7.127 7.124 7.108 7.104 7.089 7.085 7.082 7.063 7.059 7.056 7.017 7.011 6.996 6.990 6.975 6.969
3.443 3.438 3.432
CO2H
F
F
F
2p
1.01
2.05
1.00
2.00
7.40 7.35 7.30 7.25 7.20 7.15 7.10 7.05 7.00 6.95 6.90
f1 (ppm)
.5 9.0 8.5 8.0 7.5 7.0 6.5 6.0 5.5 5.0 4.5 4.0 3.5 3.0 2.5 2.0 1.5 1.0 0.5 0.0 -0
f1 (ppm)

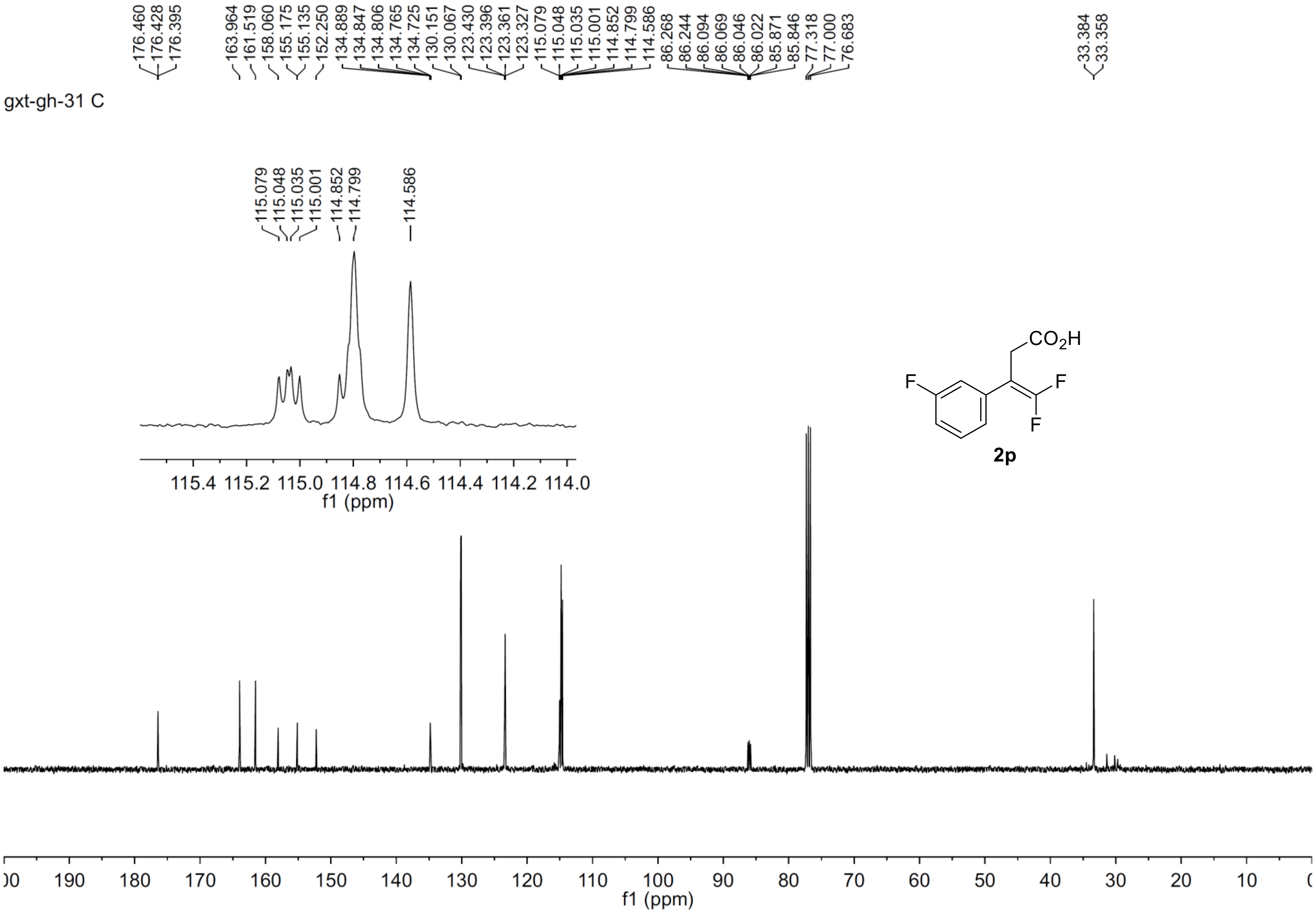
gxt-gh-31 C
176.460
176.428
176.395
163.964
161.519
158.060
155.175
155.135
152.250
134.889
134.847
134.806
134.765
134.725
130.151
130.067
123.430
123.396
123.361
123.327
115.079
115.048
115.035
115.001
114.852
114.799
114.586
86.268
86.244
86.094
86.069
86.046
86.022
85.871
85.846
77.318
77.000
76.683
33.384
33.358
115.4 115.2 115.0 114.8 114.6 114.4 114.2 114.0
f1 (ppm)
CO2H
F
F
F
2p
190 180 170 160 150 140 130 120 110 100 90 80 70 60 50 40 30 20 10
f1 (ppm)

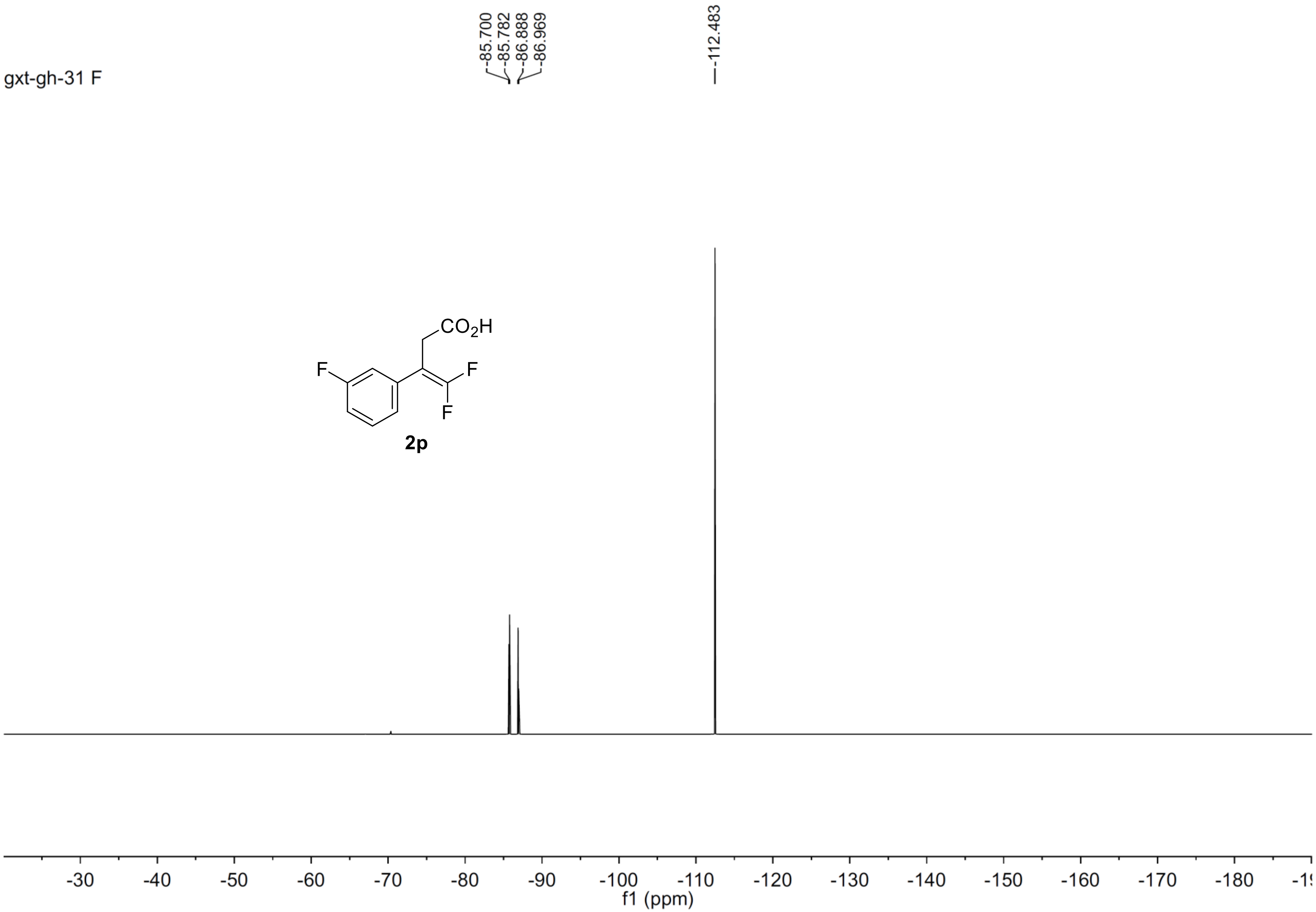
gxt-gh-31 F
-85.700
-85.782
-86.888
-86.969
-112.483
CO2H
F
F
F
2p
-30
-40
-50
-60
-70
-80
-90
-100
-110
-120
-130
-140
-150
-160
-170
-180
f1 (ppm)

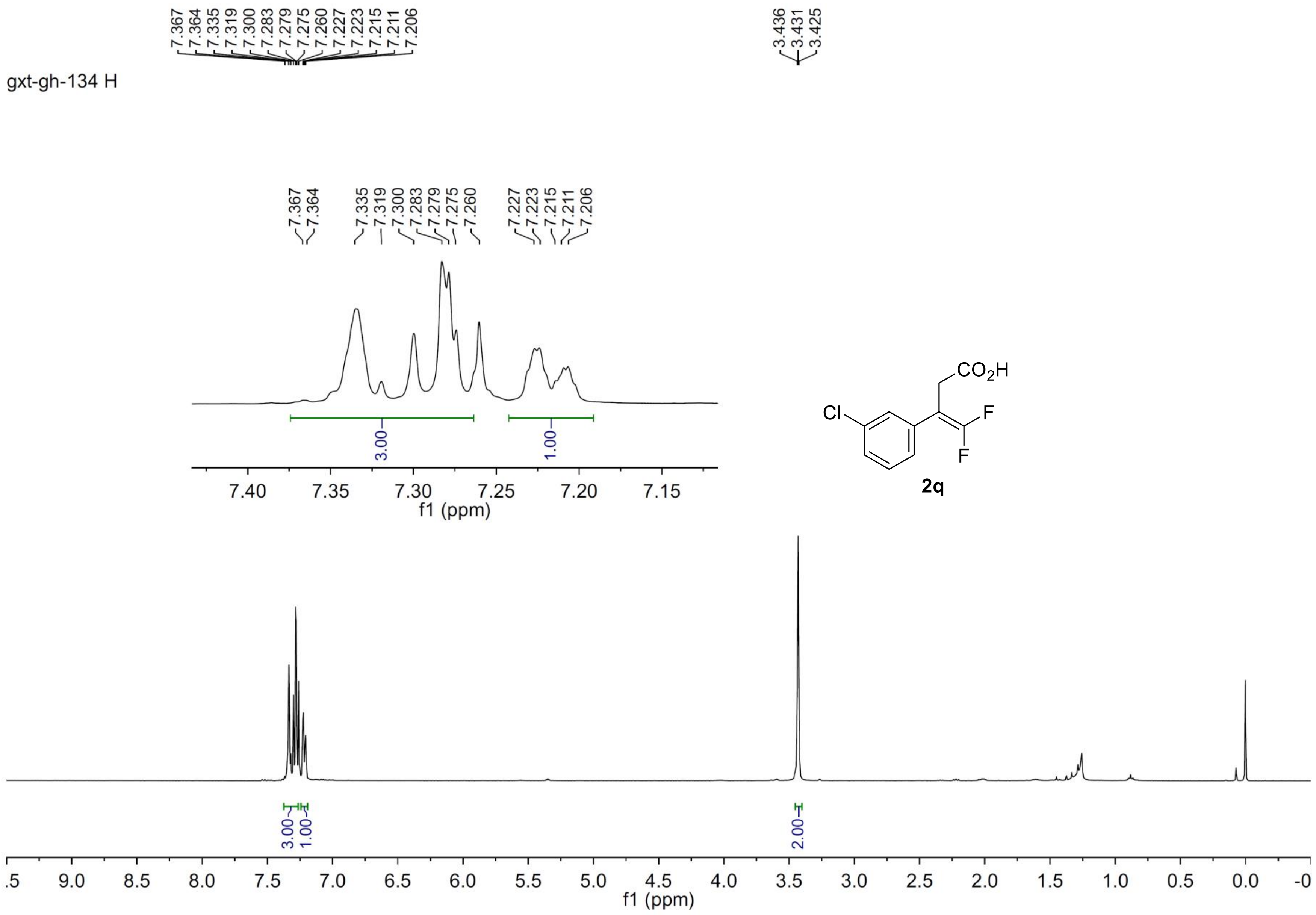
gxt-gh-134 H
7.367
7.364
7.335
7.319
7.300
7.283
7.279
7.275
7.260
7.227
7.223
7.215
7.211
7.206
3.436
3.431
3.425
3.00
1.00
2.00
7.40
7.35
7.30
7.25
7.20
7.15
f1 (ppm)
9.0
8.5
8.0
7.5
7.0
6.5
6.0
5.5
5.0
4.5
4.0
3.5
3.0
2.5
2.0
1.5
1.0
0.5
0.0
CO2H
Cl
F
F

2q

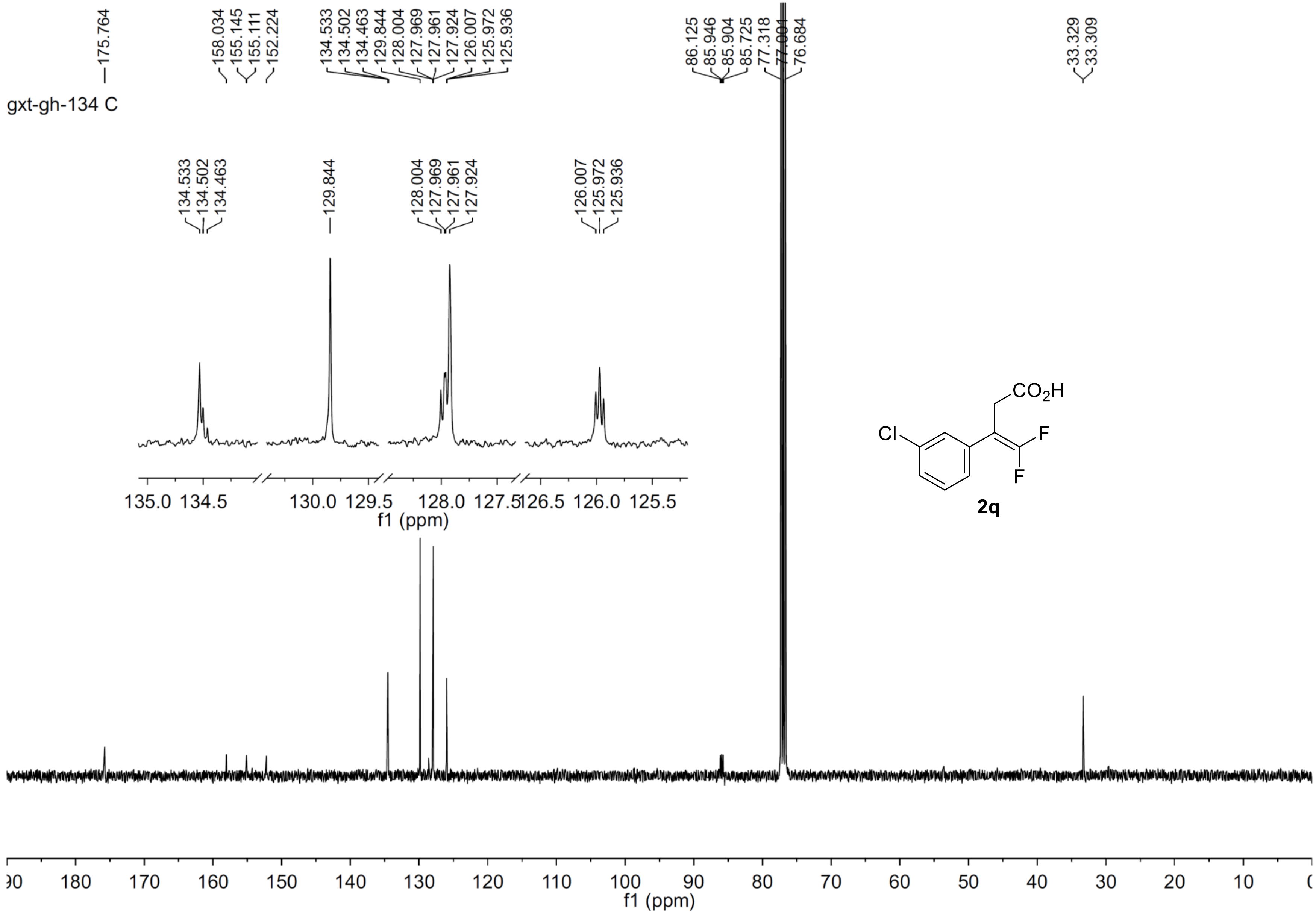
gxt-gh-134 C
175.764
158.034
155.145
155.111
152.224
134.533
134.502
134.463
129.844
128.004
127.969
127.961
127.924
126.007
125.972
125.936
86.125
85.946
85.904
85.725
77.318
77.001
76.684
33.329
33.309
135.0 134.5 130.0 129.5 128.0 127.5 126.5 126.0 125.5
f1 (ppm)
CO2H
Cl
F
F
2q
180 170 160 150 140 130 120 110 100 90 80 70 60 50 40 30 20 10
f1 (ppm)

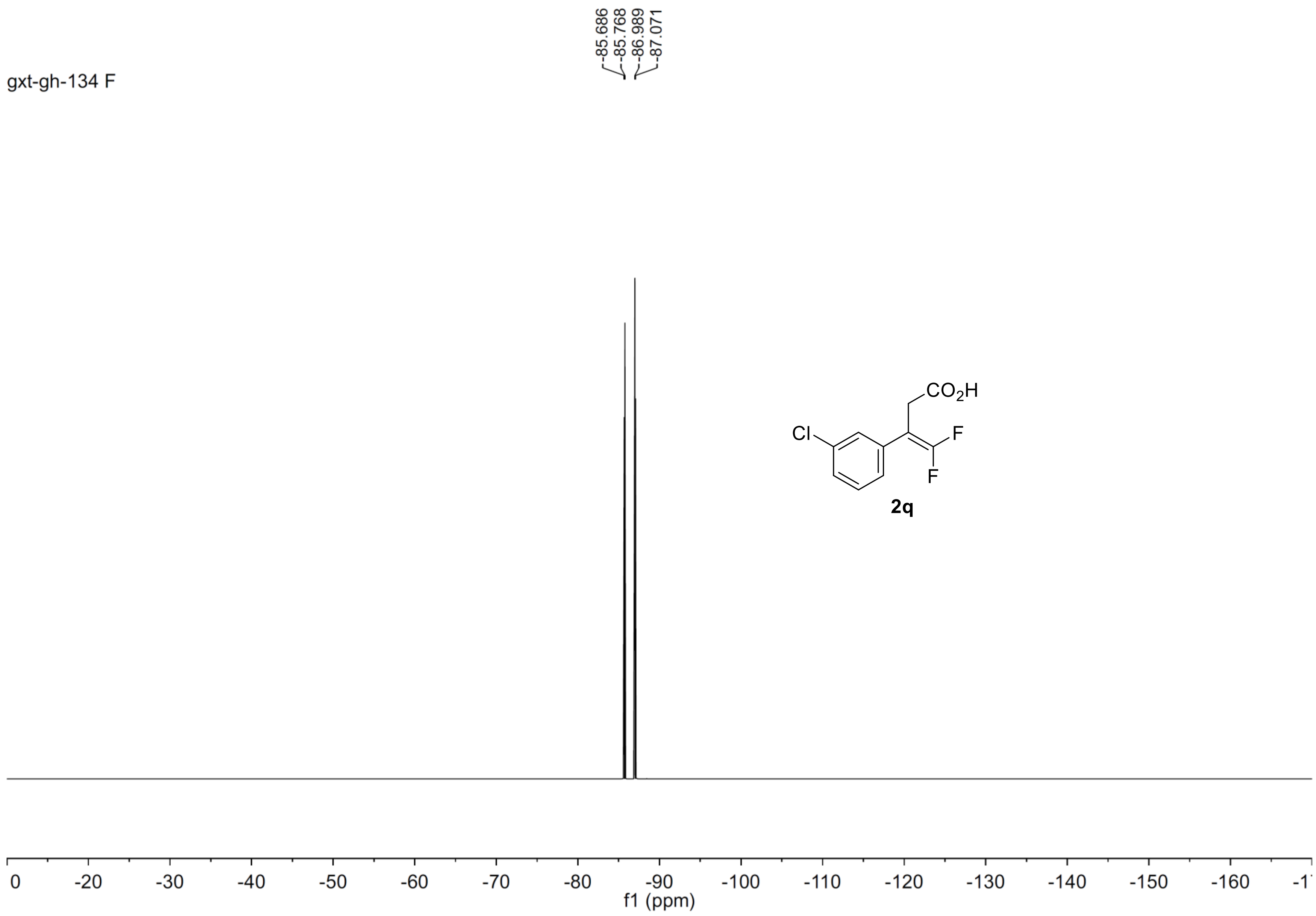
gxt-gh-134 F
-85.686
-85.768
-86.989
-87.071
CO2H
Cl
F
F
2q
0
-20
-30
-40
-50
-60
-70
-80
-90
-100
-110
-120
-130
-140
-150
-160
f1 (ppm)

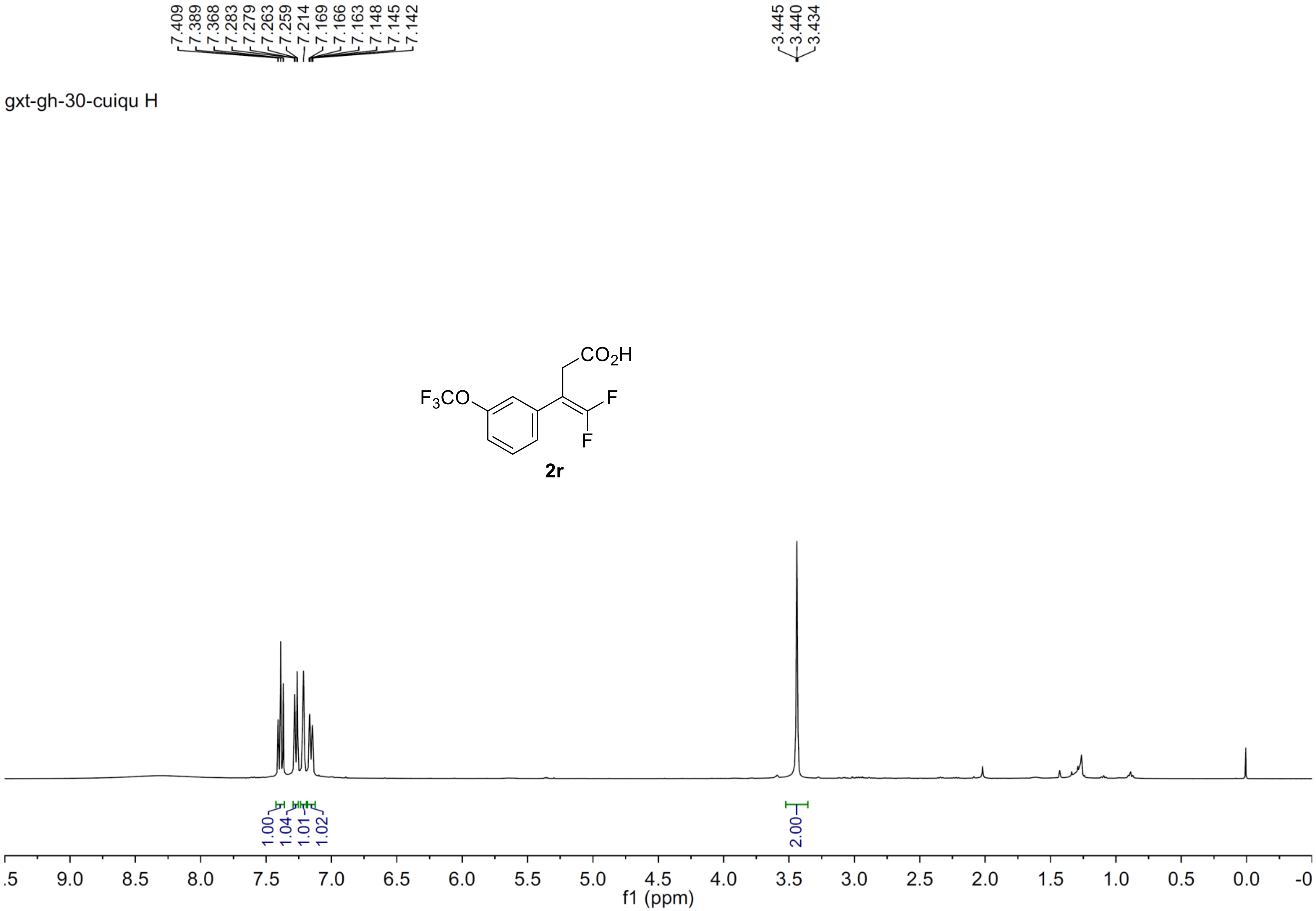
7.409
7.389
7.368
7.283
7.279
7.263
7.259
7.214
7.169
7.166
7.163
7.148
7.145
7.142
3.445
3.440
3.434
gxt-gh-30-cuiqu H
CO2H
F3CO
F
F
2r
1.00
1.04
1.01
1.02
2.00
f1 (ppm)

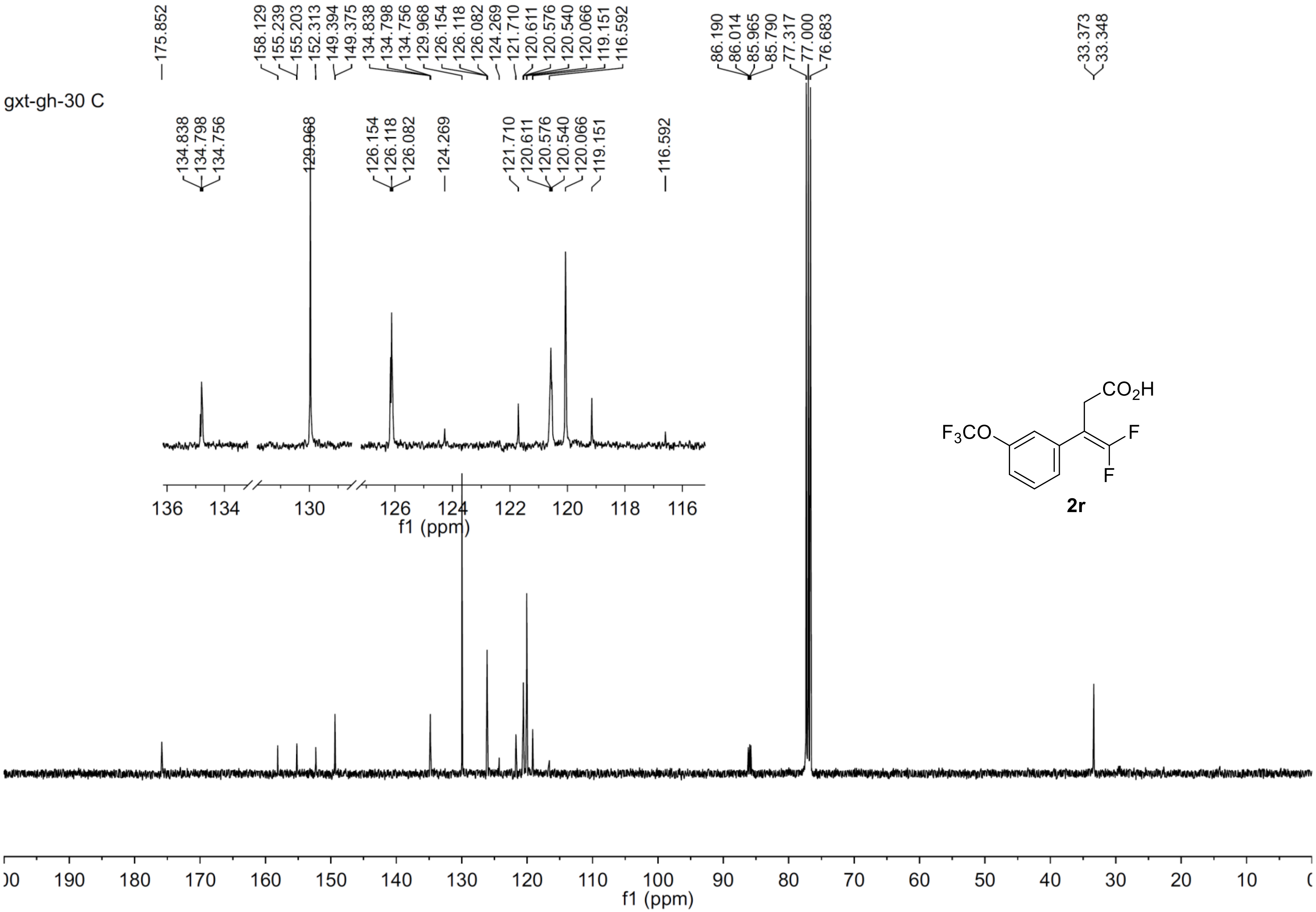
gxt-gh-30 C
175.852
158.129
155.239
155.203
152.313
149.394
149.375
134.838
134.798
134.756
129.968
126.154
126.118
126.082
124.269
121.710
120.611
120.576
120.540
120.066
119.151
116.592
86.190
86.014
85.965
85.790
77.317
77.000
76.683
33.373
33.348
f1 (ppm)
F3CO
CO2H
F
F
2r

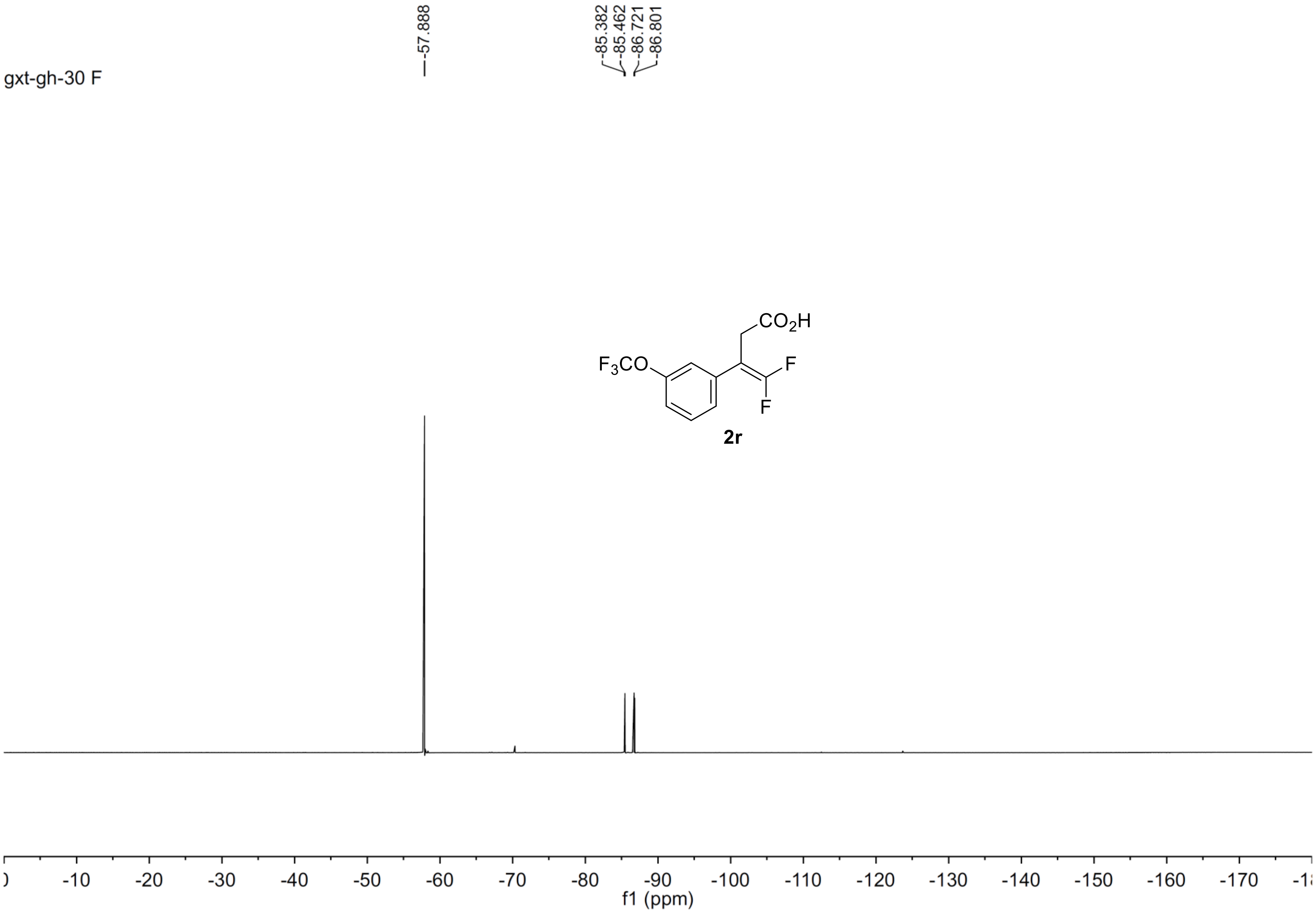
gxt-gh-30 F
-57.888
-85.382
-85.462
-86.721
-86.801
CO2H
F3CO
F
F
2r
-10
-20
-30
-40
-50
-60
-70
-80
-90
-100
-110
-120
-130
-140
-150
-160
-170
f1 (ppm)

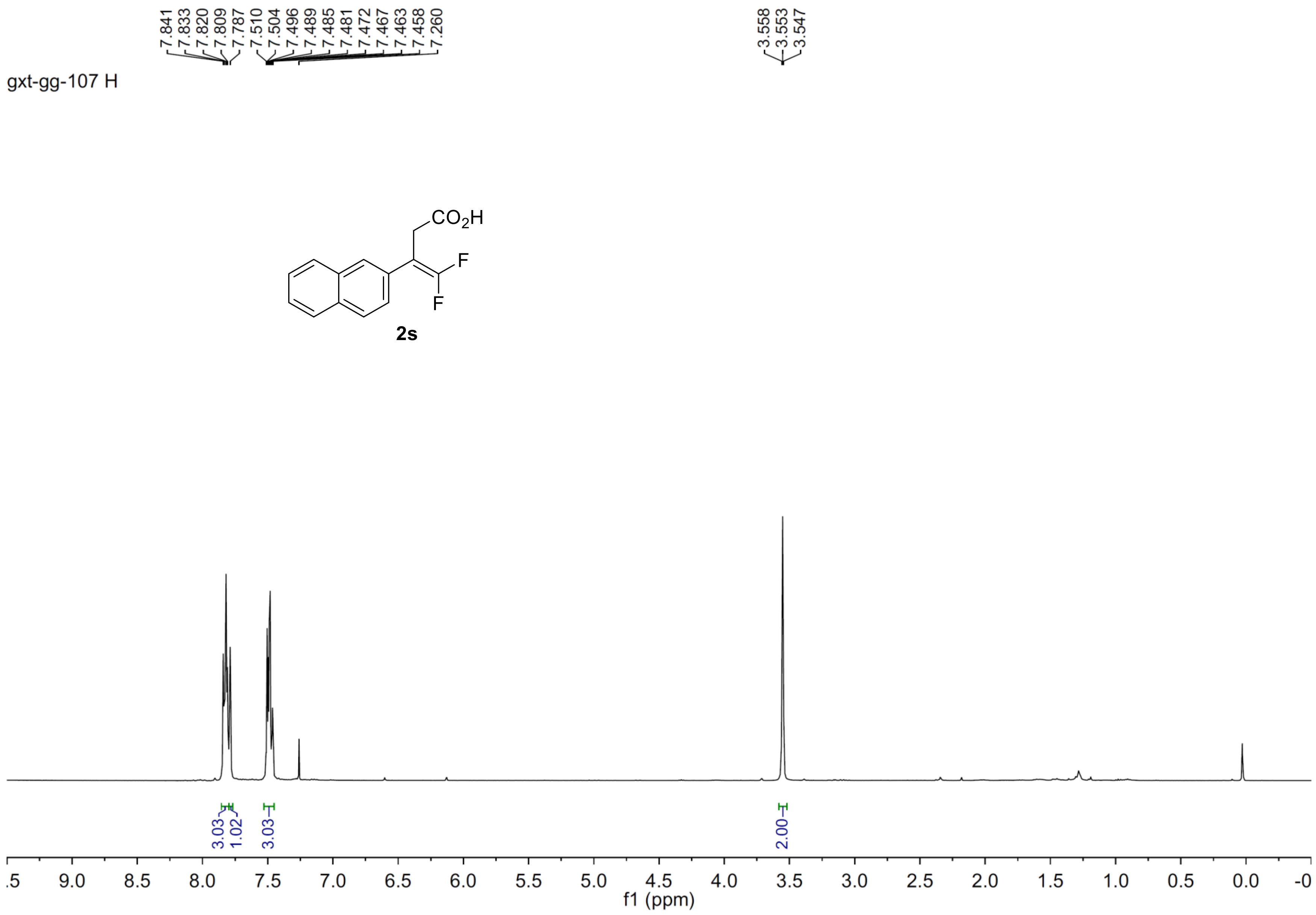
gxt-gg-107 H
7.841
7.833
7.820
7.809
7.787
7.510
7.504
7.496
7.489
7.485
7.481
7.472
7.467
7.463
7.458
7.260
3.558
3.553
3.547
CO_2H
F
F
2s
3.03
1.02
3.03
2.00
.5 9.0 8.5 8.0 7.5 7.0 6.5 6.0 5.5 5.0 4.5 4.0 3.5 3.0 2.5 2.0 1.5 1.0 0.5 0.0 -0
f1 (ppm)

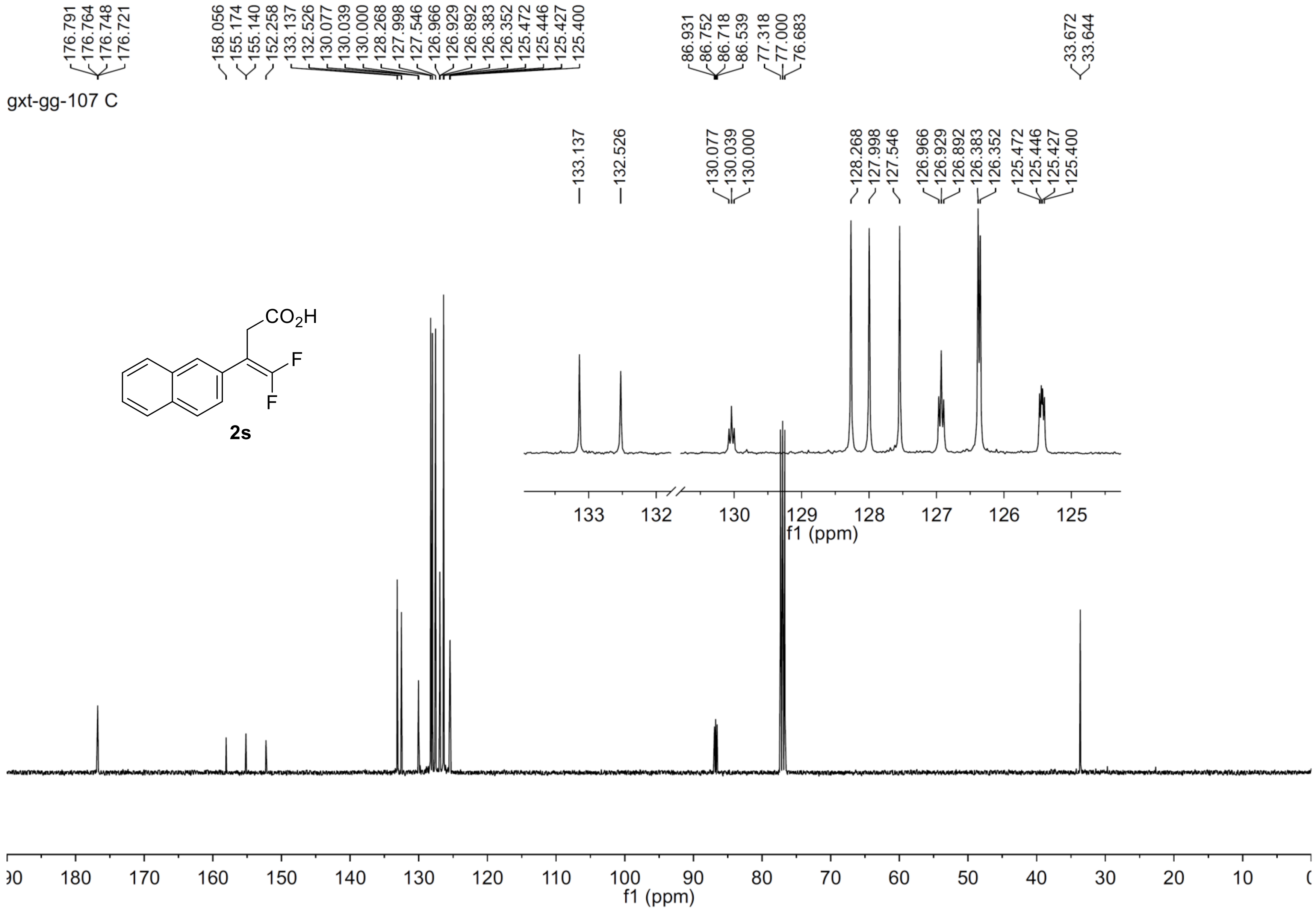
gxt-gg-107 C
176.791
176.764
176.748
176.721
158.056
155.174
155.140
152.258
133.137
132.526
130.077
130.039
130.000
128.268
127.998
127.546
126.966
126.929
126.892
126.383
126.352
125.472
125.446
125.427
125.400
86.931
86.752
86.718
86.539
77.318
77.000
76.683
33.672
33.644
CO2H
F
F
2s
f1 (ppm)
f1 (ppm)

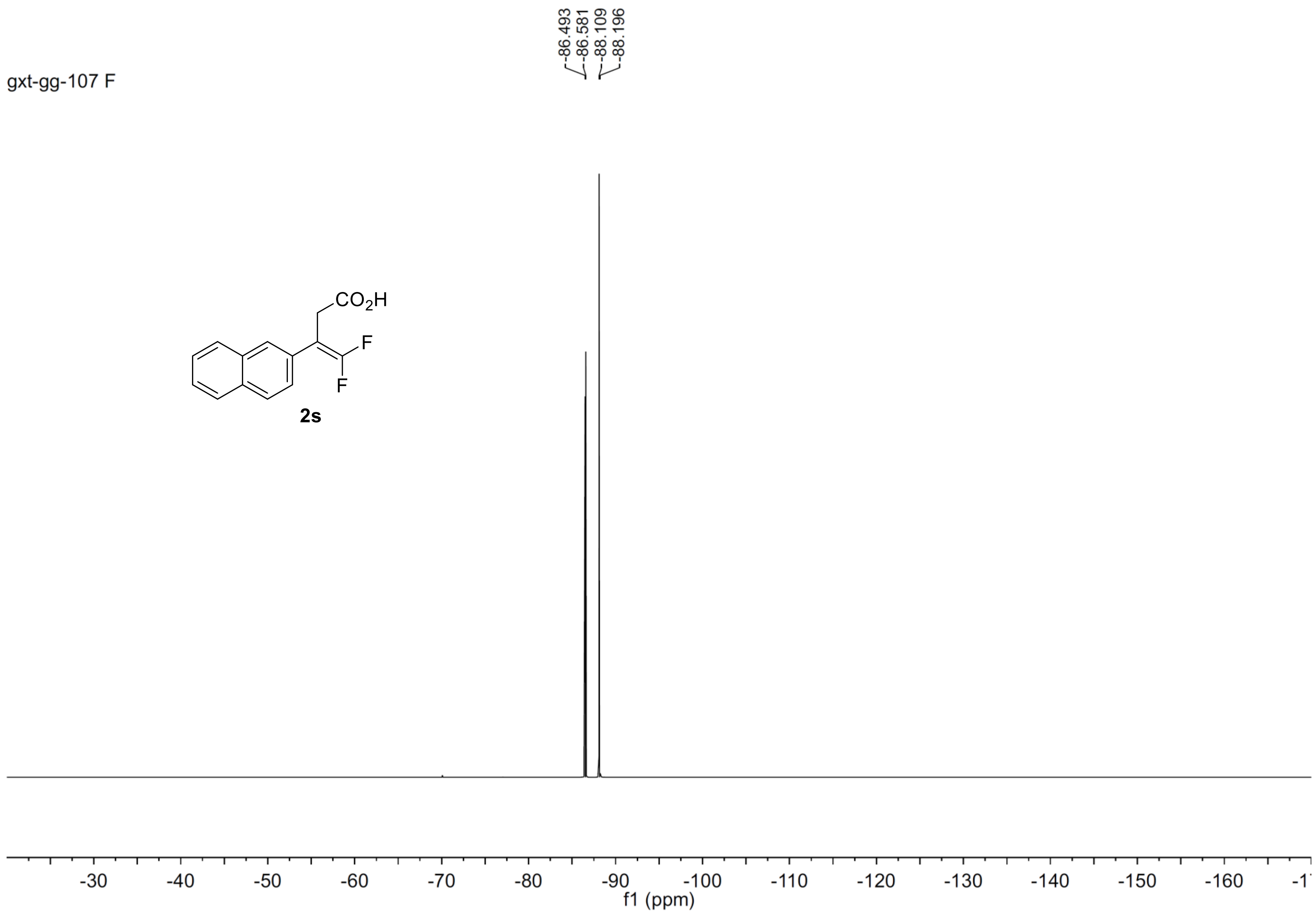
gxt-gg-107 F
-86.493
-86.581
-88.109
-88.196
CO2H
F
F
2s
-30
-40
-50
-60
-70
-80
-90
-100
-110
-120
-130
-140
-150
-160
f1 (ppm)

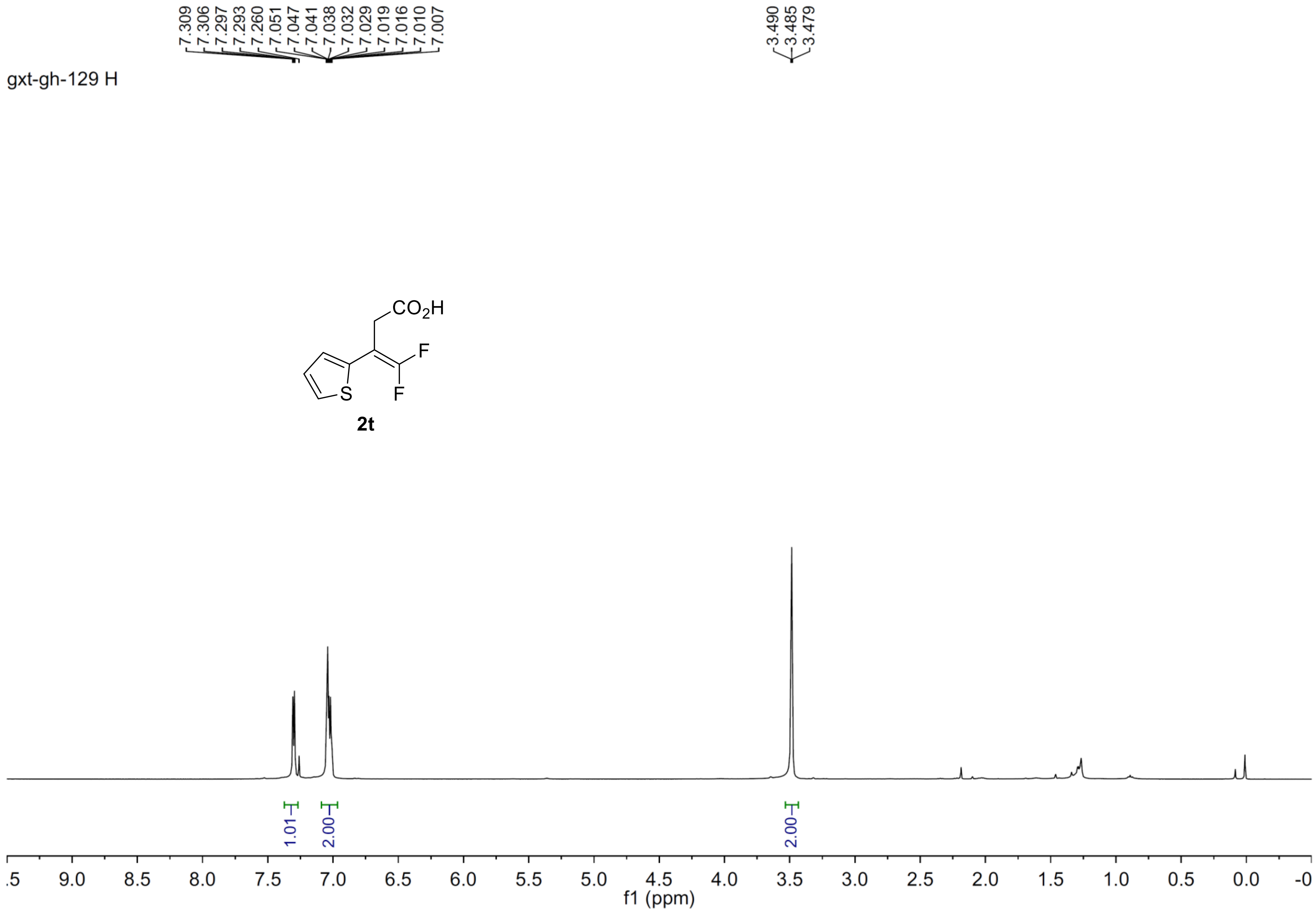
gxt-gh-129 H
7.309
7.306
7.297
7.293
7.260
7.051
7.047
7.041
7.038
7.032
7.029
7.019
7.016
7.010
7.007
3.490
3.485
3.479
CO2H
F
F
S
2t
1.01
2.00
2.00
.5
9.0
8.5
8.0
7.5
7.0
6.5
6.0
5.5
5.0
4.5
4.0
3.5
3.0
2.5
2.0
1.5
1.0
0.5
0.0
-0
f1 (ppm)

2s

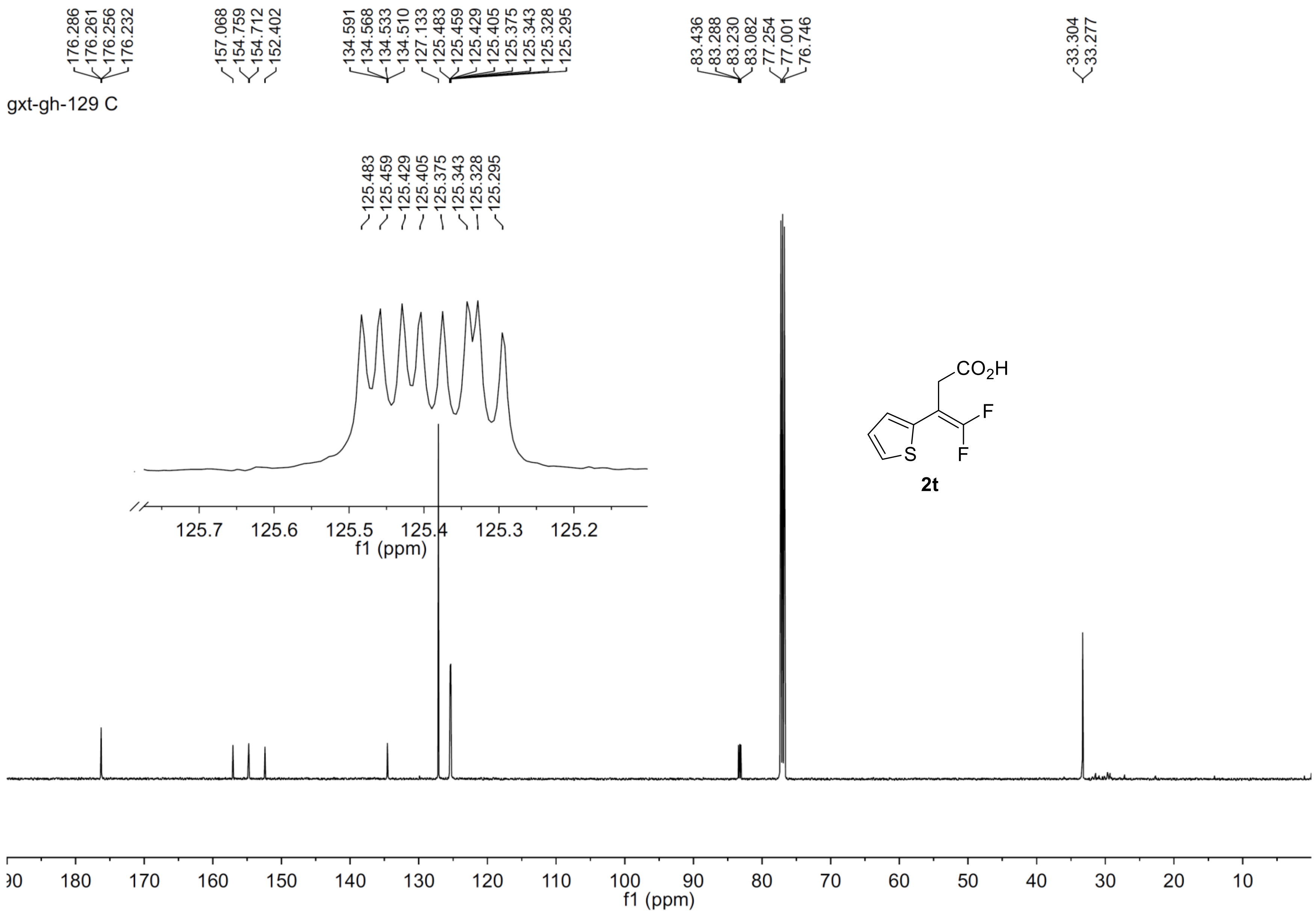

176.286
176.261
176.256
176.232
157.068
154.759
154.712
152.402
134.591
134.568
134.533
134.510
127.133
125.483
125.459
125.429
125.405
125.375
125.343
125.328
125.295
83.436
83.288
83.230
83.082
77.254
77.001
76.746
33.304
33.277
gxt-gh-129 C
125.7 125.6 125.5 125.4 125.3 125.2
f1 (ppm)
CO_2H
F
F
S
2t
90 180 170 160 150 140 130 120 110 100 90 80 70 60 50 40 30 20 10
f1 (ppm)

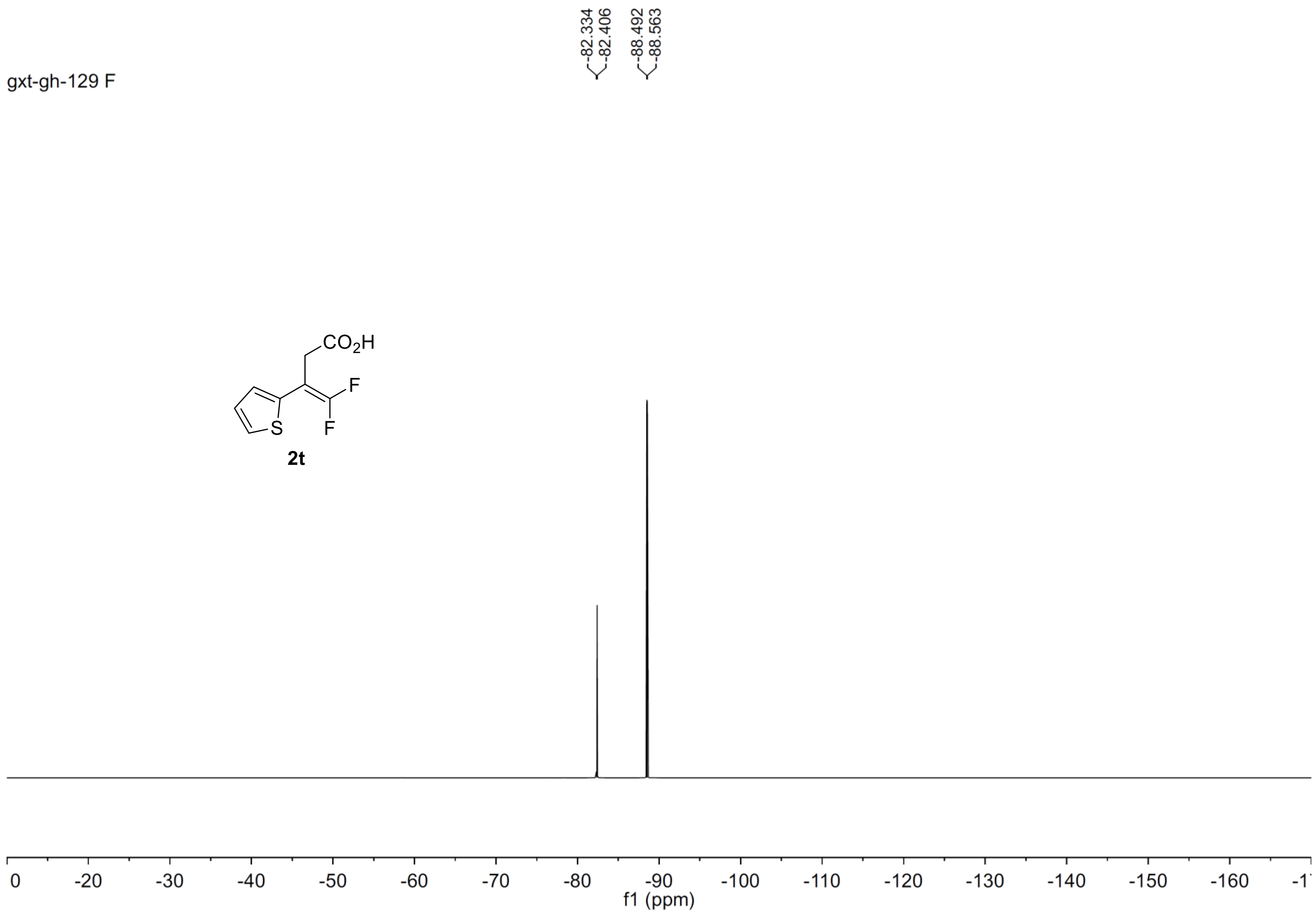
gxt-gh-129 F
-82.334
-82.406
-88.492
-88.563
CO2H
F
F
S
2t
0
-20
-30
-40
-50
-60
-70
-80
-90
-100
-110
-120
-130
-140
-150
-160
f1 (ppm)

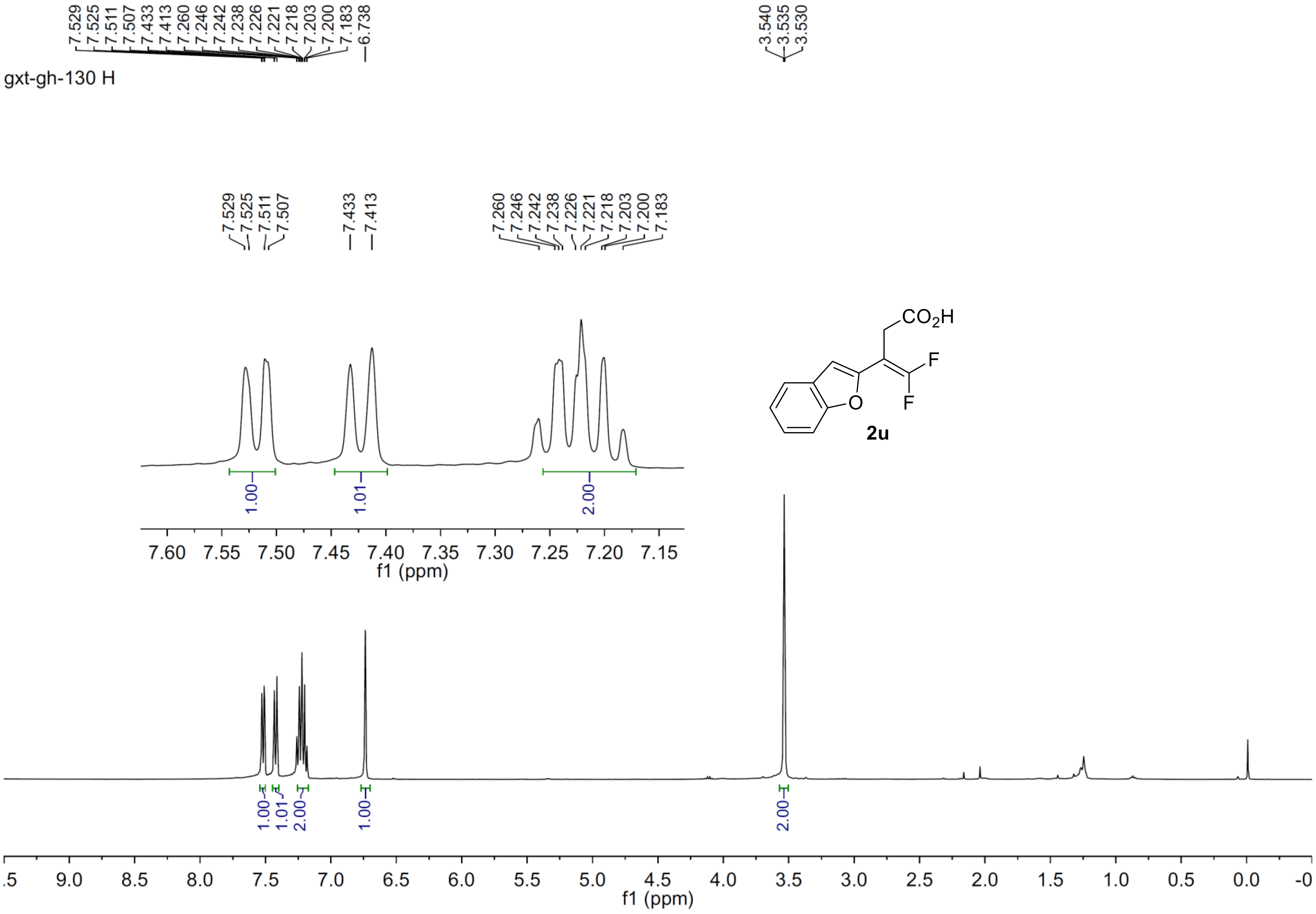

gxt-gh-130 H
7.529
7.525
7.511
7.507
7.433
7.413
7.260
7.246
7.242
7.238
7.226
7.221
7.218
7.203
7.200
7.183
6.738
3.540
3.535
3.530
CO_2H
F
F
O
2u
1.00
1.01
2.00
1.00
2.00
f1 (ppm)

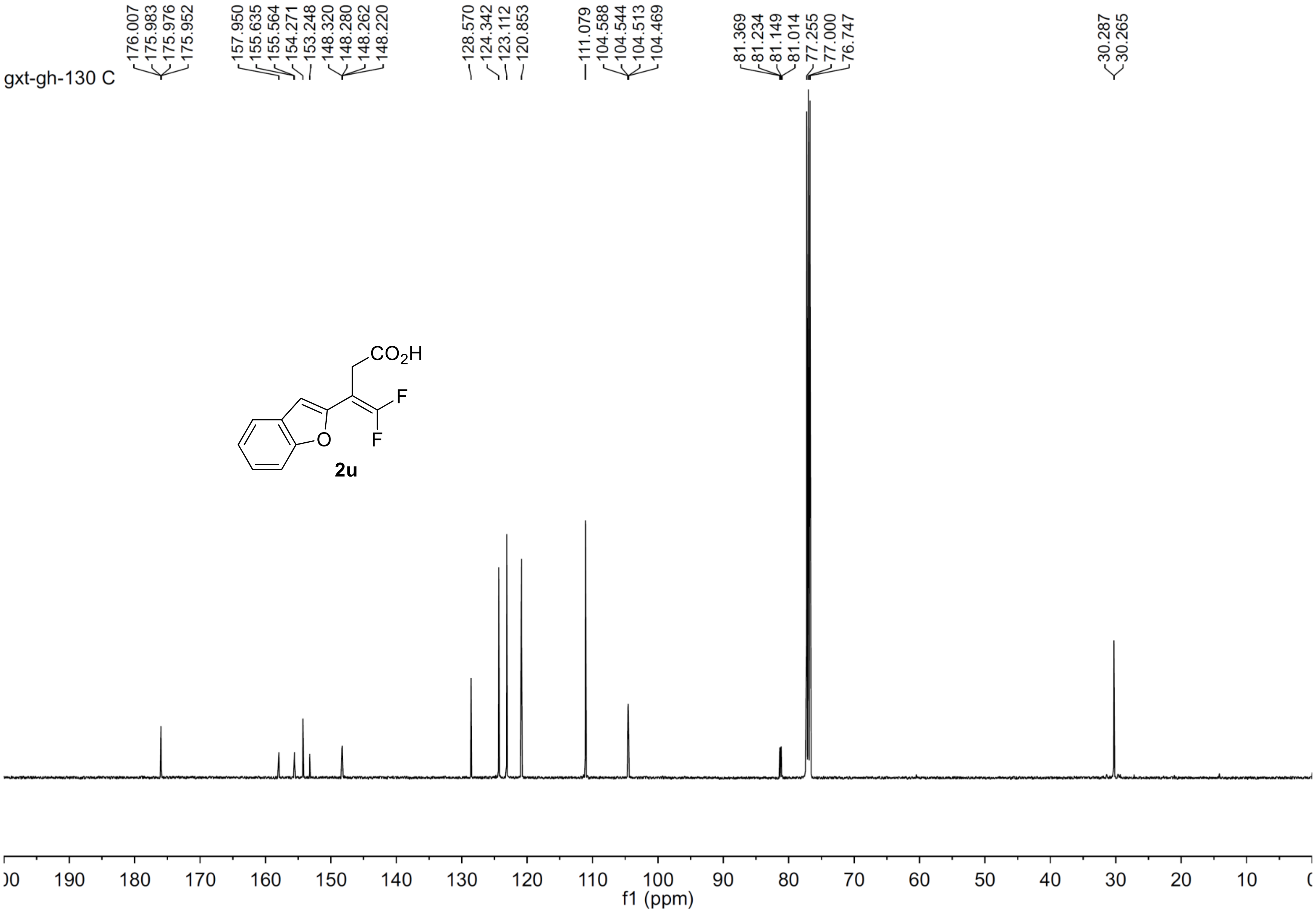
gxt-gh-130 C
176.007
175.983
175.976
175.952
157.950
155.635
155.564
154.271
153.248
148.320
148.280
148.262
148.220
128.570
124.342
123.112
120.853
111.079
104.588
104.544
104.513
104.469
81.369
81.234
81.149
81.014
77.255
77.000
76.747
30.287
30.265
CO2H
F
F
O
2u
190
180
170
160
150
140
130
120
110
100
90
80
70
60
50
40
30
20
10
f1 (ppm)

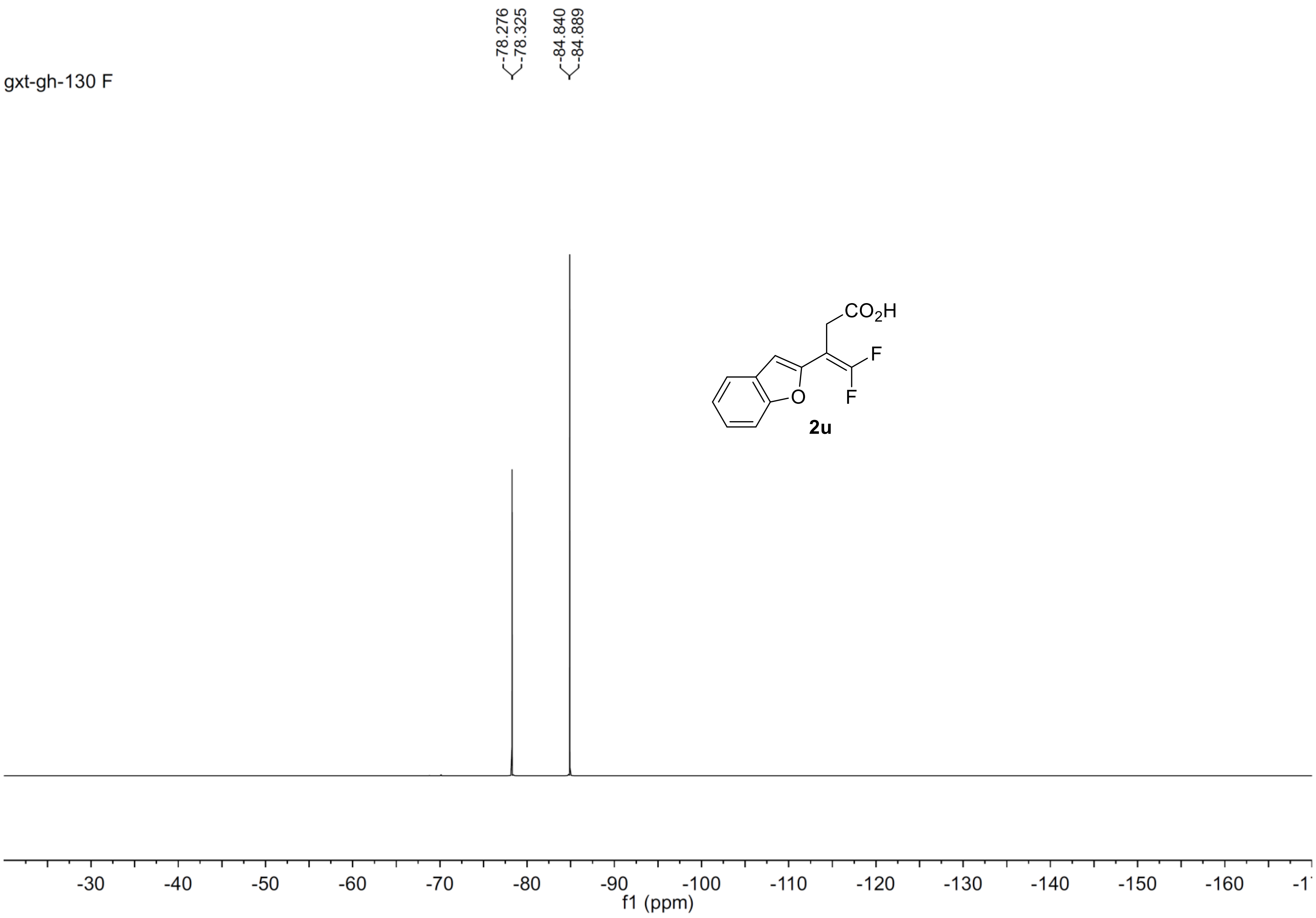
gxt-gh-130 F
-78.276
-78.325
-84.840
-84.889
CO2H
F
F
O
2u
-30
-40
-50
-60
-70
-80
-90
-100
-110
-120
-130
-140
-150
-160
f1 (ppm)

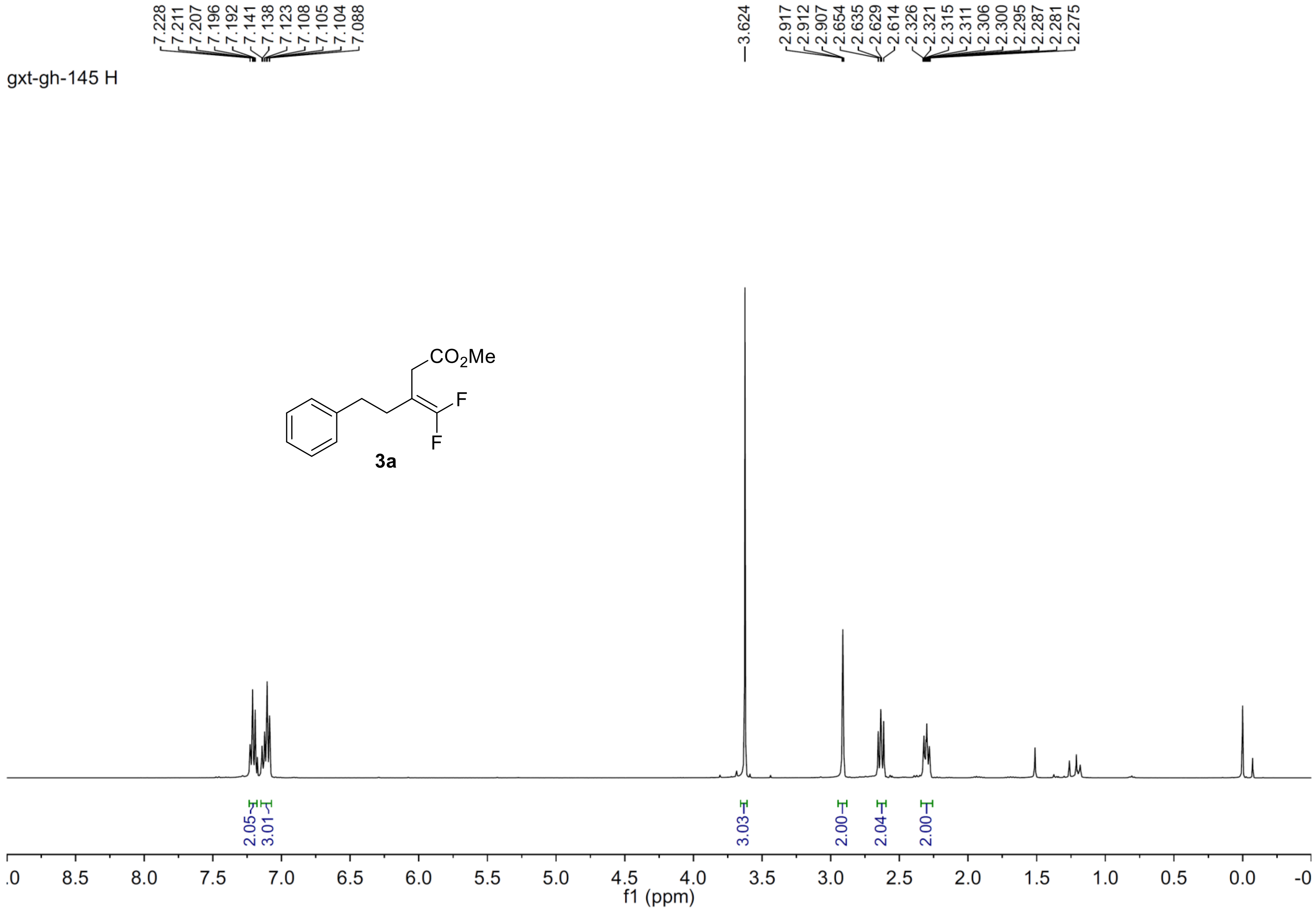
gxt-gh-145 H
7.228
7.211
7.207
7.196
7.192
7.141
7.138
7.123
7.108
7.105
7.104
7.088
3.624
2.917
2.912
2.907
2.654
2.635
2.629
2.614
2.326
2.321
2.315
2.311
2.306
2.300
2.295
2.287
2.281
2.275
CO2Me
F
F
3a
2.05
3.01
3.03
2.00
2.04
2.00
8.5
8.0
7.5
7.0
6.5
6.0
5.5
5.0
4.5
4.0
3.5
3.0
2.5
2.0
1.5
1.0
0.5
0.0
f1 (ppm)

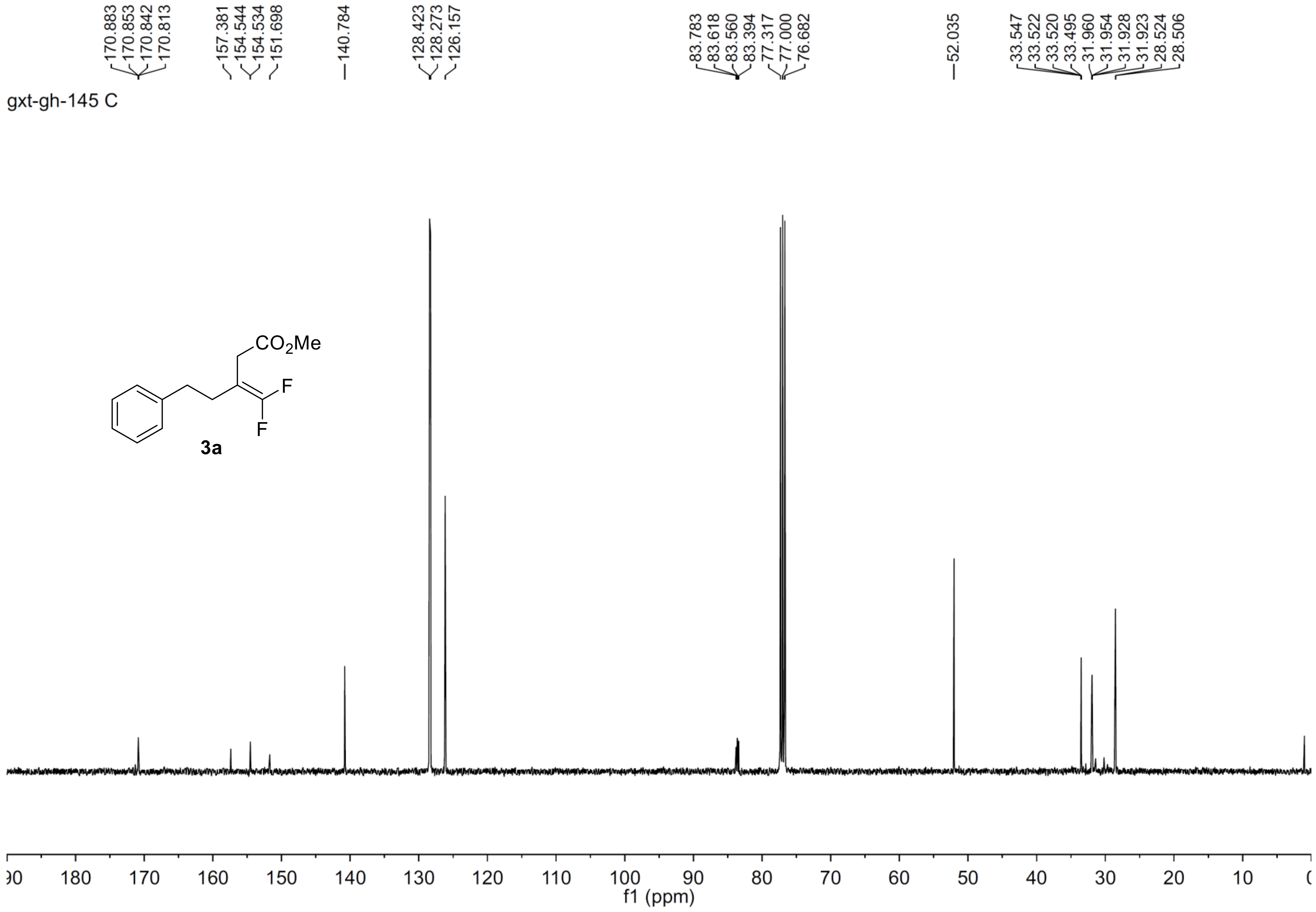
170.883
170.853
170.842
170.813
157.381
154.544
154.534
151.698
140.784
128.423
128.273
126.157
83.783
83.618
83.560
83.394
77.317
77.000
76.682
52.035
33.547
33.522
33.520
33.495
31.960
31.954
31.928
31.923
28.524
28.506
gxt-gh-145 C
CO2Me
F
F
3a
180 170 160 150 140 130 120 110 100 90 80 70 60 50 40 30 20 10
f1 (ppm)

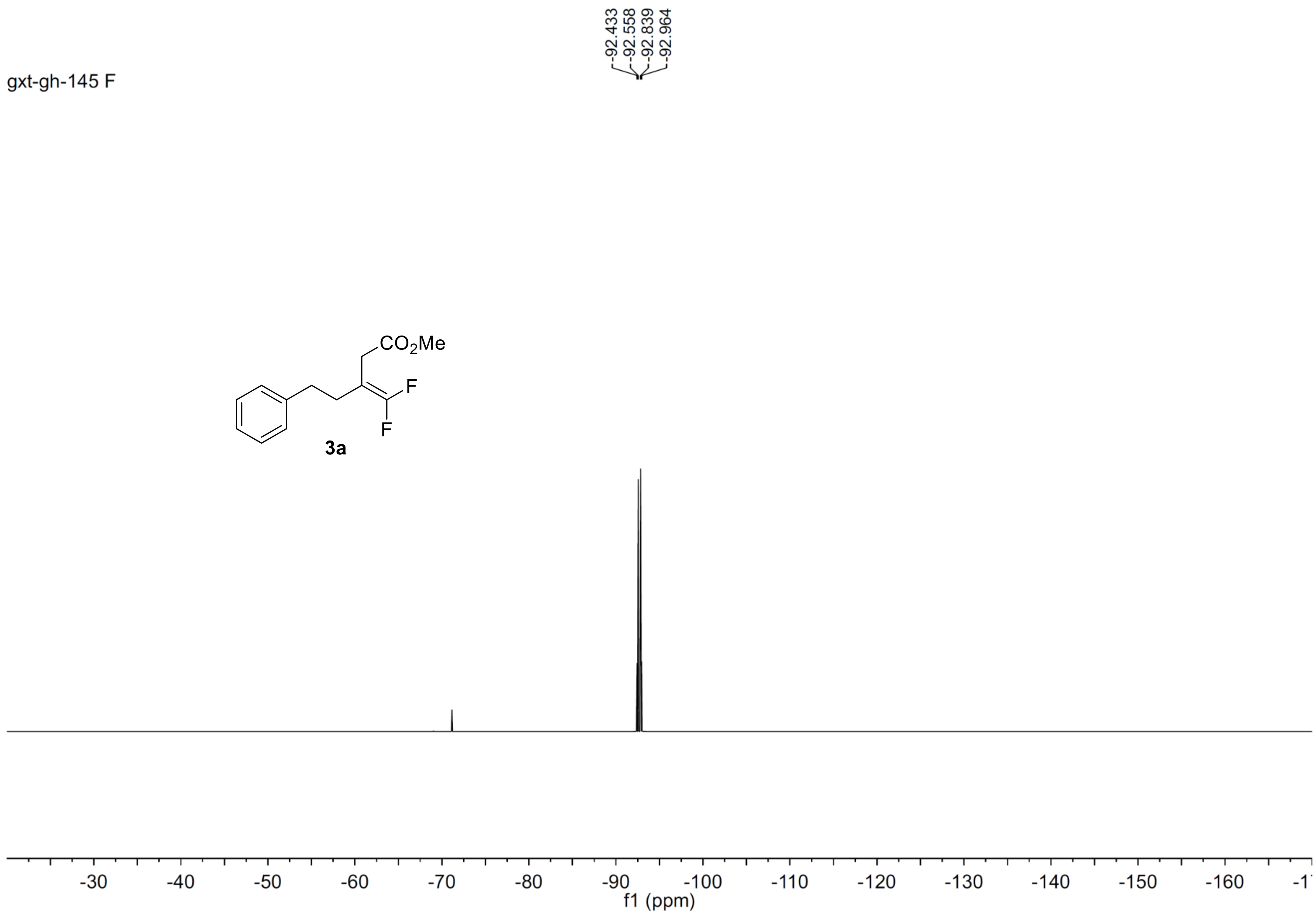
gxt-gh-145 F
-92.433
-92.558
-92.839
-92.964
CO2Me
F
F
3a
-30
-40
-50
-60
-70
-80
-90
-100
-110
-120
-130
-140
-150
-160
f1 (ppm)

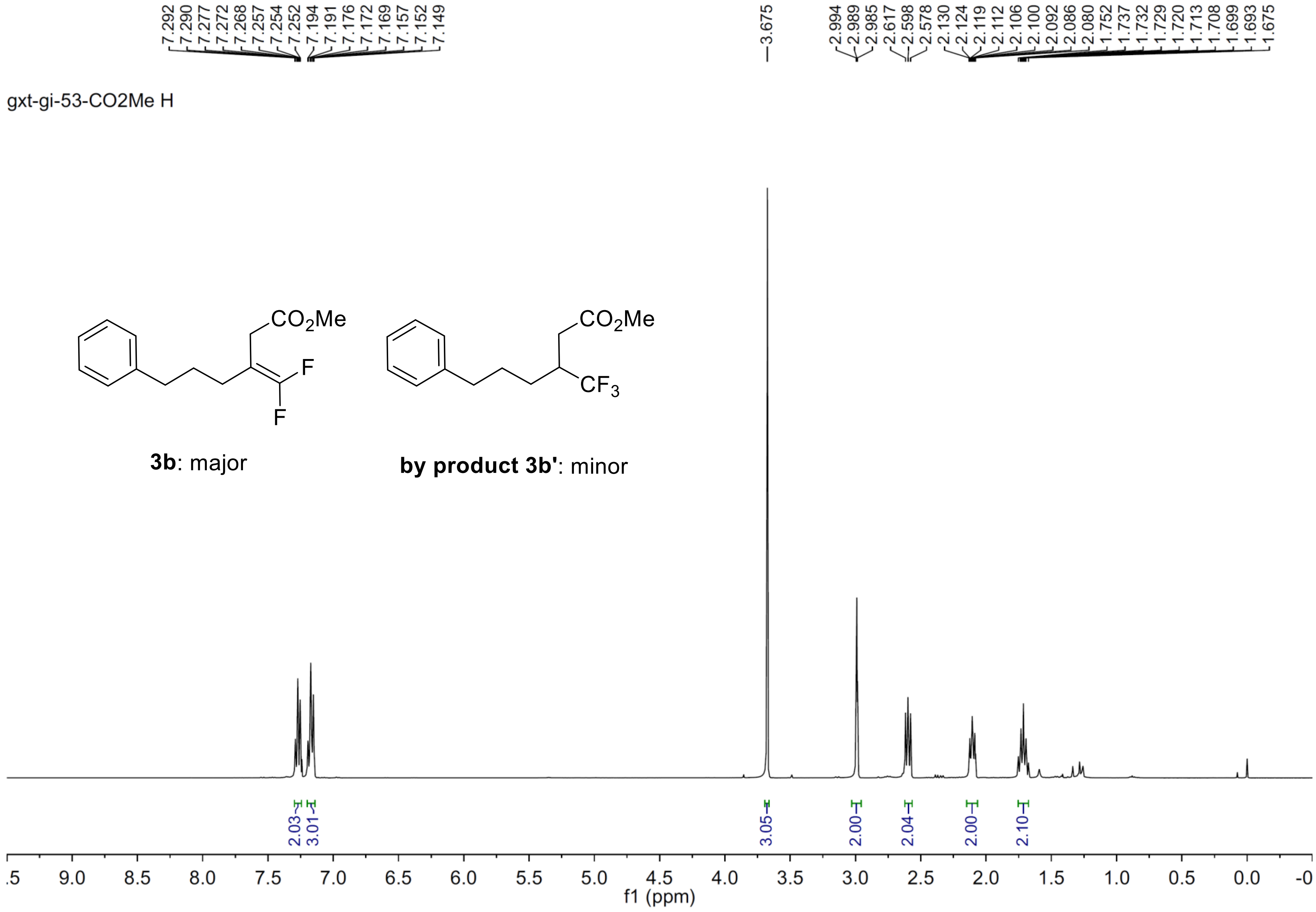
gxt-gi-53-CO2Me H
7.292 7.290 7.277 7.272 7.268 7.257 7.254 7.252 7.194 7.191 7.176 7.172 7.169 7.157 7.152 7.149
3.675
2.994 2.989 2.985 2.617 2.598 2.578 2.130 2.124 2.119 2.112 2.106 2.100 2.092 2.086 2.080 1.752 1.737 1.732 1.729 1.720 1.713 1.708 1.699 1.693 1.675
CO2Me
F
F
CF3
3b: major
by product 3b': minor
2.03
3.01
3.05
2.00
2.04
2.00
2.10
f1 (ppm)

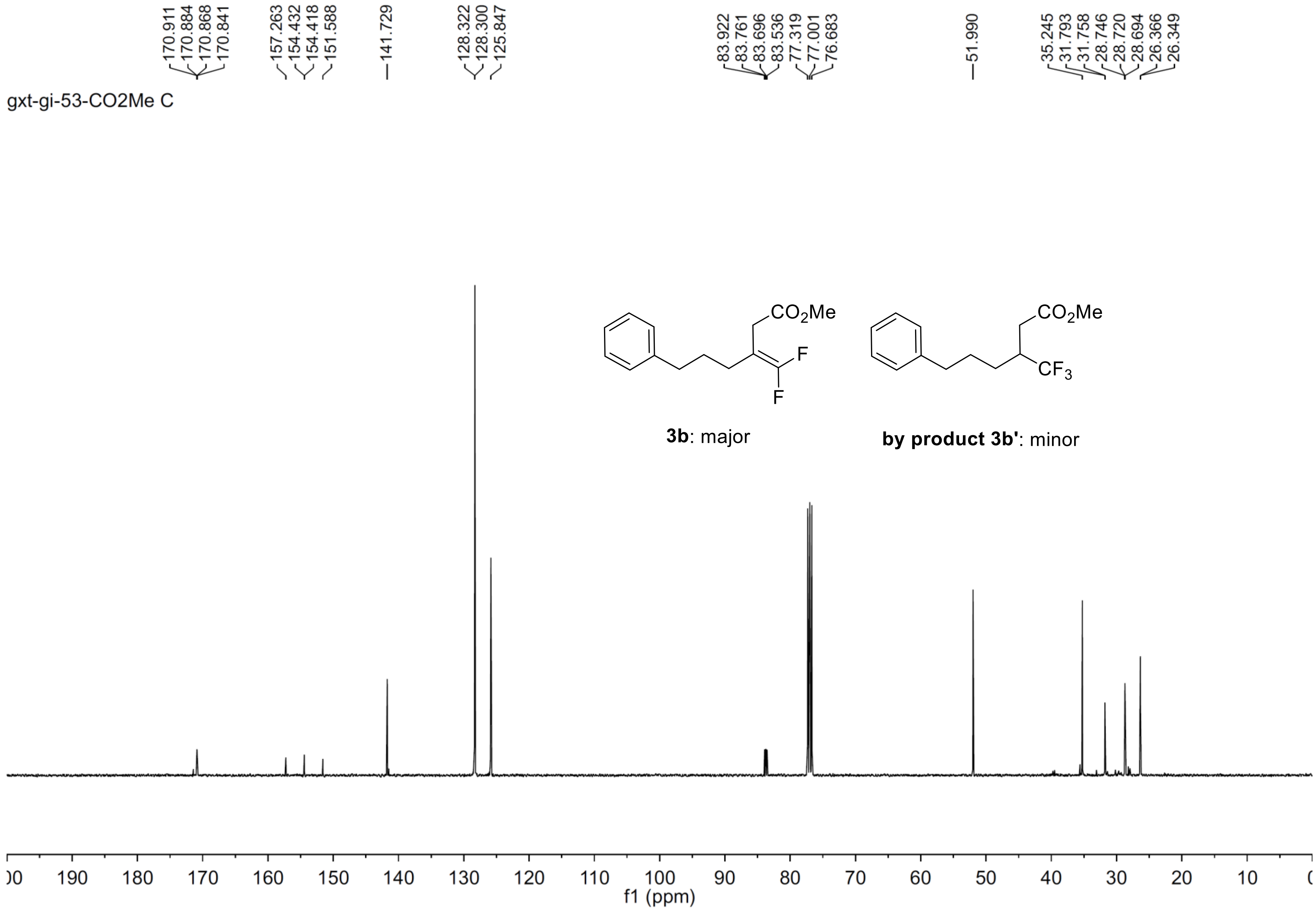
gxt-gi-53-CO2Me C
170.911
170.884
170.868
170.841
157.263
154.432
154.418
151.588
141.729
128.322
128.300
125.847
83.922
83.761
83.696
83.536
77.319
77.001
76.683
51.990
35.245
31.793
31.758
28.746
28.720
28.694
26.366
26.349
CO_2Me
F
F
3b: major
CO_2Me
CF_3
by product 3b': minor
190
180
170
160
150
140
130
120
110
100
90
80
70
60
50
40
30
20
10
f1 (ppm)

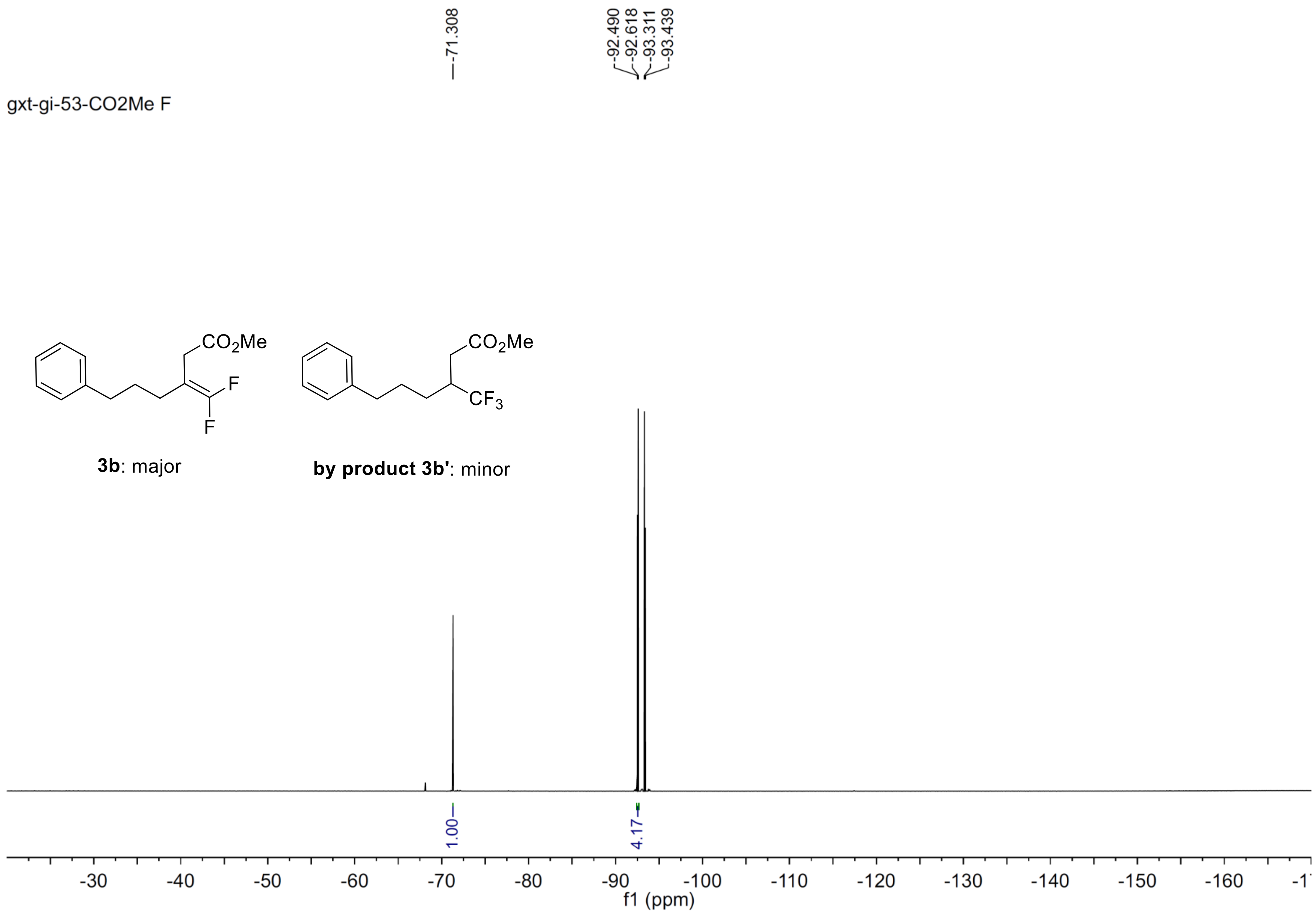
gxt-gi-53-CO2Me F
-71.308
-92.490
-92.618
-93.311
-93.439
CO2Me
F
F
CO2Me
CF3
3b: major
by product 3b': minor
1.00
4.17
-30
-40
-50
-60
-70
-80
-90
-100
-110
-120
-130
-140
-150
-160
f1 (ppm)

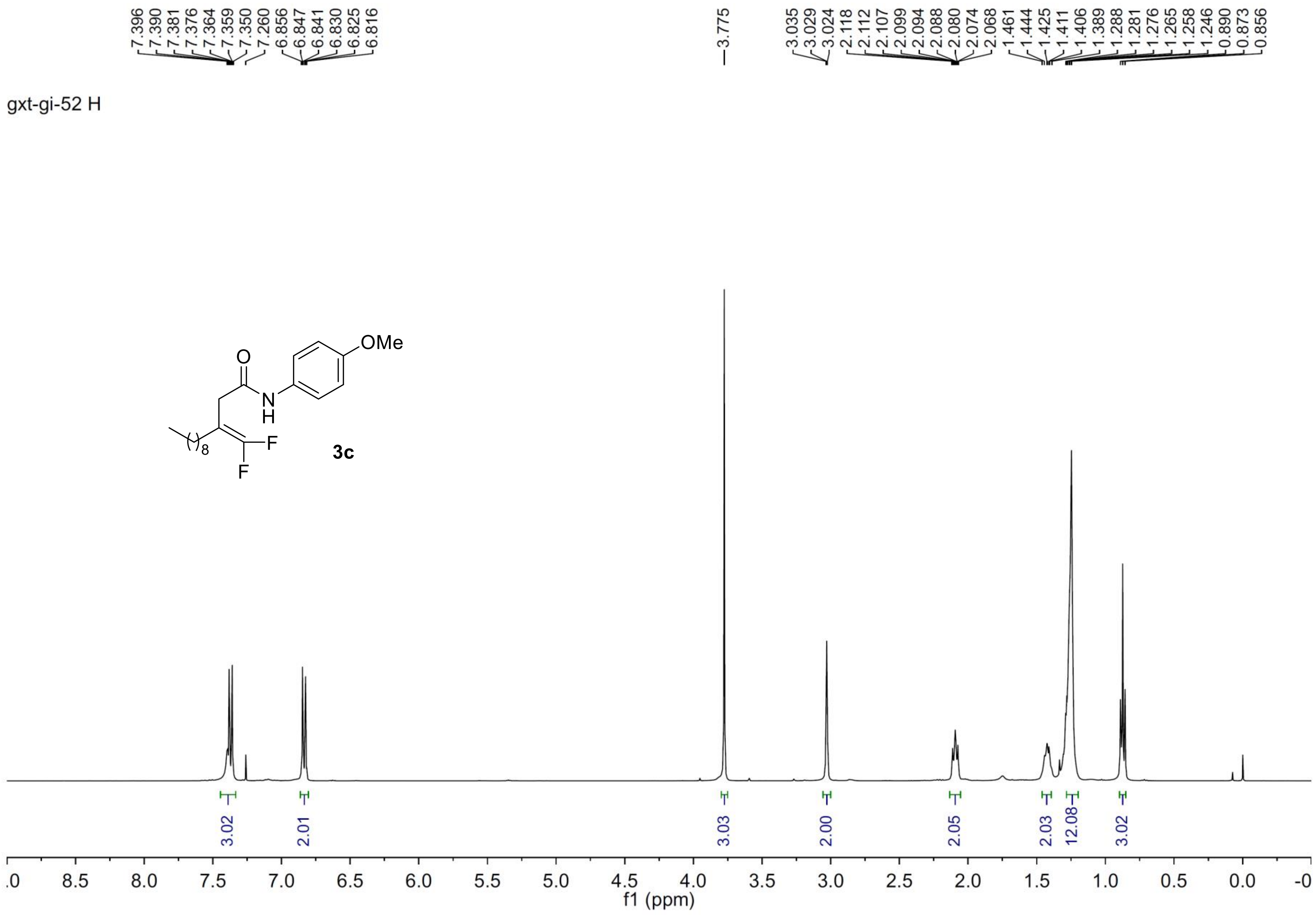
7.396
7.390
7.381
7.376
7.364
7.359
7.350
7.260
6.856
6.847
6.841
6.830
6.825
6.816
3.775
3.035
3.029
3.024
2.118
2.112
2.107
2.099
2.094
2.088
2.080
2.074
2.068
1.461
1.444
1.425
1.411
1.406
1.389
1.288
1.281
1.276
1.265
1.258
1.246
0.890
0.873
0.856
gxt-gi-52 H
OMe
O
N
H
F
F
8
3c
3.02
2.01
3.03
2.00
2.05
2.03
12.08
3.02
f1 (ppm)

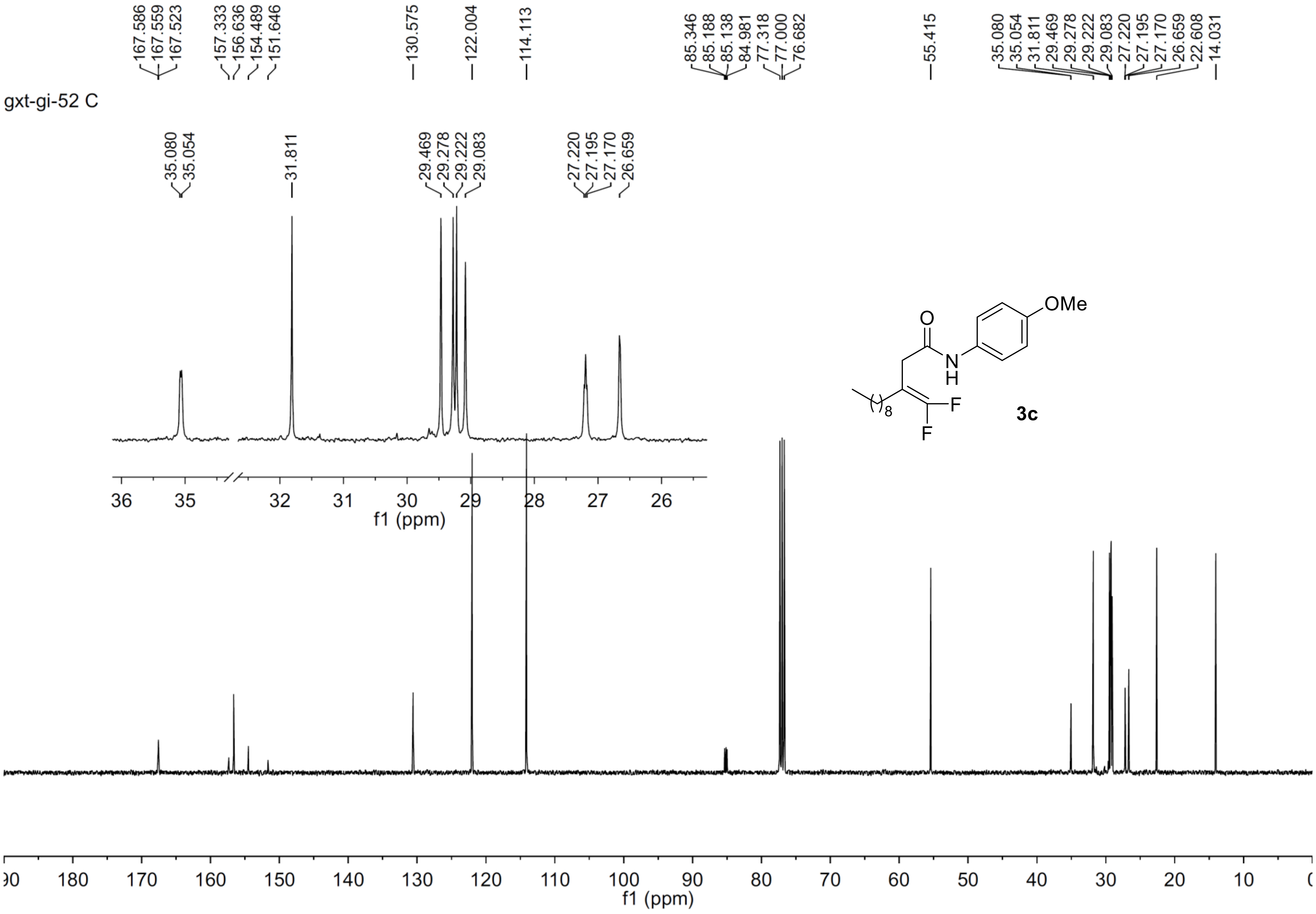
gxt-gi-52 C
167.586
167.559
167.523
157.333
156.636
154.489
151.646
130.575
122.004
114.113
85.346
85.188
85.138
84.981
77.318
77.000
76.682
55.415
35.080
35.054
31.811
29.469
29.278
29.222
29.083
27.220
27.195
27.170
26.659
22.608
14.031
OMe
O
N
H
F
F
8
3c
f1 (ppm)

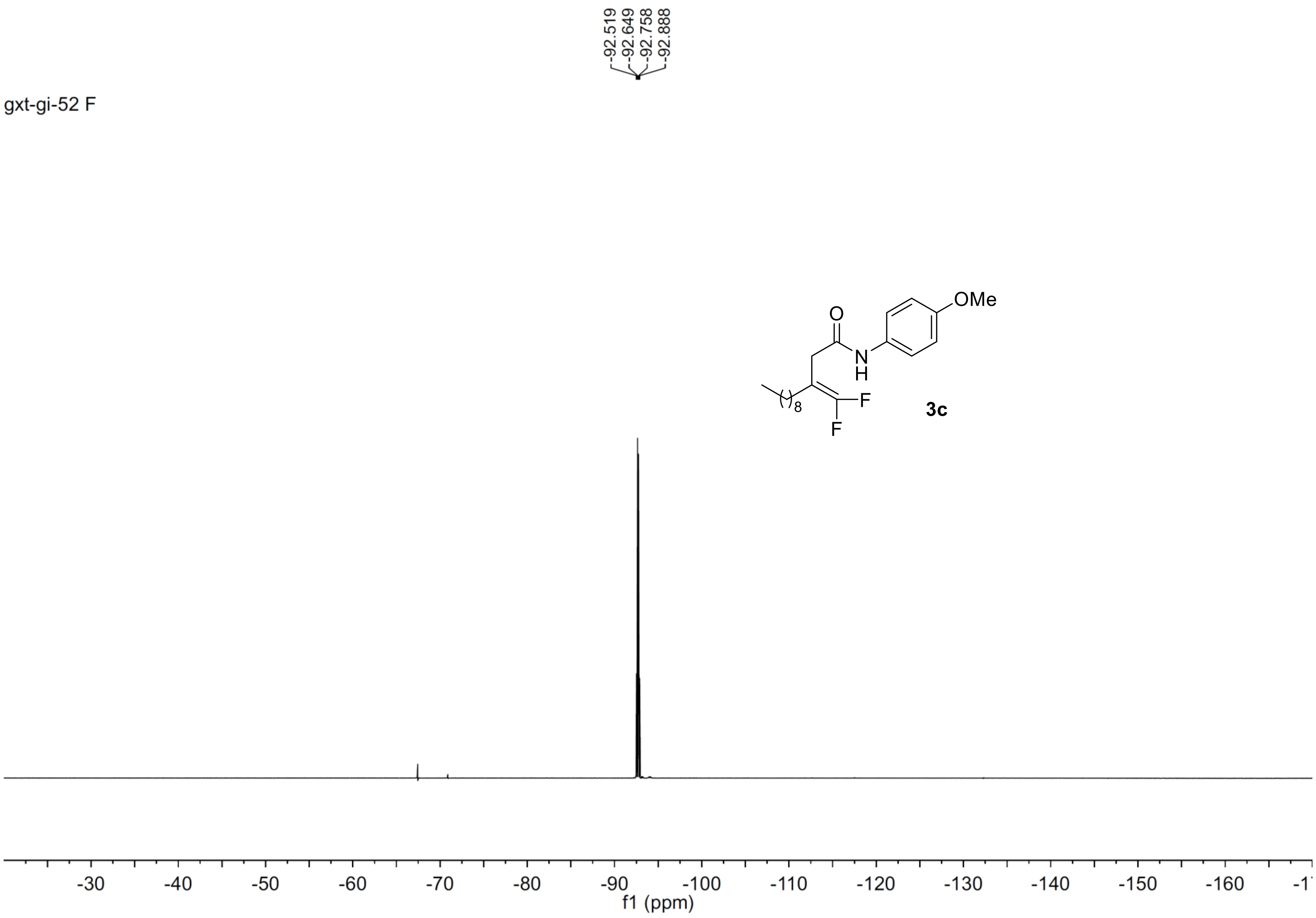
-92.519
-92.649
-92.758
-92.888
gxt-gi-52 F
OMe
O
N
H
8
F
F
3c
-30
-40
-50
-60
-70
-80
-90
-100
-110
-120
-130
-140
-150
-160
f1 (ppm)

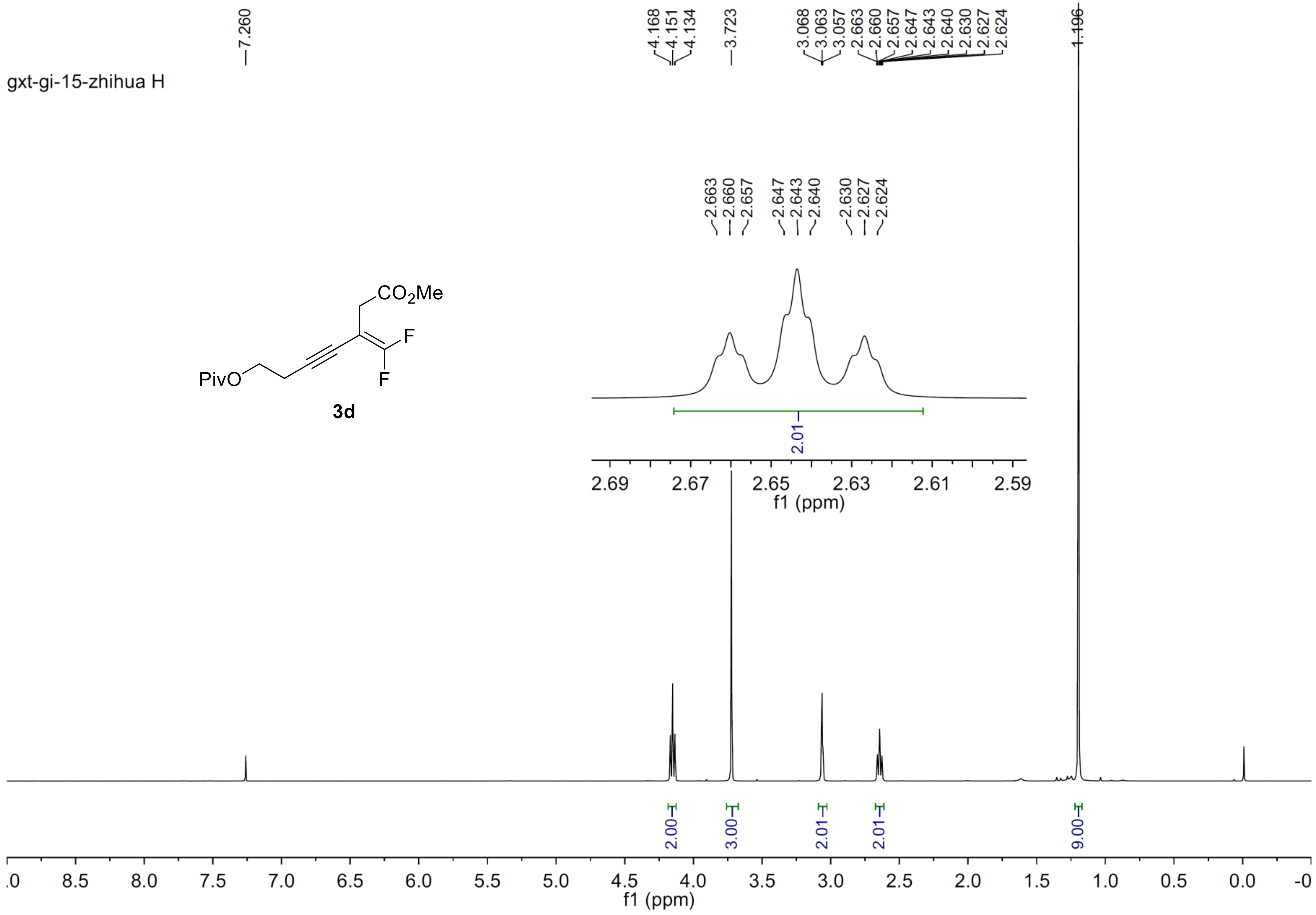
gxt-gi-15-zhihua H
7.260
4.168
4.151
4.134
3.723
3.068
3.063
3.057
2.663
2.660
2.657
2.647
2.643
2.640
2.630
2.627
2.624
1.196
CO2Me
F
F
PivO
3d
2.01
2.69 2.67 2.65 2.63 2.61 2.59
f1 (ppm)
2.00
3.00
2.01
2.01
9.00
9.0 8.5 8.0 7.5 7.0 6.5 6.0 5.5 5.0 4.5 4.0 3.5 3.0 2.5 2.0 1.5 1.0 0.5 0.0 -0
f1 (ppm)

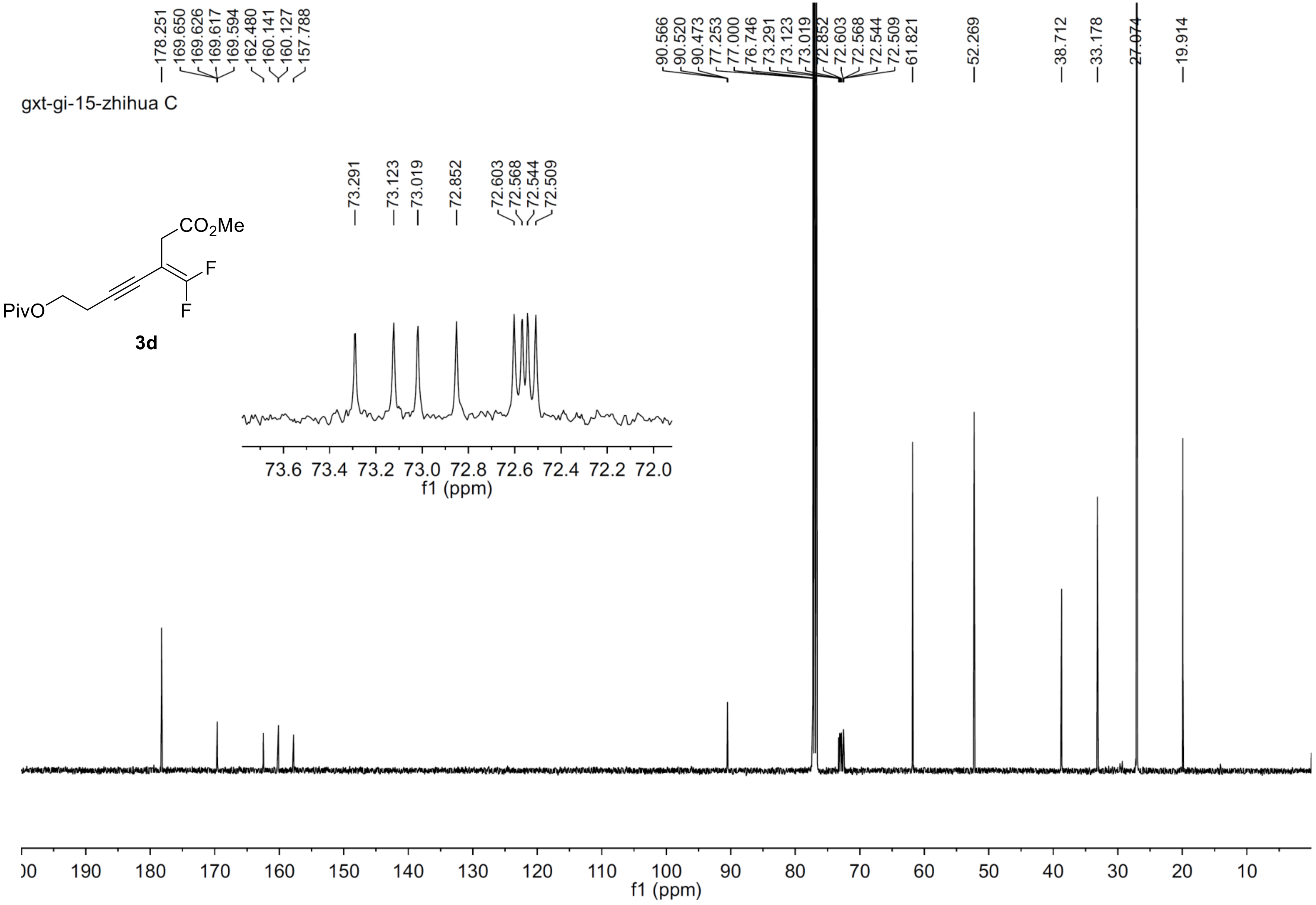
178.251
169.650
169.626
169.617
169.594
162.480
160.141
160.127
157.788
gxt-gi-15-zhihua C
90.566
90.520
90.473
77.253
77.000
76.746
73.291
73.123
73.019
72.852
72.603
72.568
72.544
72.509
61.821
52.269
38.712
33.178
27.074
19.914
CO2Me
F
F
PivO
3d
73.6 73.4 73.2 73.0 72.8 72.6 72.4 72.2 72.0
f1 (ppm)
190 180 170 160 150 140 130 120 110 100 90 80 70 60 50 40 30 20 10
f1 (ppm)

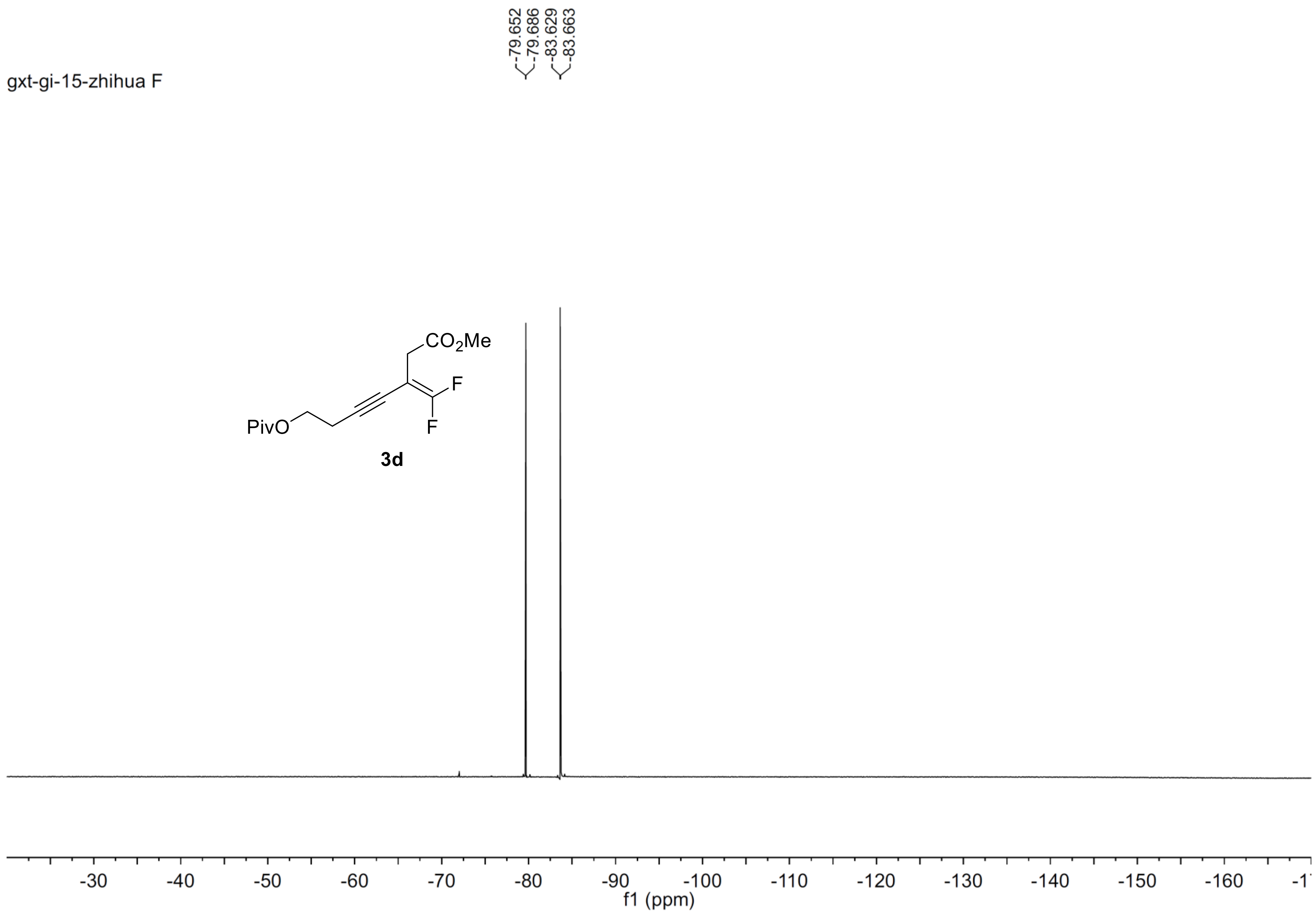
gxt-gi-15-zhihua F
-79.652
-79.686
-83.629
-83.663
CO2Me
F
F
PivO
3d
-30 -40 -50 -60 -70 -80 -90 -100 -110 -120 -130 -140 -150 -160
f1 (ppm)

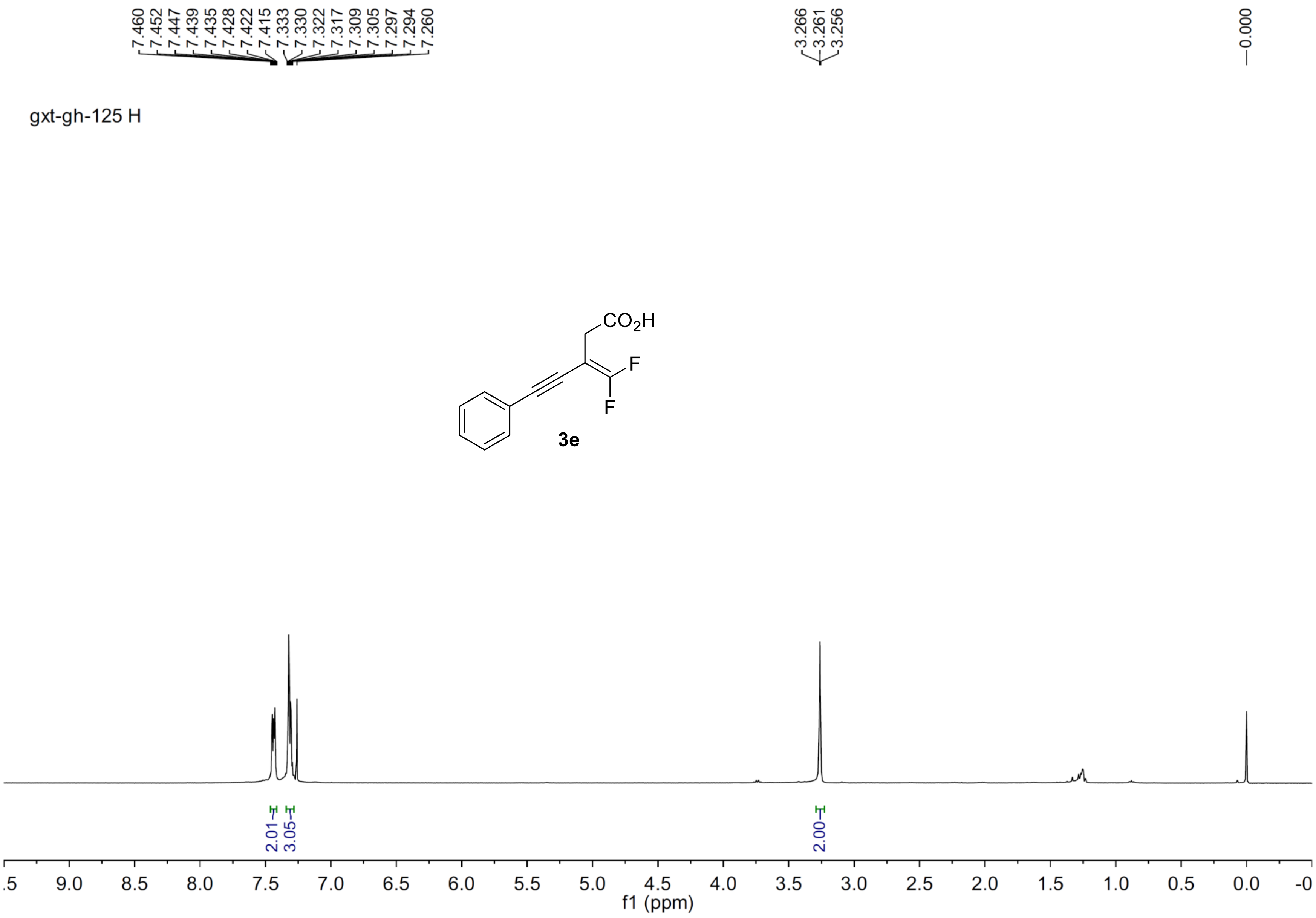
7.460
7.452
7.447
7.439
7.435
7.428
7.422
7.415
7.333
7.330
7.322
7.317
7.309
7.305
7.297
7.294
7.260
3.266
3.261
3.256
0.000
gxt-gh-125 H
CO2H
F
F
3e
2.01
3.05
2.00
.5 9.0 8.5 8.0 7.5 7.0 6.5 6.0 5.5 5.0 4.5 4.0 3.5 3.0 2.5 2.0 1.5 1.0 0.5 0.0 -0
f1 (ppm)

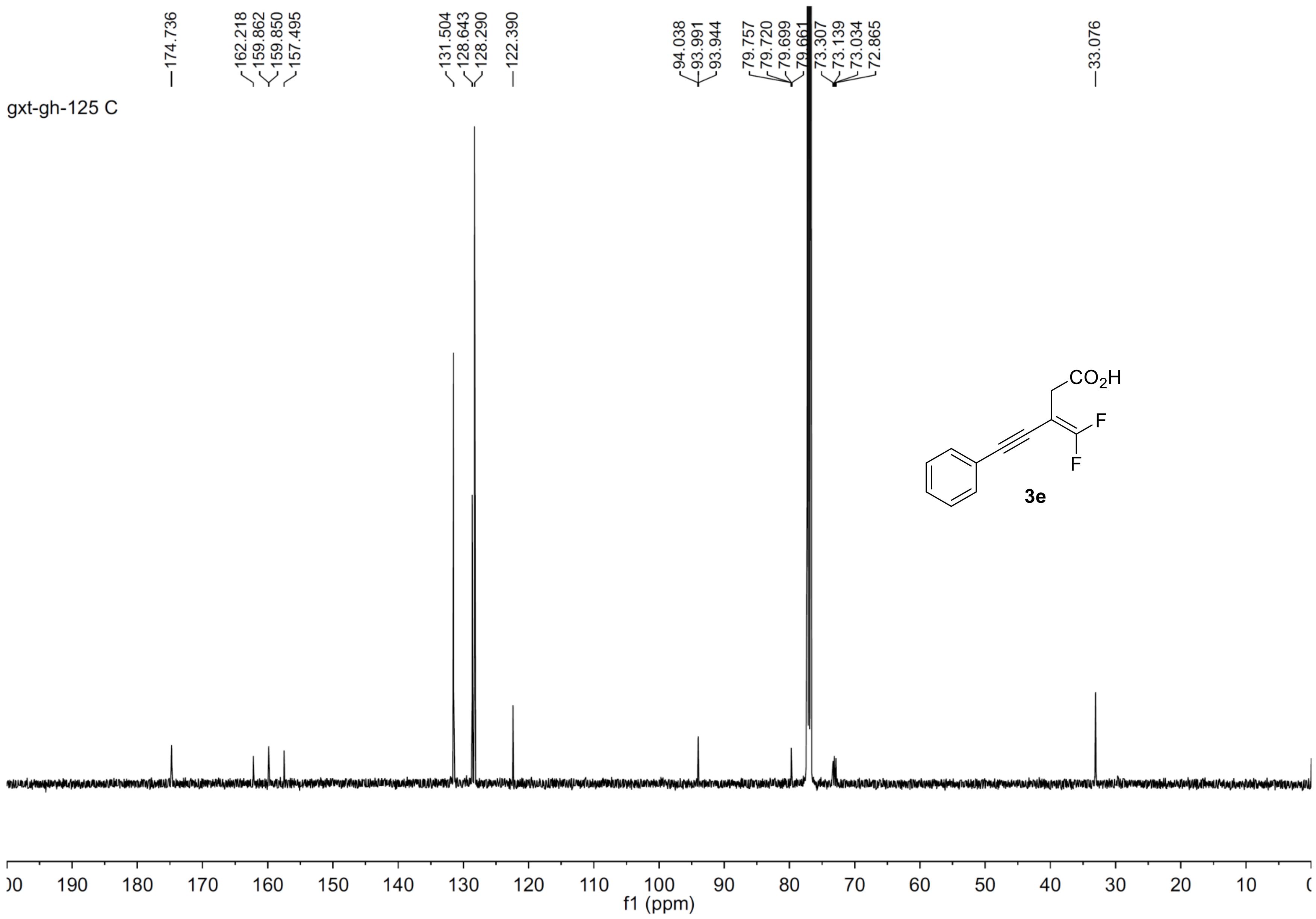

gxt-gh-125 C
174.736
162.218
159.862
159.850
157.495
131.504
128.643
128.290
122.390
94.038
93.991
93.944
79.757
79.720
79.699
79.661
73.307
73.139
73.034
72.865
33.076
CO_2H
F
F
3e
190
180
170
160
150
140
130
120
110
100
90
80
70
60
50
40
30
20
10
f1 (ppm)

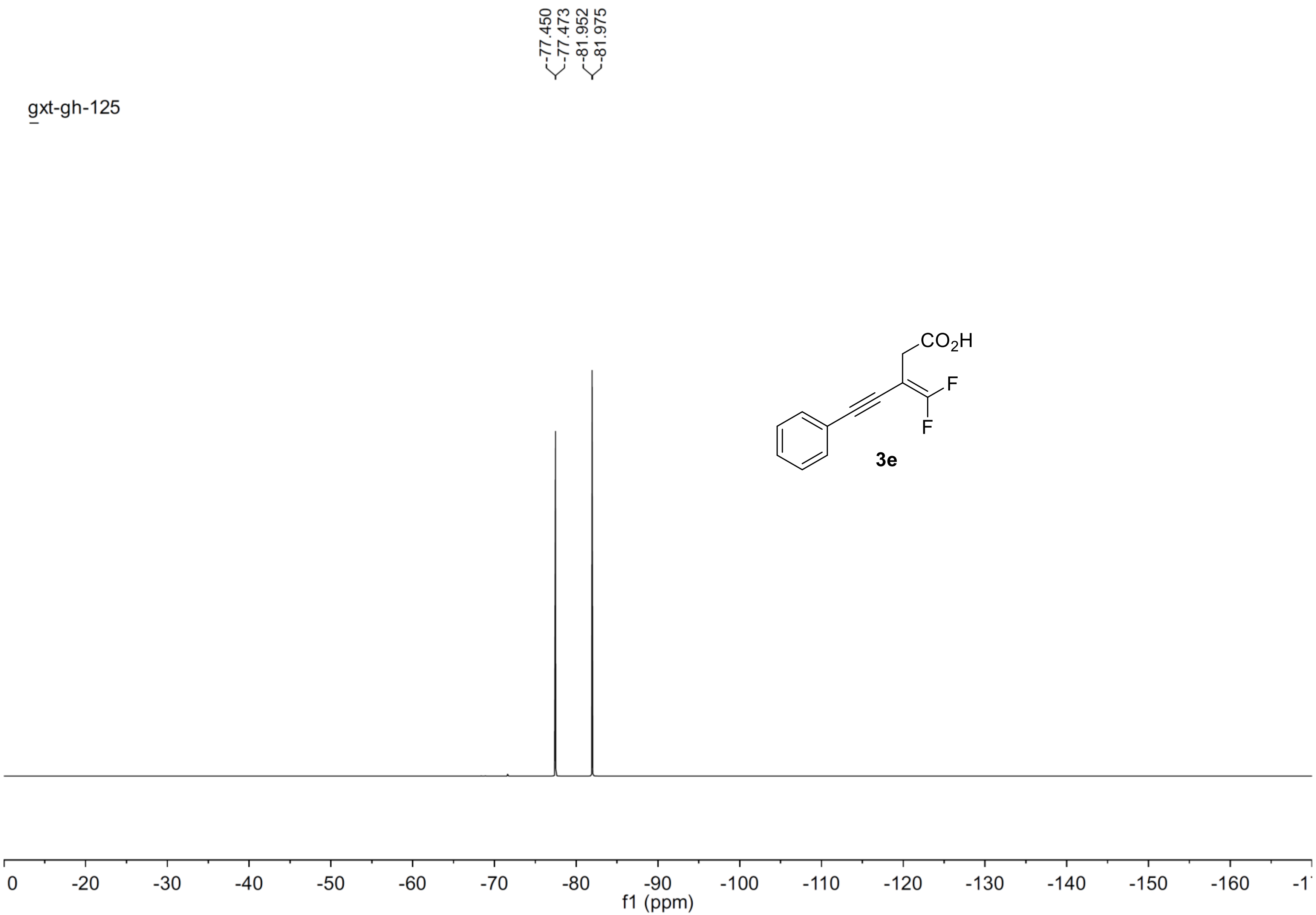
-77.450
-77.473
-81.952
-81.975
gxt-gh-125
CO_2H
F
F
3e
0
-20
-30
-40
-50
-60
-70
-80
-90
-100
-110
-120
-130
-140
-150
-160
f1 (ppm)

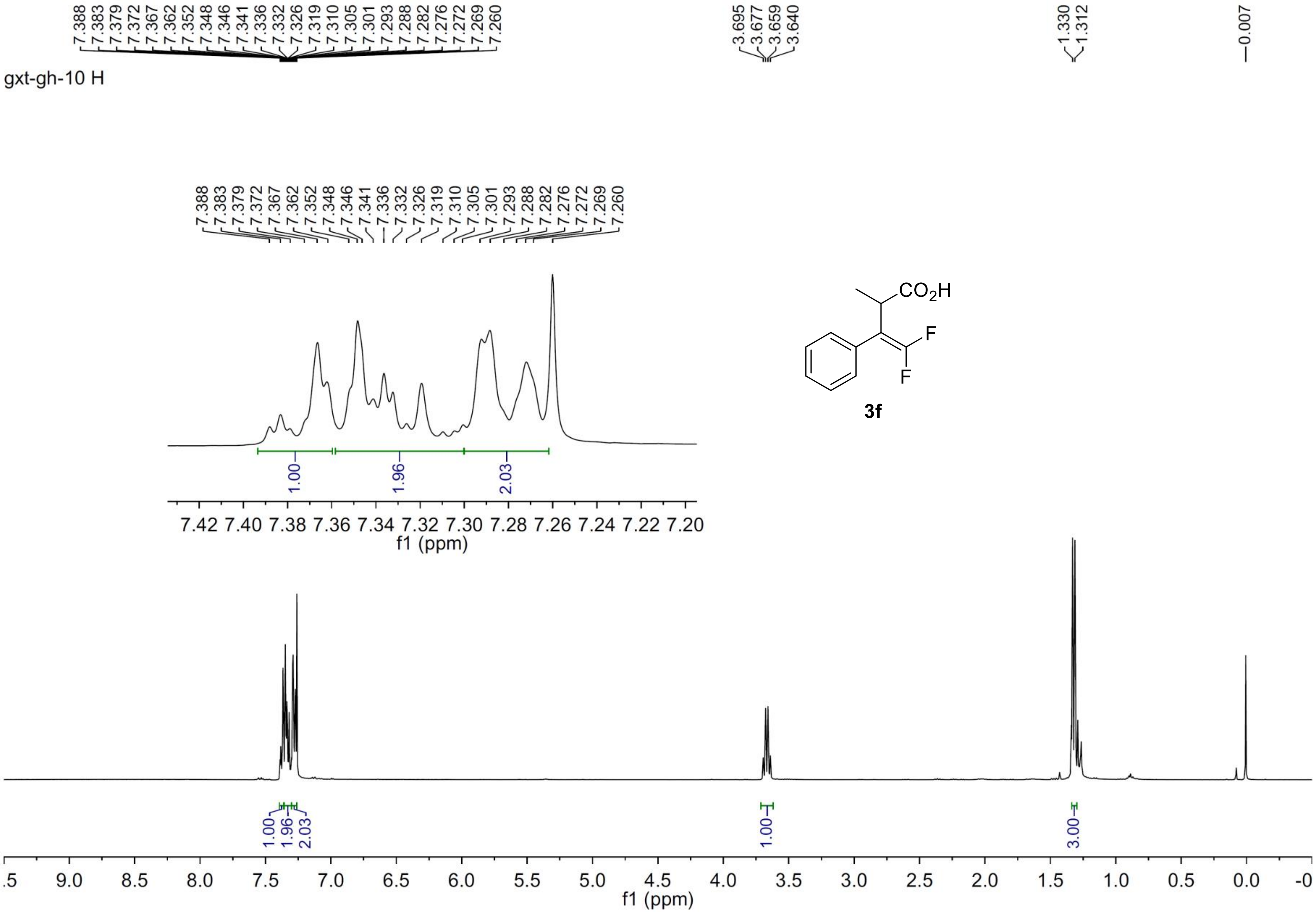
gxt-gh-10 H
7.388 7.383 7.379 7.372 7.367 7.362 7.352 7.348 7.346 7.341 7.336 7.332 7.326 7.319 7.310 7.305 7.301 7.293 7.288 7.282 7.276 7.272 7.269 7.260
3.695 3.677 3.659 3.640
1.330 1.312
0.007
1.00 1.96 2.03
7.42 7.40 7.38 7.36 7.34 7.32 7.30 7.28 7.26 7.24 7.22 7.20
f1 (ppm)
CO_2H
F
F
3f
1.00 1.96 2.03 1.00 3.00
.5 9.0 8.5 8.0 7.5 7.0 6.5 6.0 5.5 5.0 4.5 4.0 3.5 3.0 2.5 2.0 1.5 1.0 0.5 0.0 -0
f1 (ppm)

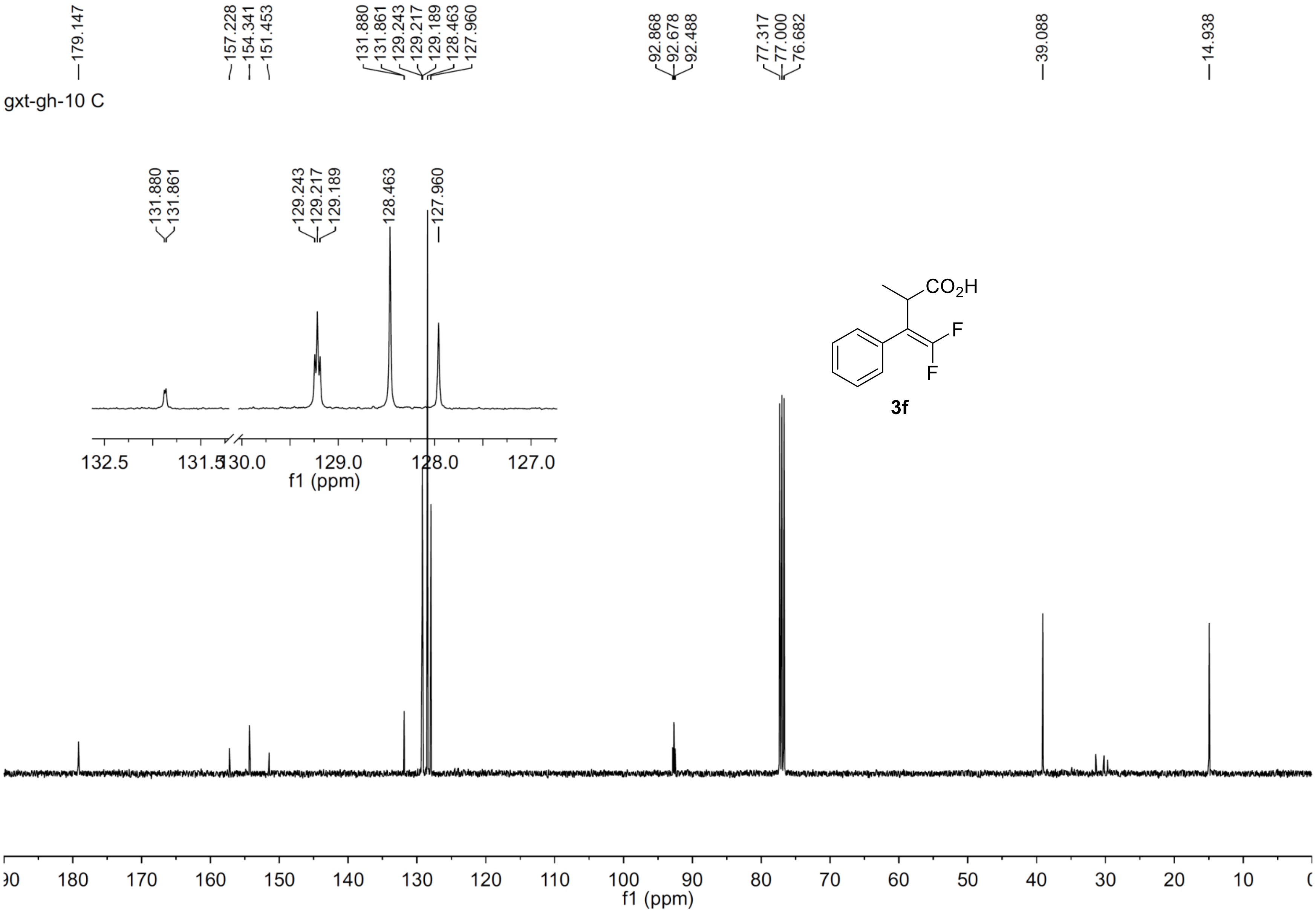

179.147
157.228
154.341
151.453
131.880
131.861
129.243
129.217
129.189
128.463
127.960
92.868
92.678
92.488
77.317
77.000
76.682
39.088
14.938
gxt-gh-10 C
CO2H
F
F
3f
132.5
131.5
130.0
129.0
128.0
127.0
f1 (ppm)
180
170
160
150
140
130
120
110
100
90
80
70
60
50
40
30
20
10

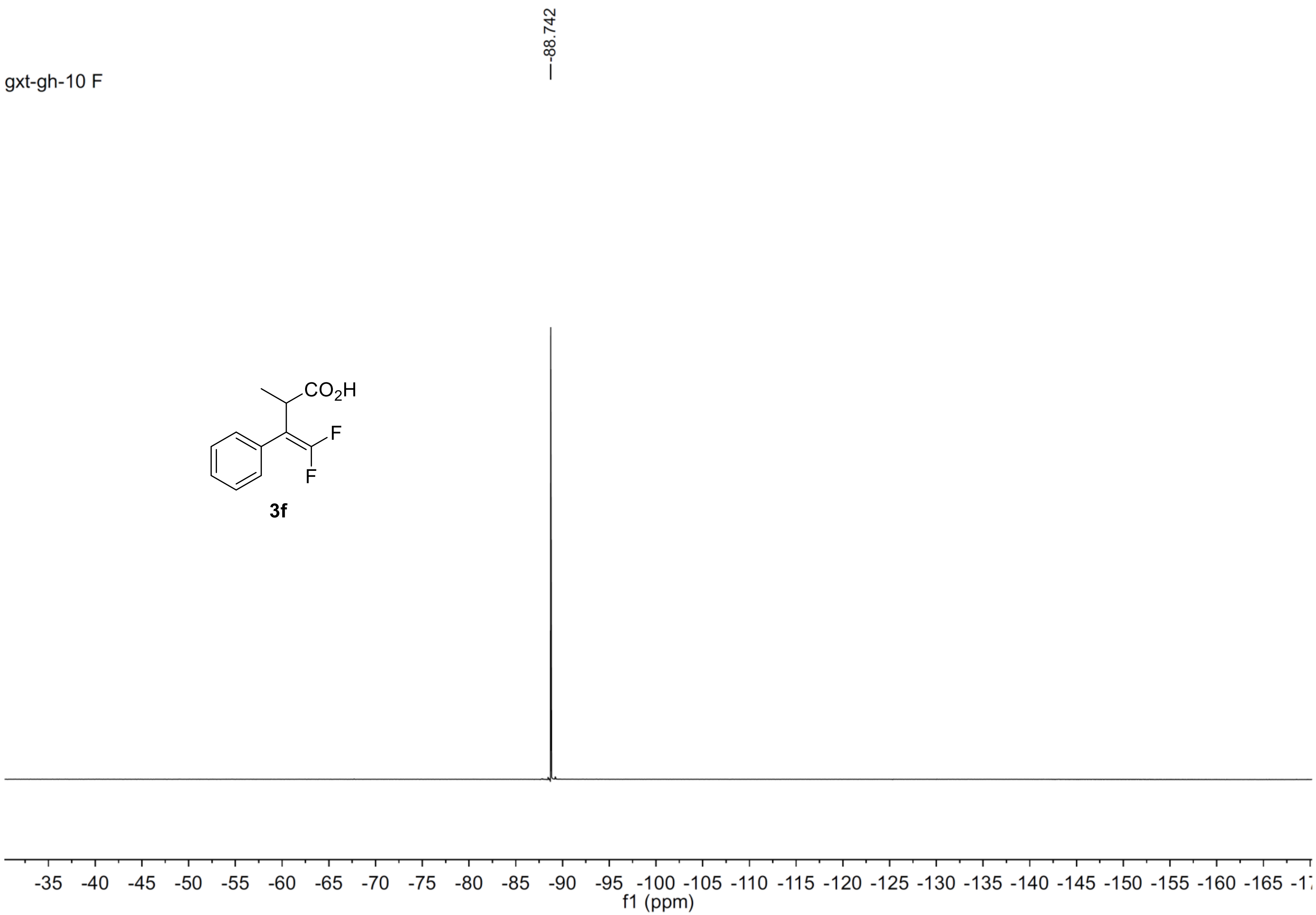
gxt-gh-10 F
-88.742
CO2H
F
F
3f
-35 -40 -45 -50 -55 -60 -65 -70 -75 -80 -85 -90 -95 -100 -105 -110 -115 -120 -125 -130 -135 -140 -145 -150 -155 -160 -165
f1 (ppm)

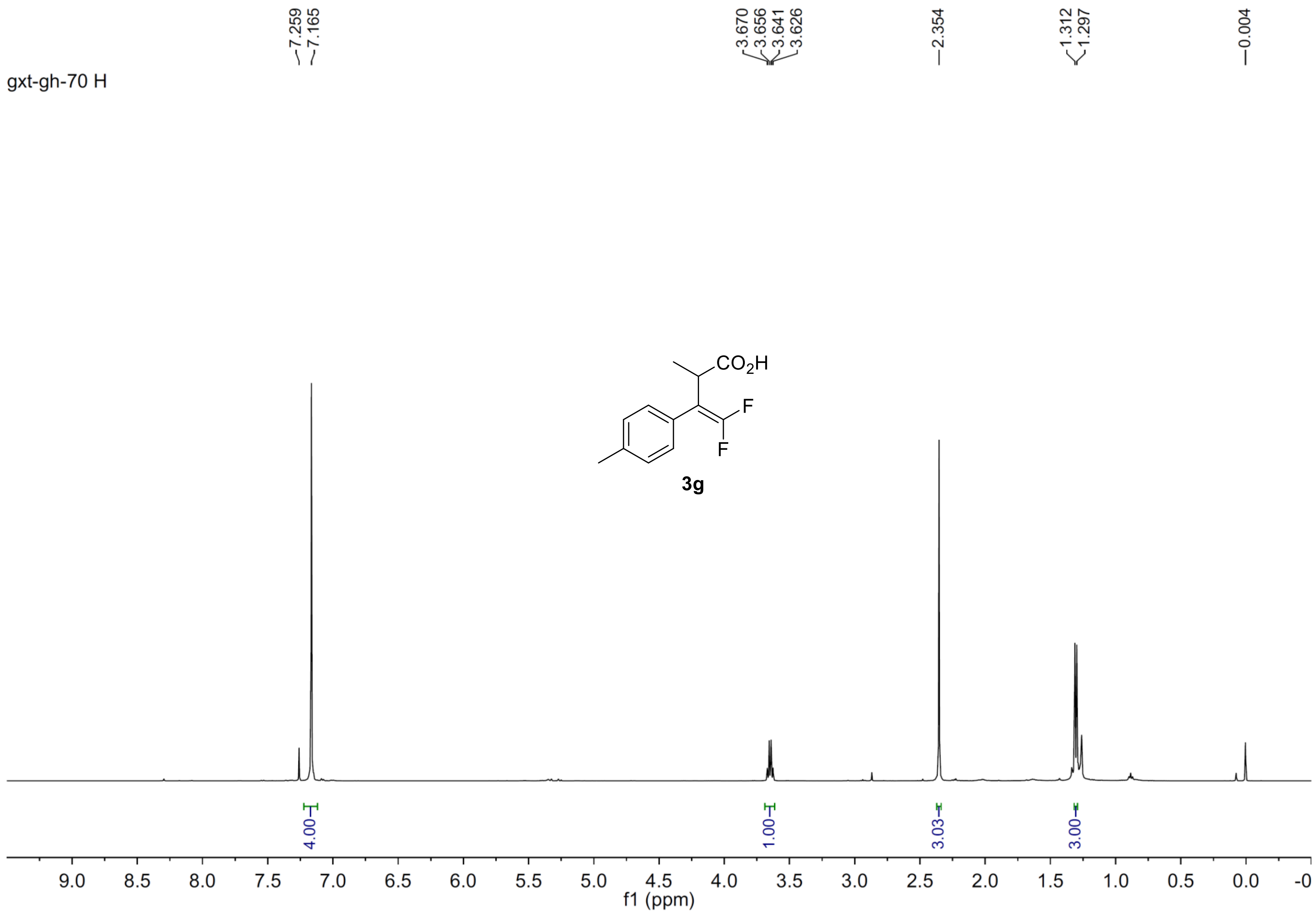
gxt-gh-70 H
7.259
7.165
3.670
3.656
3.641
3.626
2.354
1.312
1.297
0.004
CO2H
F
F
3g
4.00
1.00
3.03
3.00
9.0 8.5 8.0 7.5 7.0 6.5 6.0 5.5 5.0 4.5 4.0 3.5 3.0 2.5 2.0 1.5 1.0 0.5 0.0 -0
f1 (ppm)

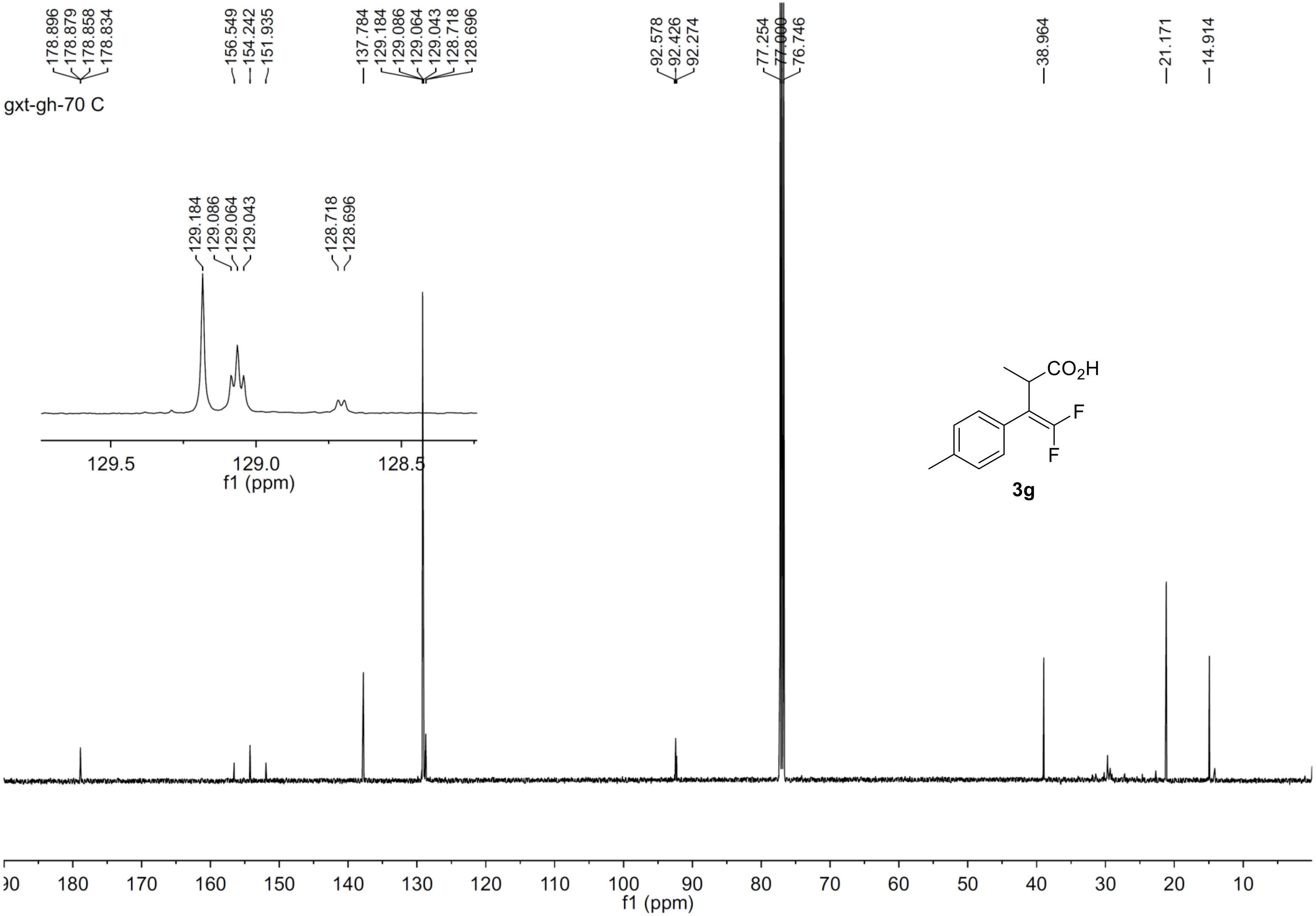
178.896
178.879
178.858
178.834
156.549
154.242
151.935
137.784
129.184
129.086
129.064
129.043
128.718
128.696
92.578
92.426
92.274
77.254
77.000
76.746
38.964
21.171
14.914
gxt-gh-70 C
129.5
129.0
128.5
f1 (ppm)
CO2H
F
F
3g
180
170
160
150
140
130
120
110
100
90
80
70
60
50
40
30
20
10
f1 (ppm)

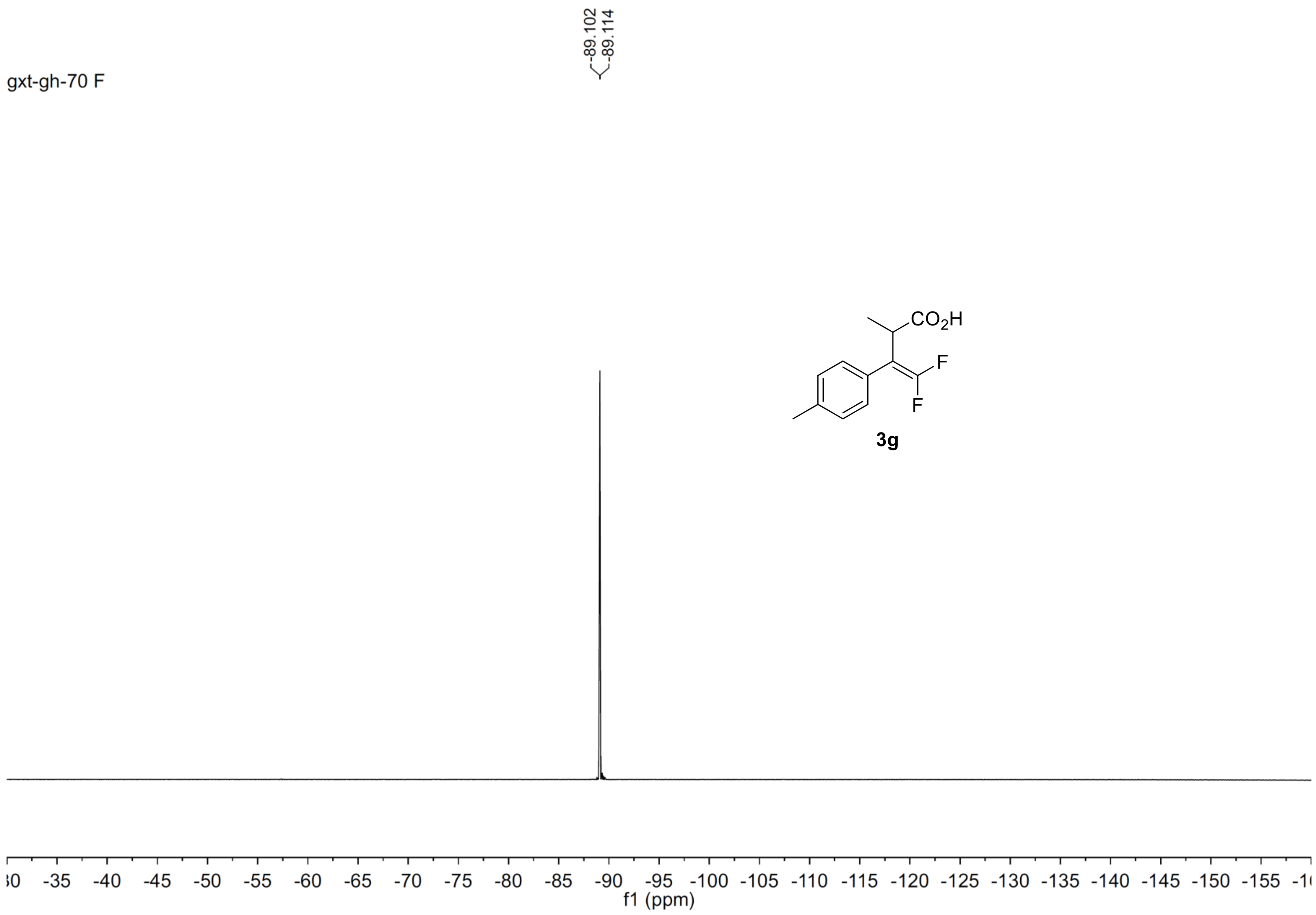
gxt-gh-70 F
-89.102
-89.114
CO2H
F
F
3g
-35 -40 -45 -50 -55 -60 -65 -70 -75 -80 -85 -90 -95 -100 -105 -110 -115 -120 -125 -130 -135 -140 -145 -150 -155
f1 (ppm)

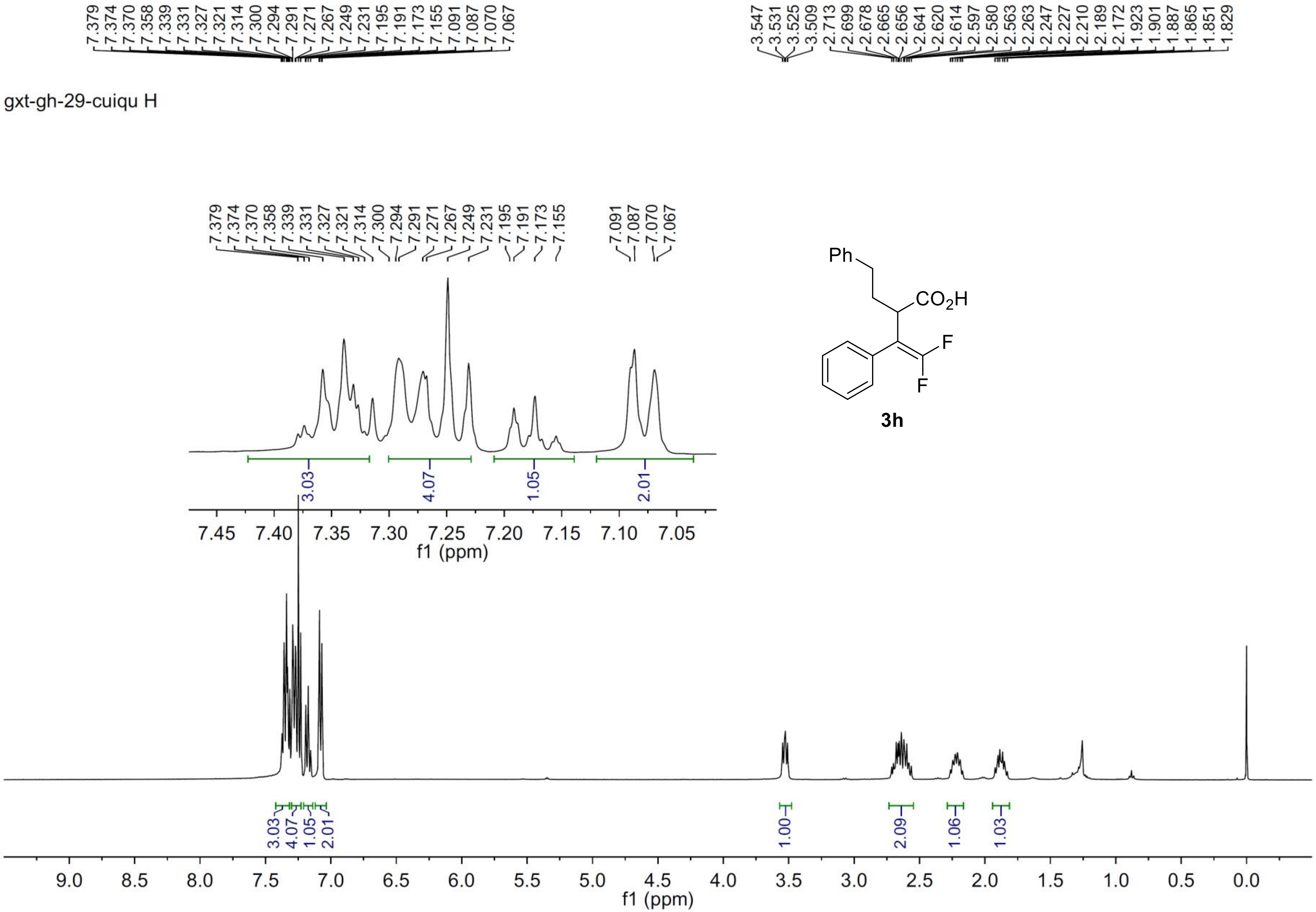
gxt-gh-29-cuiqu H
7.379 7.374 7.370 7.358 7.339 7.331 7.327 7.321 7.314 7.300 7.294 7.291 7.271 7.267 7.249 7.231 7.195 7.191 7.173 7.155 7.091 7.087 7.070 7.067
3.547 3.531 3.525 3.509 2.713 2.699 2.678 2.665 2.656 2.641 2.620 2.614 2.597 2.580 2.563 2.263 2.247 2.227 2.210 2.189 2.172 1.923 1.901 1.887 1.865 1.851 1.829
Ph
CO2H
F
F
3h
3.03
4.07
1.05
2.01
1.00
2.09
1.06
1.03
f1 (ppm)

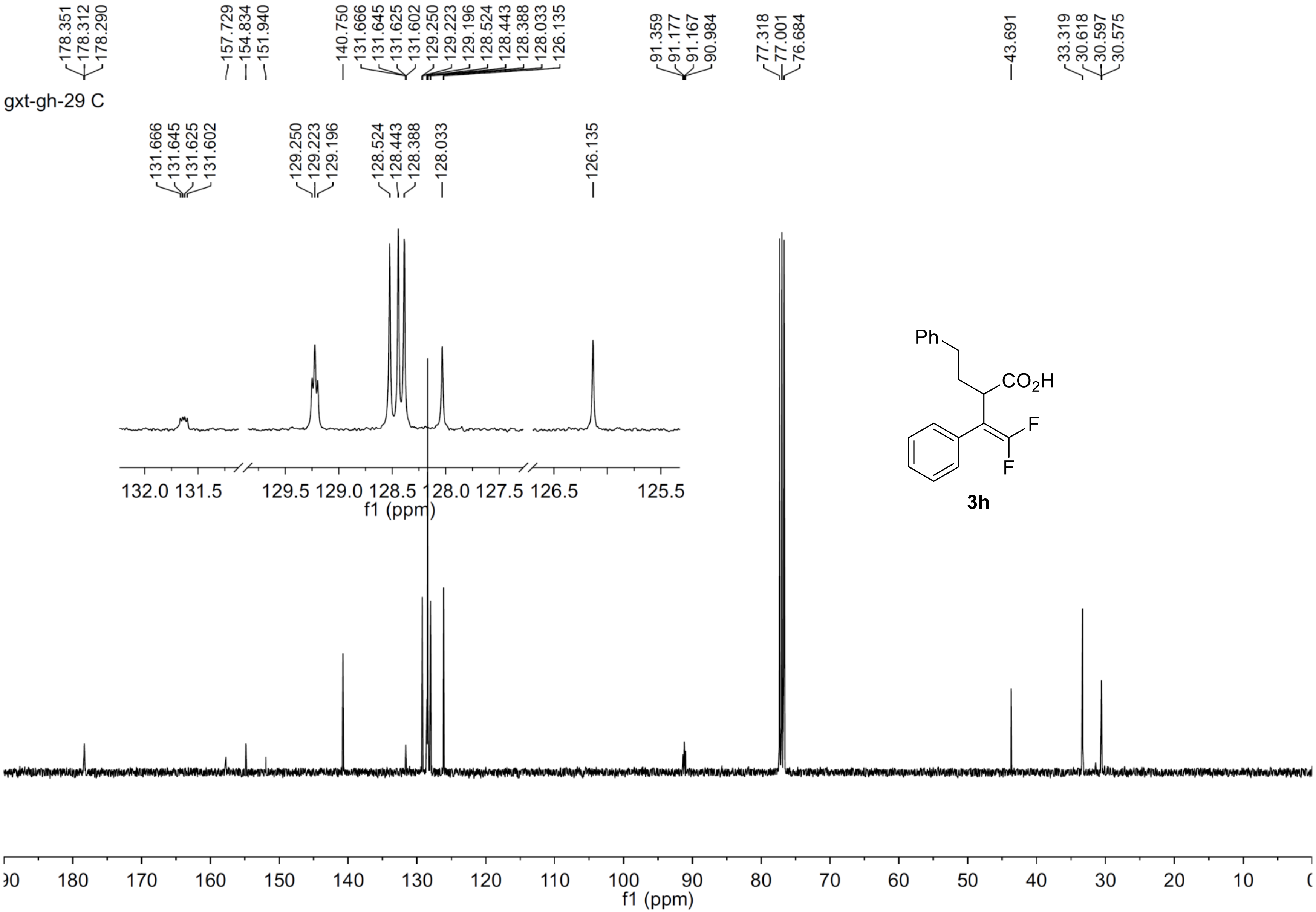
gxt-gh-29 C
178.351
178.312
178.290
157.729
154.834
151.940
140.750
131.666
131.645
131.625
131.602
129.250
129.223
129.196
128.524
128.443
128.388
128.033
126.135
91.359
91.177
91.167
90.984
77.318
77.001
76.684
43.691
33.319
30.618
30.597
30.575
f1 (ppm)
Ph
CO_2H
F
F
3h

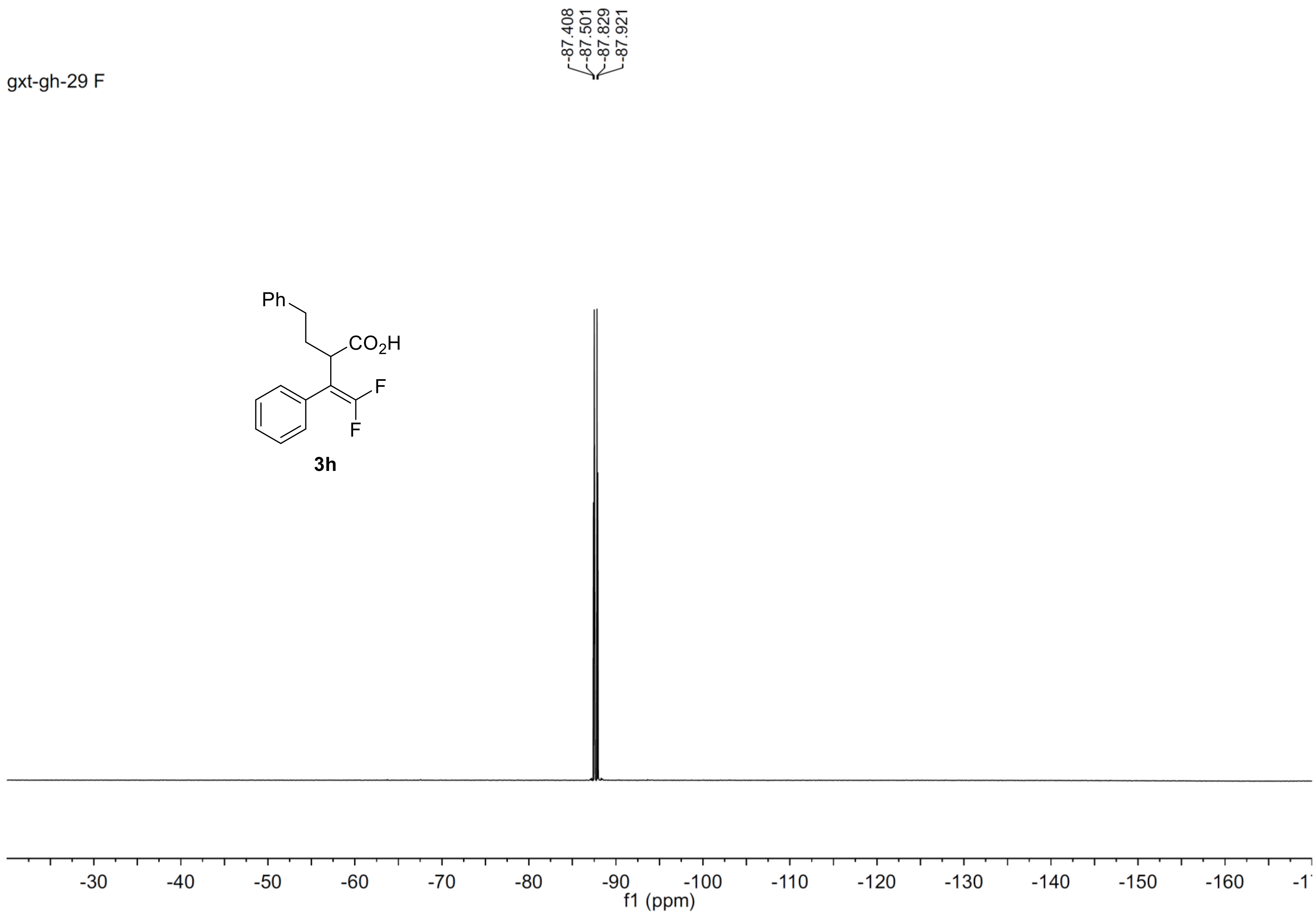
gxt-gh-29 F
-87.408
-87.501
-87.829
-87.921
Ph
CO_2H
F
F
3h
-30
-40
-50
-60
-70
-80
-90
-100
-110
-120
-130
-140
-150
-160
f1 (ppm)

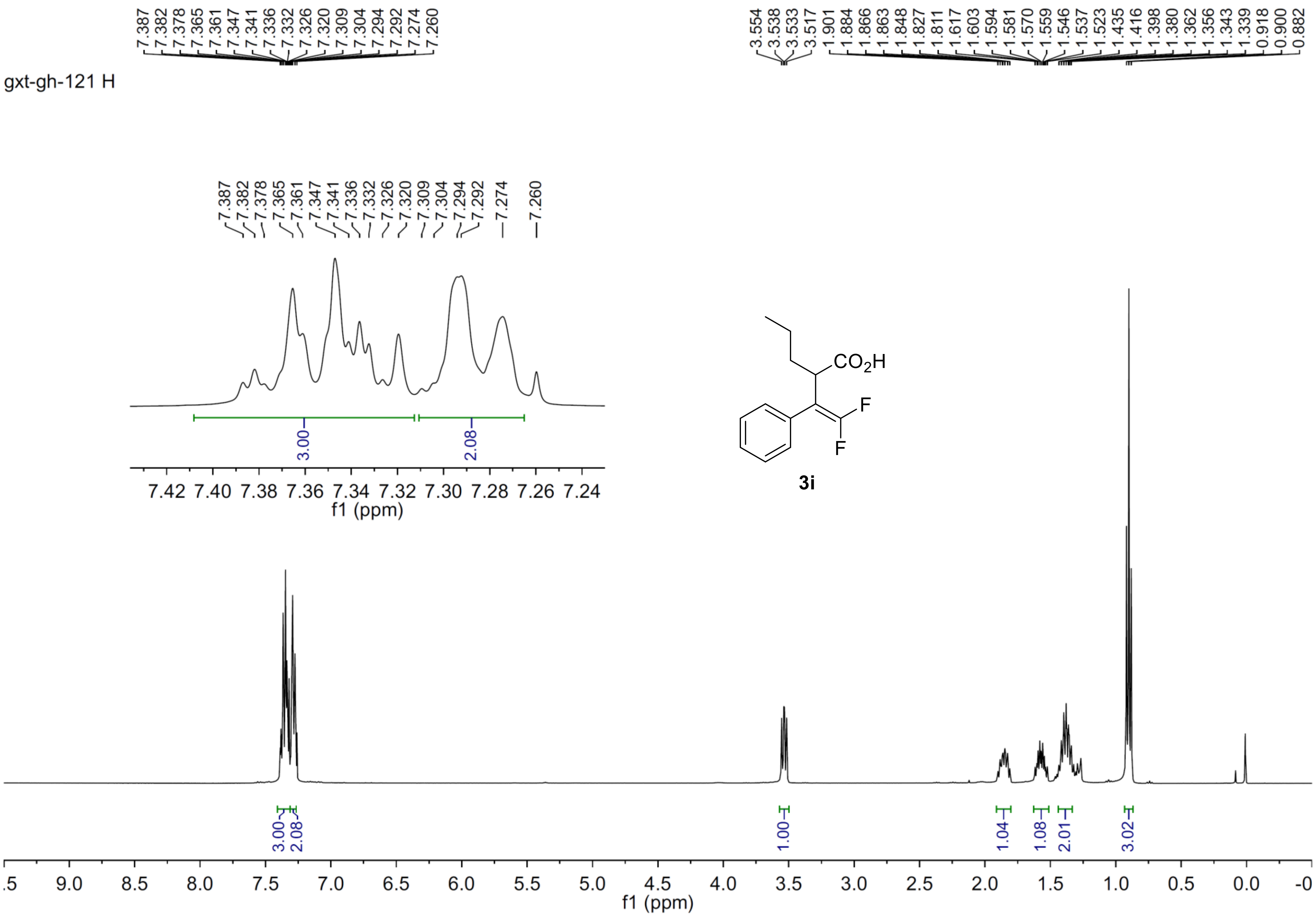
gxt-gh-121 H
7.387 7.382 7.378 7.365 7.361 7.347 7.341 7.336 7.332 7.326 7.320 7.309 7.304 7.294 7.292 7.274 7.260
3.554 3.538 3.533 3.517 1.901 1.884 1.866 1.863 1.848 1.827 1.811 1.617 1.603 1.594 1.581 1.570 1.559 1.546 1.537 1.523 1.435 1.416 1.398 1.380 1.362 1.356 1.343 1.339 0.918 0.900 0.882
3.00
2.08
f1 (ppm)
CO2H
F
F
3i
1.00
1.04
1.08
2.01
3.02

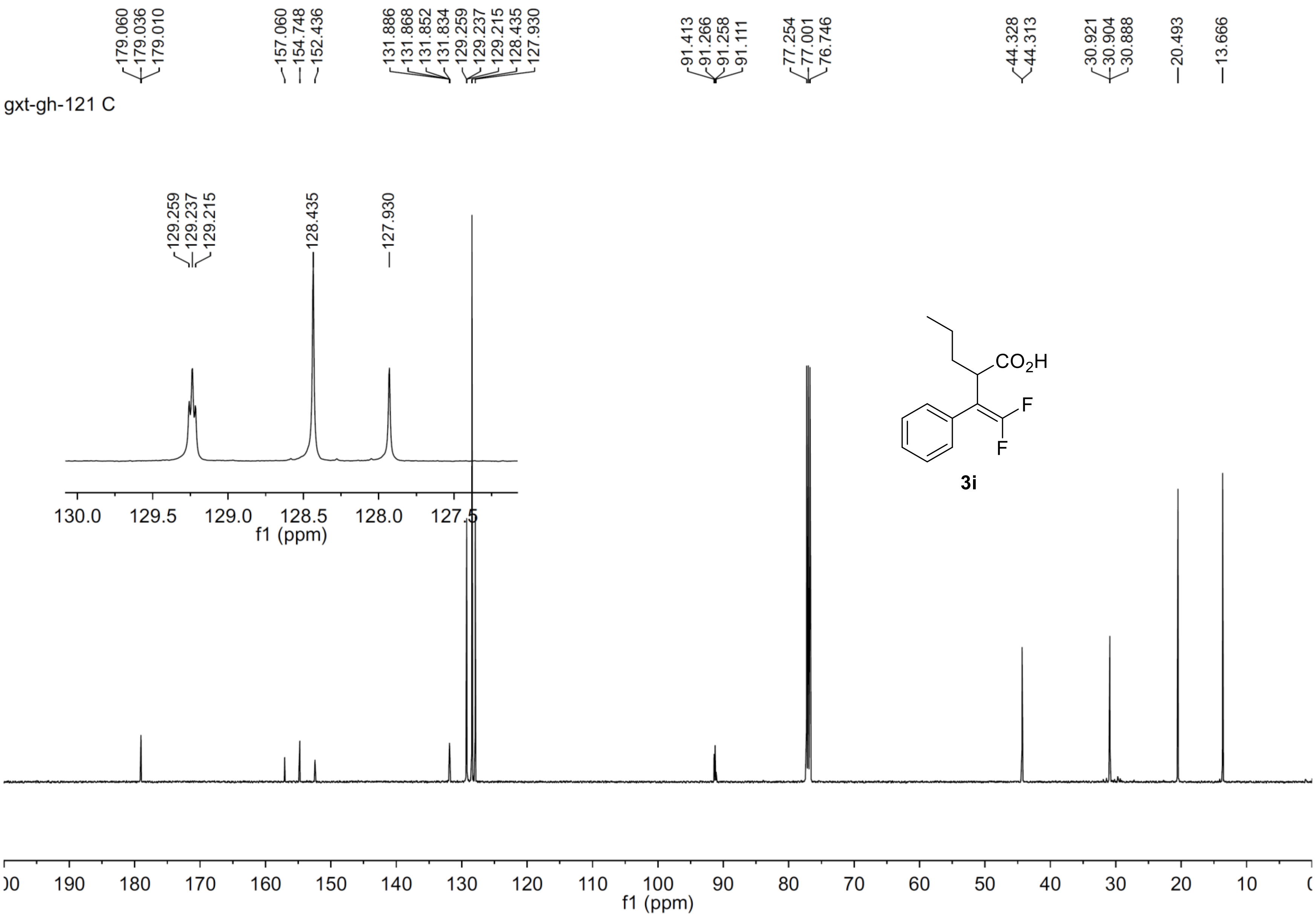
gxt-gh-121 C
179.060
179.036
179.010
157.060
154.748
152.436
131.886
131.868
131.852
131.834
129.259
129.237
129.215
128.435
127.930
91.413
91.266
91.258
91.111
77.254
77.001
76.746
44.328
44.313
30.921
30.904
30.888
20.493
13.666
CO2H
F
F
3i
f1 (ppm)

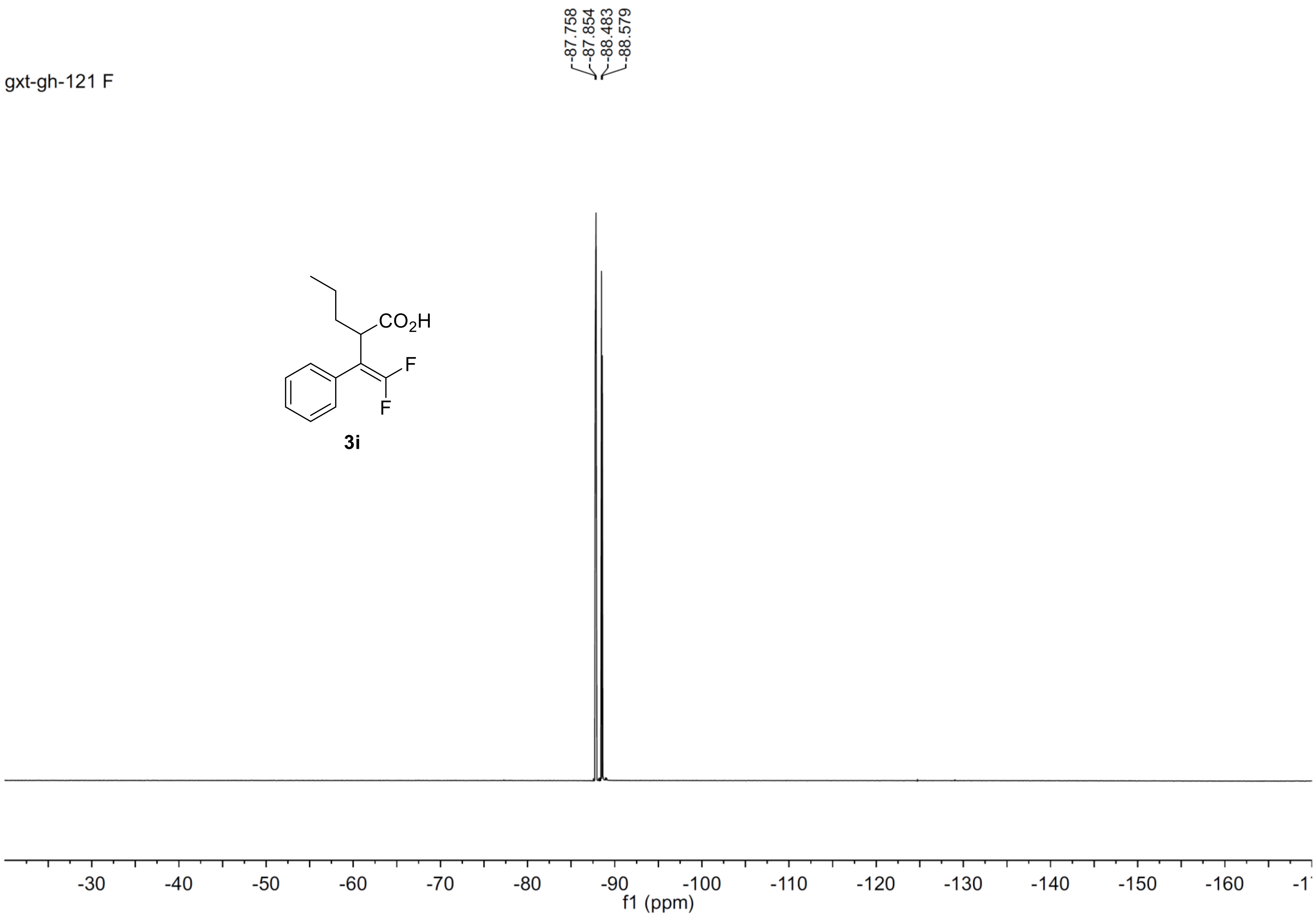
gxt-gh-121 F
-87.758
-87.854
-88.483
-88.579
CO2H
F
F
3i
-30
-40
-50
-60
-70
-80
-90
-100
-110
-120
-130
-140
-150
-160
f1 (ppm)

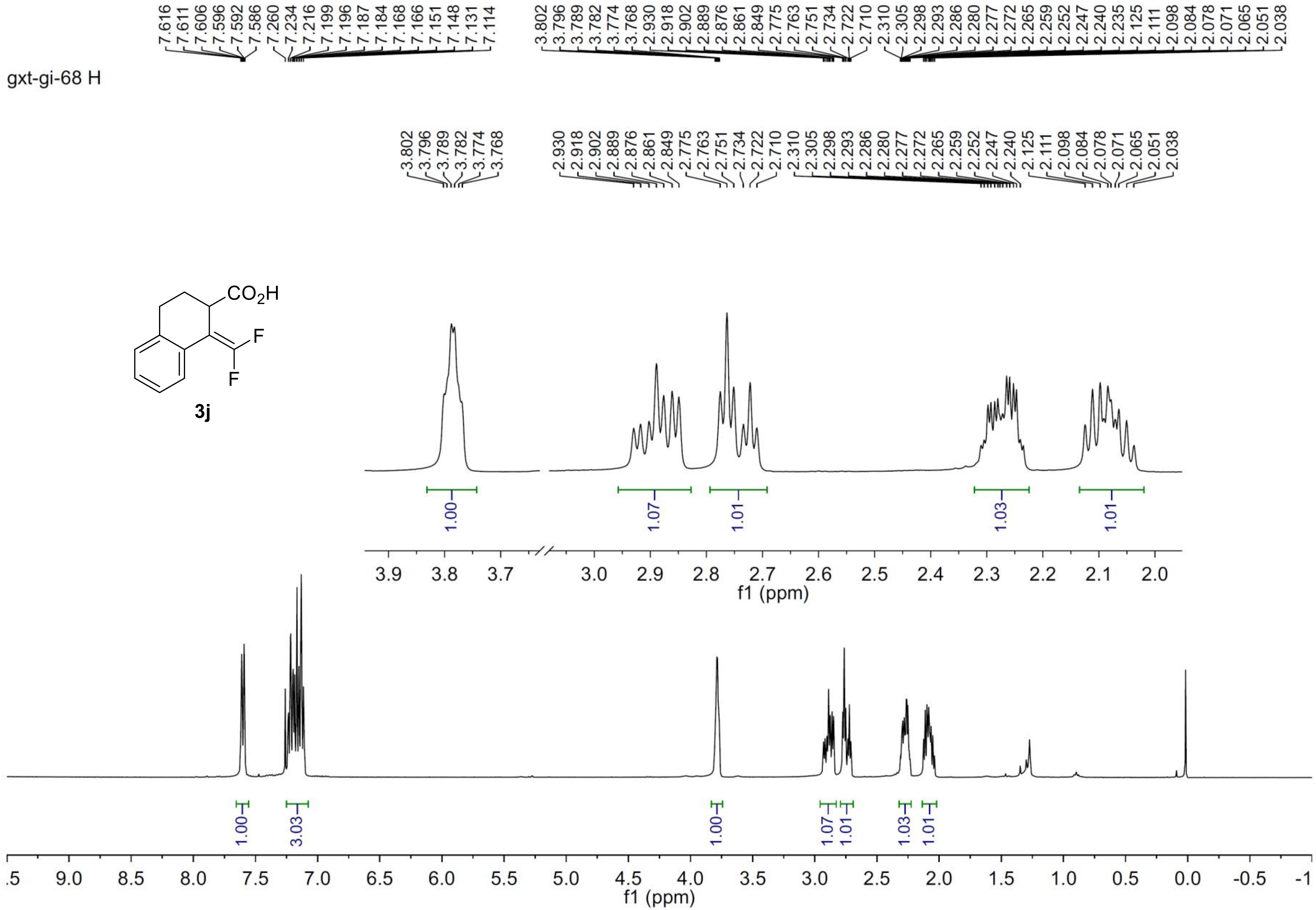
gxt-gi-68 H
7.616 7.611 7.606 7.596 7.592 7.586 7.260 7.234 7.216 7.199 7.196 7.187 7.184 7.168 7.166 7.151 7.148 7.131 7.114
3.802 3.796 3.789 3.782 3.774 3.768 2.930 2.918 2.902 2.889 2.876 2.861 2.849 2.775 2.763 2.751 2.734 2.722 2.710 2.310 2.305 2.298 2.293 2.286 2.280 2.277 2.272 2.265 2.259 2.252 2.247 2.240 2.235 2.125 2.111 2.098 2.084 2.078 2.071 2.065 2.051 2.038
CO_2H
F
F
3j
1.00
1.07
1.01
1.03
1.01
3.03
f1 (ppm)

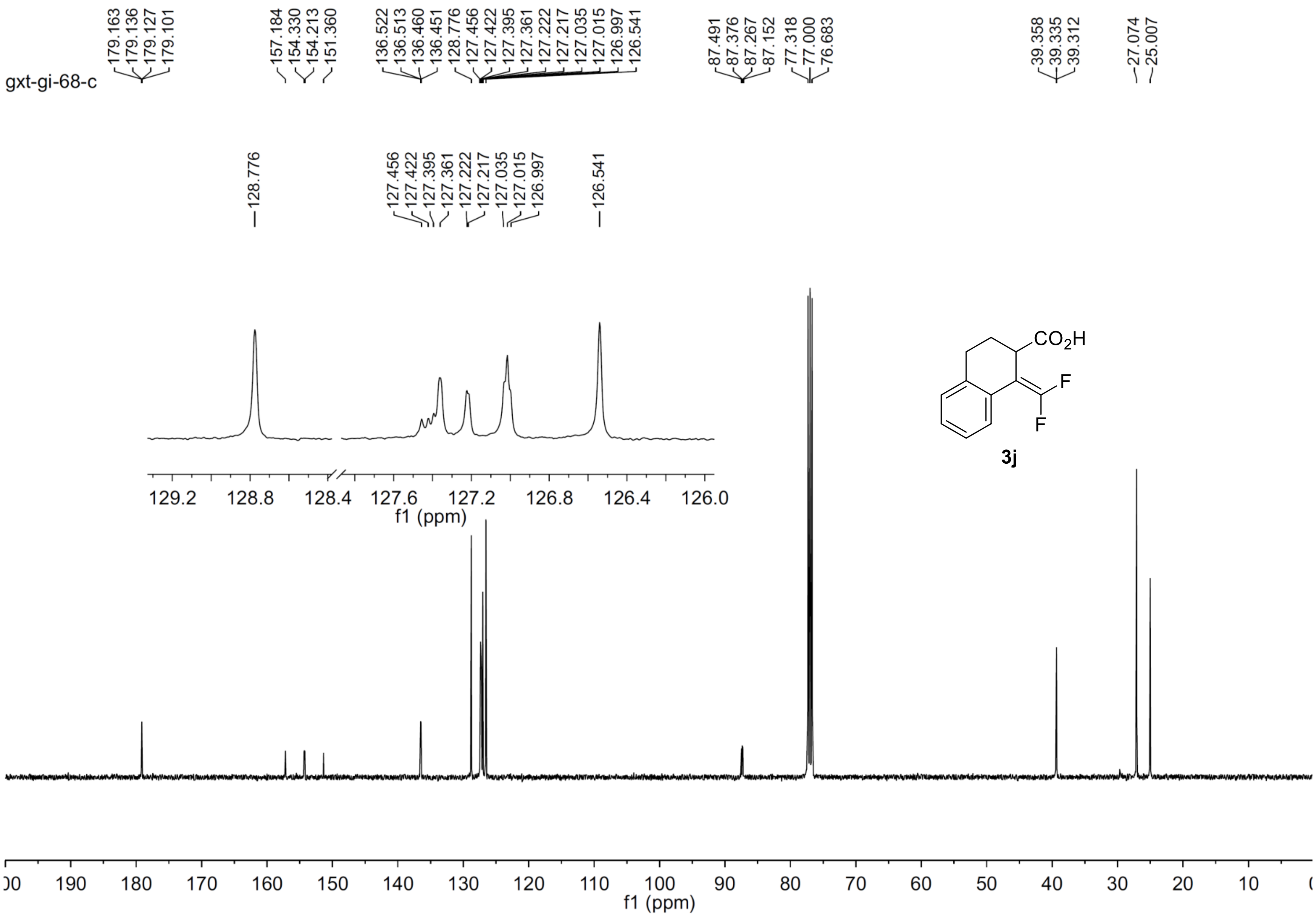
gxt-gi-68-c
179.163
179.136
179.127
179.101
157.184
154.330
154.213
151.360
136.522
136.513
136.460
136.451
128.776
127.456
127.422
127.395
127.361
127.222
127.217
127.035
127.015
126.997
126.541
87.491
87.376
87.267
87.152
77.318
77.000
76.683
39.358
39.335
39.312
27.074
25.007
CO2H
F
F
3j
f1 (ppm)

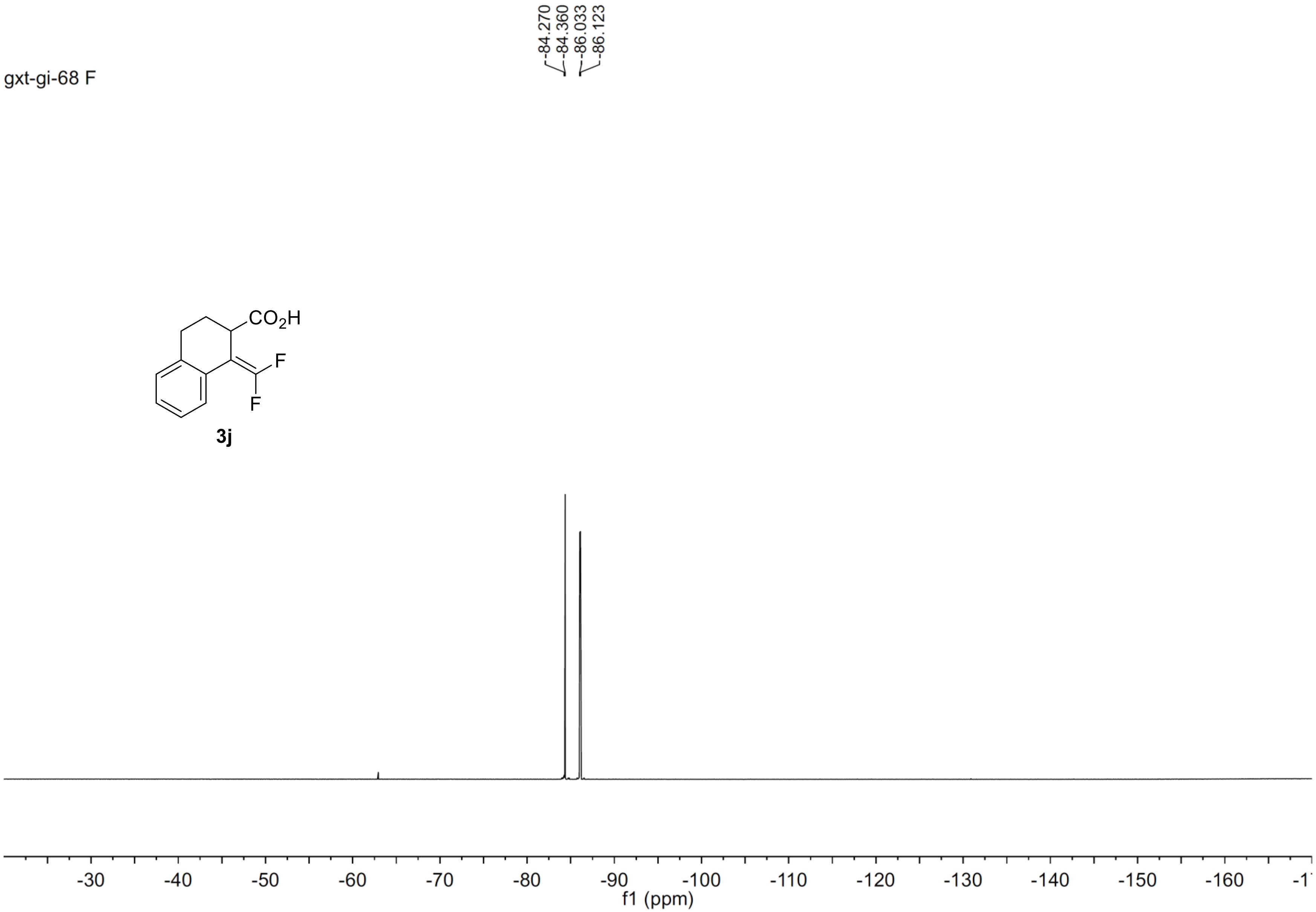
gxt-gi-68 F
-84.270
-84.360
-86.033
-86.123
CO_2H
F
F
3j
-30
-40
-50
-60
-70
-80
-90
-100
-110
-120
-130
-140
-150
-160
f1 (ppm)

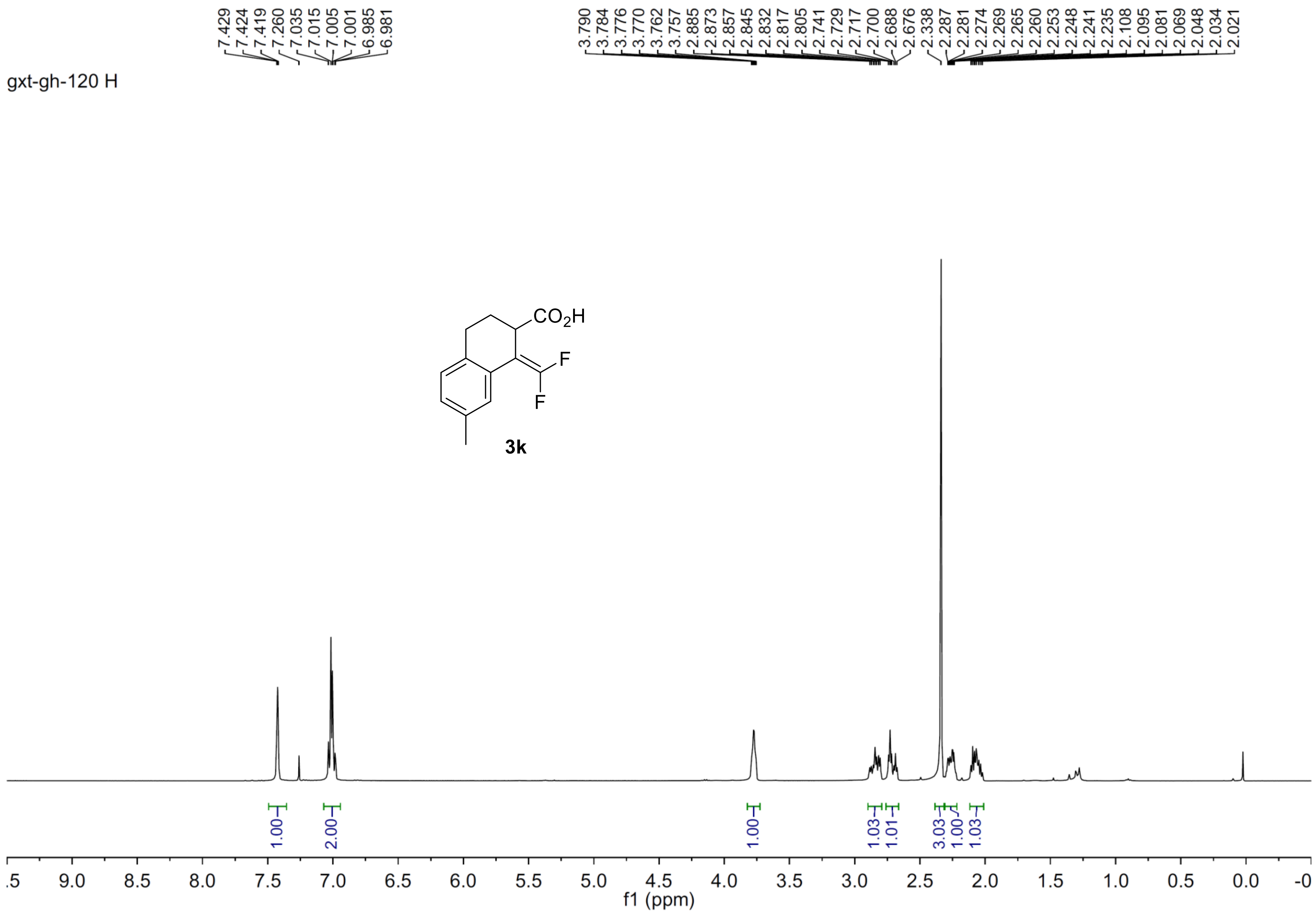
gxt-gh-120 H
7.429
7.424
7.419
7.260
7.035
7.015
7.005
7.001
6.985
6.981
3.790
3.784
3.776
3.770
3.762
3.757
2.885
2.873
2.857
2.845
2.832
2.817
2.805
2.741
2.729
2.717
2.700
2.688
2.676
2.338
2.287
2.281
2.274
2.269
2.265
2.260
2.253
2.248
2.241
2.235
2.108
2.095
2.081
2.069
2.048
2.034
2.021
CO2H
F
F
3k
1.00
2.00
1.00
1.03
1.01
3.03
1.00
1.03
9.0
8.5
8.0
7.5
7.0
6.5
6.0
5.5
5.0
4.5
4.0
3.5
3.0
2.5
2.0
1.5
1.0
0.5
0.0
f1 (ppm)

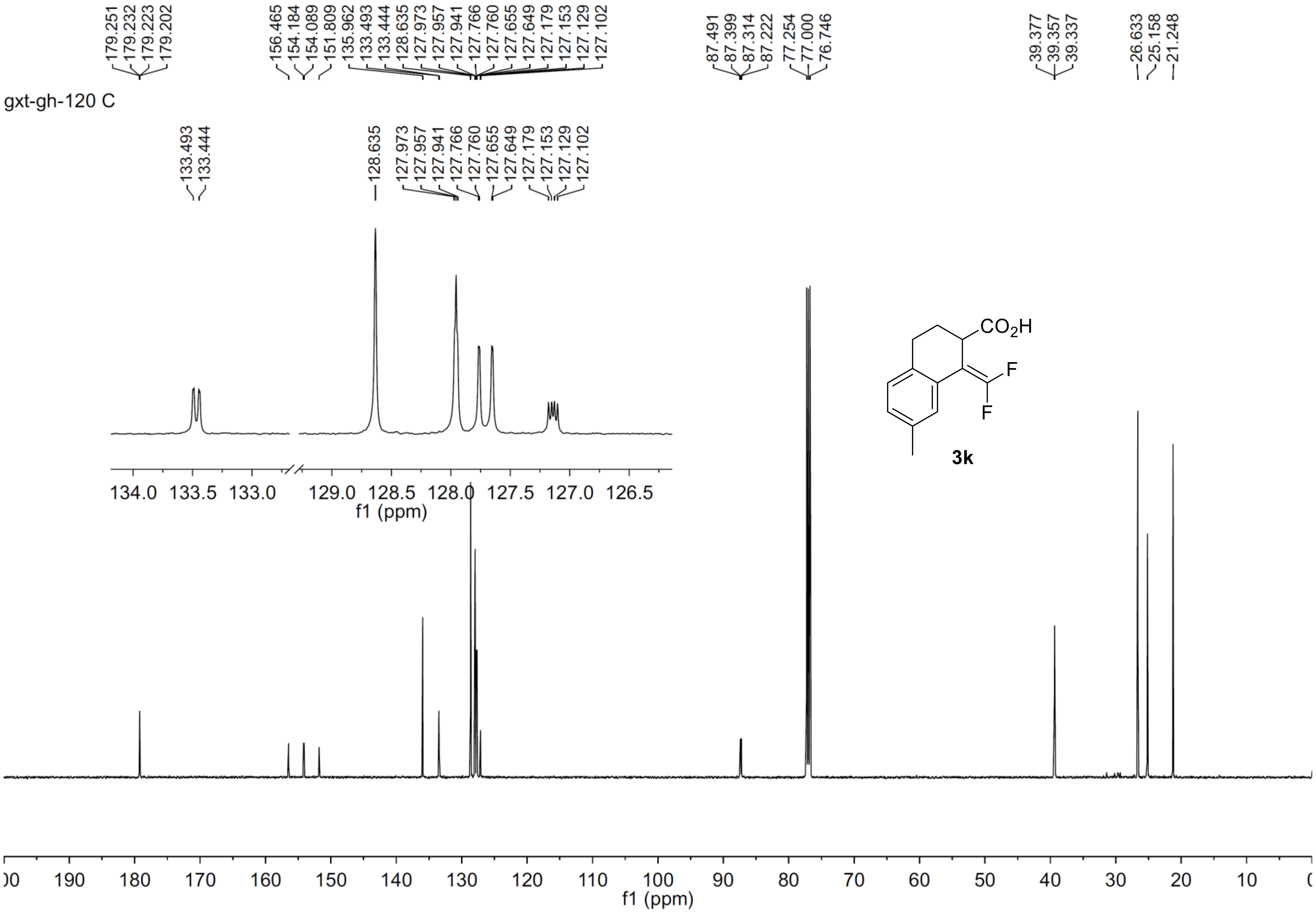
gxt-gh-120 C
179.251
179.232
179.223
179.202
156.465
154.184
154.089
151.809
135.962
133.493
133.444
128.635
127.973
127.957
127.941
127.766
127.760
127.655
127.649
127.179
127.153
127.129
127.102
87.491
87.399
87.314
87.222
77.254
77.000
76.746
39.377
39.357
39.337
26.633
25.158
21.248
CO2H
F
F
3k
134.0 133.5 133.0 129.0 128.5 128.0 127.5 127.0 126.5
f1 (ppm)
190 180 170 160 150 140 130 120 110 100 90 80 70 60 50 40 30 20 10
f1 (ppm)

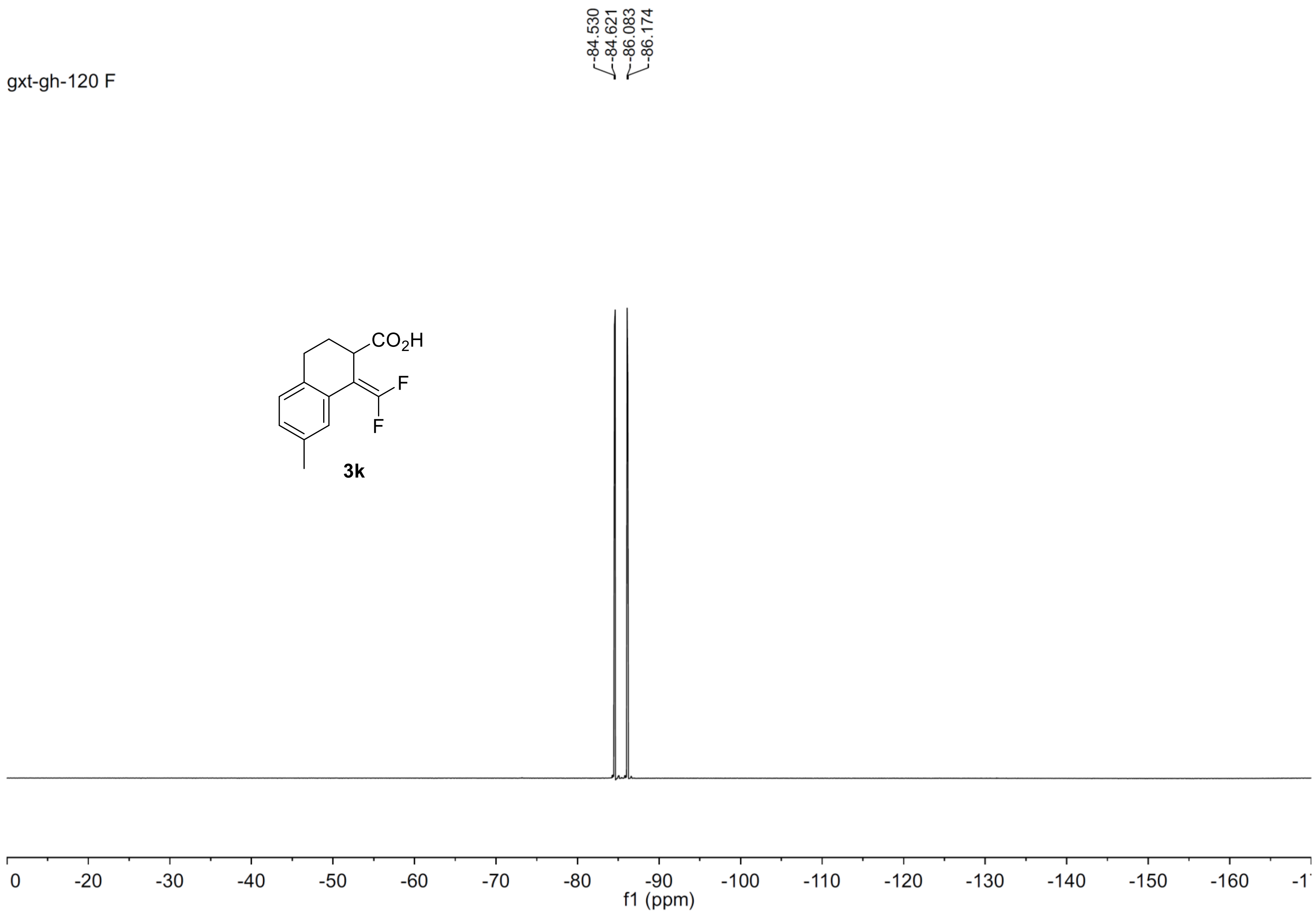
gxt-gh-120 F
-84.530
-84.621
-86.083
-86.174
CO_2H
F
F
3k
0
-20
-30
-40
-50
-60
-70
-80
-90
-100
-110
-120
-130
-140
-150
-160
f1 (ppm)

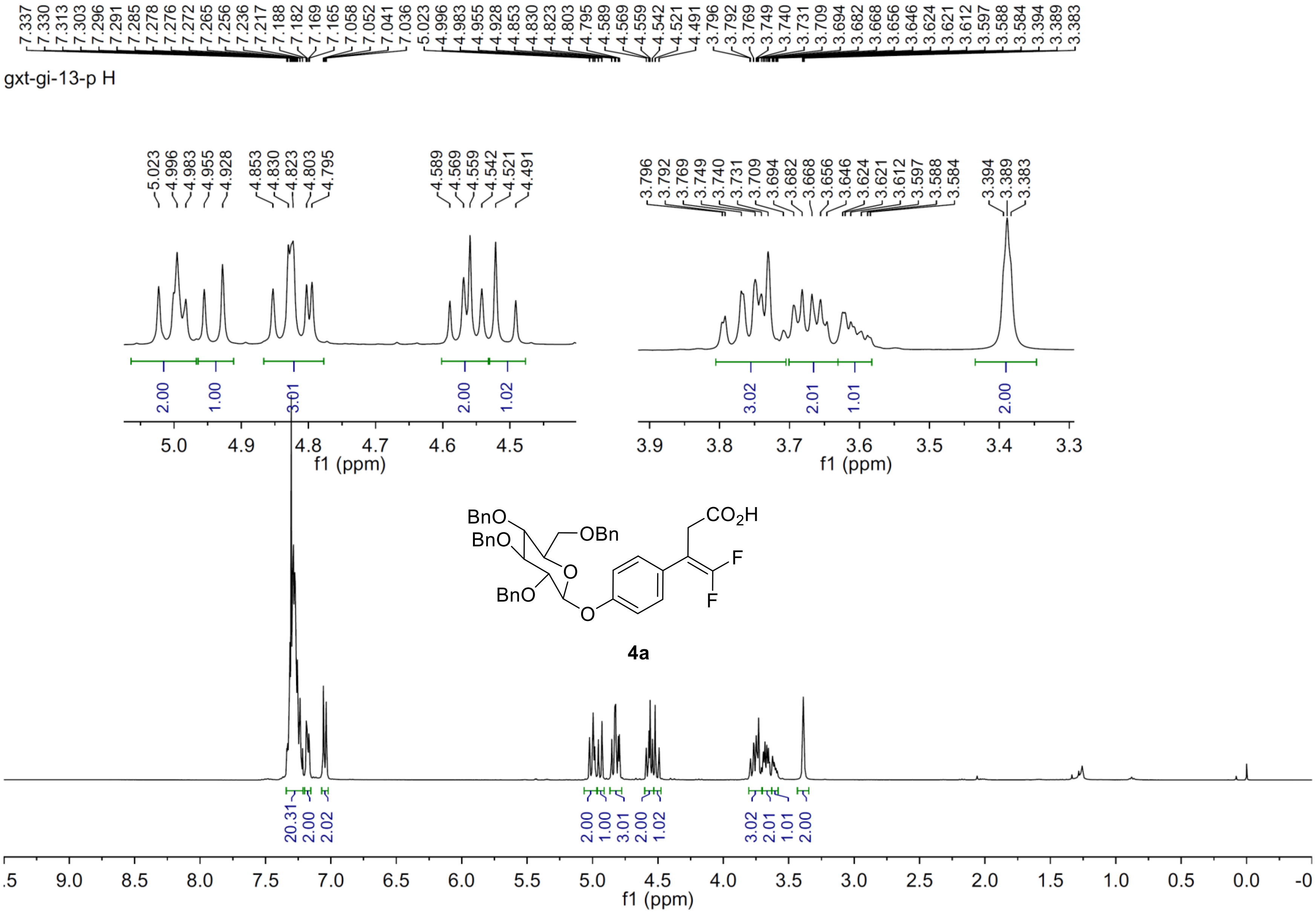
7.337 7.330 7.313 7.303 7.296 7.291 7.285 7.278 7.276 7.272 7.265 7.256 7.236 7.217 7.188 7.182 7.169 7.165 7.058 7.052 7.041 7.036 5.023 4.996 4.983 4.955 4.928 4.853 4.830 4.823 4.803 4.795 4.589 4.569 4.559 4.542 4.521 4.491 3.796 3.792 3.769 3.749 3.740 3.731 3.709 3.694 3.682 3.668 3.656 3.646 3.624 3.621 3.612 3.597 3.588 3.584 3.394 3.389 3.383
gxt-gi-13-p H
BnO
BnO
OBn
BnO
O
O
CO_2H
F
F
4a
f1 (ppm)

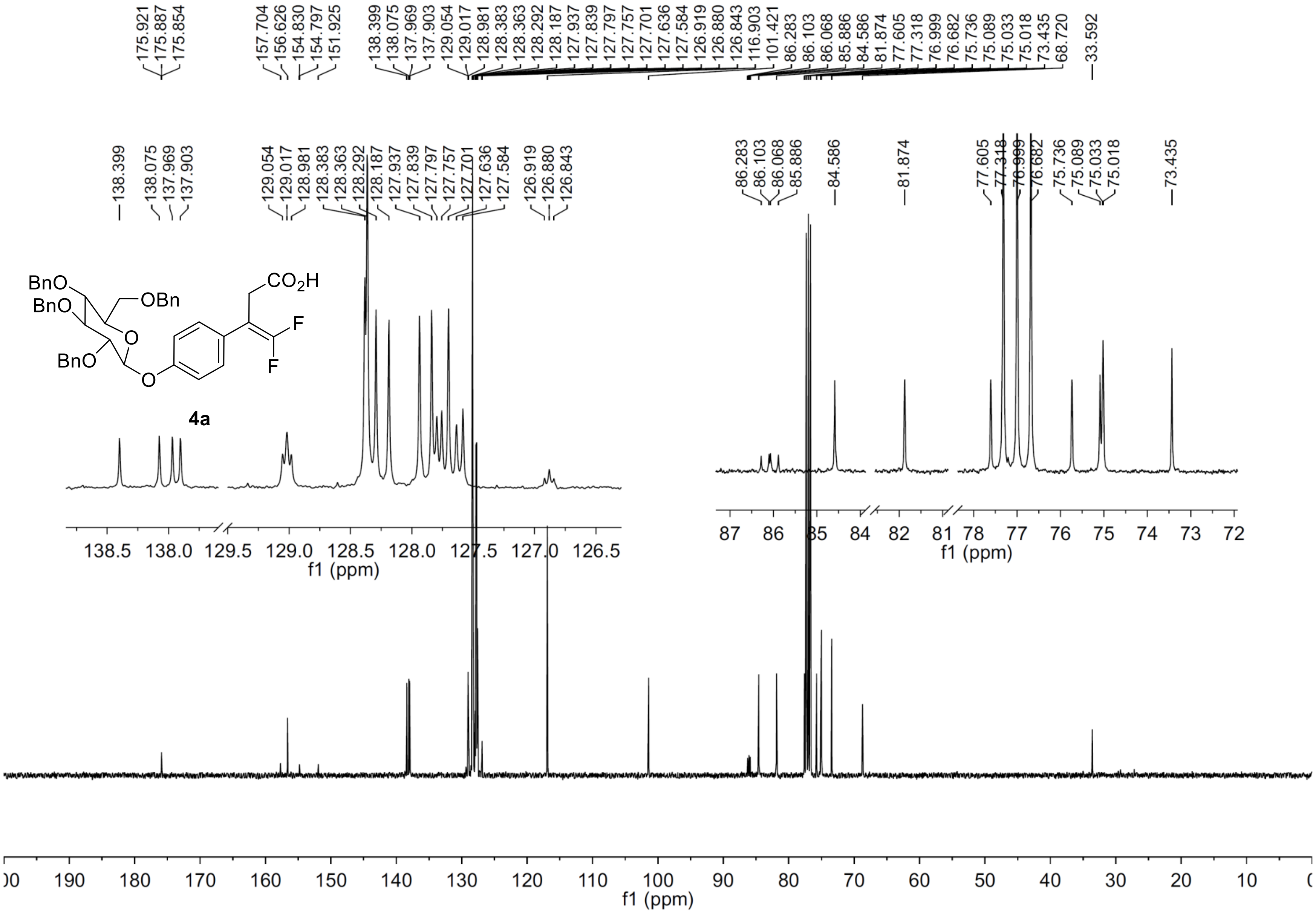
175.921
175.887
175.854
157.704
156.626
154.830
154.797
151.925
138.399
138.075
137.969
137.903
129.054
129.017
128.981
128.383
128.363
128.292
128.187
127.937
127.839
127.797
127.757
127.701
127.636
127.584
126.919
126.880
126.843
116.903
101.421
86.283
86.103
86.068
85.886
84.586
81.874
77.605
77.318
76.999
76.682
75.736
75.089
75.033
75.018
73.435
68.720
33.592
BnO
BnO
OBn
BnO
O
O
CO_2H
F
F
4a
f1 (ppm)
f1 (ppm)
f1 (ppm)

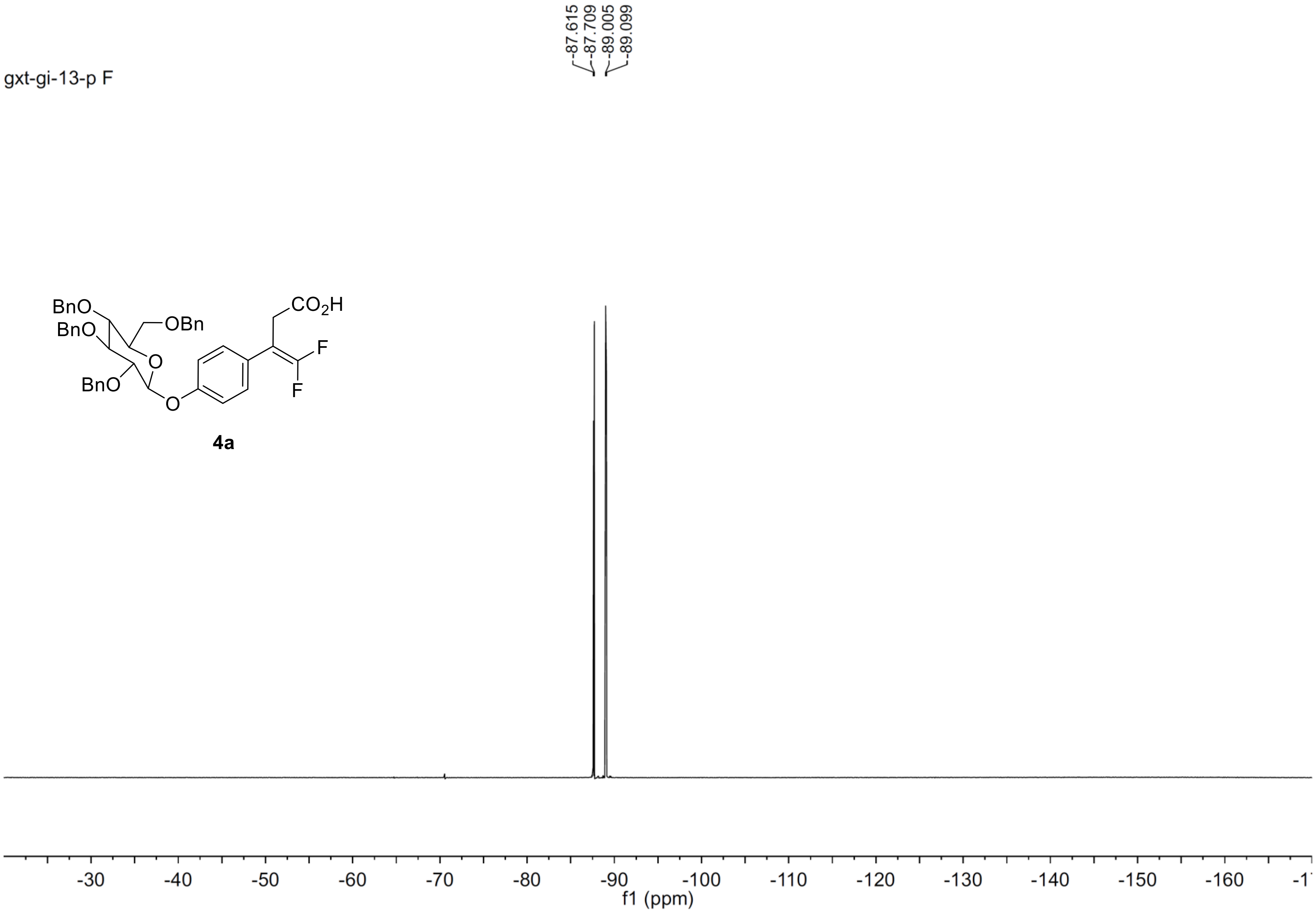
gxt-gi-13-p F
-87.615
-87.709
-89.005
-89.099
BnO
BnO
BnO
OBn
O
O
CO2H
F
F
4a
-30
-40
-50
-60
-70
-80
-90
-100
-110
-120
-130
-140
-150
-160
f1 (ppm)

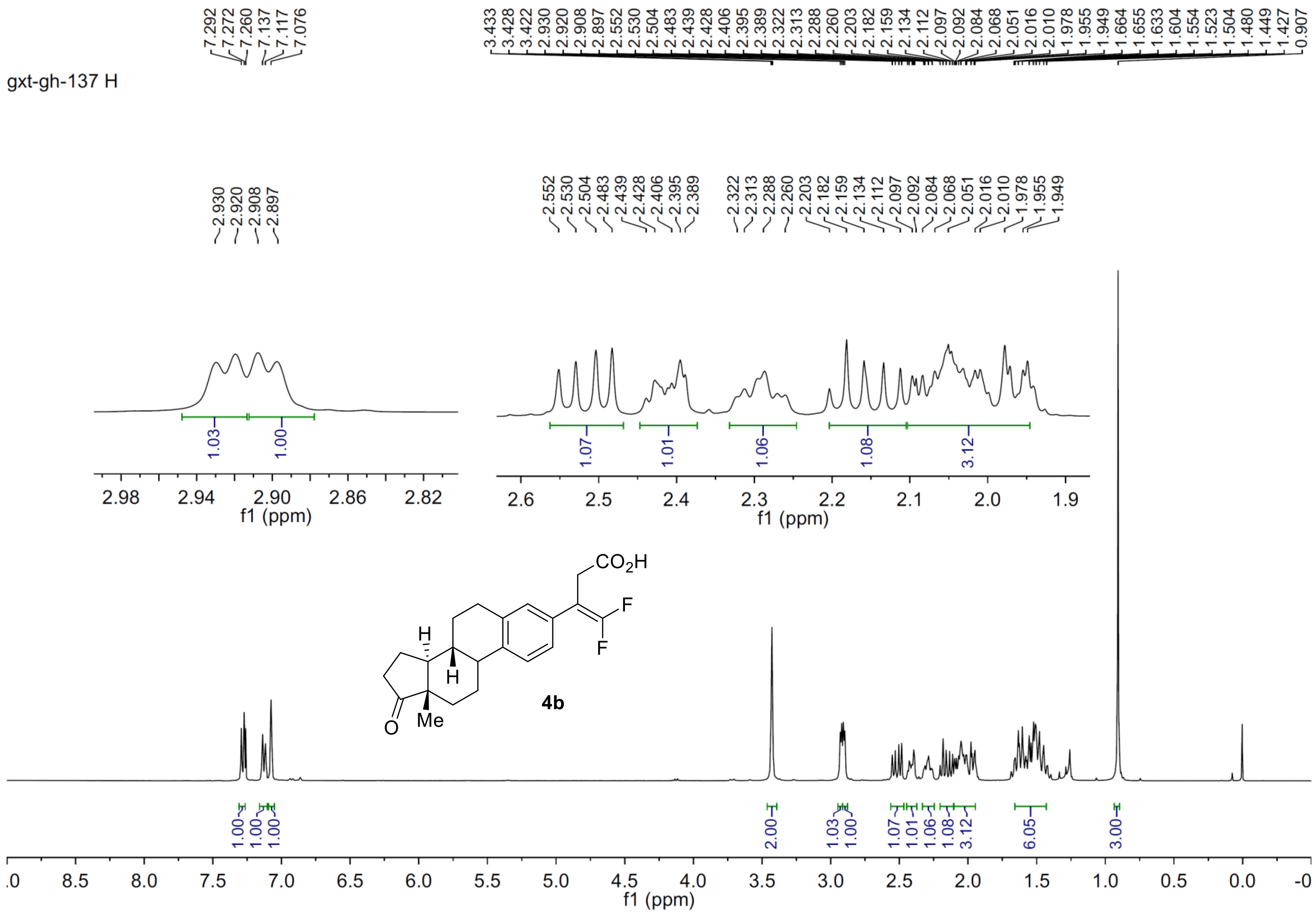
gxt-gh-137 H
7.292
7.272
7.260
7.137
7.117
7.076
3.433
3.428
3.422
2.930
2.920
2.908
2.897
2.552
2.530
2.504
2.483
2.439
2.428
2.406
2.395
2.389
2.322
2.313
2.288
2.260
2.203
2.182
2.159
2.134
2.112
2.097
2.092
2.084
2.068
2.051
2.016
2.010
1.978
1.955
1.949
1.664
1.655
1.633
1.604
1.554
1.523
1.504
1.480
1.449
1.427
0.907
CO2H
F
F
H
H
O
Me
4b
f1 (ppm)

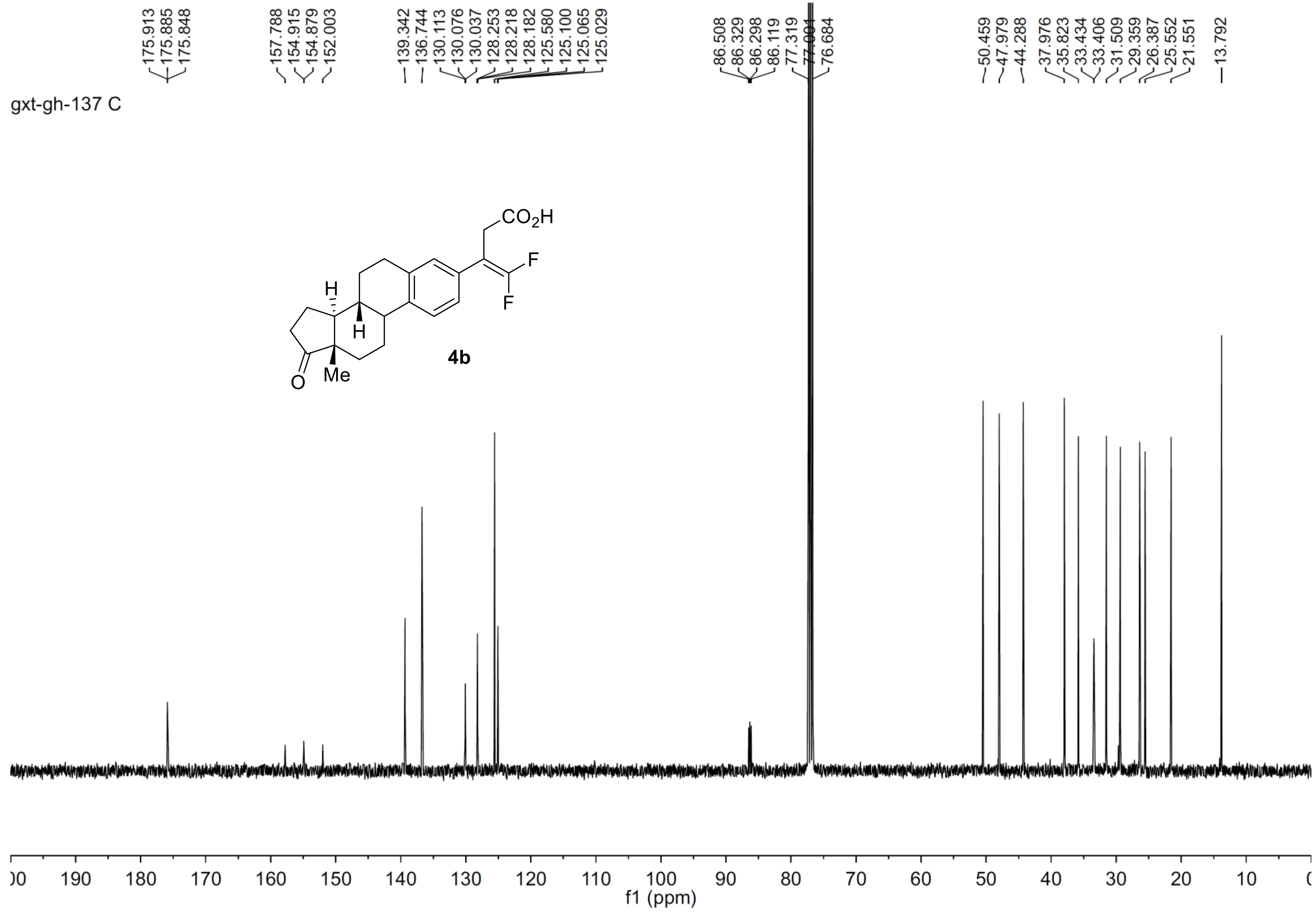

gxt-gh-137 C
175.913
175.885
175.848
157.788
154.915
154.879
152.003
139.342
136.744
130.113
130.076
130.037
128.253
128.218
128.182
125.580
125.100
125.065
125.029
86.508
86.329
86.298
86.119
77.319
77.001
76.684
50.459
47.979
44.288
37.976
35.823
33.434
33.406
31.509
29.359
26.387
25.552
21.551
13.792
CO2H
F
F
H
H
O
Me
4b
190
180
170
160
150
140
130
120
110
100
90
80
70
60
50
40
30
20
10
f1 (ppm)

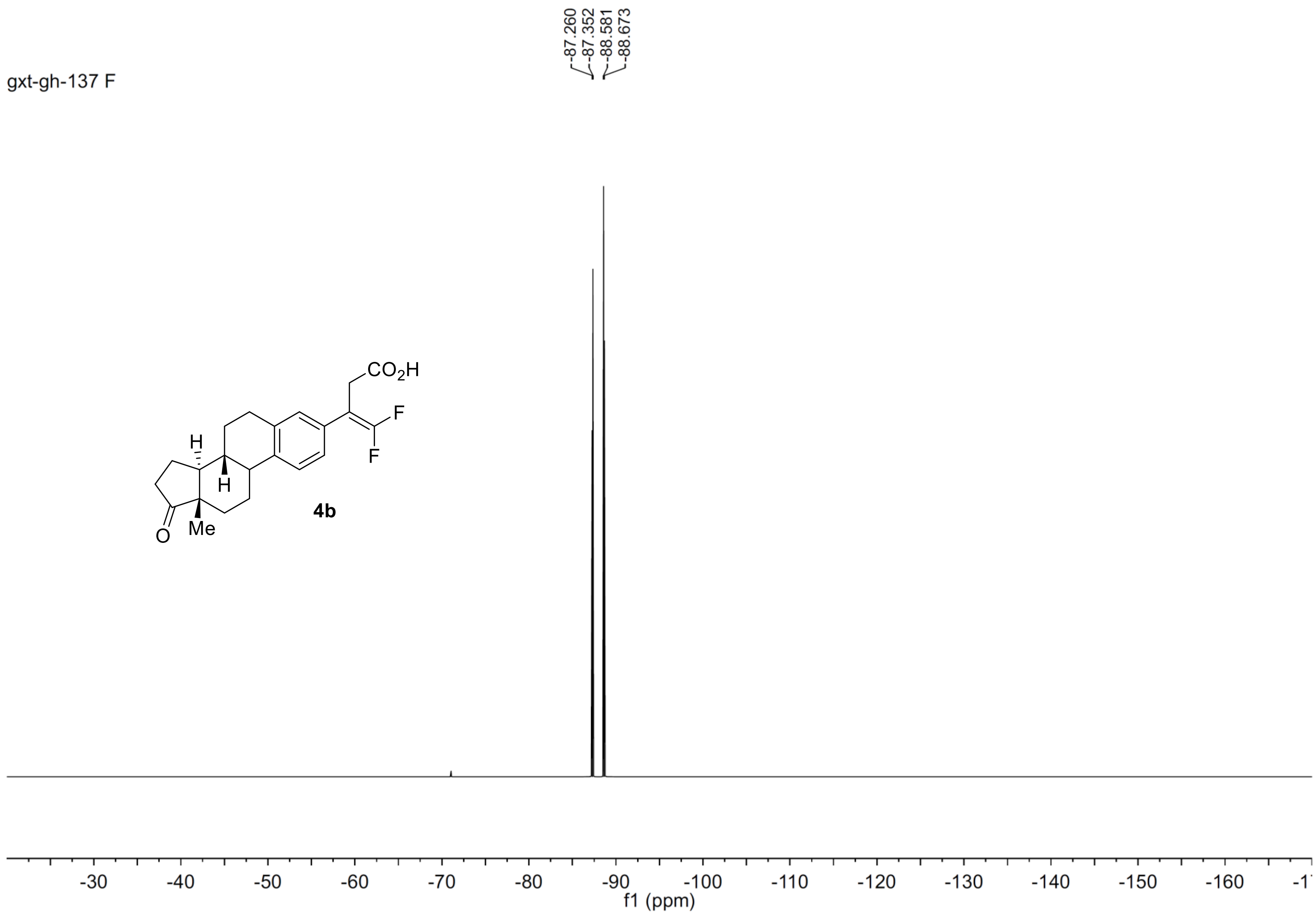
gxt-gh-137 F
-87.260
-87.352
-88.581
-88.673
CO2H
F
F
H
H
O
Me
4b
-30
-40
-50
-60
-70
-80
-90
-100
-110
-120
-130
-140
-150
-160
f1 (ppm)

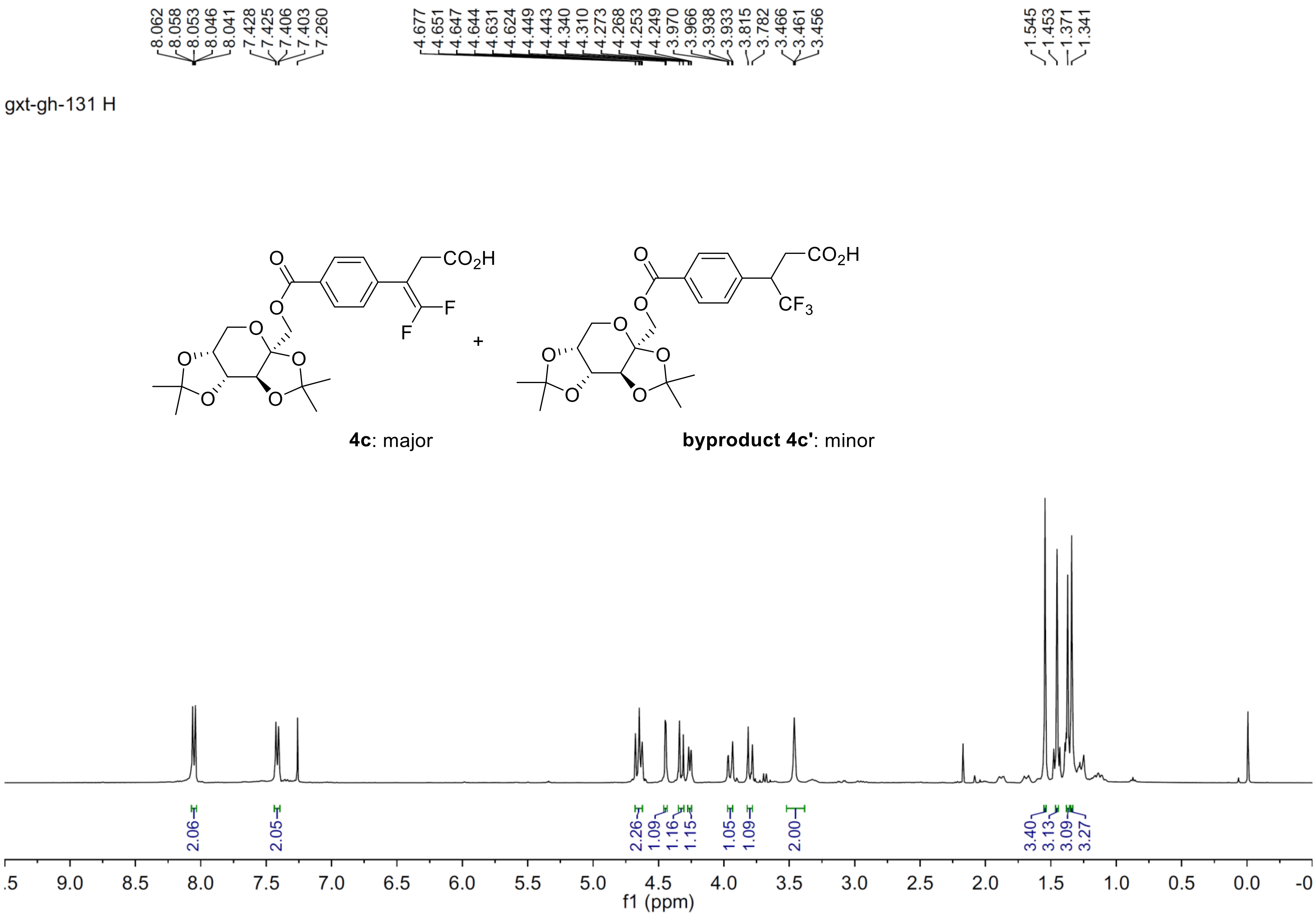

4c: major

byproduct 4c': minor

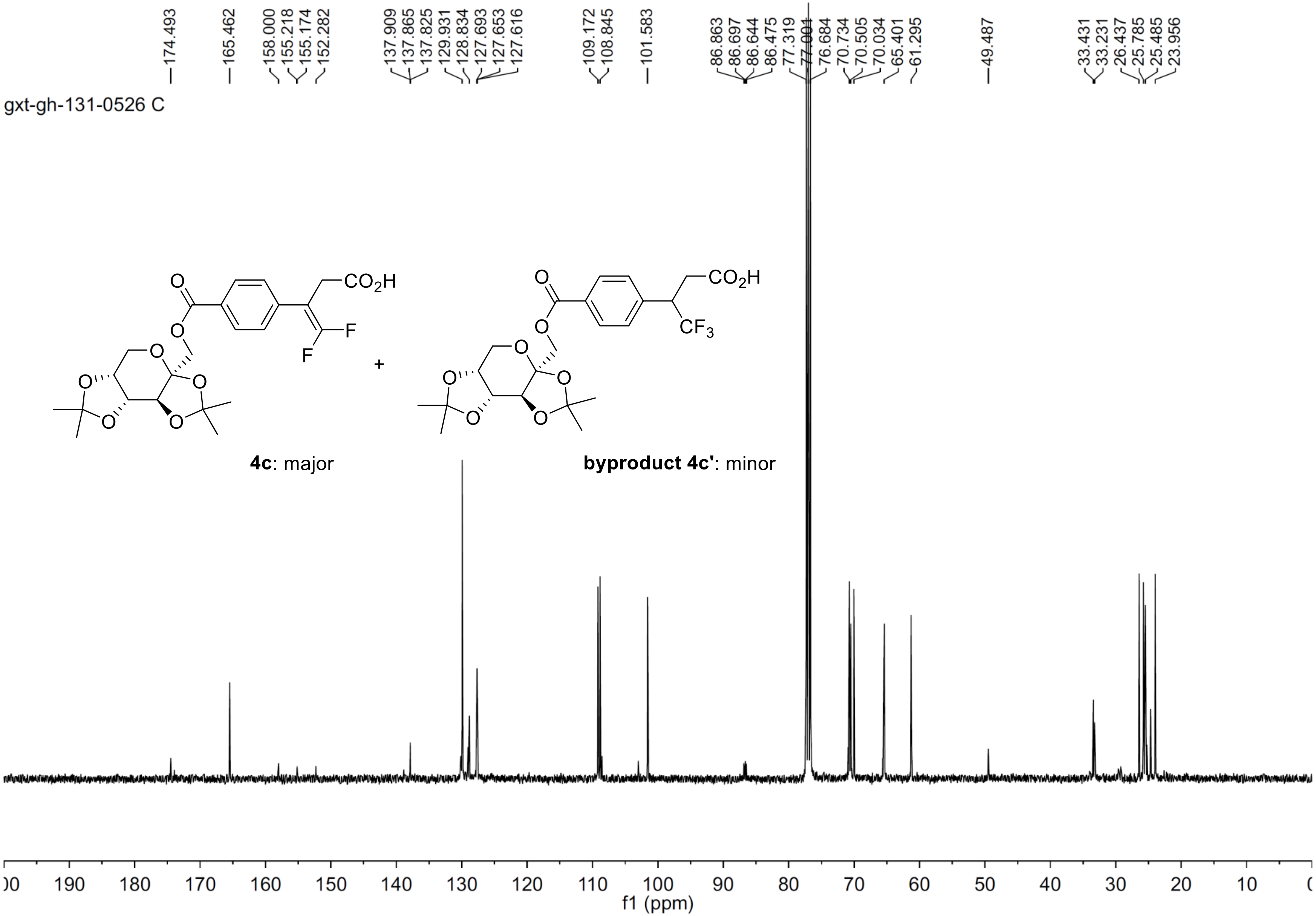
gxt-gh-131-0526 C
174.493
165.462
158.000
155.218
155.174
152.282
137.909
137.865
137.825
129.931
128.834
127.693
127.653
127.616
109.172
108.845
101.583
86.863
86.697
86.644
86.475
77.319
77.001
76.684
70.734
70.505
70.034
65.401
61.295
49.487
33.431
33.231
26.437
25.785
25.485
23.956
CO_2H
F
F
+
CF_3
4c: major
byproduct 4c': minor
f1 (ppm)

gxt-gh-131 F

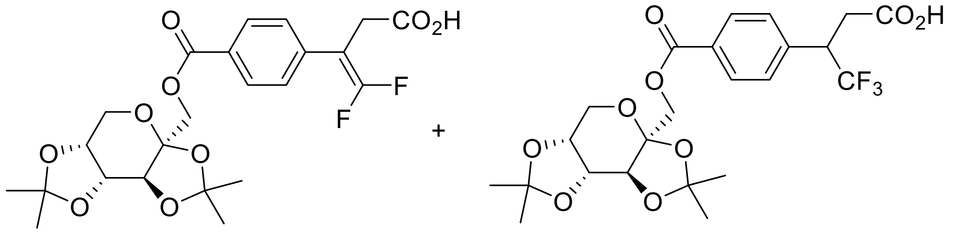

4c: major + **byproduct 4c'**: minor

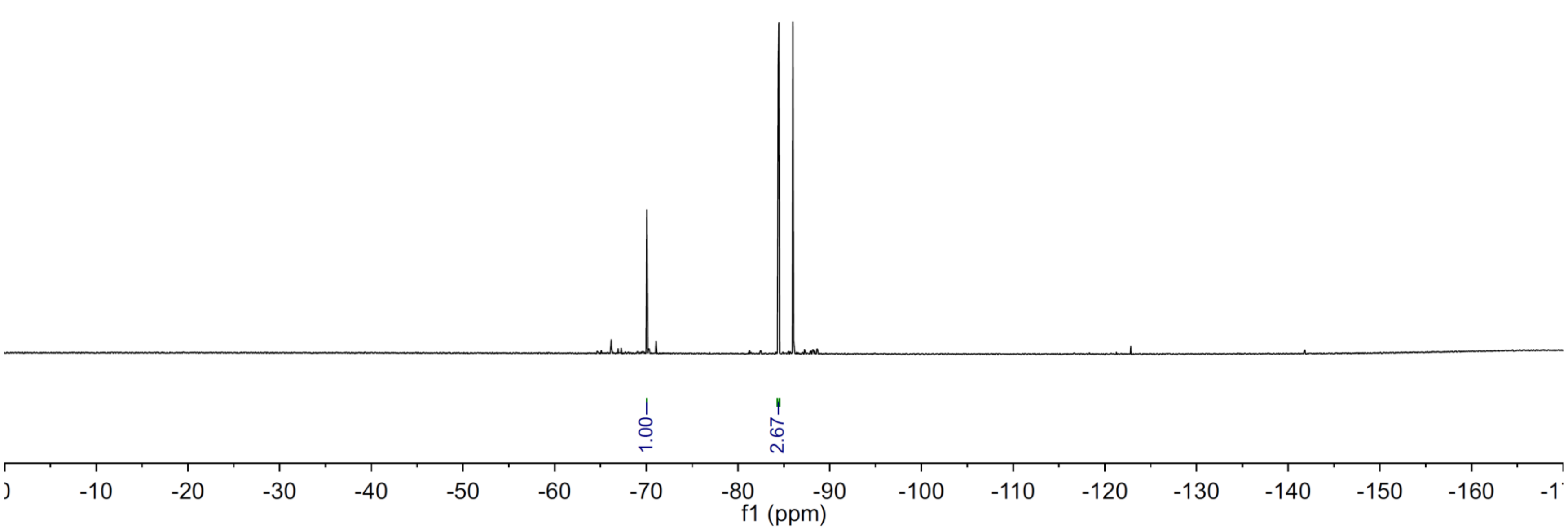

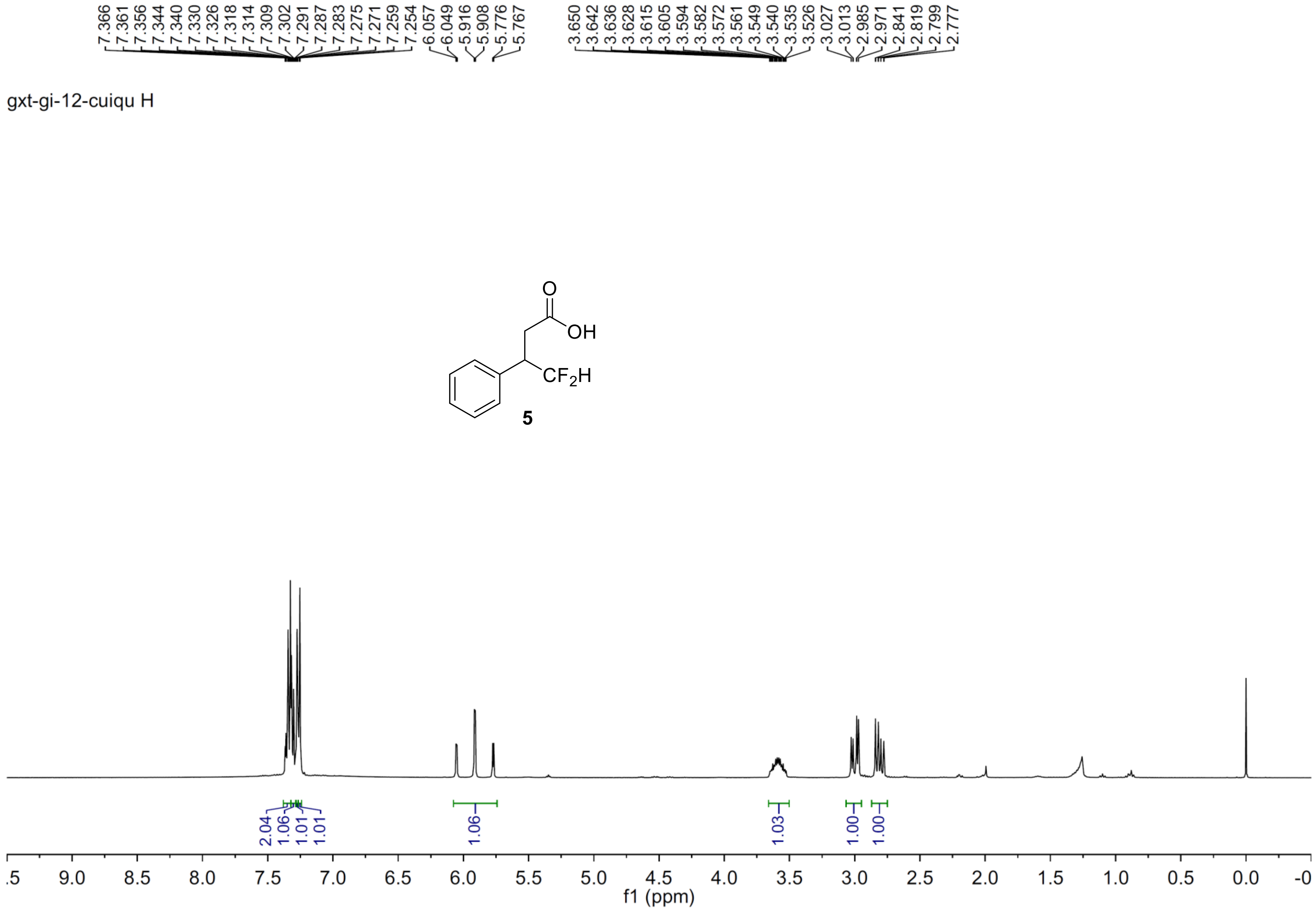
7.366
7.361
7.356
7.344
7.340
7.330
7.326
7.318
7.314
7.309
7.302
7.291
7.287
7.283
7.275
7.271
7.259
7.254
6.057
6.049
5.916
5.908
5.776
5.767
3.650
3.642
3.636
3.628
3.615
3.605
3.594
3.582
3.572
3.561
3.549
3.540
3.535
3.526
3.027
3.013
2.985
2.971
2.841
2.819
2.799
2.777
gxt-gi-12-cuiqu H
O
OH
CF2H
5
2.04
1.06
1.01
1.01
1.06
1.03
1.00
1.00
.5
9.0
8.5
8.0
7.5
7.0
6.5
6.0
5.5
5.0
4.5
4.0
3.5
3.0
2.5
2.0
1.5
1.0
0.5
0.0
-0
f1 (ppm)

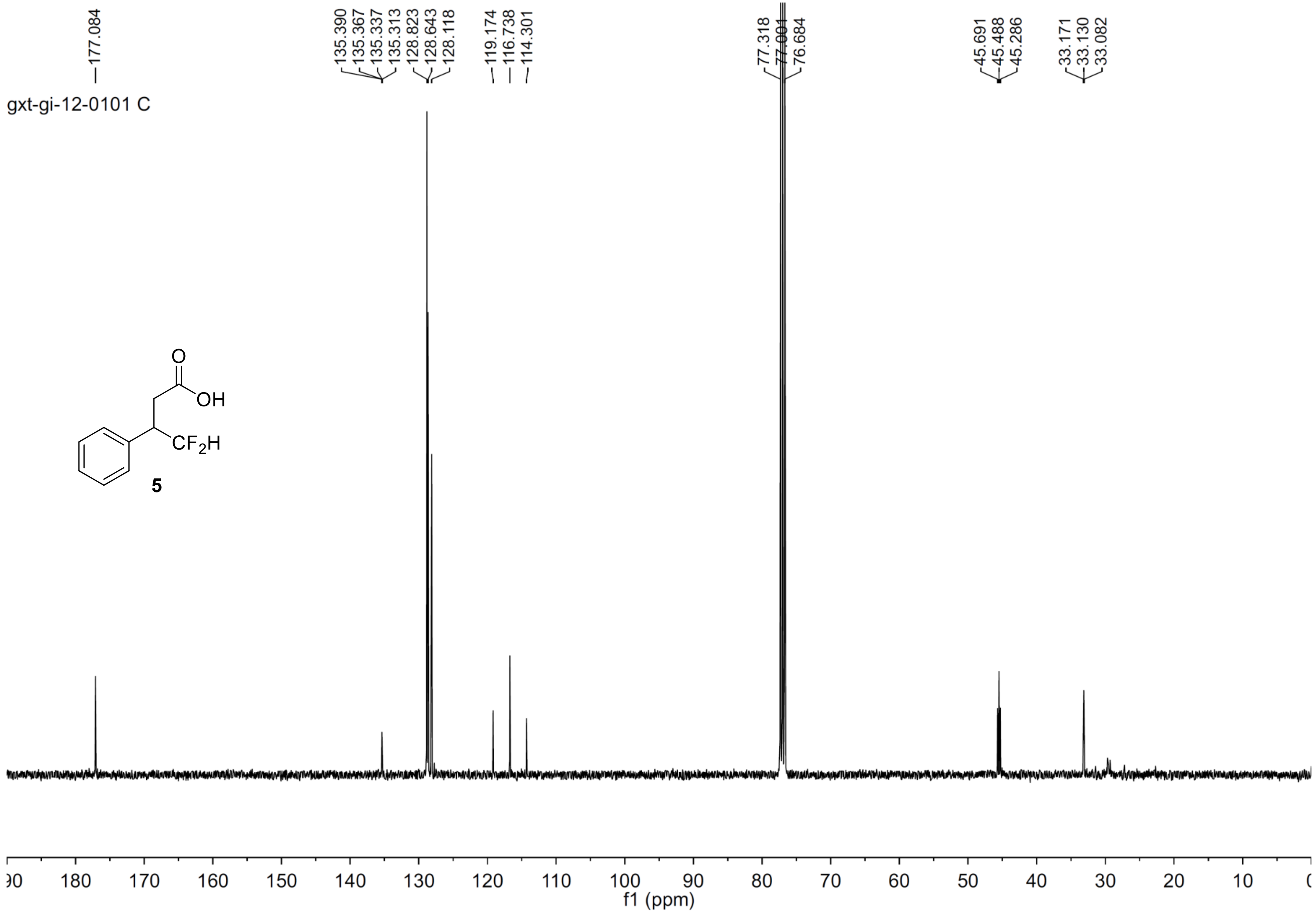
gxt-gi-12-0101 C
177.084
135.390
135.367
135.337
135.313
128.823
128.643
128.118
119.174
116.738
114.301
77.318
77.001
76.684
45.691
45.488
45.286
33.171
33.130
33.082
O
OH
CF2H
5
f1 (ppm)

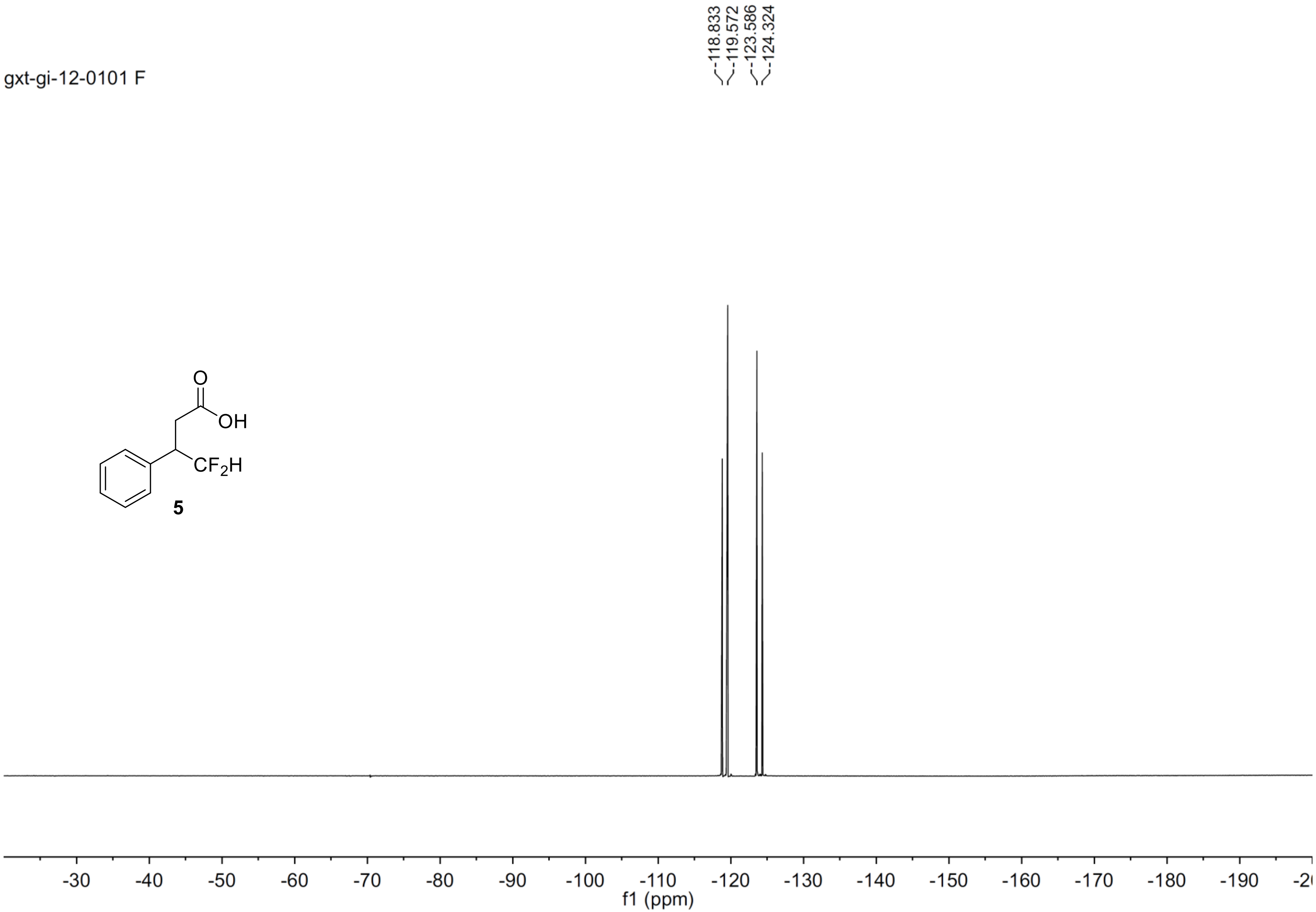
gxt-gi-12-0101 F
-118.833
-119.572
-123.586
-124.324
OH
O
CF2H
5
-30
-40
-50
-60
-70
-80
-90
-100
-110
-120
-130
-140
-150
-160
-170
-180
-190
f1 (ppm)

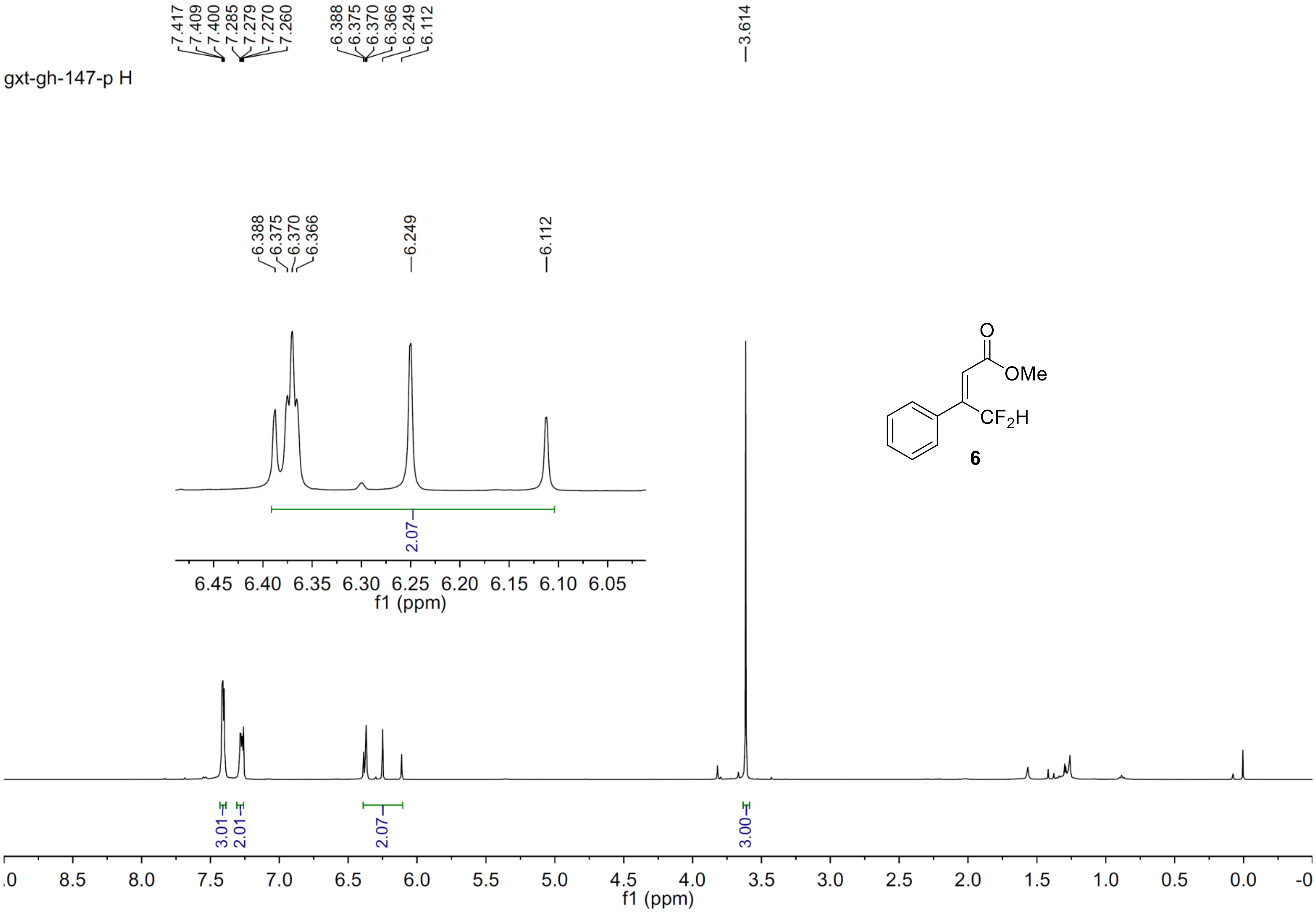
gxt-gh-147-p H
7.417
7.409
7.400
7.285
7.279
7.270
7.260
6.388
6.375
6.370
6.366
6.249
6.112
3.614
OMe
CF2H
6
2.07
6.45 6.40 6.35 6.30 6.25 6.20 6.15 6.10 6.05
f1 (ppm)
3.01
2.01
2.07
3.00
.0 8.5 8.0 7.5 7.0 6.5 6.0 5.5 5.0 4.5 4.0 3.5 3.0 2.5 2.0 1.5 1.0 0.5 0.0 -0
f1 (ppm)

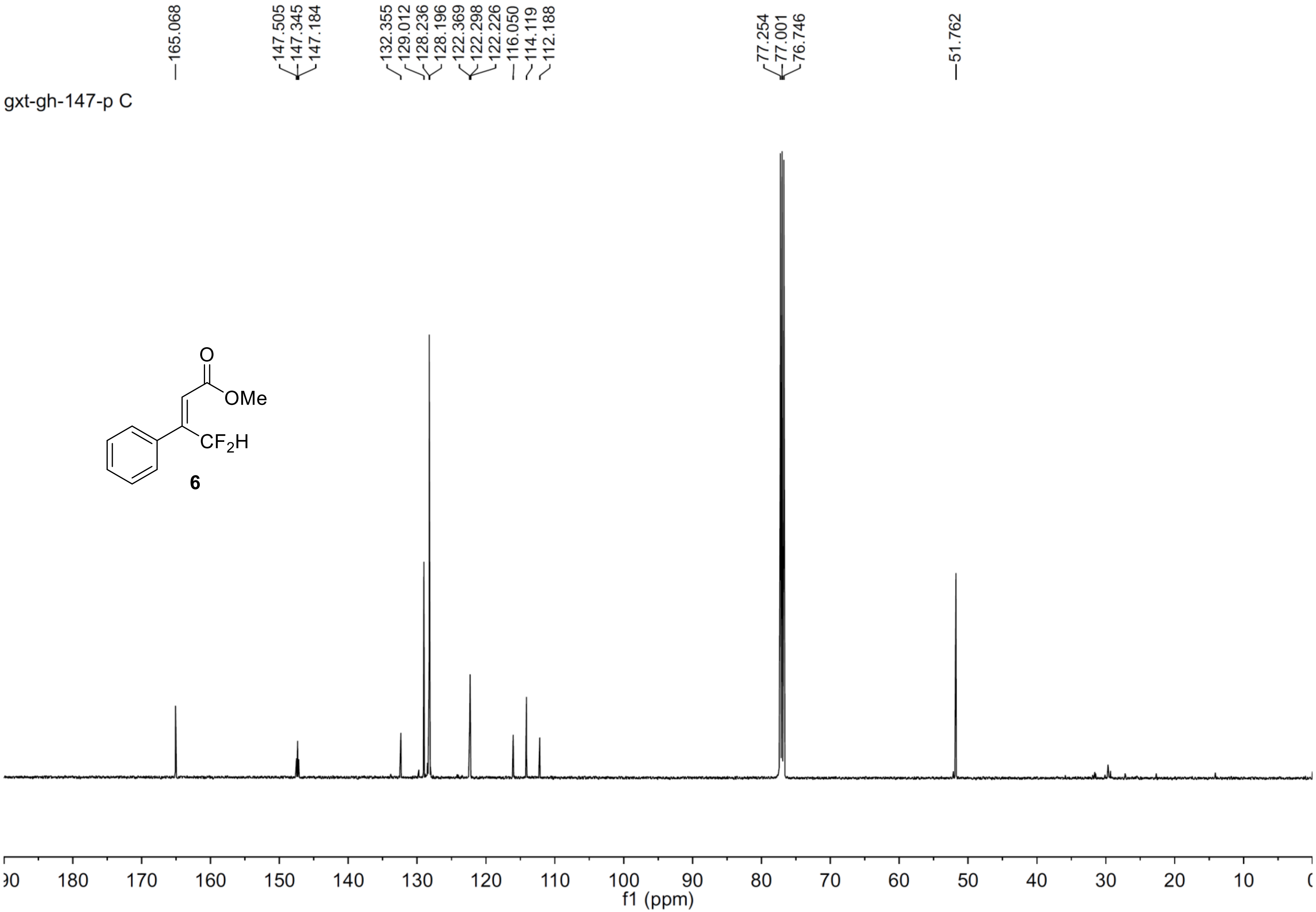
gxt-gh-147-p C
165.068
147.505
147.345
147.184
132.355
129.012
128.236
128.196
122.369
122.298
122.226
116.050
114.119
112.188
77.254
77.001
76.746
51.762
O
OMe
CF2H
6
180
170
160
150
140
130
120
110
100
90
80
70
60
50
40
30
20
10
f1 (ppm)

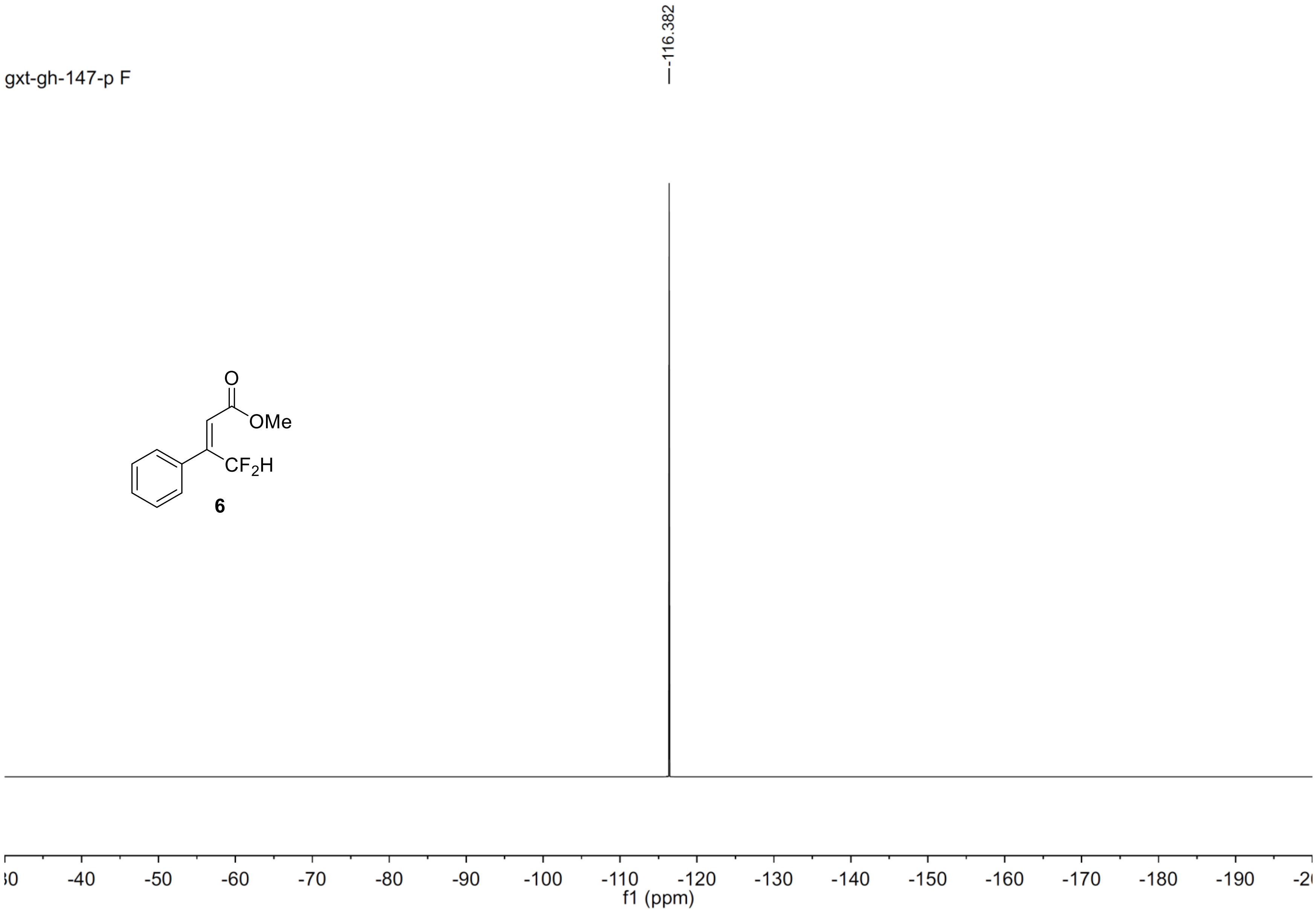
gxt-gh-147-p F
-116.382
O
OMe
CF2H
6
-40
-50
-60
-70
-80
-90
-100
-110
-120
-130
-140
-150
-160
-170
-180
-190
f1 (ppm)

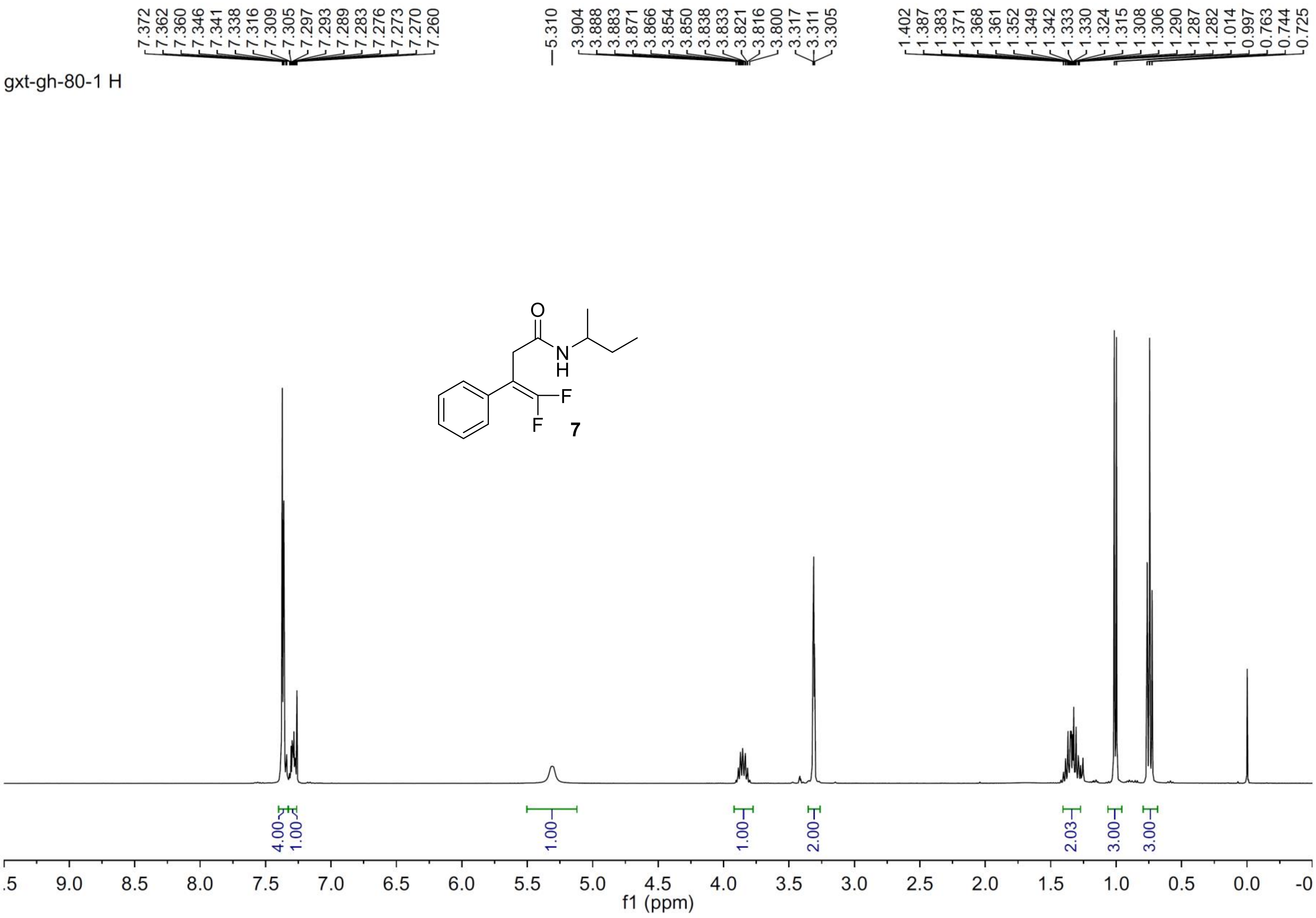
gxt-gh-80-1 H
7.372
7.362
7.360
7.346
7.341
7.338
7.316
7.309
7.305
7.297
7.293
7.289
7.283
7.276
7.273
7.270
7.260
5.310
3.904
3.888
3.883
3.871
3.866
3.854
3.850
3.838
3.833
3.821
3.816
3.800
3.317
3.311
3.305
1.402
1.387
1.383
1.371
1.368
1.361
1.352
1.349
1.342
1.333
1.330
1.324
1.315
1.308
1.306
1.290
1.287
1.282
1.014
0.997
0.763
0.744
0.725
O
N
H
F
F
7
4.00
1.00
1.00
1.00
2.00
2.03
3.00
3.00
f1 (ppm)

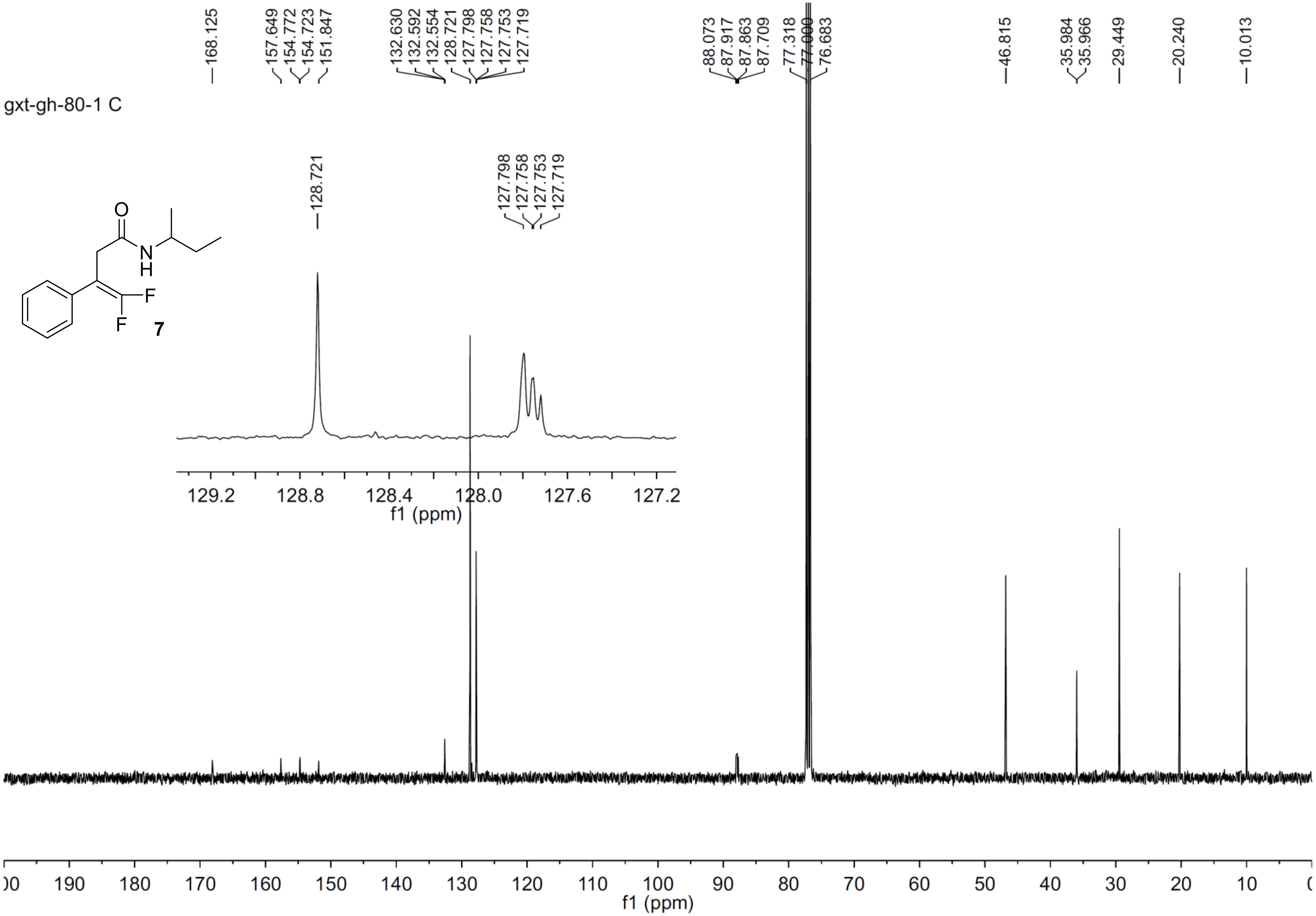
gxt-gh-80-1 C
168.125
157.649
154.772
154.723
151.847
132.630
132.592
132.554
128.721
127.798
127.758
127.753
127.719
88.073
87.917
87.863
87.709
77.318
77.000
76.683
46.815
35.984
35.966
29.449
20.240
10.013
O
N
H
F
F
7
129.2
128.8
128.4
128.0
127.6
127.2
f1 (ppm)
190
180
170
160
150
140
130
120
110
100
90
80
70
60
50
40
30
20
10
f1 (ppm)

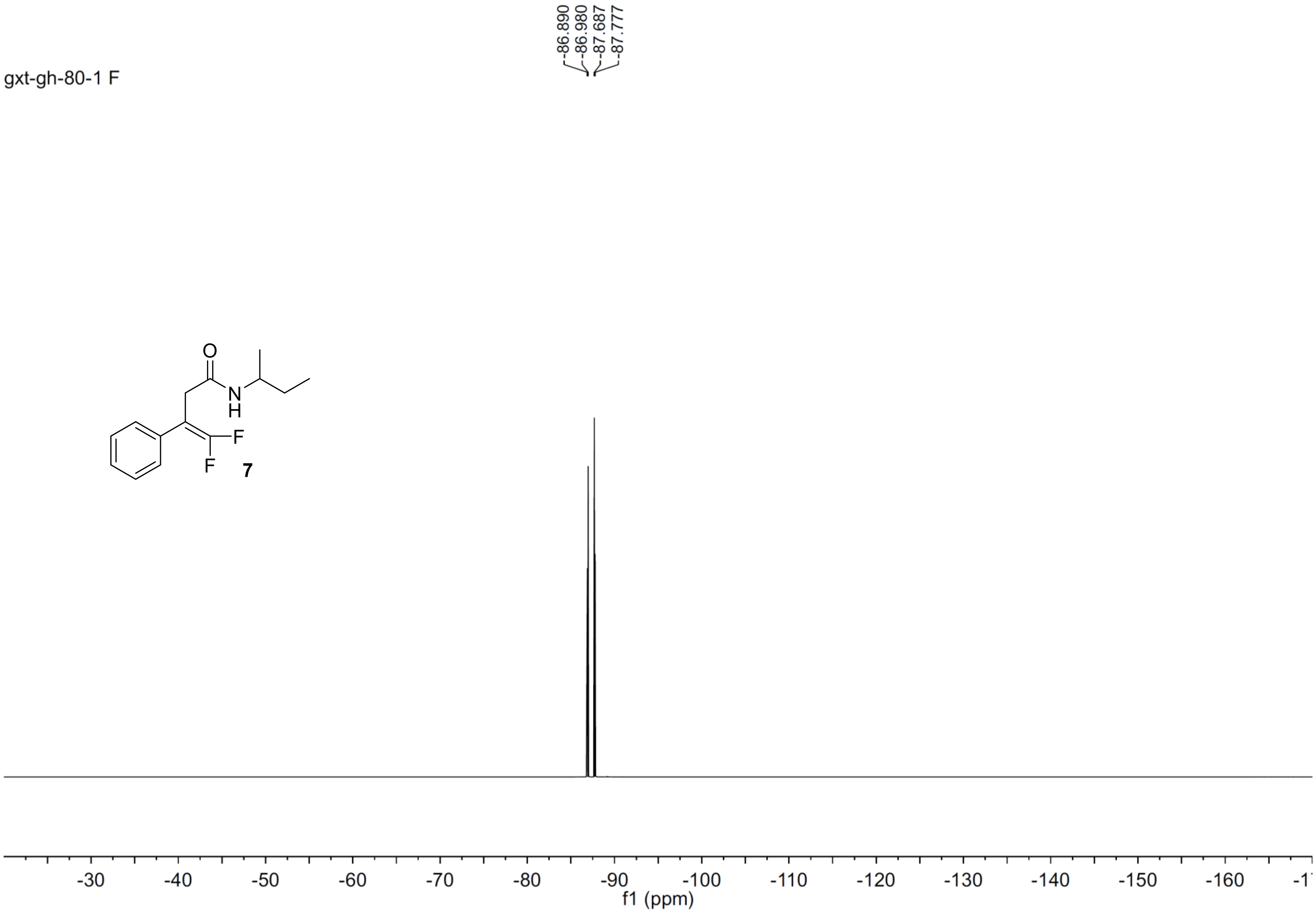
gxt-gh-80-1 F
-86.890
-86.980
-87.687
-87.777
O
N
H
F
F
7
-30
-40
-50
-60
-70
-80
-90
-100
-110
-120
-130
-140
-150
-160
f1 (ppm)

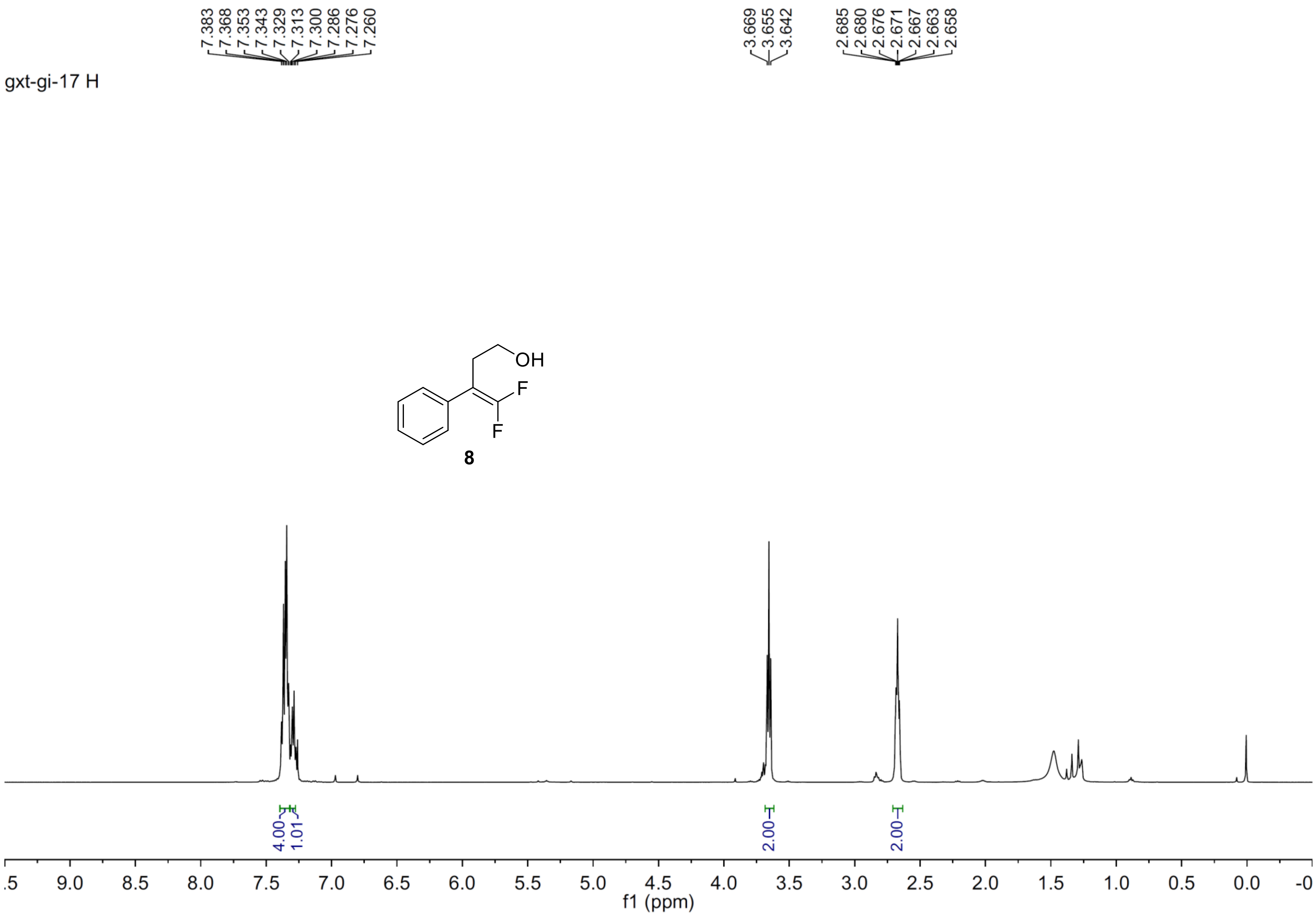
gxt-gi-17 H
7.383
7.368
7.353
7.343
7.329
7.313
7.300
7.286
7.276
7.260
3.669
3.655
3.642
2.685
2.680
2.676
2.671
2.667
2.663
2.658
OH
F
F
8
4.00
1.01
2.00
2.00
.5
9.0
8.5
8.0
7.5
7.0
6.5
6.0
5.5
5.0
4.5
4.0
3.5
3.0
2.5
2.0
1.5
1.0
0.5
0.0
-0
f1 (ppm)

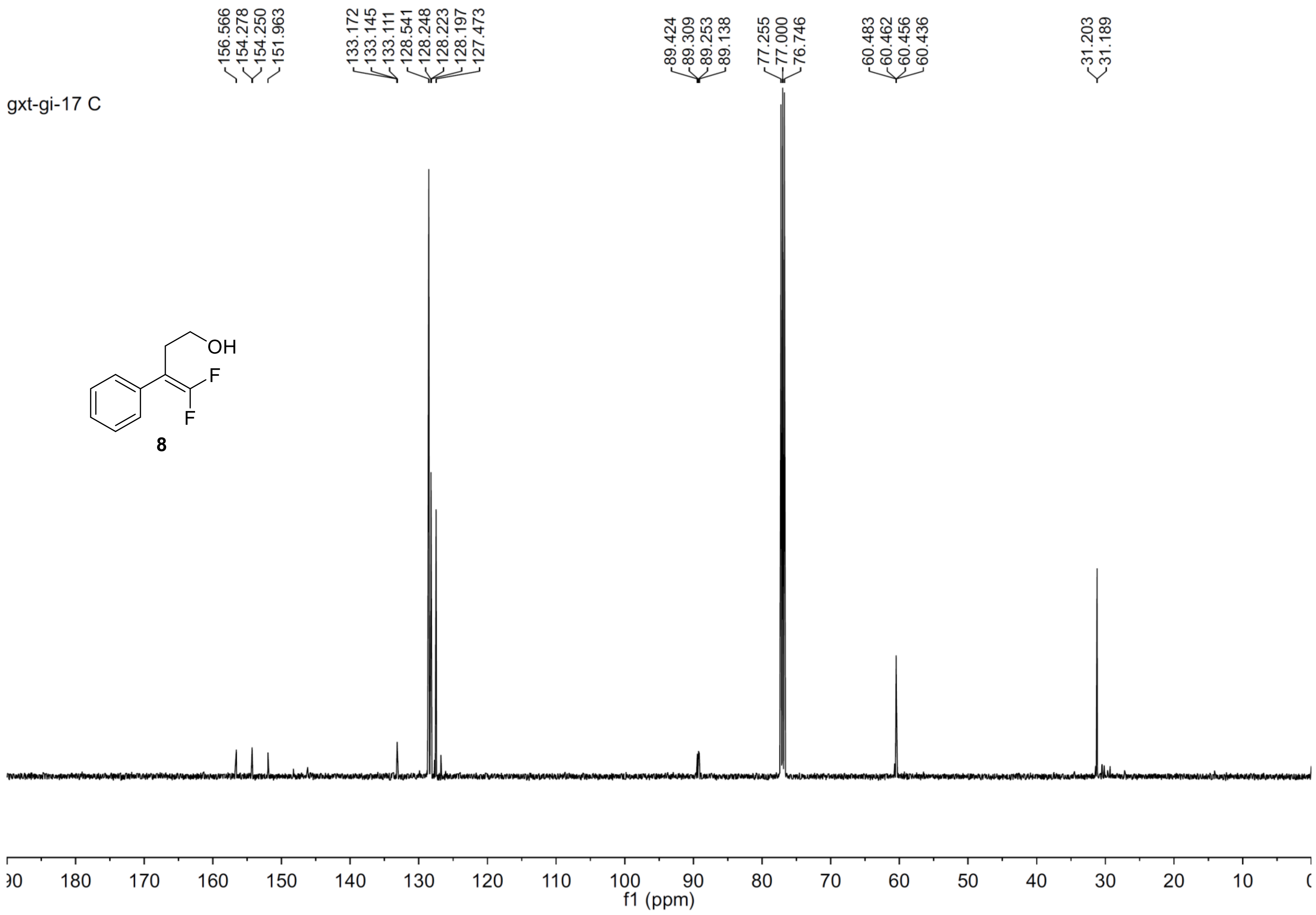

156.566
154.278
154.250
151.963
133.172
133.145
133.111
128.541
128.248
128.223
128.197
127.473
89.424
89.309
89.253
89.138
77.255
77.000
76.746
60.483
60.462
60.456
60.436
31.203
31.189
gxt-gi-17 C
OH
F
F
8
180 170 160 150 140 130 120 110 100 90 80 70 60 50 40 30 20 10
f1 (ppm)

gxt-gi-17 F

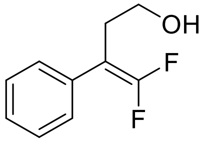

OH
F
F

8

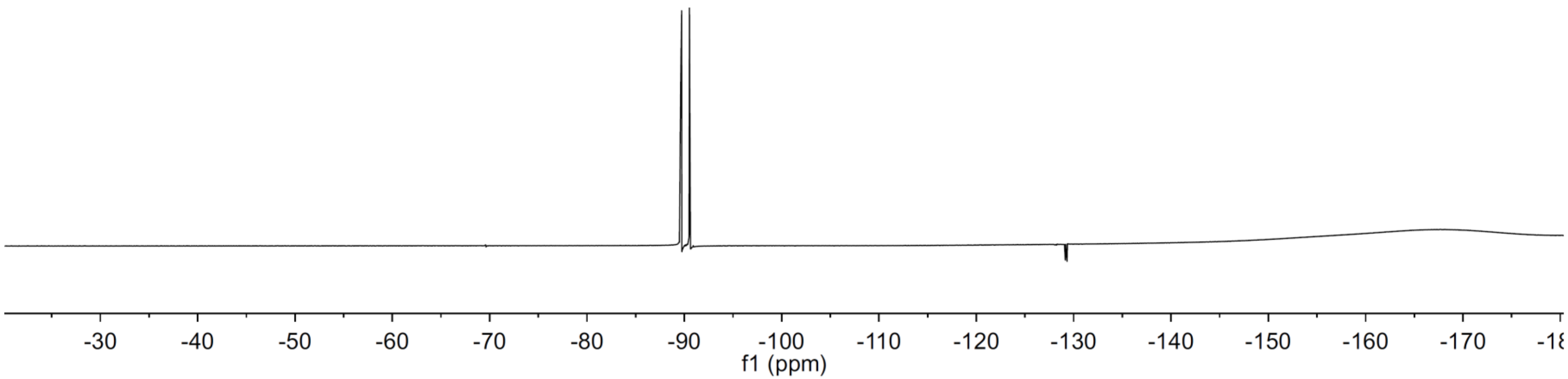

-89.638
-89.725
-90.523
-90.610
-30
-40
-50
-60
-70
-80
-90
-100
-110
-120
-130
-140
-150
-160
-170
f1 (ppm)

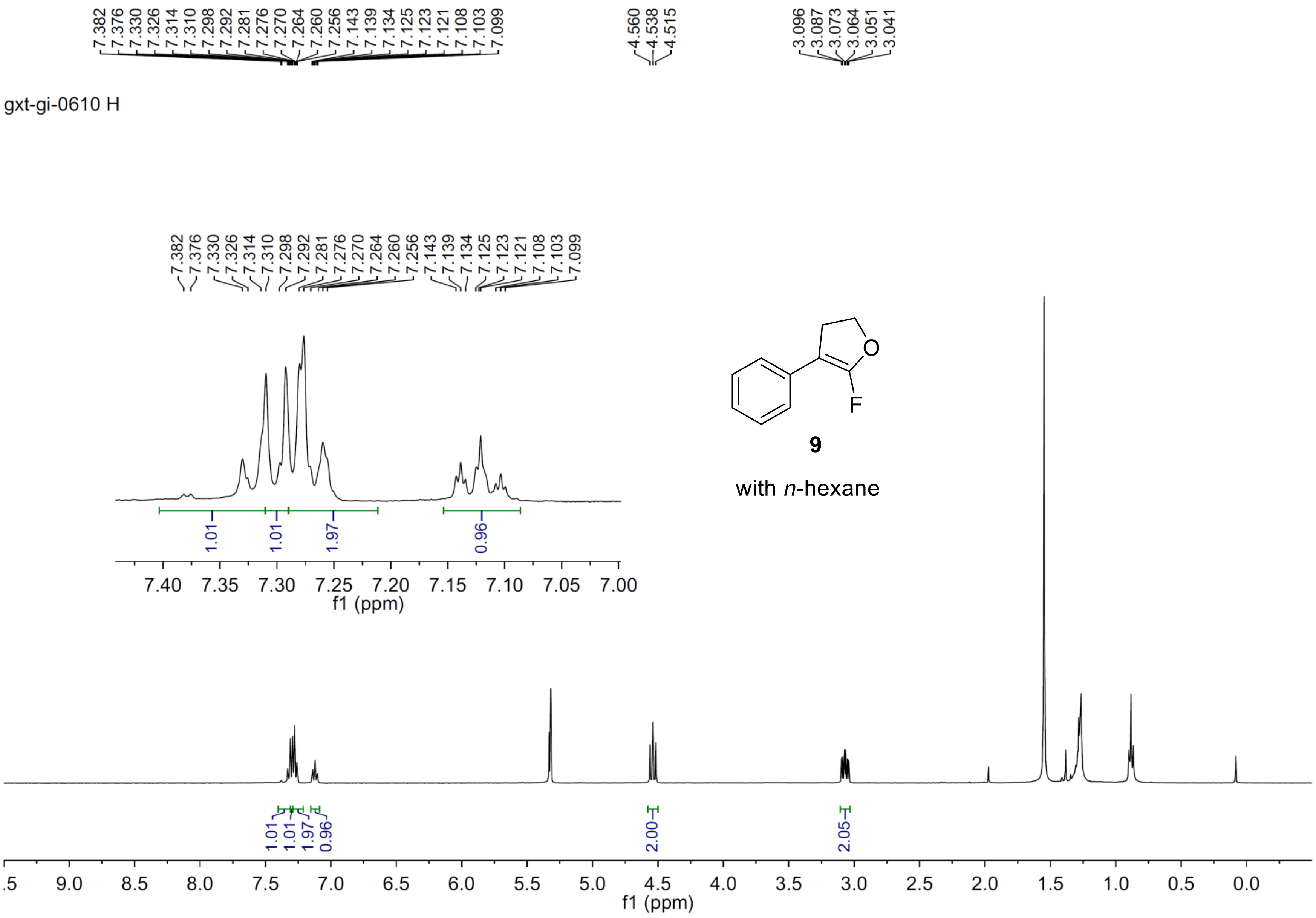
gxt-gi-0610 H
9
with *n*-hexane
f1 (ppm)

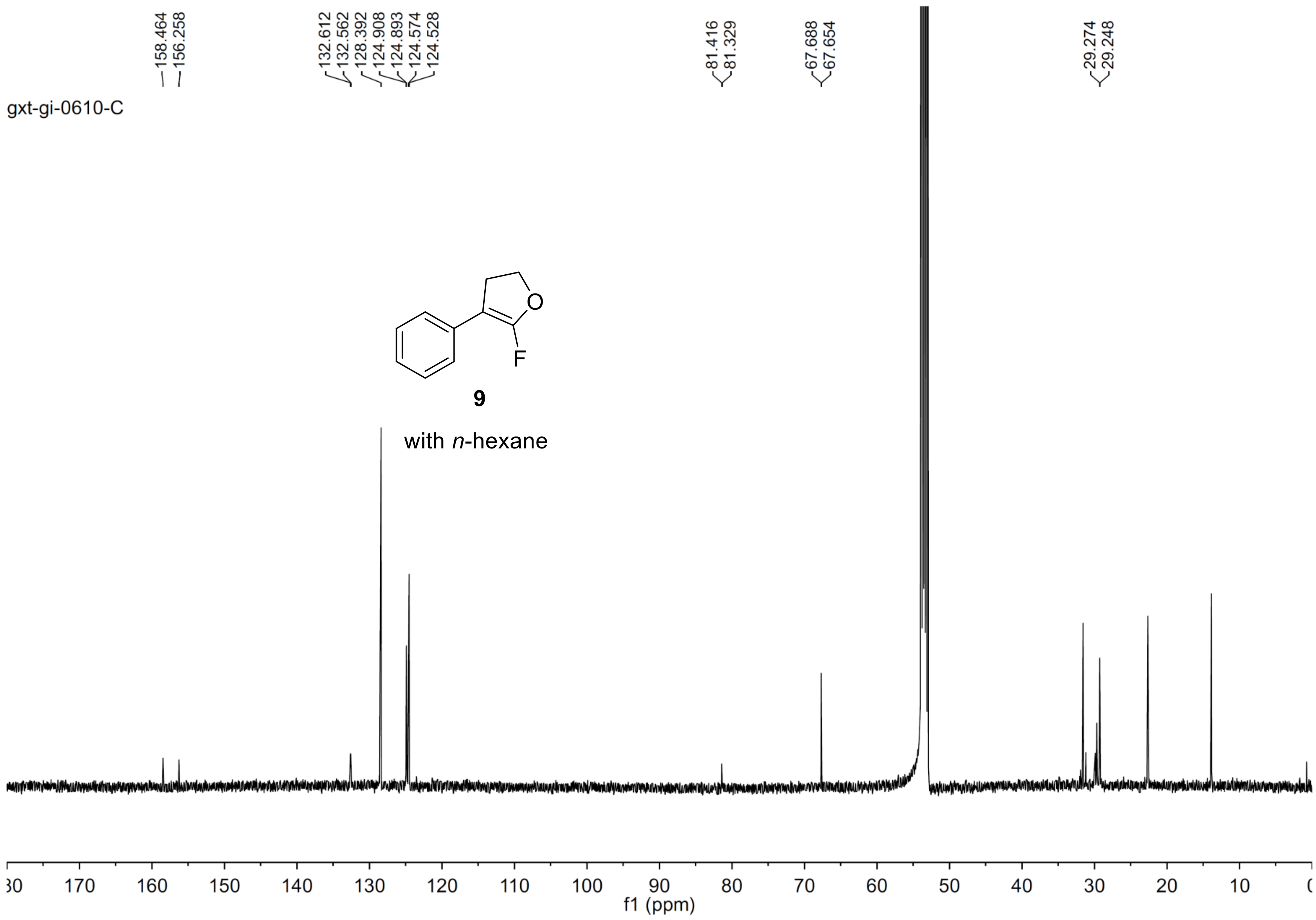
gxt-gi-0610-C
158.464
156.258
132.612
132.562
128.392
124.908
124.893
124.574
124.528
81.416
81.329
67.688
67.654
29.274
29.248
O
F
9
with n-hexane
170 160 150 140 130 120 110 100 90 80 70 60 50 40 30 20 10
f1 (ppm)

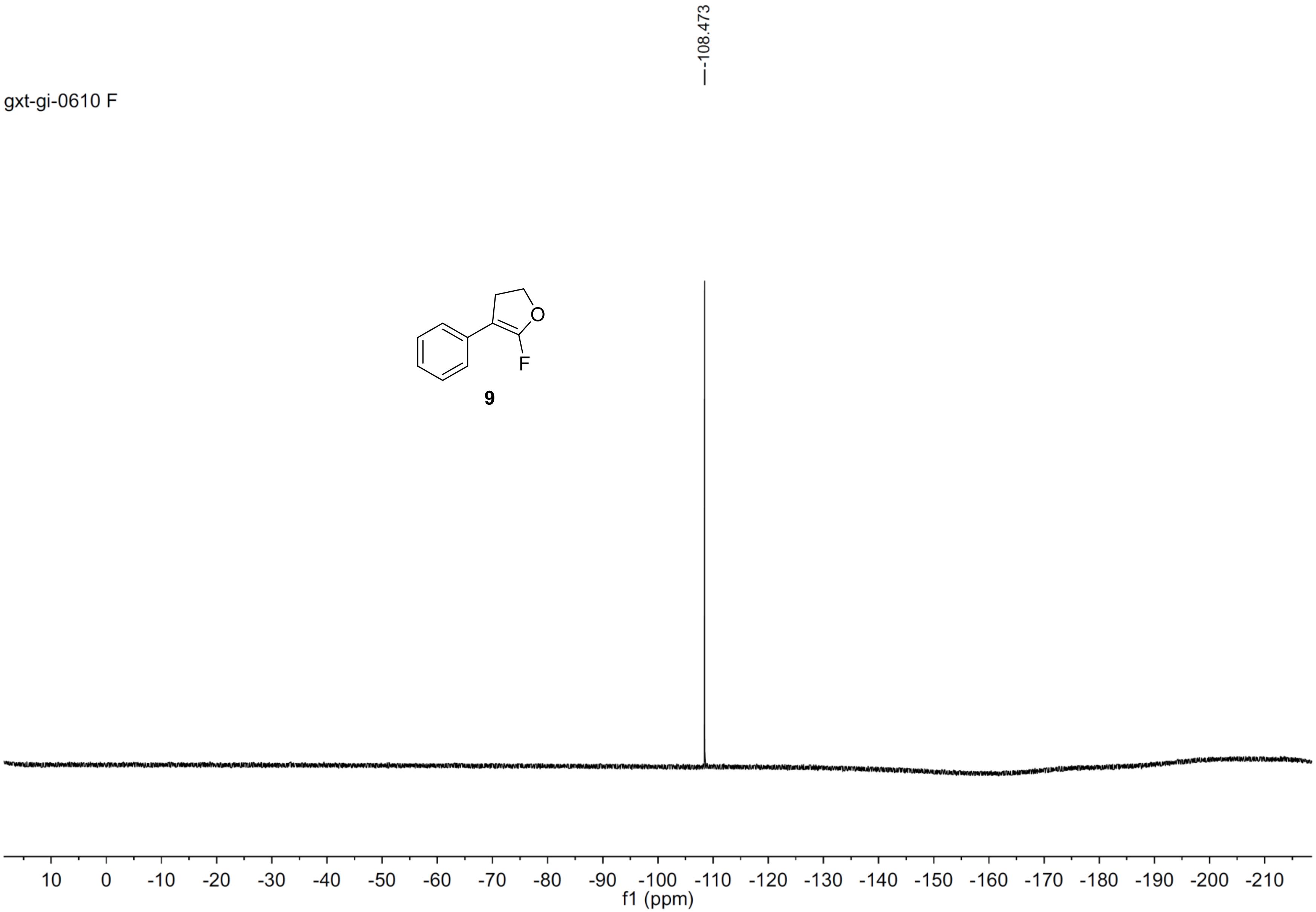
gxt-gi-0610 F
-108.473
O
F
9
10 0 -10 -20 -30 -40 -50 -60 -70 -80 -90 -100 -110 -120 -130 -140 -150 -160 -170 -180 -190 -200 -210
f1 (ppm)

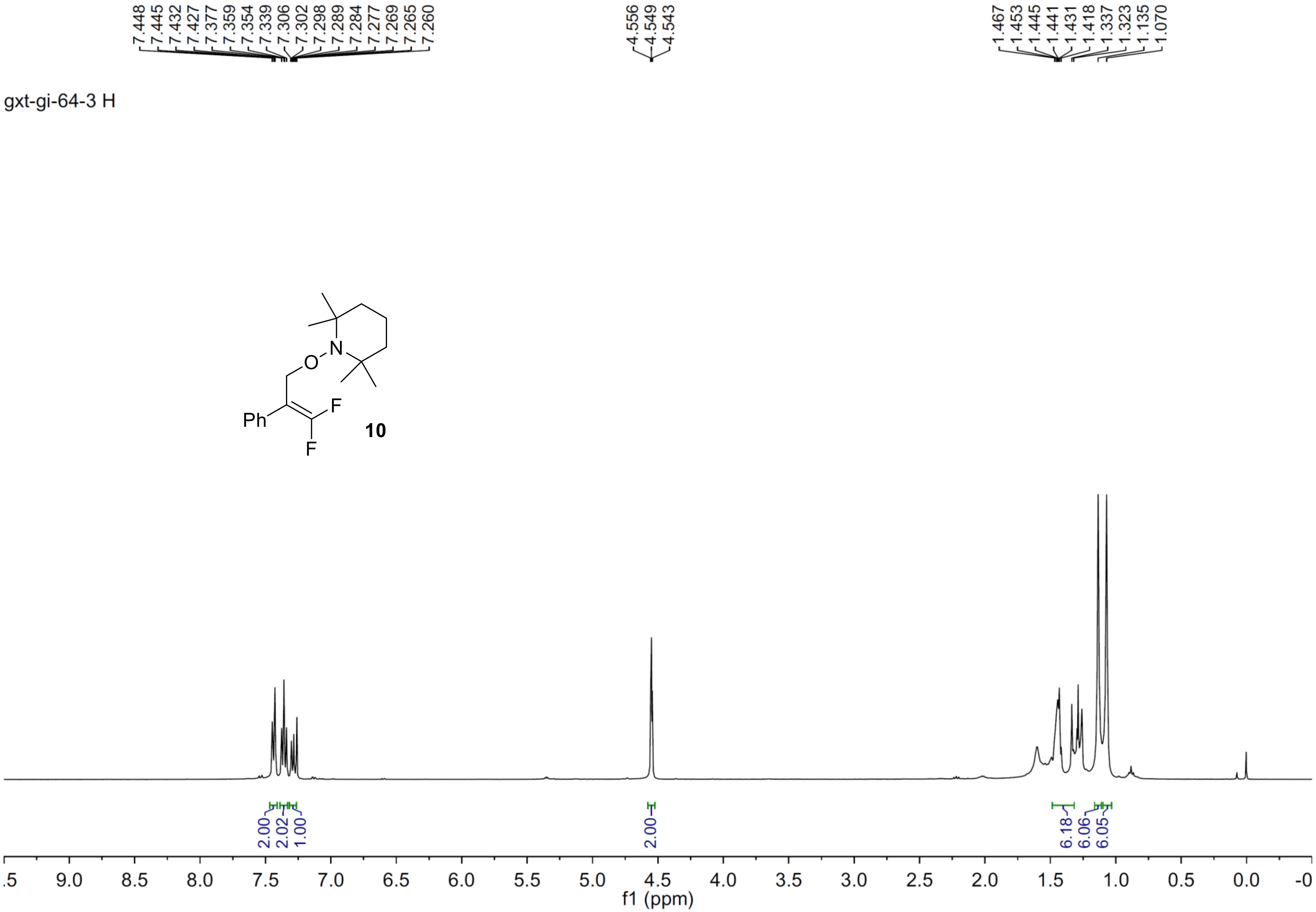
7.448 7.445 7.432 7.427 7.377 7.359 7.354 7.339 7.306 7.302 7.298 7.289 7.284 7.277 7.269 7.265 7.260
4.556 4.549 4.543
1.467 1.453 1.445 1.441 1.431 1.418 1.337 1.323 1.135 1.070
gxt-gi-64-3 H
O
N
Ph
F
F
10
2.00 2.02 1.00
2.00
6.18 6.06 6.05
.5 9.0 8.5 8.0 7.5 7.0 6.5 6.0 5.5 5.0 4.5 4.0 3.5 3.0 2.5 2.0 1.5 1.0 0.5 0.0 -0
f1 (ppm)

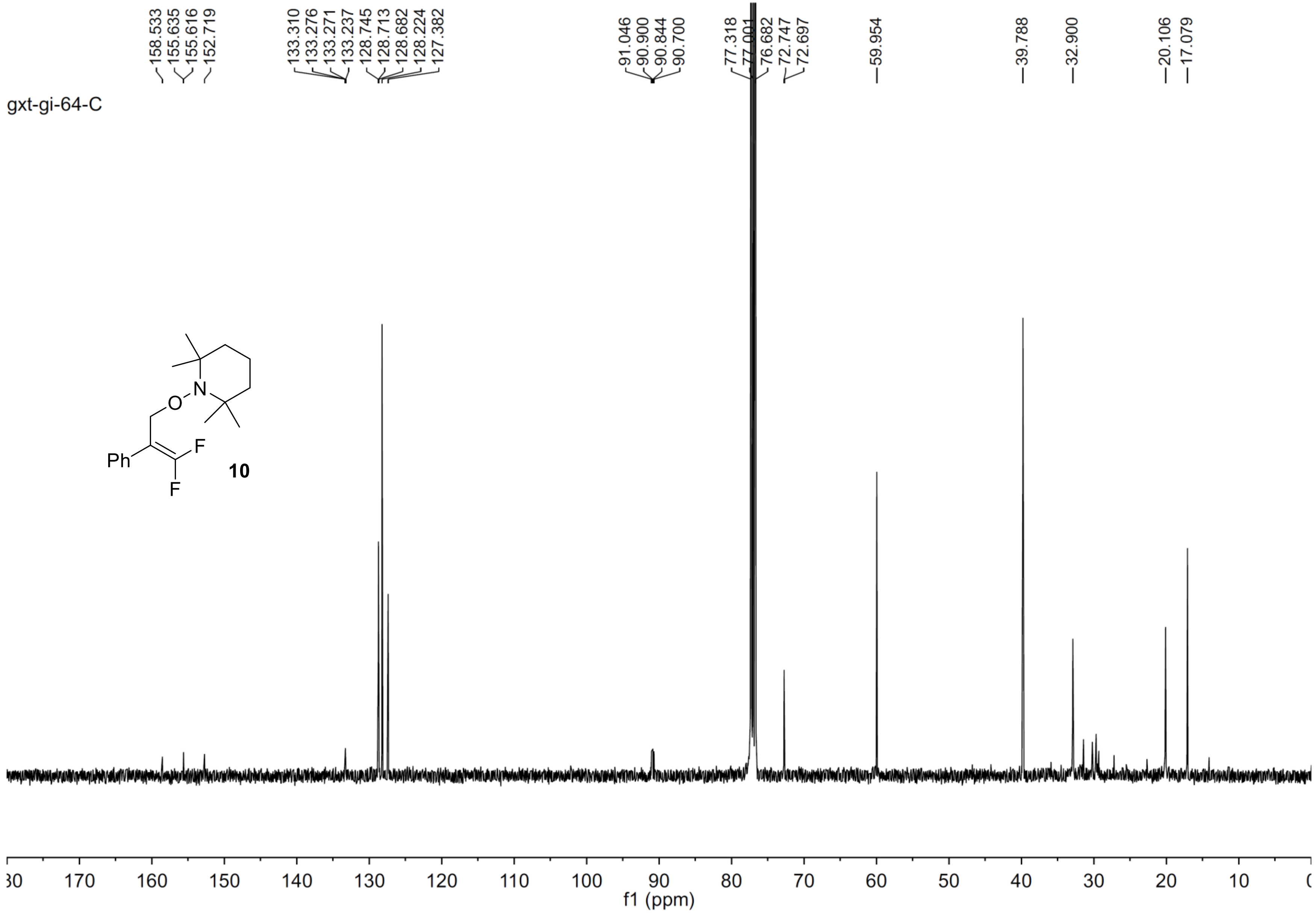
gxt-gi-64-C
158.533
155.635
155.616
152.719
133.310
133.276
133.271
133.237
128.745
128.713
128.682
128.224
127.382
91.046
90.900
90.844
90.700
77.318
77.001
76.682
72.747
72.697
59.954
39.788
32.900
20.106
17.079
N
O
Ph
F
F
10
170
160
150
140
130
120
110
100
90
80
70
60
50
40
30
20
10
f1 (ppm)

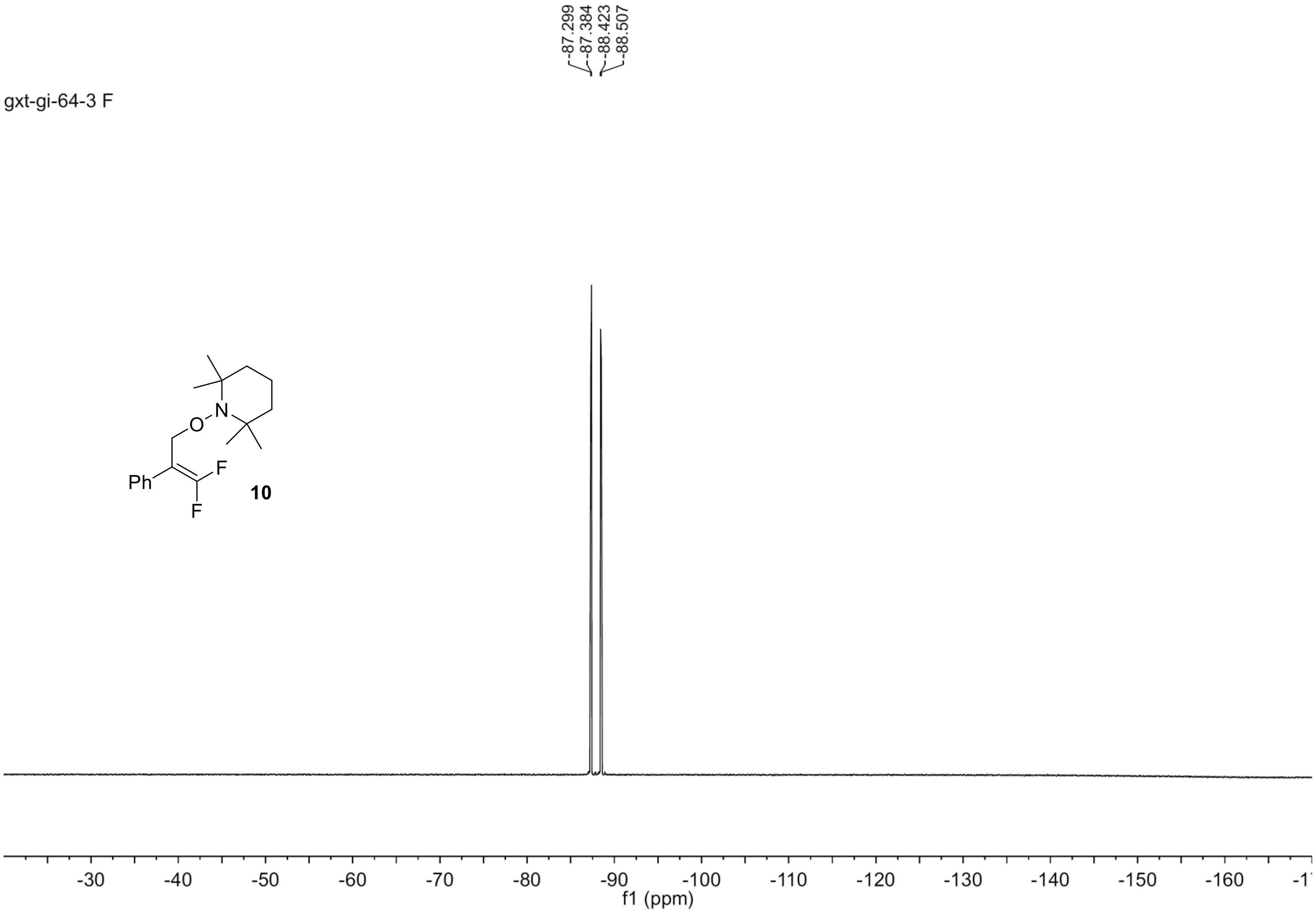
-87.299
-87.384
-88.423
-88.507
gxt-gi-64-3 F
Ph
F
F
O
N
10
-30
-40
-50
-60
-70
-80
-90
-100
-110
-120
-130
-140
-150
-160
f1 (ppm)

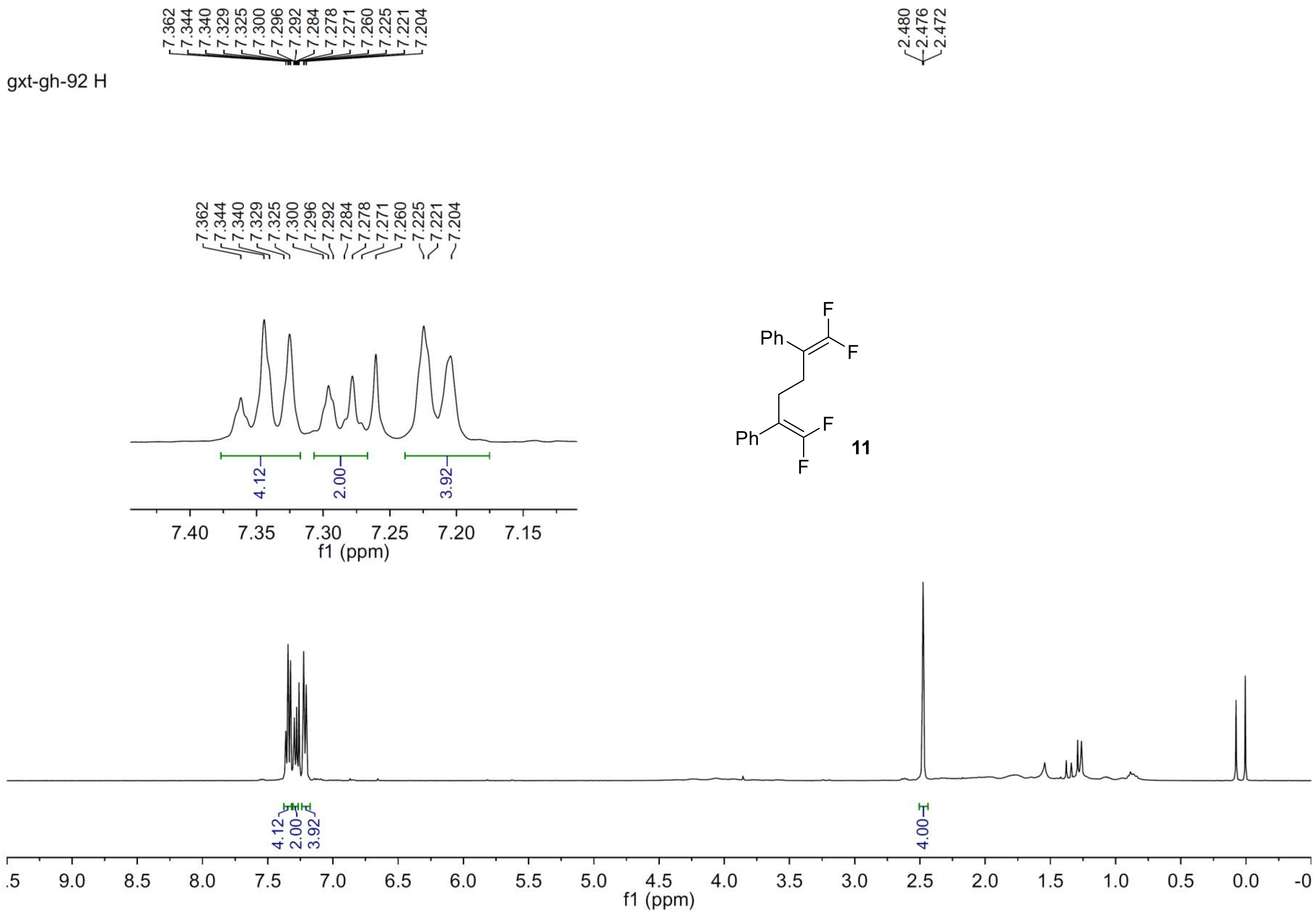
gxt-gh-92 H
7.362
7.344
7.340
7.329
7.325
7.300
7.296
7.292
7.284
7.278
7.271
7.260
7.225
7.221
7.204
2.480
2.476
2.472
4.12
2.00
3.92
7.40 7.35 7.30 7.25 7.20 7.15
f1 (ppm)
Ph
F
F
Ph
F
F
11
4.00
.5 9.0 8.5 8.0 7.5 7.0 6.5 6.0 5.5 5.0 4.5 4.0 3.5 3.0 2.5 2.0 1.5 1.0 0.5 0.0 -0
f1 (ppm)

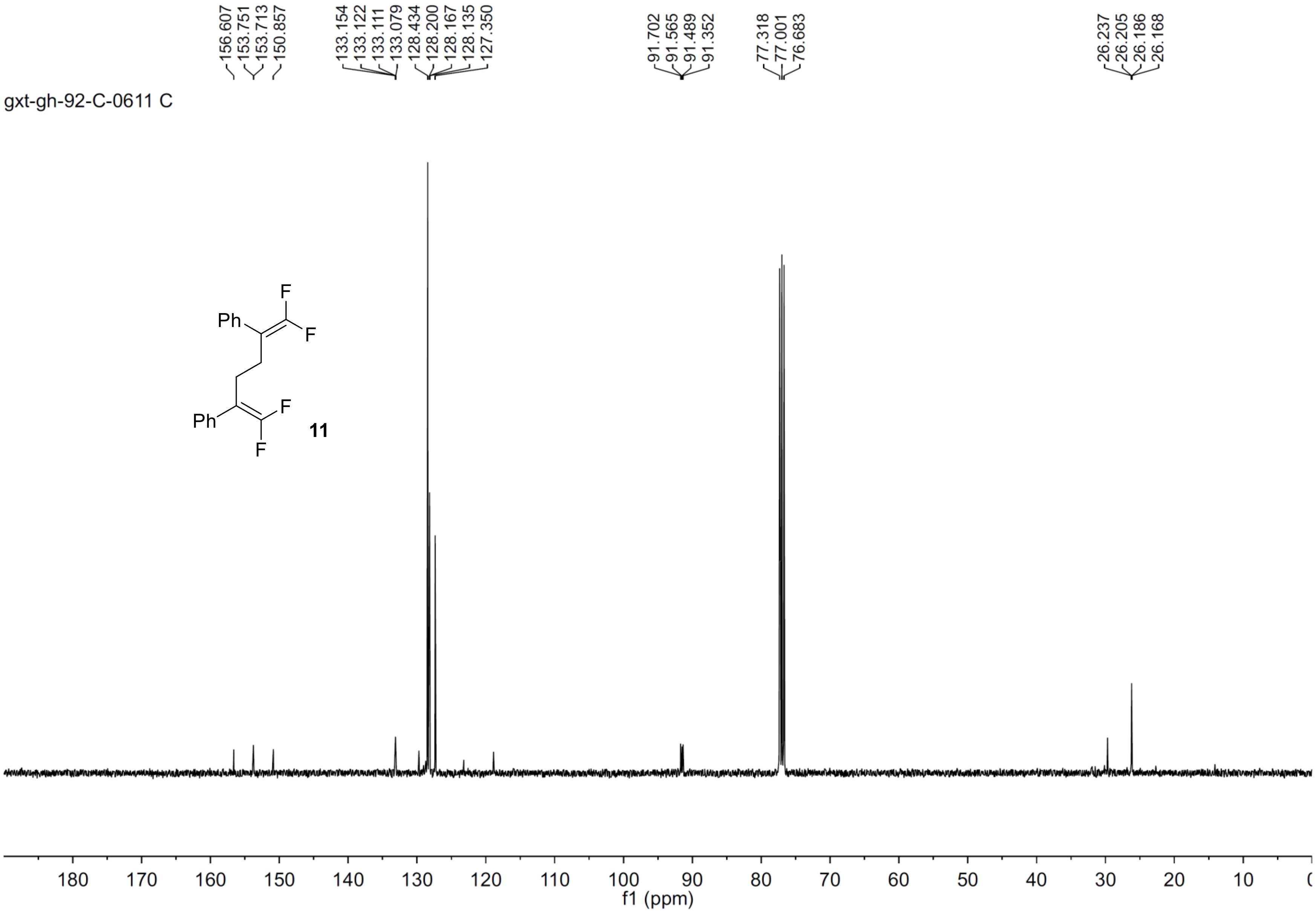
156.607
153.751
153.713
150.857
133.154
133.122
133.111
133.079
128.434
128.200
128.167
128.135
127.350
91.702
91.565
91.489
91.352
77.318
77.001
76.683
26.237
26.205
26.186
26.168
gxt-gh-92-C-0611 C
Ph
F
F
Ph
F
F
11
180
170
160
150
140
130
120
110
100
90
80
70
60
50
40
30
20
10
f1 (ppm)

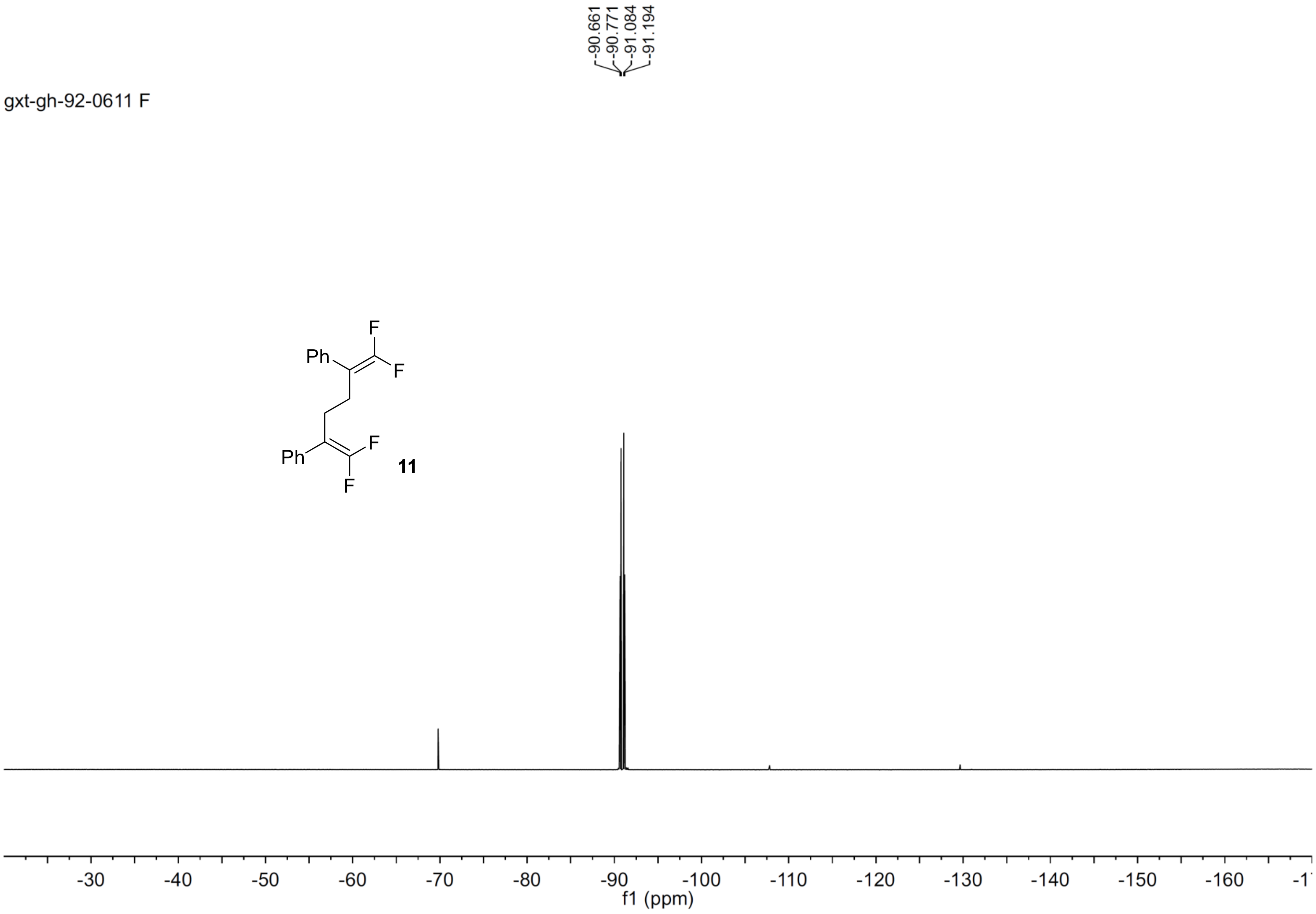
-90.661
-90.771
-91.084
-91.194
gxt-gh-92-0611 F
Ph
F
F
Ph
F
F
11
-30
-40
-50
-60
-70
-80
-90
-100
-110
-120
-130
-140
-150
-160
f1 (ppm)

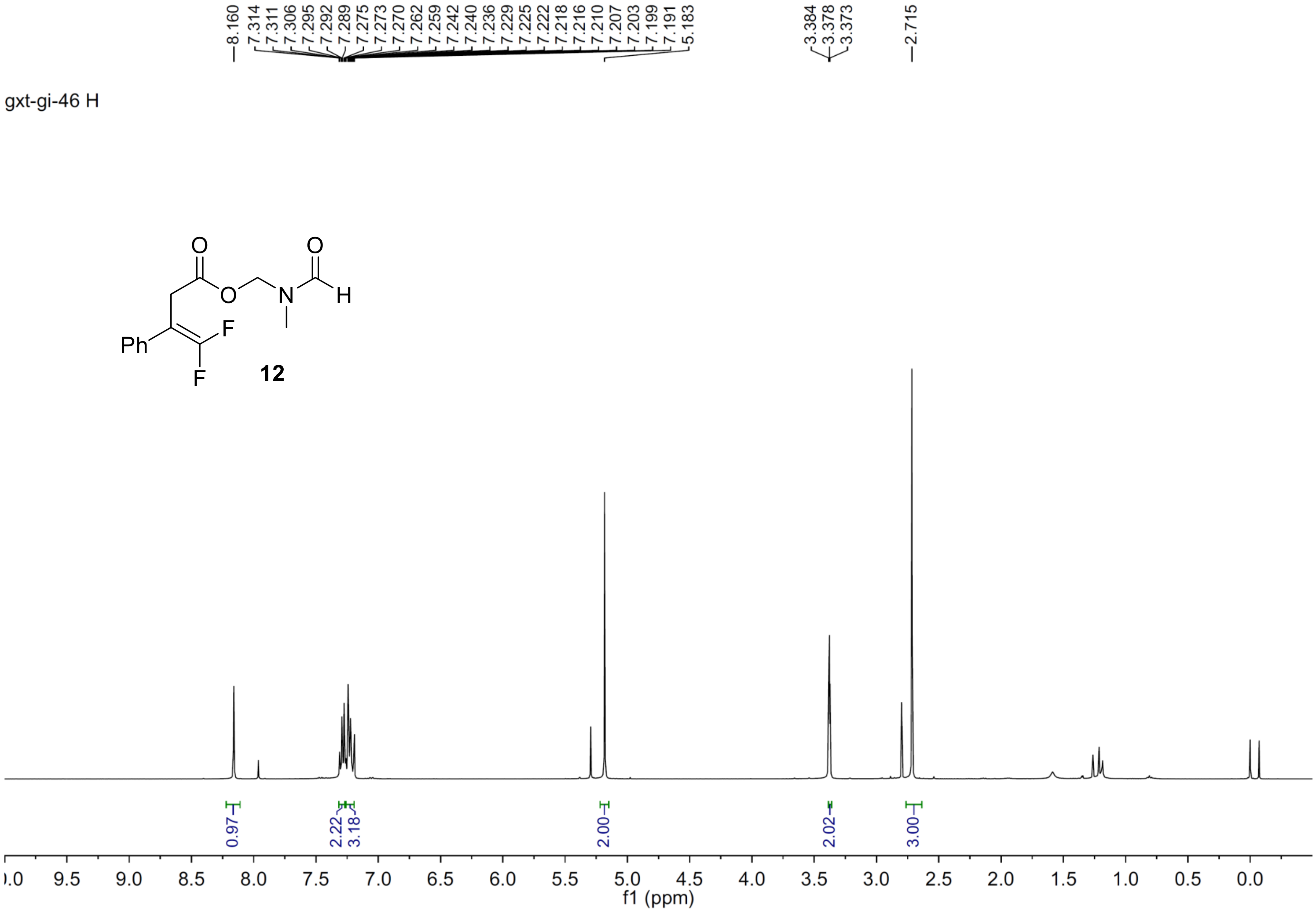
gxt-gi-46 H
8.160
7.314
7.311
7.306
7.295
7.292
7.289
7.275
7.273
7.270
7.262
7.259
7.242
7.240
7.236
7.229
7.225
7.222
7.218
7.216
7.210
7.207
7.203
7.199
7.191
5.183
3.384
3.378
3.373
2.715
Ph
F
F
O
O
N
O
H
12
0.97
2.22
3.18
2.00
2.02
3.00
f1 (ppm)

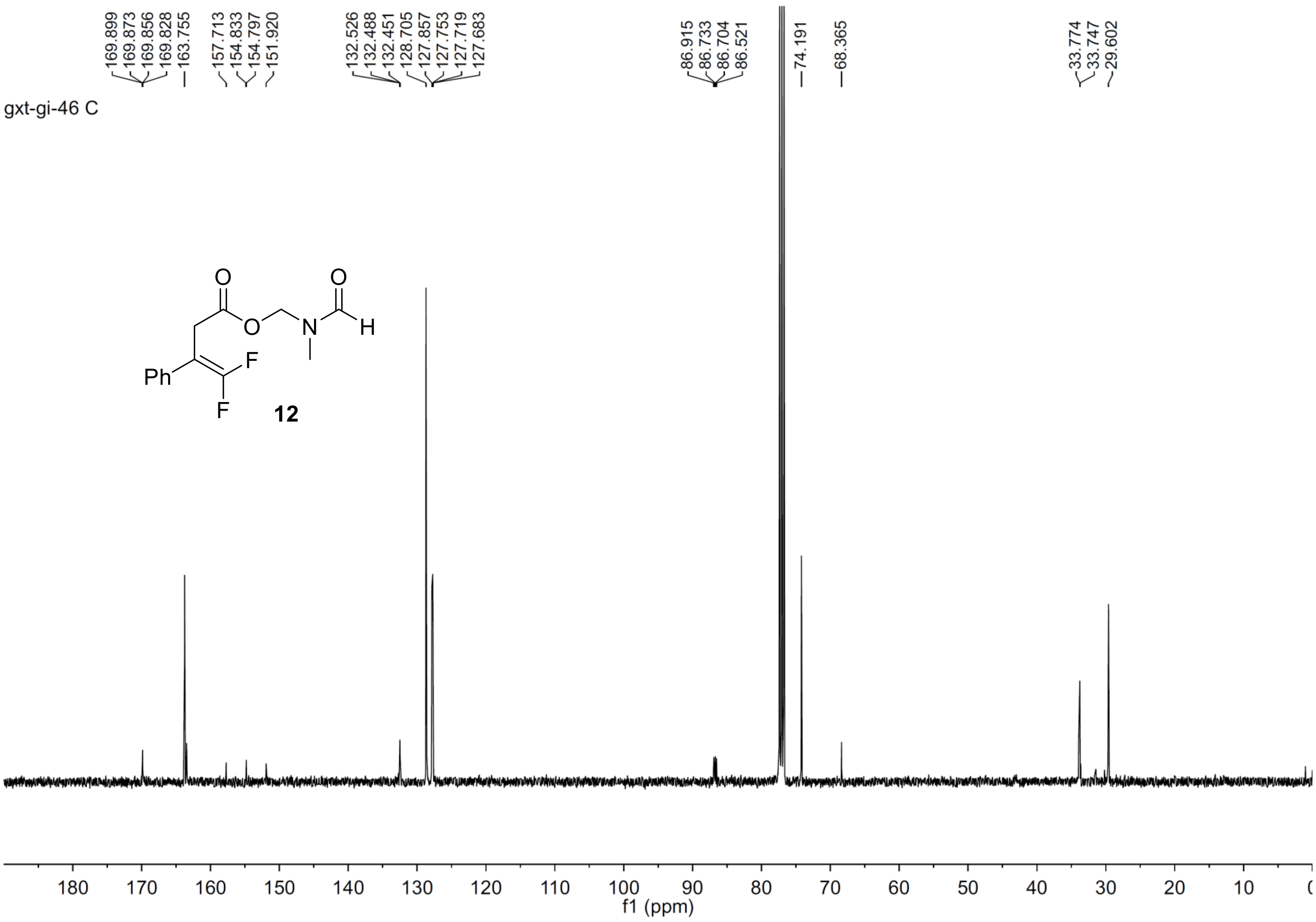
gxt-gi-46 C
169.899
169.873
169.856
169.828
163.755
157.713
154.833
154.797
151.920
132.526
132.488
132.451
128.705
127.857
127.753
127.719
127.683
86.915
86.733
86.704
86.521
74.191
68.365
33.774
33.747
29.602
O
O
O
N
H
Ph
F
F
12
180
170
160
150
140
130
120
110
100
90
80
70
60
50
40
30
20
10
f1 (ppm)

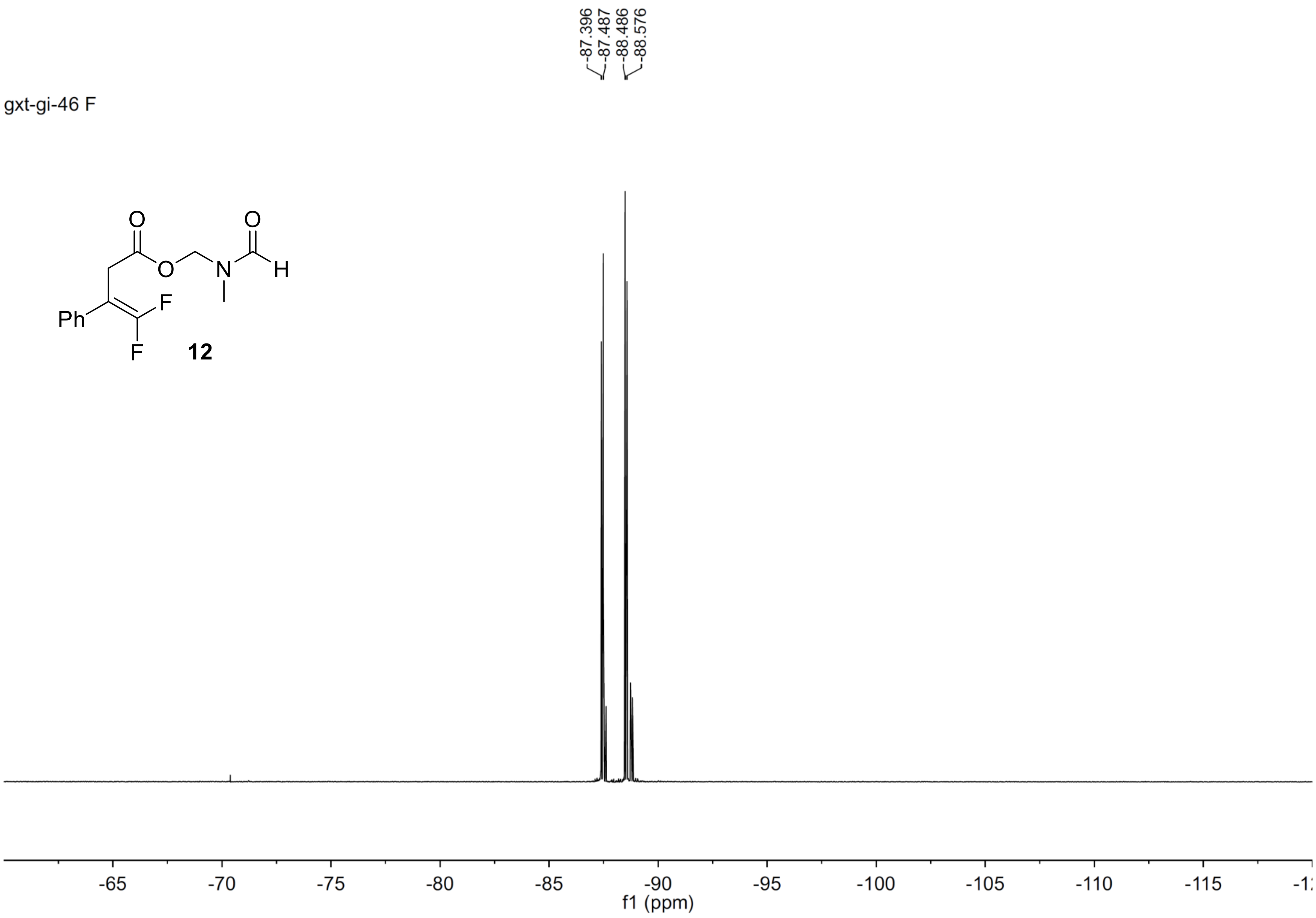

gxt-gi-46 F
-87.396
-87.487
-88.486
-88.576
O
O
O
N
H
Ph
F
F
12
-65
-70
-75
-80
-85
-90
-95
-100
-105
-110
-115
f1 (ppm)

8. References

[1] Y. Liu, Y. Zhou, Y. Zhao and J. Qu, *Org. Lett.,* 2017, **19**, 946.

[2] X.-X. Wang, T.-J. Gong, J.-J. Pi, S.-J. He and Y. Fu, *Chem. Sci.,* 2019, **10**, 809.

[3] B. M. Trost and L. Debien, *J. Am. Chem. Soc.,* 2015, **137**, 11606.

[4] C.-M. Hu, F. Hong and Y.-Y. Xu, *J. Fluorine Chem.,* **1993**, *64*, 1.

[5] M. Engman, P. Cheruku, P. Tolstoy, J. Bergquist, S. F. Völker and P. G. Andersson, *Adv. Synth. Catal.,* 2009, **351**, 375.

[6] Z. Lin, Y. Lan and C. Wang, *ACS Catal.,* 2019, **9**, 775.

[7] Y. He, D. Anand, Z. Sun and L. Zhou, *Org. Lett.,* 2019, **21**, 3769.

[8] J. J. Medvedev, X. V. Medvedeva, F. Li, T. A. Zienchuk and A. Klinkova, *ACS Sustainable Chem. Eng.,* 2019, **7**, 19631.

[9] (a) A. Alkayal, V. Tabas, S. Montanaro, I. A. Wright, A. V. Malkov and B. R. Buckley, *J. Am. Chem. Soc.,* 2020, **142**, 1780; (b) Y. Kim, G. D. Park, M. Balamurugan, J. Seo, B. K. Min and K. T. Nam, *Adv. Sci.,* 2020, **7**, 1900137.

[10] T. Nihei, N. Iwai, T. Matsuda and T. Kitazume, *J. Org. Chem.,* 2005, **70**, 5912.

[11] Y. Nakamura, M. Okada, H. Horikawa and T. Taguchi, *J. Fluorine Chem.,* 2002, **117**, 143.

[12] M. J. Frisch, et al., *Gaussian 09*, Revision A. 1, Gaussian, Inc., Wallingford, CT, 2009.

[13] Y. Zhao and D. G. Truhlar, *Theor. Chem. Acc.,* 2008, **120**, 215.

[14] (a) B. Mennucci and J. Tomasi, *J. Chem. Phys.,* 1997, **106**, 5151; (b) B. Mennucci, E. Cances and J. Tomasi, *J. Phys. Chem. B,* 1997, **101**, 10506; (c) J. Tomasi, B. Mennucci and E. Cancès, *J. Mol. Struct. (THEOCHEM)*, 1999, **464**, 211.

[15] A. E. Reed, L. A. Curtiss and F. Weinhold, *Chem. Rev.*, 1988, **88**, 899.

[16] X. Liu, R. Liu, J. Qiu, X. Cheng and G. Li, *Angew. Chem., Int. Ed.,* 2020, **59**, 13962

[17] J. J. Medvedev, X. V. Medvedeva, F. Li, T. A. Zienchuk and A. Klinkova, *ACS Sustainable Chem. Eng.,* 2019, **7**, 19631.
